# Functional Gene Array-Based Ultrasensitive and Quantitative Detection of Microbial Populations in Complex Communities

**DOI:** 10.1128/mSystems.00296-19

**Published:** 2019-06-18

**Authors:** Zhou Shi, Huaqun Yin, Joy D. Van Nostrand, James W. Voordeckers, Qichao Tu, Ye Deng, Mengting Yuan, Aifen Zhou, Ping Zhang, Naijia Xiao, Daliang Ning, Zhili He, Liyou Wu, Jizhong Zhou

**Affiliations:** a Institute for Environmental Genomics and Department of Microbiology and Plant Biology, University of Oklahoma, Norman, Oklahoma, USA; b Key Laboratory of Biometallurgy of Ministry of Education, School of Minerals Processing and Bioengineering, Central South University, Changsha, China; c Institute of Marine Science and Technology, Shandong University, Qingdao, China; d School of Environmental Science and Engineering, Environmental Microbiomics Research Center, Sun Yat-sen University, Guangzhou, China; e Earth and Environmental Sciences, Lawrence Berkeley National Laboratory, Berkeley, California, USA; f School of Environment, Tsinghua University, Beijing, China; Institute for Systems Biology

**Keywords:** functional gene array, microarrays, microbial communities

## Abstract

While functional gene arrays (FGAs) have greatly expanded our understanding of complex microbial systems, specificity, sensitivity, and quantitation challenges remain. We developed a new generation of FGA, GeoChip 5.0, using the Agilent platform. Two formats were created, a smaller format (GeoChip 5.0S), primarily covering carbon-, nitrogen-, sulfur-, and phosphorus-cycling genes and others providing ecological services, and a larger format (GeoChip 5.0M) containing the functional categories involved in biogeochemical cycling of C, N, S, and P and various metals, stress response, microbial defense, electron transport, plant growth promotion, virulence, *gyrB*, and fungus-, protozoan-, and virus-specific genes. GeoChip 5.0M contains 161,961 oligonucleotide probes covering >365,000 genes of 1,447 gene families from broad, functionally divergent taxonomic groups, including bacteria (2,721 genera), archaea (101 genera), fungi (297 genera), protists (219 genera), and viruses (167 genera), mainly phages. Computational and experimental evaluation indicated that designed probes were highly specific and could detect as little as 0.05 ng of pure culture DNAs within a background of 1 μg community DNA (equivalent to 0.005% of the population). Additionally, strong quantitative linear relationships were observed between signal intensity and amount of pure genomic (∼99% of probes detected; *r* > 0.9) or soil (∼97%; *r* > 0.9) DNAs. Application of the GeoChip to a contaminated groundwater microbial community indicated that environmental contaminants (primarily heavy metals) had significant impacts on the biodiversity of the communities. This is the most comprehensive FGA to date, capable of directly linking microbial genes/populations to ecosystem functions.

**IMPORTANCE** The rapid development of metagenomic technologies, including microarrays, over the past decade has greatly expanded our understanding of complex microbial systems. However, because of the ever-expanding number of novel microbial sequences discovered each year, developing a microarray that is representative of real microbial communities, is specific and sensitive, and provides quantitative information remains a challenge. The newly developed GeoChip 5.0 is the most comprehensive microarray available to date for examining the functional capabilities of microbial communities important to biogeochemistry, ecology, environmental sciences, and human health. The GeoChip 5 is highly specific, sensitive, and quantitative based on both computational and experimental assays. Use of the array on a contaminated groundwater sample provided novel insights on the impacts of environmental contaminants on groundwater microbial communities.

## Abstract (precis)

The rapid development of metagenomic technologies, including microarrays, over the past decade has greatly expanded our understanding of complex microbial systems. However, because of the ever-expanding number of novel microbial sequences discovered each year, developing a microarray that is representative of real microbial communities, is specific and sensitive, and provides quantitative information remains a challenge. The newly developed GeoChip 5.0 is the most comprehensive microarray available to date for examining the functional capabilities of microbial communities important to biogeochemistry, ecology, environmental sciences, and human health. The GeoChip 5 is highly specific, sensitive, and quantitative based on both computational and experimental assays. Use of the array on a contaminated groundwater sample provided novel insights on the impacts of environmental contaminants on groundwater microbial communities.

## INTRODUCTION

Microorganisms are the most diverse and ubiquitous life on earth. They are integral to ecosystem processes and functions of critical importance in global biogeochemical cycling, climate change, environmental remediation, engineering, and agriculture (1, 2). Despite their importance, determining microbial community structure and functions remains challenging for several reasons. First, microbial diversity is extremely high in most environments. For example, 1 g of soil could contain 2,000 to 8.3 million species (3–5), a majority of which (>99%) have not been cultivated (6). The number of microbial cells within environmental habitats is also extremely large. Microbial cell numbers have been estimated to be 1.2 × 10^29^ in the open ocean (7), 2.9 × 10^29^ in subseafloor sediment (8), and 2.6 × 10^29^ in soil (7). These communities also represent a high diversity of functional potential (9). Establishing mechanistic linkages between microbial biodiversity and ecosystem functioning poses another grand challenge for microbiome research.

Several types of high-throughput technologies have been developed to characterize microbial communities, including next-generation sequencing (10–15), microarrays (16–19), and quantitative PCR (20–22). These technologies have provided unprecedented insights into microbial biodiversity and allowed for the detection of novel processes and functions (23). Among these, high-throughput sequencing and microarrays are two of the most widely used (24), with distinct differences in susceptibility to random sampling and nontarget DNA errors, detection of novel and rare species, quantitation, and data analysis (24). Consequently, both have unique advantages and disadvantages in terms of detection specificity, sensitivity, quantification, and reproducibility (24). It is highly beneficial if both types of technologies are used in a complementary fashion to address fundamental questions in microbial ecology (24).

Over the last few decades, a variety of DNA microarray-based technologies have been developed for microbial detection and community analysis (25). Phylogenetic gene arrays contain probes from phylogenetic markers such as rRNA genes, which are useful for identifying specific taxa and studying phylogenetic relationships. Functional gene arrays (FGAs) target genes involved in various functional processes (24) and are valuable for assessing the functional composition and structure of microbial communities. Although various types of FGAs are available (24), GeoChip, a generic FGA targeting hundreds of functional gene categories important to biogeochemical, ecological, and environmental analyses, is the most widely used. GeoChip has been shown to be an effective, sensitive, and quantitative tool for examining the functional structure of microbial communities (19, 26–31) from a variety of environments (32, 33), including soils (27–29, 31, 34–36), aquatic ecosystems (37, 38), extreme environments (26, 39), contaminated habitats (40–47), and bioreactors (48–51).

Although many technical issues regarding microarray technology have been solved, several critical bottlenecks still exist. One of the greatest challenges is that most of the probes on previous versions of GeoChip were derived from genes/sequences in publicly available databases and do not necessarily fully represent the diversity of the microbial communities of interest given the rapid expansion of sequence information in these databases. Consequently, it could be difficult to use these older versions of GeoChip to fully address research questions in a comprehensive manner. Thus, further developments are needed to improve representativeness. In this study, we aimed to develop a more comprehensive and representative generation of FGA, GeoChip 5.0. Previous functional gene families were updated, and more than 1,000 new functional gene families were added. The newly developed GeoChip 5.0 was systematically evaluated in terms of specificity, sensitivity, and quantitative capability. It was then applied to analyze the responses of groundwater microbial communities to high concentrations of U(VI), nitrate, and low pH. Our results demonstrate that the developed GeoChip is highly specific, sensitive, and quantitative for functionally profiling microbial communities.

## RESULTS

### Selection of gene families and categories for array fabrication.

Functional gene families from previous GeoChip versions (410 gene families) were updated and included in GeoChip 5. During this update, some gene families were combined or separated based on newly discovered gene families or increased sequence availability. For example, 12 dioxygenase gene families were combined into three families due to similarities in the sequences of these families; *norB* was split into two gene families to differentiate a new subgroup discovered after the design of GeoChip 4. GeoChip 5.0 also greatly expanded overall gene and sequence coverage by adding more than 1,000 new gene families from broad, functionally divergent taxonomic groups of bacteria, archaea, fungi, algae, protists, and viruses. The rationale for selecting various gene families is detailed in the supplemental material and previous publications (16, 17, 52–54).

Probes for the GeoChip 5.0S and 5.0M cover 1,517 gene families, including those involved in C (118 gene families), N (22 gene families), S (17 gene families), and P (7 gene families) cycling; organic contaminant degradation (157 gene families); stress response (86 gene families); metal homeostasis (105 gene families); microbial defense (87 gene families); plant growth promotion (31 gene families); electron transport (35 gene families); virulence (587 gene families); virus-, fungus-, and protozoan-specific genes (115, 66, and 83 gene families, respectively); and *gyrB* (Table 1). GeoChip 5.0M has substantially more probes than GeoChip 4, 19% to 597% more for most of the functional gene categories (Table 1). However, the number of probes for N cycling and organic contaminant degradation decreased slightly due to a greater coverage by group-specific probes (Table 1). From a taxonomic/phylogenetic perspective, GeoChip 5.0M targets ∼6,500 bacterial strains (1,122 genera), 282 archaeal strains (101 genera), 625 fungi (297 genera), 362 protists (219 genera), 86 other lower eukaryotes (64 genera), 1,364 viral strains (167 genera), and uncultured/unidentified organisms (33 genera) (Table 2; see also Table S2 in the supplemental material). Phylogenetic coverage in GeoChip 5 is 93% to 166% greater than in GeoChip 4. Detailed comparisons of functional gene and phylogenetic coverage in GeoChip 4 and 5 are presented in Tables S1 and S2.

**TABLE 1:** Summary of probes on GeoChip 5.0M by functional gene categories^*c*^

Functional gene category	No. of:						% of probe changes compared to GeoChip 4
	Subcategories	Genes or enzymes	Sequence-specific probes	Group-specific probes	Total probes	Covered CDS	
C cycling	3	118	4,354	19,261	23,615	50,040	+114
N cycling	7	22	2,397	3,600	5,997	11,654	−19
S cycling	5	17	1,969	2,317	4,286	6,823	+38
P cycling	4	7	960	2,300	3,260	6,245	+143
Metal homeostasis	24	105	5,084	37,543	42,627	91,614	+360
Organic contaminant degradation	7	157	2,204	9,241	11,445	27,938	−33
Electron transport	3	35	612	1,348	1,960	3,351	+72.3
Stress response	18	86	2,098	23,634	25,732	79,356	+19
Plant growth promotion	7	31	957	2,263	3,220	5,720	NA^*a*^
Microbial defense	4	87	3,284	19,954	23,238	50,019	+597
Virulence	10	587	1,264	3,596	4,860	10,863	+30
Virus specific	4	115	1,521	1,336	2,857	5,182	+167
Protozoan specific	10	84	845	615	1,460	2,146	NA^*a*^
Fungus specific	9	66	2,559	2,079	4,638	6,987	−7
GyrB	1	1	532	2,234	2,766	9,997	+18

Total	116	1,447	30,640	131,321	161,961	365,651^*b*^	+97

a NA (not applicable) because this is a new category for GeoChip 5.0.

b Total number of covered coding DNA sequences (CDS) does not equal the sum of those from individual categories due to the presence of CDS that were covered in two or more categories.

c Detailed information on individual subcategories of functional genes is presented in Table S1.

**TABLE 2:** Summary of probes in GeoChip 5.0M within broad microbial groups^*e*^

Major microbial group	No. of:							% of probe changes compared to GeoChip 4
	Phyla	Genera	Species	Strains	Genes	Probes	Covered CDS	
Bacteria	33	1,122	2,721	6,465	1,003	141,153	333,675	+93
Archaea	6	101	188	282	269	5,728	38,978	+124
Fungi	7	297	404	625	226	8,856	21,101	+130
Protists	10	219	251	362	201	2,051	5,376	
Other eukaryotes^*a*^	7	64	66	86	62	509	1,170	
Viruses	1	167	311	1,364	116	2,848	6,028	+166
Unclassified^*d*^					125	816	2,561	+116
Total	64	1,970	3,941	9,184	1,447^*b*^	161,961	365,651^*c*^	+97

a Other eukaryotes include Metazoa and Viridiplantae.

b Total number of genes does not equal the sum of those from individual taxonomic groups due to the presence of the genes shared across two or more taxonomic groups.

c Total number of covered CDS does not equal the sum of those from individual taxonomic groups due to the presence of the CDS covered in two or more taxonomic groups.

d The sequences are unclassified due to missing annotations in the data source; most of these are metagenomics sequencing contigs.

e Detailed information on the phylogenetic distribution of functional genes is in Table S2.

10.1128/mSystems.00296-19.7 TABLE S1 Functional gene families covered on the GeoChip 5.0. Download Table S1, PDF file, 0.3 MB. Copyright © 2019 Shi et al. 2019 Shi et al. This content is distributed under the terms of the Creative Commons Attribution 4.0 International license.

10.1128/mSystems.00296-19.8 TABLE S2 Summary of probes on GeoChip 5.0M based on the phylogenetic distribution of the functional genes. Download Table S2, DOCX file, 0.04 MB. Copyright © 2019 Shi et al. 2019 Shi et al. This content is distributed under the terms of the Creative Commons Attribution 4.0 International license.

### GeoChip 5.0 design and overall features.

GeoChip 5.0 was *in situ* synthesized by Agilent’s SurePrint technology. The spots are circular (30-μm diameter). Compared to other array technologies, Agilent arrays have a wider dynamic range, higher sensitivity, and better quantitative capability (55). GeoChip 5.0S contains ∼57,000 probes for ∼151,000 target genes and is focused on the analysis of key ecological and geochemical processes (C, N, S, and P) and other important functional gene groups such as organic contaminant degradation genes, a subset of metal resistance genes that transform the metal (reductases, oxidases, and transferases), and antibiotic resistance genes that alter or degrade the target antibiotic (Table S3).

10.1128/mSystems.00296-19.9 TABLE S3 Overall differences between GeoChip 4 and 5. Download Table S3, DOCX file, 0.01 MB. Copyright © 2019 Shi et al. 2019 Shi et al. This content is distributed under the terms of the Creative Commons Attribution 4.0 International license.

GeoChip 5.0M is a more comprehensive design and contains ∼162,000 probes from ∼365,000 target genes, covers all the functions on the smaller array, and includes a wider range of genes from additional functional categories, such as virulence, plant growth promotion, and microbial defense, across different organismal groups (bacteria, archaea, fungi, algae, protists, and viruses) (Table 1; Table S1). GeoChip 5.0M was designed for a general survey of environmental, ecological, and biogeochemical processes. Detailed differences in the numbers of probes across different gene families between GeoChip 5.0S and 5.0M are listed in Table S3.

Control probes for hybridization, gridding, and data analysis are present in both GeoChip 5.0S and 5.0M (Table S3). GeoChip 5.0M contains 5,282 probes targeting 16S rRNA sequences as positive controls and 3,390 Agilent negative controls. To assist with normalization of signal intensity, GeoChip 5.0M has 3,378 probes targeting six sequenced hyperthermophile genomes and 1,360 common oligonucleotide reference standards (56). GeoChip 5.0S contains the same controls but with fewer probes for each (Table S3).

### Optimization of hybridization conditions.

Agilent arrays typically use 60-mer probes and are hybridized at 65°C with pure genomic DNAs (57). However, GeoChip probes are 50-mers and are used for detecting microbial populations in complex communities, so hybridization conditions need to be optimized. First, temperature is one of the most important variables in determining hybridization specificity and efficiency. In addition, our previous studies indicated that adding formamide to the hybridization buffer is useful for achieving high specificity and low background for environmental DNAs (16, 17, 52, 58, 59). Therefore, hybridization temperatures (60 to 75°C) and formamide concentrations (0 to 25%) were evaluated. Our results indicated that good hybridization can be achieved at 67°C and 10% formamide as judged visually (i.e., 16S and reference standard control probes are visible, and a reasonable number of target probes are positive) (Fig. S1).

10.1128/mSystems.00296-19.2 FIG S1 Effects of DNA concentration on hybridization. Different amounts of grassland soil community DNAs (1 ng to 1,000 ng) were labeled with Cy3 (red spots) and hybridized to GeoChip 5.0S at 67°C, plus 10% formamide. Cy5-labeled common oligonucleotide reference standards (green spots) were added to the hybridization solution as a control. Download FIG S1, TIF file, 0.5 MB. Copyright © 2019 Shi et al. 2019 Shi et al. This content is distributed under the terms of the Creative Commons Attribution 4.0 International license.

**FIG 1:**
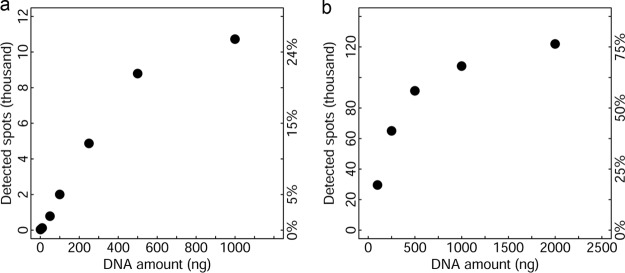
Relationship between detected spots and the concentration of community DNAs used. (a) Hybridization of grassland soil community DNAs with GeoChip 5.0S (see the images in Fig. S2). (b) Hybridization of community DNAs from a wastewater treatment plant with GeoChip 5.0M. Different amounts of unamplified community DNAs were labeled with Cy3 in triplicate. Hybridizations were carried out at 67°C plus 10% formamide for 24 h. Any spots with a signal-to-noise ratio (SNR) of >2 were considered positive.

Template DNA concentration also has significant impacts on hybridization efficiency. Thus, different amounts of microbial community DNAs were hybridized using the optimized hybridization conditions determined above. Although the number of spots detected increased as DNA concentration increased, the percentage of positive spots began to plateau at about 500 ng for GeoChip 5.0S and about 1,000 ng for 5.0M (Fig. 1a and b). However, for both GeoChip 5.0S and 5.0M, 250 ng of DNA resulted in approximately half the number of positive spots as with the recommended DNA amount (Fig. 1a and b), and similar hybridization patterns were present with 250 to 1,000 ng DNA (Fig. S1). As such, smaller amounts of DNA could be used if sufficient DNA is not available to avoid the need for amplification as long as the same amount is used for all samples. Based on the results of these experiments, the recommended hybridization conditions for the Agilent format GeoChip are 1,000 (5.0M) or 500 (5.0S) ng DNA and hybridization at 67°C plus 10% formamide.

10.1128/mSystems.00296-19.3 FIG S2 (A) DCA based on measured environmental variables. (B) DCA based on functional genes detected from these 12 wells. Download FIG S2, TIF file, 0.2 MB. Copyright © 2019 Shi et al. 2019 Shi et al. This content is distributed under the terms of the Creative Commons Attribution 4.0 International license.

### Specificity of designed arrays.

**FIG 2:**
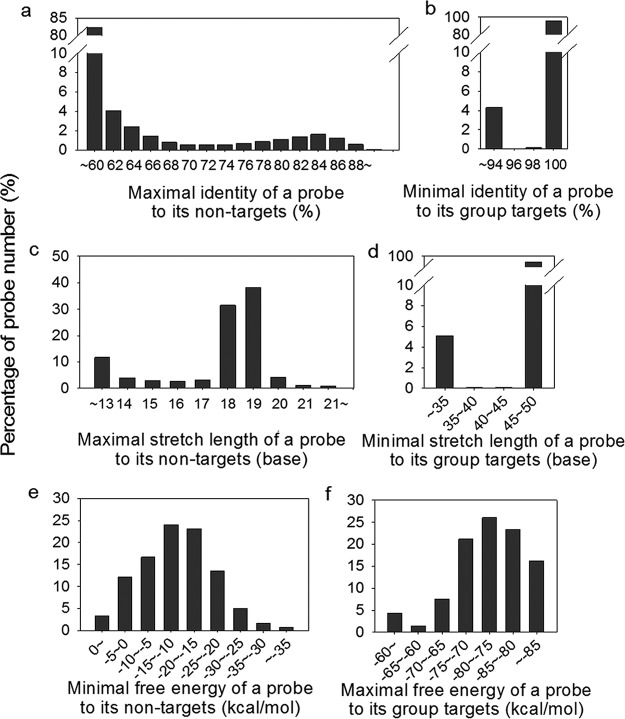
Computational evaluation of the specificity of the designed probes based on sequence identity, length of continuous sequence stretch, and free energy. Three parameters were evaluated by comparing the designed probes to sequences in the databases. (a) Maximal sequence identity (%) of a probe (sequence or group specific) to its closest nontarget sequences. (b) Minimal sequence identity (%) of a group-specific probe to its targeted group sequences. (c) Maximal sequence stretch length (bp) of a probe to its closest nontarget sequences. (d) Minimal sequence stretch length (bp) of a group-specific probe to its targeted group sequences. (e) Minimal free energy (kcal/mol) of a probe to its closest nontarget sequence. (f) Maximal free energy (kcal/mol) of a group-specific probe to its targeted group sequences.

The specificity of the designed probes was determined computationally and experimentally. For sequence-specific probes, the maximum identity, maximum stretch length, and minimal free energy to the closest nontarget sequences were calculated. Most of the sequence- or group-specific probes (82.2%) had <60% maximum sequence identities to nontarget sequences in the NCBI databases (nt and env_nt) (Fig. 2a). Less than 1% of the probes showed 86 to 90% sequence identity with nontarget sequences, and none had >90% sequence identity with nontarget sequences (Fig. 2a). Most of the probes (93.8%) had maximal continuous sequence stretches of <19 bp to nontarget sequences (Fig. 2c). In addition, 99.3% of probes had minimal free energy of >−30 kcal/mol (Fig. 2e). As previously demonstrated experimentally, the designed probes would be highly specific if they have <90 to 92% sequence identity, <20-bp continuous sequence stretch, and >−35 kcal/mol free energy to nontarget sequences (60).

There are potential mismatches between group-specific probes and corresponding target sequences that could affect hybridization efficiency and hence subsequent sensitivity and quantification. Thus, group-specific probes were further required to have minimal sequence identity of >94%, minimal continuous stretch length of >35 bp, and maximal free energy of <−60 kcal/mol to the corresponding targeted sequences (16, 17). More than 94% of the designed group-specific probes had a sequence identity of ≥98%, continuous sequence stretches of ≥45 bp, and free energy of ≤−70 kcal/mol to corresponding target sequences (Fig. 2b, d, and f).

**FIG 3:**
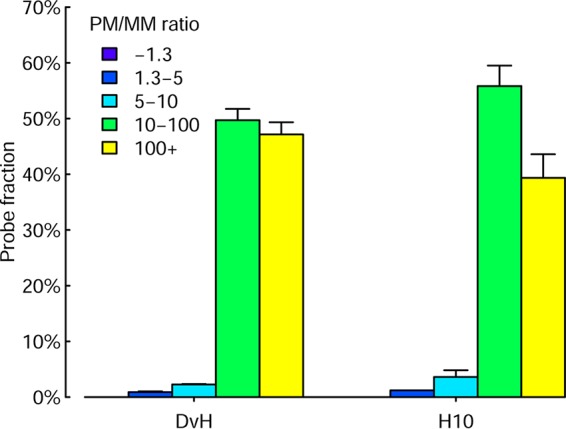
Experimental evaluation on the specificity of designed arrays with perfect match (PM)/mismatch (MM) probes. One hundred nanograms of genomic DNAs was labeled with Cy3 and hybridized with a modified GeoChip 5.0S in triplicate. For each PM or MM pair, the net signal intensity was obtained by subtracting the signal intensity from the Agilent negative controls within a subarray from the raw signal intensity. The ratio of PM to MM probe pairs was estimated. DvH, *D. vulgaris* Hildenborough.

Hybridization specificity was further evaluated using perfect match (PM)/mismatch (MM) probes (61). A set of 938 PM probes and a corresponding set of 938 MM probes for both Desulfovibrio vulgaris Hildenborough (Gram negative, GC content ∼63%), and H10 (Gram-positive, GC content ∼37%) were added to the GeoChip 5.0S. MM probes were generated by dividing a PM probe into 5 equal segments and randomly introducing one mismatch into each segment (61), for a total of 5 mismatches (10% difference). Hybridization signals from the MM probes should represent nonspecific cross-hybridization (i.e., background noise) to the corresponding PM probes (61). Previous studies suggested that any probes with a signal intensity ratio of PM/MM >1.3 would be considered a positive hybridization signal (19). To test specificity, equal amounts (100 ng) of pure culture DNAs were mixed, labeled, and hybridized in triplicate. Under the hybridization conditions used (67°C and 10% formamide), most probes (96.8% for *D. vulgaris* Hildenborough and 95.1% for H10) had PM/MM ratios of >10 (Fig. 3). None of the PM/MM probes had a ratio of <1.3, and a very small portion (0.8% for *D. vulgaris* Hildenborough and 1.2% for H10) had ratios of <5.

### Sensitivity of the designed arrays.

The sensitivity of the arrays was evaluated with genomic DNAs from *D. vulgaris* Hildenborough and H10. Pure culture DNAs (0.05, 0.1, 0.5, 1, 5, 10, 50, and 100 ng) were mixed with grassland soil DNAs so that the total amount of DNAs used for hybridization was 1,000 ng. The mixed DNAs were hybridized in triplicate with the GeoChip 5.0S containing the PM/MM probes.

**FIG 4:**
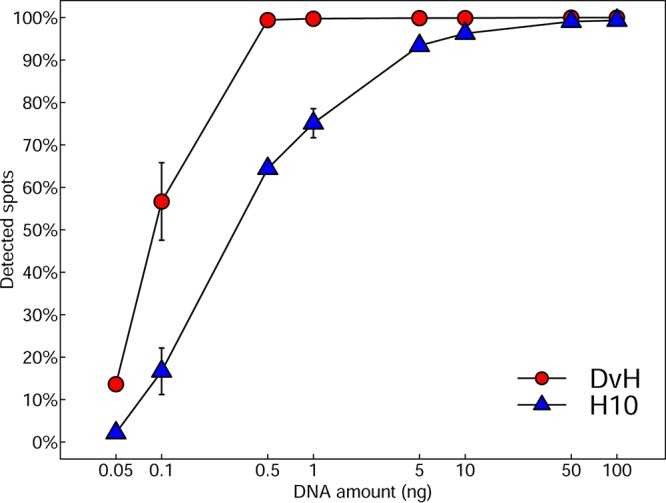
Sensitivity evaluation of the designed arrays with pure genomic DNAs. Genomic DNAs from *D. vulgaris* Hildenborough and H10 (0.05 ng to 100 ng) were mixed with grassland soil community DNAs as a background to equal 1,000 ng. The mixed DNAs were labeled with Cy3 and hybridized in triplicate to a GeoChip 5.0S containing 938 probes each from *D. vulgaris* Hildenborough (DvH) and H10.

As shown in Fig. 4, >90% (∼932) of the pure culture probes were detected at a genomic DNA concentration of 0.5 ng (0.05% of the total community DNA) for *D. vulgaris* Hildenborough and 5 ng (0.5% of the total) for H10. Over 50% of the probes showed positive hybridization at a genomic DNA concentration of 0.1 ng (0.01% of the total) for *D. vulgaris* Hildenborough and 0.5 ng (0.05% of the total) for H10. A small percentage of probes (13.6% *D. vulgaris* Hildenborough, 2.1% H10) were detected even at 0.05 ng DNA (0.005% of the total). The low-GC-content organism (H10) had a hybridization sensitivity roughly 10 times lower than the high-GC organism (*D. vulgaris* Hildenborough), likely due to the weaker bond between the A and T bases compared to that between G and C.

### Quantitation of the designed arrays.

**FIG 5:**
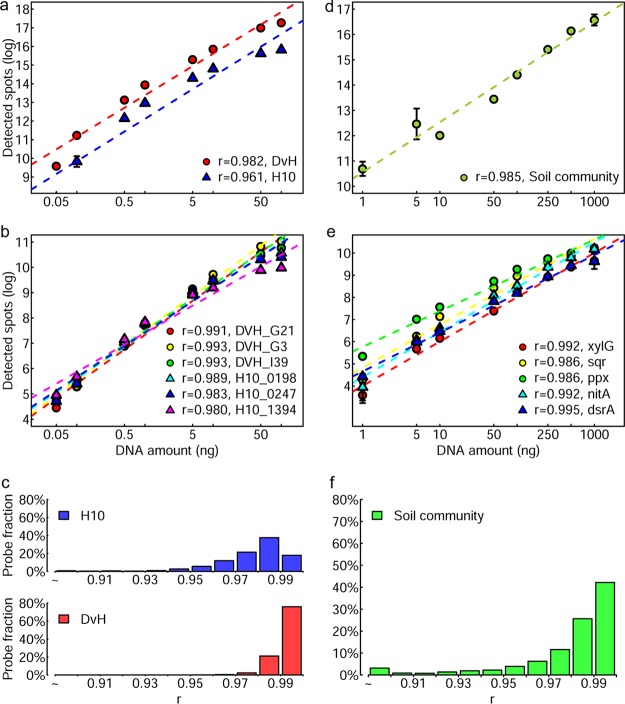
Quantitative evaluation of the designed arrays with pure culture and soil community DNAs. (a) Relationship of total signal intensity of all detected spots to the amount of pure culture DNAs used. (b) Relationship of the signal intensity of selected representative probes to the amount of pure culture DNAs used. (c) Distribution of Pearson correlation coefficients (*r*) based on individual spots for pure culture DNAs. (d) Relationship of total signal intensity of all detected spots to the amount of soil community DNAs used. (e) Relationship of signal intensity of selected representative probes to the amount of soil community DNAs used. (f) Distribution of Pearson correlation coefficients (*r*) based on individual spots for soil community DNAs.

The quantitative capability of the arrays was first evaluated with *D. vulgaris* Hildenborough and H10 in the presence of soil DNAs as background (Fig. 4). Both signal intensity and DNA concentration were log transformed. The total signal intensity for all genes was highly correlated with the total amount of DNAs used for both *D. vulgaris* Hildenborough (Pearson correlation coefficient, *r *=* *0.982) and H10 (*r *=* *0.961) (Fig. 5a). Also, all detected genes showed significant correlations (*r *=* *0.824 to 0.999; *P < *0.05) with DNA concentration over more than 3 orders of magnitude. Extremely strong correlations between signal intensity and DNA concentration were observed for some representative genes (Fig. 5b). In addition, 937 *D. vulgaris* Hildenborough and 877 H10 genes were detected in at least 6 of the concentrations tested and about 99% had *r *>* *0.9 (Fig. 5c).

The quantitative nature of the arrays was also assessed with soil DNAs. Soil DNAs from a grassland (1, 5, 10, 50, 100, 250, 500, and 1,000 ng) were mixed with salmon sperm DNAs as a background to equal 1,000 ng DNA. The mixed DNAs were hybridized with GeoChip 5.0S. As with pure culture DNAs, strong correlations were observed between the total signal intensity of all detected probes and DNA concentrations used (Fig. 5d). A total of 2,496 genes were detected in the two highest concentrations and across at least 4 of the other concentrations, all of which showed significant correlations (*P < *0.05) between the signal intensity and DNA concentrations across at least 3 orders of magnitude. Some genes had correlations of >0.99 (Fig. 5e). About 97% of the genes had *r *of >0.9 (Fig. 5f).

### Application of GeoChip 5.0 to analysis of contaminated groundwater microbial communities.

To demonstrate the usefulness of the developed GeoChip, we examined the impacts of heavy metal contamination on groundwater microbial communities at the Oak Ridge Integrated Field Research Center (OR-FRC). Twelve wells, representing a range of contamination levels [no (L0), low (L1), intermediate (L2), and high (L3) contamination] were selected. A number of physical, chemical, and biological variables were measured for each sample, including heavy metals, pH, nitrate, and sulfide (Table S4) (47, 62). Detrended correspondence analysis (DCA) of the environmental variables showed that, overall, each group of wells (i.e., L0, L1, etc.) was distinctly different from the other groups, but the individual samples within a group were highly similar (Fig. S2a), indicating that the geochemical environments are quite different among these wells.

10.1128/mSystems.00296-19.10 TABLE S4 Environmental variable measurements from groundwater samples used in the application study. A total of 12 samples were collected from wells representing 4 levels of contamination (L0, L1, L2, and L3). Variables were divided into 5 categories: general environmental parameters (Env. Parameters), gas thermal conductivity detector (TCD), dissolved carbon (C), anion, and metal ion. Download Table S4, DOCX file, 0.02 MB. Copyright © 2019 Shi et al. 2019 Shi et al. This content is distributed under the terms of the Creative Commons Attribution 4.0 International license.

A total of 20,295 genes were detected across all samples, varying significantly across samples. As expected, both functional gene richness and Shannon-Weaver diversity decreased significantly as contamination increased, but there was no influence on evenness (data not shown). Microbial community functional structure was also quite different among these sample groups as shown in the DCA ordination plots (Fig. S2b) with more obvious separation among the groups and tighter clustering within groups than with the environmental variables (Fig. S2b).

A total of 114 gene families involved in metal homeostasis were detected across all samples. Significant (*P < *0.05) differences in the relative abundances of many gene families (32% to 55%) were observed among the contaminated group samples (L1 to L3) and the control (L0) (Fig. S3). Compared with L0, the relative abundances of 37 gene families were significantly different in L1, with 18 (e.g., *arxA* and *arsAF* for As homeostasis) having a higher relative abundance in L1; 47 gene families in L2 were significantly different, 25 of which (e.g., *merH* and *merG* for Hg homeostasis) were higher; and 63 gene families were significantly different in L3, with 31 (e.g., *chrA* and *chrR* for Cr homeostasis) that were higher. Significantly higher relative abundances of gene families involved in metal resistance (e.g., *chrA* and *chrR* for Cr; *corC* for Co; *metC, merB*, *merG*, and *merH* for Hg; *zitB* for Zn; and *silA* for Ag) were observed in L2 and L3 compared to L0, but not L1. The above results suggest that the composition of functional genes in the contaminated samples, especially L2 and L3, had altered compared to L0, with an enhanced capability for resistance to relevant metal contaminants (e.g., Cr, Co, Ni, Hg, Zn, and Ag).

10.1128/mSystems.00296-19.4 FIG S3 Relative difference of metal homeostasis gene abundance between L0 and L1, L2, and L3. Dots represent the relative difference of each gene between samples of L0 versus L1 (blue), L0 versus L2 (orange), and L0 versus L3 (red), respectively. Filled dots indicate a statistically significant difference. Relative difference is calculated as the difference of the mean abundance of two groups divided by the higher mean abundance. Level of significance is calculated based on U test (nonparametric, two-tailed *P* < 0.1, i.e., one-tailed *P* < 0.05). Download FIG S3, TIF file, 0.3 MB. Copyright © 2019 Shi et al. 2019 Shi et al. This content is distributed under the terms of the Creative Commons Attribution 4.0 International license.

**FIG 6:**
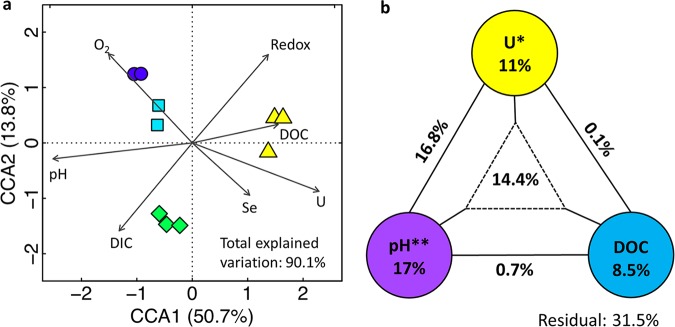
Associating variations in microbial functional gene structure with environmental variables. (a) CCA based on selected environmental variables. A total of 7 environmental factors (U, pH, redox, Se, O_2_, DIC, and DOC) were selected from 41 measured variables. The top two axes (CCA1 and CCA2) were included and accounted for 50.7% and 13.8% microbial functional gene structure variation, respectively. (b) Partial CCA-based VPA assigning variance to U, pH, and DOC. The value inside each colored circle indicates the fraction of variance assigned to that variable alone. Asterisks show level of significance of test in partial CCA: *, *P* < 0.05; **, *P* < 0.01. The value by the solid black line indicates the variance assigned to the interactive effect of the two connected variables. The value inside the dashed triangle indicates the variance assigned to the interactive effect of all three variables.

Canonical correspondence analysis (CCA) was also performed to further understand which environmental variables controlled the groundwater microbial community structure at this site. Among the 41 environmental variables, many were highly correlated with each other (Fig. S4a) and 7 major clusters were identified based on hierarchical clustering analysis (Fig. S4b). We selected one variable from each cluster (U, pH, redox, Se, O_2_, dissolved inorganic C [DIC], dissolved organic C [DOC] [boxed in Fig. S4b]) to represent the variables in that cluster for subsequent CCA. The CCA results showed that differences in the functional gene composition of the groundwater microbial communities were significantly (*P < *0.001) correlated with changes in the selected variables (Fig. 6a). The forward selection procedure identified pH, U, and DOC as variables constraining the most variation; thus, they were further used in partial CCA and variation partitioning analysis (VPA). Results from these assays showed that all three variables combined could constrain 90.1% of the total variation in the microbial community and that pH and U alone were significantly correlated with the observed variations and explained 17% and 11% of the total variation, respectively (Fig. 6b). A relatively large fraction of the variation was also assigned to the interactive effects of pH and U (16.8%) and of pH, U, and DOC (14.4%) (Fig. 6b).

10.1128/mSystems.00296-19.5 FIG S4 Correlation analysis and hierarchical clustering analysis based on measured environmental variables. (a) Heat map of correlation (Pearson correlation) matrix among all environmental factors. The original values of conductivity, Cl, NO_3_, SO_4_, Ag, Al, As, Ba, Be, Bi, Ca, Cd, Co, Cr, Cs, Cu, Fe, Ga, K, Li, Mg, Mn, Na, Ni, Pb, Se, Sr, U, and Zn were log transformed due to the nature of the measurements. (b) Each cluster is labeled with a different color, and representative variables selected from each cluster are boxed with dashed lines. Download FIG S4, TIF file, 0.4 MB. Copyright © 2019 Shi et al. 2019 Shi et al. This content is distributed under the terms of the Creative Commons Attribution 4.0 International license.

### Comparison of GeoChip to shotgun metagenomic sequencing.

Shotgun metagenomic sequencing is frequently used to assess the functional diversity and potential of microbial communities. To compare the performance of these two methods, the same 9 wells examined above were sequenced using shotgun metagenomic sequencing. GeoChip detected a much higher average functional diversity than did shotgun sequencing for the genes of interest (GeoChip, 58,929 ± 10,400; shotgun sequencing, 5,725 ± 496) (Table 3). In addition, when comparing communities from L0, GeoChip detected a higher number of significantly different genes in L1 (GeoChip, 1,987; shotgun, 782) and L2 (1,501; 221), while similar results were obtained for L3 (832; 971).

**TABLE 3:** Comparison of GeoChip and shotgun metagenomics sequencing

Contamination level and sequencing method	Functional gene richness (no. of genes)^*a*^	No. of significantly different genes^*b*^
L0		
Shotgun	6,166 ± 415	
GeoChip	63,739 ± 3,663	
L1		
Shotgun	5,462 ± 396	782
GeoChip	66,225 ± 12,710	1,987
L2		
Shotgun	6,040 ± 180	221
GeoChip	53,999 ± 7,848	1,501
L3		
Shotgun	5,231 ± 285	971
GeoChip	53,357 ± 11,180	832

a Standard deviation of triplicate samples.

b Compared to gene abundance in L0, *t* test.

## DISCUSSION

Although development and application of high-throughput metagenomics technologies have revolutionized the capability of microbiologists to analyze microbial communities in the environment, experimental and computational challenges still exist (24). Thus, in this study, we have developed a new generation of FGA (GeoChip 5.0) which contains 161,961 probes covering functional groups involved in microbial C, N, S, and P cycling, organic contaminant degradation, stress response, metal homeostasis, microbial defense, plant growth promotion, electron transport, virulence, and virus-, fungus-, and protozoan-specific genes and *gyrB*. To the best of our knowledge, this is the most comprehensive FGA currently available for studying microbial communities important to biogeochemistry, ecology, and environmental sciences.

Compared with previous generations, GeoChip 5.0 has several improved features. First, new functional categories (e.g., microbial defense, plant growth promotion, and protozoa) and subcategories (e.g., antimicrobial biosynthesis and environmental toxins) were added. Second, gene coverage of functional gene families and targeted genes more than tripled. Last, GeoChip 5.0 is synthesized using a different chemistry. Agilent’s novel inkjet printing technology increases the sequence fidelity of probes compared to that achieved by conventional printing methods, and the hydrophobic array substrate reduces background signal from nonspecific binding to the array surface (63). These features make GeoChip 5.0 a more comprehensive tool for analyzing microbial communities and linking community structure with environmental factors and ecosystem functioning.

Specificity is critical for microbial detection, particularly for analyzing complex environmental samples such as soils because there are numerous homologous sequences for each gene present. Multiple criteria were used to achieve appropriate specificity. First, seed sequences for a given gene were carefully selected by manual examination to confirm that the identity of these sequences was correct and to exclude irrelevant sequences. Second, experimentally determined criteria based on sequence identity, continuous stretch length, and free energy were simultaneously applied for selecting both sequence- and group-specific probes (60, 64, 65). Last, the specificity of the selected probes was verified against NCBI databases. The above quality control protocols resulted in a highly specific final probe set as demonstrated by computational evaluation showing that most (95%) of the designed probes were far from the criterion thresholds, consistent with previous GeoChip versions (16, 17, 52, 64). Experimental evaluation using PM/MM probes showed considerable differences of signal intensity between PM and MM probes for both high- and low-GC DNAs. Collectively, these results suggest that this probe design strategy is extremely robust and capable of consistently producing highly specific probes regardless of the microarray platform (16, 17, 52, 64).

Reproducibility is another essential attribute of microarrays and other high-throughput technology. Several features of the GeoChip 5 reduce variation in signal, thus improving reproducibility. The high specificity and sensitivity of the GeoChip 5.0 reduce variation from false-positive or -negative signals, and the use of CORS probes reduces the variation from hybridization (56). The close clustering of replicate samples in the CCA and DCA plots (Fig. 6 and Fig. S2b) demonstrates a high reproducibility among samples. Further, the reproducibility of the GeoChip array has been systematically evaluated and has been found to be highly reproducible (J. D. Van Nostrand, J. Shi, H. Yin, D. Ning, L. Wu, and J. Zhou, unpublished data). A 90 to 95% overlap in detected probe overlap was observed among technical replicates in that study.

Array sensitivity is important for detecting lower-abundance community members. This GeoChip version appears to be more sensitive than previous versions using other formats (17, 58, 59, 66, 67). Our studies showed a detection limit as low as 0.005% of DNA from a complex soil community, indicating the GeoChip 5 can detect low-abundance populations. Previous versions were able to detect 5% of the microbial population (59). As little as 0.2 μg community genomic DNA is enough for hybridization without amplification. Shotgun sequencing is less sensitive than GeoChip as demonstrated by the lower functional diversity detected by shotgun sequencing. This lack of sensitivity in shotgun sequencing has been observed previously (68). If very little DNA is available, whole-community-genome amplification (67) can be used. Although this likely introduces additional variation, the experimental results are still meaningful as demonstrated by application of the GeoChip 5.0 to analyze contaminated groundwater microbial communities having low biomass in this study.

Effective and meaningful ecological comparisons across different ecosystems require an accurate quantitation of taxon and gene abundances. This is particularly true for ecosystem modeling. Previous studies of conventional PCR amplification in amplicon-based target sequencing demonstrated that target gene sequencing has little to no quantitative ability in complex communities (69–71) as is the consensus (72, 73). It is generally believed that shotgun sequencing should be quantitative since conventional PCR is not involved (71, 74). However, due to the high inherent variation among experimental protocols and the uncertainty in selecting bioinformatics tools for analysis (74–76), it may be impossible to obtain absolute abundance estimations based on shotgun sequencing data alone (74). While sequencing does not provide reliable quantitative data, qPCR can be used in conjunction with sequencing to provide abundance data. However, qPCR and other gene amplification assays (e.g., functional gene amplicon sequencing) require the use of conserved PCR primers. Designing primers for many functional genes can be difficult due to lack of available sequences in public databases or difficulty in finding conserved (present in all gene family members) or specific (present in only that gene family) regions of the gene sequence. So, gene-specific amplification can be performed on only some functional genes. In contrast, GeoChip probes were designed to be specific to a single or similar group of sequences, so probes can be designed for any functional gene present in the database. Theoretically, the signal intensity from array hybridization reflects the absolute abundance of DNAs used for hybridization (24). Highly quantitative results were obtained in the current study with both complex soil DNAs (*r* = 0.985) and pure culture DNAs (*r* = 0.995). Similar quantitative abilities have been observed with other Agilent-based arrays (55, 77). A comparison of GeoChip and qPCR results on the same samples demonstrated a high correlation between gene copy number and signal intensity for GeoChip 2 and 3 (*r* = 0.530 and 0.724, respectively) (27), consistent with previous experimental evaluations with both DNAs and RNAs (16, 59, 66, 67, 78).

The GeoChip 5.0 was applied to investigate microbial communities in contaminated groundwater. The observed shift in functional gene composition as contamination increased was expected as the contaminants at this site (e.g., NO_3_^−^, metals, and pH) may stimulate specific functional processes (e.g., denitrification, sulfate reduction, and metal homeostasis) that utilize or are induced by the contaminants. Changes in the relative abundance of metal homeostasis genes were observed in this study, and similar results have been observed in other studies of ecosystems contaminated by heavy metals (79–82). Contaminants can also inhibit the growth of the microbial species, which could in turn affect general functional processes such as C cycling and drive further functional gene changes by decreasing the abundances of related genes. Decreases in functional gene diversity were another major impact brought about by the presence of contaminants. Only a few microbial species with strong tolerance or degradation ability are likely to be enhanced in this environment, while most species are likely to be reduced due to their higher sensitivity to the toxicity of the contaminants (81, 83, 84). A recent study of this same site reported that emulsified vegetable oil addition led to the dominance of several sulfate-reducing bacterial species that may be responsible for U(IV) reduction and suggested that U(IV) was one of the key factors controlling the local microbial diversity (85). These findings were further supported by results from this study demonstrating significant correlations between several microbial functional genes and environmental factors, particularly U(IV) concentration. Similar conclusions have been drawn from other studies at this contaminated site using GeoChip versions 2 (52, 86), 3 (87), 4 (D. J. Curtis, P. Zhang, J. D. Van Nostrand, and J. Zhou, unpublished data), and 5 (47), indicating the data generated by the GeoChip are consistent from version to version. In addition, a study examining a contaminated and uncontaminated well at the OR-FRC using shotgun metagenomics found that communities from highly contaminated wells were less diverse and had a higher abundance of stress and metal resistance genes compared to the pristine well (88), as has been observed in the GeoChip studies referenced above. In the current study, the GeoChip 5 was able to detect a much higher functional diversity in these communities than shotgun sequencing and was able to detect more or similar numbers of genes with significant differences. These results showcase the effectiveness of the GeoChip 5.0 in characterizing complex environmental microbial communities from a functional gene perspective.

In summary, the developed GeoChip 5.0 contains ∼160,000 probes, covering ∼370,000 sequences in ∼1,500 gene families. It is the most comprehensive FGA available to date for dissecting the functional structure of complex microbial communities. Computational and experimental evaluations demonstrated that GeoChip 5.0 is highly specific, sensitive, and quantitative for characterizing microbial community functional composition and structure. The GeoChip allows for rapid, high-throughput, and cost-effective analysis of microbial communities. As previously discussed (24), open-format sequencing-based and closed-format array-based technologies have different advantages and disadvantages in terms of specificity, sensitivity, quantitation, resolution, reproducibility, and novel discovery. Thus, they should ideally be used in a complementary fashion to address complex ecological questions within the context of ecological, environmental, and medical applications (24). The FGA developed here is an important part of the integrated omics toolbox for microbial community analysis.

## MATERIALS AND METHODS

### Sequence retrieval and probe design.

Sequence retrieval and probe design for the GeoChip 5.0 were performed using the GeoChip design pipeline as described previously (16, 17). To maintain consistency between GeoChip versions and minimize the number of probes that needed to be designed, legacy probes from previous versions of GeoChip that were still valid were included on GeoChip 5 (see Fig. S5 in the supplemental material). Probe design was performed using a new version of the CommOligo software (65).

10.1128/mSystems.00296-19.6 FIG S5 The workflow of GeoChip 5.0 development includes four major steps: (a) candidate sequence retrieval, (b) candidate sequence confirmation, (c) target screening and legacy probe validation, and (d) probe design and selection. (a) A keyword query was manually crafted for every functional gene family and submitted as a query to the NCBI databases for retrieval of candidate sequences. (b) Seeds for each gene were selected from the candidate sequences to build a hidden Markov model (HMM), and then the homology of candidate sequences was confirmed by searching them against the HMM. The confirmed candidate sequences were selected as targets to be covered by this version of GeoChip. (c) The targets were searched against the legacy probes to exclude targets covered by legacy probes (target screening) and those legacy probes that were no longer specific (legacy probe validation). Targets covered by invalid legacy probes were recycled in panel d. (d) Targets were pooled as input for probe design, and highly specific probes were selected by searching against NCBI databases. Download FIG S5, TIF file, 0.4 MB. Copyright © 2019 Shi et al. 2019 Shi et al. This content is distributed under the terms of the Creative Commons Attribution 4.0 International license.

### Microarray analysis.

Two versions of the GeoChip 5.0 array were developed. The smaller version (GeoChip 5.0S) has ∼60,000 probes per array (see Table S1 for details). The larger format (GeoChip 5.0M) has ∼180,000 probes per array (Table S1). All GeoChip 5.0 microarrays were manufactured by Agilent (Santa Clara, CA, USA) using either the 8 by 60,000 (8 arrays per slide) or the 4 by 180,000 (4 arrays per slide) format.

Genomic DNA from Desulfovibrio vulgaris Hildenborough and Clostridium cellulolyticum H10 (H10) was extracted using a GenElute bacterial genomic DNA kit (Sigma-Aldrich, St. Louis, MO, USA) following the manufacturer’s instructions. Soil (5 g) and groundwater (4 to 6 liters) were extracted using freeze-grinding mechanical lysis (89). Wastewater samples were extracted using a PowerSoil DNA isolation kit (Qiagen, Germantown, MD, USA).

Since very small amounts of community DNAs were obtained from groundwater, whole-community-genome amplification was required (67). DNA was labeled with Cy3 using random priming with Klenow fragment, cleaned using a QIAquick purification kit (Qiagen) per the manufacturer’s instructions, and then dried. Labeled DNA suspended in hybridization solution containing 10% formamide was pipetted into the center of a gasket slide well (Agilent), covered with an array slide, sealed using a SureHyb chamber, placed into the hybridization oven, and hybridized at 67°C for 24 h. After hybridization, slides were rinsed and imaged with a NimbleGen MS200 microarray scanner (Roche NimbleGen, Madison, WI, USA).

All statistical analyses were performed in R (version 3.4.4, 2018-03-15) using packages stats, ape, and vegan.

A more detailed description of methods used is in Text S1 in the supplemental material.

10.1128/mSystems.00296-19.1 TEXT S1 Detailed materials and methods and a detailed description of all genes covered on the GeoChip 5. Download Text S1, PDF file, 0.5 MB. Copyright © 2019 Shi et al. 2019 Shi et al. This content is distributed under the terms of the Creative Commons Attribution 4.0 International license.
