# Supplementary material for: Functional Gene Array-Based Ultrasensitive and Quantitative Detection of Microbial Populations in Complex Communities
Source: mSystems. 2019 Jun 18;4(4):e00296-19. doi: 10.1128/mSystems.00296-19 (PMC6581690; doi:10.1128/mSystems.00296-19)
Supplement: TABLE S1 [file mSystems.00296-19-st001.pdf]

Table S1. Functional gene families covered on the GeoChip 5.0.

Category	Subcategory	Subcategory 2	Gene	Encoded Enzyme	No. of sequence-specific probes[a]	No. of group-specific probes[a]	No. of total probes on 5M	No. of covered CDS[a]	No. of total probes on 5S
**Categories for Geochemical Cycling**									
**Carbon Cycling**	**Carbon Degradation**	**Agar**	beta_agarase	Agarase	42	79		217	121
				Agar Total	*42*	*79*	*0*	*217*	*121*
		**Alginate**	alginase	Alginase	45	200		512	245
				Alginate Total	*45*	*200*	*0*	*512*	*245*
		**Cellulose**	Endoglucanase	Endoglucanase	198	248	446	740	343
				Cellulose Total	*198*	*248*	*446*	*740*	*343*
		**Chitin**	Acetylglucosaminidase	Acetylglucosaminidase	119	976	1095	3088	1116
			Chitinase	Chitinase	649	1231	1880	3285	1411
			endochitinase	Endochitinase	213	334		1104	547
			exochitinase	Exochitinase	14	55		259	69
				Chitin Total	*995*	*2596*	*2975*	*7736*	*3143*
		**Glyoxylate cycle**	AceA	Isocitrate lyase	69	373	442	887	0
			AceB	Malate synthase A	90	610	700	1457	0
				Glyoxylate cycle Total	*159*	*983*	*1142*	*2344*	*0*
		**Hemicellulose**	Ara	Arabinofuranosidase	172	543	715	1367	827
			Mannanase	Mannanase	159	238	397	639	478
			Xylanase	Xylanase	150	653	803	1739	858
				Hemicellulose Total	*481*	*1434*	*1915*	*3745*	*2163*
		**Heparin**	heparinase	Heparinase	*7*	*53*		*166*	60
				Heparin Total	*7*	*53*	*0*	*166*	*60*
		**Hyaluronic acid**	hyaluronidase	Hyaluronidase	*8*	*80*		*383*	88
				Hyaluronidase Total	*8*	*80*	*0*	*383*	*88*
		**Lignin**	Glx	Glyoxal oxidase	103	40	143	178	125
			Mnp	Manganese peroxidase	50	13	63	74	66
			Phenol_oxidase	Laccase or phenol oxidase	188	372	560	1009	677
				Lignin Total	*341*	*425*	*766*	*1261*	*868*
		**Pectin**	Pectinase (pectate_lyase)	Pectate lyase	53	252	305	827	321
			Pme	Pectin methylesterase	25	240	265	593	313
			RgaE	Rhamnogalacturonan acetylesterase	50	272	322	685	361
			Rgh	Rhamnogalacturonan hydrolase	32	45	77	121	104
			Rgl	Rhamnogalacturonan lyase	104	185	289	542	341
				Pectin Total	*264*	*994*	*1258*	*2768*	*1440*
		**Starch**	AmyA	Alpha-amylase	748	5010	5758	15122	6508
			Amyx	Pullulanase	2	13	15	36	18
			Apu	Amylopullulanase	2	6	8	14	14
			Cda	Cyclomaltodextrinase	110	518	628	1503	488
			Isopullulanase	Isopullulanase	6	1	7	8	0
			NplT	Neopullulanase	44	127	171	342	0
			Pula	Pullulanase	58	385	443	1084	413
				Starch Total	*970*	*6060*	*7030*	*18109*	*7441*
		**Terpenes**	Cdh	Carveol dehydrogenase	5	59	64	156	75
			Limeh	Limonene-1,2-epoxide hydrolase	16	69	85	153	92
			Lmo	Limonene 1,2-monooxygenase	5	6	11	17	8
				Terpenes Total	*26*	*134*	*160*	*326*	*175*
		**Vanillin/Lignin**	Vana	Vanillate O-demethylase oxygenase subunit	30	215	245	501	219
			Vdh	Vanillin: NAD+ oxidoreductase	19	48	67	121	79
				Vanillin/Lignin Total	*49*	*263*	*312*	*622*	*298*
		Carbon Degradation Total			***3585***	***13549***	***16004***	***38929***	***16385***
	**Carbon Fixation**	**3-hydroxypropionate bicycle**	accD	Acetyl-CoA carboxylase, carboxyl transferase subunit beta	0	4	4	8	5
			Mch	MaoC domain-containing protein dehydratase	1	0	1	1	1
			Mcl	HpcH/HpaI aldolase	0	2	2	4	3
			MCM	Methylmalonyl-CoA mutase, large subunit	0	8	8	16	9
			Mcr	Short-chain dehydrogenase/reductase SDR	0	2	2	4	3
			Mct	L-carnitine dehydratase/bile acid-inducible protein F	0	2	2	4	2
			MMCE	Glyoxalase/bleomycin resistance protein/dioxygenase	0	2	2	4	3
			Pcc_3HP	Propionyl-CoA carboxylase	0	3	3	6	0
			Pcs	AMP-dependent synthetase/ligase	0	2	2	4	3
			SdhA	Succinate dehydrogenase flavoprotein subunit	0	2	2	4	3
				3-hydroxypropionate bicycle Total	*1*	*27*	*28*	*55*	*32*
		**3-hydroxypropionate/4-hydroxybutyrate cycle**	3HP_CoAs	3-hydroxypropionyl-CoA synthetase	1	2	3	5	4
			4HB_CoA_synthetase	4-hydroxybutyryl-CoA synthetase	1	0	1	1	1
			4hbcd	4-hydroxybutyryl-CoA dehydratase	3	5	8	13	11
			AcaB_arch	Putative acyl-CoA acyltransferase	0	6	6	12	8
			Acc_arch	Acetyl/propionyl-CoA carboxylase, alpha subunit	0	3	3	17	3
			C_CoA_hydratase	Crotonyl-CoA hydratase	0	4	4	8	4
			Fumarase_3HP4HB	Fumarate lyase	0	2	2	4	3
			MCM_3HP4HB	Methylmalonyl-CoA mutase	0	6	6	12	8
			MMCE_3HP4HB	Methylmalonyl-CoA epimerase	0	6	6	12	7
			Pcc_3HP4HB	Propionyl-CoA carboxylase beta chain	1	2	3	4	3
			Suc_CoA_red	Succinate semialdehyde reductase (NADPH)	3	0	3	3	3
			Suc_sa_red	Succinate semialdehyde reductase (NADPH)	2	1	3	4	3
				3-hydroxypropionate/4-hydroxybutyrate cycle Total	*11*	*37*	*48*	*95*	*58*
		**Bacterial Microcompartments**	CcmL	Ethanolamine utilization protein EutN/carboxysome structural protein Ccml	17	231	248	533	279
			CcmM	Carbon dioxide concentrating mechanism protein ccmM	2	11	13	26	17
			CcmN	Carboxysome protein CcmN	1	7	8	15	9
			CsoS1_CcmK	Microcompartments protein	26	507	533	1137	604
			CsoS2	Carboxysome shell protein CsoS2	4	33	37	73	44
			CsoSCA	Carboxysome shell polypeptide	5	33	38	72	46
			IcfA	Carbonic anhydrase	0	7	7	14	12
				Bacterial Microcompartments Total	*55*	*829*	*884*	*1870*	*1011*
		**Calvin cycle**	FBP_aldolase	Fructose-bisphosphate aldolase	24	178	202	424	223
			FBPase	Inositol phosphatase/fructose-16-bisphosphatase	23	371	394	994	437
			GAPDH_Calvin	Glyceraldehyde-3-phosphate dehydrogenase, type I	41	444	485	1014	541
			Pgk	Phosphoglycerate kinase	18	231	249	552	277
			PRI	Ribose 5-phosphate isomerase	16	268	284	632	310
			PRK	Phosphoribulokinase	9	158	167	354	191
			Rubisco	Ribulose-bisphosphate carboxylase	208	262	470	807	534
			TIM	Triosephosphate isomerase	22	283	305	713	340
			TktA	Transketolase	42	525	567	1186	632
				Calvin cycle Total	*403*	*2720*	*3123*	*6676*	*3485*
		**Dicarboxylate/4-hydroxybutyrate cycle**	4hbcd_dic4hb	4-Hydroxybutyryl-CoA dehydratase	0	11	11	22	11
			AACT_DiC4HB	Acetoacetyl-CoA beta-ketothiolase	9	17	26	43	28
			AdhC	Alcohol dehydrogenase	2	3	5	8	6
			C_CoA_hydratase_DiC4HB	Crotonyl-CoA hydratase	0	13	13	30	13
			FrdA	Succinate dehydrogenase or fumarate reductase, flavoprotein subunit	8	5	13	18	0
			Fumarase_DiC4HB	L(+)-Tartrate dehydratase class I, alpha subunit	0	31	31	65	37
			Mdh_DiC4HB	Malate dehydrogenase (NAD)	0	9	9	18	12
			PEP_synthase	Phosphoenolpyruvate synthase (pyruvate,water dikinase)	0	23	23	47	25
			PEPC	Phosphoenolpyruvate carboxylase	1	15	16	31	16
			Por_DiC4HB	Pyruvate synthase	3	34	37	71	42
			Suc_CoA_red_DiC4HB	Succinate-semialdehyde dehydrogenase (NAD(P)(+))	1	2	3	5	4
			SucA_DiC4HB	Succinyl-CoA synthetase, subunit alpha	0	18	18	36	21

				Dicarboxylate/4-hydroxybutyrate cycle Total	*24*	*181*	*205*	*394*	*215*
		**multiple systems**	Pcc	Propionyl-CoA carboxylase	31	474	505	995	0
				Multiple systems Total	*31*	*474*	*505*	*995*	*0*
		**Reductive acetyl–CoA pathway**	Codh	Carbon monoxide dehydrogenase large subunit	60	259	319	765	151
			Fthfs	Formate--tetrahydrofolate ligase	213	782	995	2175	1151
				Reductive acetyl–CoA pathway Total	*273*	*1041*	*1314*	*2940*	*1302*
		**Reductive tricarboxylic acid cycle**	Aclb	Succinyl-CoA synthetase, beta subunit	17	75	92	181	31
			AcnA	Aconitate hydratase 2	1	62	63	185	70
			Ccl	Citrate synthase	3	8	11	21	14
			FrdA_rTCA	Succinate dehydrogenase, flavoprotein subunit	4	88	92	312	95
			Icd	Isocitrate dehydrogenase	0	6	6	15	7
			Mdh	Malate dehydrogenase	3	53	56	117	63
			OorA	2-Ooxoglutarate ferredoxin oxidoreductase, alpha subunit	0	43	43	96	46
			SucD	Succinyl-CoA synthetase, alpha subunit	2	39	41	131	46
				Reductive tricarboxylic acid cycle Total	*30*	*374*	*404*	*1058*	*372*
		Carbon Fixation Total			***828***	***5683***	***6511***	***14083***	***6475***
	**Methane Metabolism**	**Methane oxidation**	Mmox	Soluble methane monooxygenase hydroxylase component alpha-subunit	13	17	30	50	43
			Pmoa	Particulate methane monooxygenase	97	30	127	191	203
				Methane oxidation Total	*110*	*47*	*157*	*241*	*246*
		**Methanogenesis**	AckA	Acetate kinase	1	2	3	5	0
			ACS	AMP-dependent synthetase and ligase	1	3	4	7	0
			CdhC_methane	Acetyl-CoA decarbonylase/synthase complex subunit beta	1	8	9	21	0
			FmdB_fwdB	Molybdenum formylmethanofuran dehydrogenase subunit B	4	77	81	160	0
			Ftr	Formylmethanofuran/tetrahydromethanopterin N-formyltransferase	1	40	41	85	0
			HdrB	CoB--CoM heterodisulfide reductase	18	266	284	575	0
			Hmd	H(2)-dependent methylenetetrahydromethanopterin dehydrogenase	2	27	29	60	0
			Mch_methane	N(5),N(10)-methenyltetrahydromethanopterin cyclohydrolase	0	40	40	80	0
			McrA	Methyl-coenzyme M reductase alpha subunit	123	71	194	386	319
			Mer_methane	5,10-methylenetetrahydromethanopterin reductase	2	28	30	58	0
			MrtH	Tetrahydromethanopterin S-methyltransferase, MtrH subunit	3	64	67	132	0
			MT2	Methylcobalamin:coenzyme M methyltransferase	1	37	38	75	0
			MtaB	Methanol-cobalamin methyltransferase B subunit	1	28	29	58	0
			MtaC	Methanol corrinoid protein	0	12	12	24	0
			MtbB	Dimethylamine:corrinoid methyltransferase	0	10	10	22	0
			MtbC_mttC	Methyltransferase cognate corrinoid protein	0	27	27	58	0
			MtmB	Monomethylamine methyltransferase MtmB	0	9	9	27	0
			MtmC	Methyltransferase cognate corrinoid protein	0	8	8	18	0
			MttB	Trimethylamine methyltransferase	1	13	14	27	0
			MtxX	Methyltransferase	1	10	11	21	0
			Pta	Phosphotransacetylase	0	3	3	6	0
				Methanogenesis Total	*160*	*783*	*943*	*1905*	*319*
		Methane Metabolism Total			***270***	***830***	***1100***	***2146***	***565***
	Carbon Cycling Total				***4683***	***20062***	***23615***	***55158***	***23425***
**N Cycling**	**Ammonification**		gdh	Glutamate dehydrogenase	74	322	396	794	474
			Urec	Urease/ Urea amidohydrolase, alpha subunit	126	436	562	1157	626
		Ammonification Total			***200***	***758***	***958***	***1951***	***1100***
	**Anammox**		Hzo	Hydrazine oxidoreductase	16	5	21	50	36
			Hzsa	Hydrazine synthase subunit A, partial	7	3	10	17	17
		Anammox Total			***23***	***8***	***31***	***67***	***53***
	**Assimilatory N reduction**		Narb	Nitrate reductase	14	53	67	161	78
			Nasa	Assimilatory nitrate reductase	71	90	161	287	185
			NiR	Ferredoxin-nitrite reductase	15	54	69	130	0
			Nira	Ferredoxin-nitrite reductase	26	38	64	111	90
			Nirb	Aassimilatory nitrite reductase large subunit	8	95	103	214	130
		Assimilatory N reduction Total			***134***	***330***	***464***	***903***	***483***
	**Denitrification**		CnorB	Nitric oxide reductase subunit B	8	16	24	42	0
			Narg	Dissimilatory membrane-bound nitrate reductase	509	243	752	1165	923
			Nirk	Nitrite reductase	227	109	336	628	500
			Nirs	Nitrite reductase	340	119	459	722	619
			Norb	Nitric-oxide reductase	43	51	94	157	124
			Nosz	Nitrous oxide reductase	212	439	651	1396	802
		Denitrification Total			***1339***	***977***	***2316***	***4110***	***2968***
	**Dissimilatory N reduction**		Napa	Periplasmic nitrate reductase	125	105	230	370	239
			Nrfa	Formate-dependent cytochrome c nitrite reductase, c552 subunit	36	255	291	695	250
		Dissimilatory N Reduction Total			***161***	***360***	***521***	***1065***	***489***
	**Nitrification**		Amoa	Ammonia monooxygenase	46	13	59	95	62
			Amoa_quasi	Ammonia monooxygenase	197	57	254	478	0
			Hao	Hydroxylamine oxidoreductase	33	28	61	102	81
			Nxra	Nitrite oxidoreductase alpha subunit, partial	1	1	2	7	2
		Nitrification Total			***277***	***99***	***376***	***682***	***145***
	**Nitrogen fixation**		Nifh	Dinitrogenase reductase	263	1068	1331	3114	1557
		Nitrogen Fixation Total			***263***	***1068***	***1331***	***3114***	***1557***
	Nitrogen Cycling Total				***2397***	***3600***	***5997***	***11892***	***6795***
**P Cycling**	**Phosphorous oxidation**		5f1_htxA	Hypophosphite dioxygenase, alpha-ketoglutarate-dependent	1	1	2	3	0
			5f1_ptxD	Phosphonate dehydrogenase	2	9	11	25	0
		Phosphorous Oxidation Total			***3***	***10***	***13***	***28***	***0***
	**Phytic acid hydrolysis**		Phytase	Phytase	98	137	235	408	257
		Phytic Acid Hydrolysis Total			***98***	***137***	***235***	***408***	***257***
	**Polyphosphate degradation**		5f1_ppk2	Polyphosphate Kinase	431	606	1037	1795	0
			5f1_ppn	Endopolyphosphatase/metaphosphatase	28	14	42	50	0
			Ppx	Exopolyphosphatase	266	1184	1450	3065	1600
		Polyphosphate degradation Total			***725***	***1804***	***2529***	***4910***	***1600***
	**Polyphosphate synthesis**		Ppk	Polyphosphate kinase	134	349	483	899	562
		Polyphosphate Synthesis Total			***134***	***349***	***483***	***899***	***562***
	Phosporous Cycling Total				***960***	***2300***	***3260***	***6245***	***2419***
**S Cycling**	**Adenylylsulfate reductase**		AprA	Adenylylsulfate reductase	68	10	78	95	0
			Aps_apra	Adenylylsulfate reductase	154	68	222	330	323
			APS_AprB	Adenylylsulfate reductase	44	62	106	193	136
		Adenylylsulfate Reductase Total			***266***	***140***	***406***	***618***	***459***
	**DMSP degradation**		5f1_DMSP_lyase	Dimethylsulfonioproprionate lyase/dimethylsulfoniopropionate lyase/DMSP lyase/Dimethylpropiothetin dethiomethylase	18	9	27	62	0
			DmdA	Dimethyl sulfoniopropionate demethylase/DMSP demethylase	8	77	85	167	86
		DMSP Degradation Total			***26***	***86***	***112***	***229***	***86***
	**Sulfide Oxidation**		Fccab	Flavocytochrome c sulfide dehydrogenase	27	104	131	236	143
			Sqr	Sulfide-quinone reductase, putative	35	102	137	297	158
		Sulfide Oxidation Total			***62***	***206***	***268***	***533***	***301***
	**Sulfite reduction**		Dsra	Anaerobic sulfite reductase, subunit C	788	365	1153	1612	1395
			DsrB	Dissimilatory sulfite reductase subunit B	563	216	779	1080	940
			Sir	Sulfide:quinone oxidoreductase	78	239	317	583	365
			CysJ	Sulfite reductase (NADPH) flavoprotein subunit alpha	51	358	409	865	475
			CysI	Sulfite reductase hemoprotein beta component	47	262	309	646	397
		Sulfite Reduction Total			***1527***	***1440***	***2967***	***4786***	***3572***
	**Sulfur Oxidation**		SoxA	Sulfur oxidation protein (SoxA)	12	33	45	84	63
			SoxB	Sulfur oxidation protein SoxB	15	31	46	194	114
			SoxC	Sulfite dehydrogenase	12	43	55	172	78
			SoxV	SoxV gene product	1	15	16	31	16
			SoxY	Ssulfur oxidation protein SoxZ	48	323	371	783	287
		Sulfur Oxidation Total			***88***	***445***	***533***	***1264***	***558***
	Sulfur Cycling Total				***1969***	***2317***	***4286***	***7430***	***4976***

Categories Related to Microbial Response to Environmental Conditions

**Metal Homeostasis**	**Aluminum**	**Transport**	Al	Aluminum resistance protein		30	316	346	943	0
		Aluminum Total				***30***	***316***	***346***	***943***	***0***
	**Arsenic**	**Detoxification**	Aoxb	Arsenite oxidase		83	75	158	372	245
			Arra	Respiratory arsenate reductase		37	52	89	224	115
			Arsc	Arsenate reductase		70	946	1016	2605	1098
			Arsm	Arsenite S-adenosylmethyltransferase		19	71	90	175	102
			Arxa	Anaerobic arsenite oxidase		0	3	3	6	4
					Arsenic Detoxification Total	*209*	*1147*	*1356*	*3382*	*1564*
		**Transport**	ArsB	Arsenical pump membrane protein, putative		115	763	878	1930	0
					Arsenic Transport Total	*115*	*763*	*878*	*1930*	*0*
		Arsenic Total				***324***	***1910***	***2234***	***5312***	***1564***
	**Boron**	**Transport**	Atr1	Borate transporter		11	8	19	27	0
			Bor1	Boron efflux transporter, putative		3	6	9	15	0
		Boron Total				***14***	***14***	***28***	***42***	***0***
	**Cadmium**	**Transport**	CadA	Cadmium ATPase		101	903	1004	2150	*0*
			CadBD	Cadmium resistance protein		34	108	142	295	*0*
		Cadmium Total				***135***	***1011***	***1146***	***2445***	***0***
	**Cadmium,Cobalt,Zinc**	**Transport**	CzcA	Heavy metal efflux pump, CzcA family		39	475	514	1016	0
			CzcC	Cation efflux system membrane protein czcC		11	78	89	178	0
			CzcD	Cobalt-zinc-cadmium resistance protein czcD		87	541	628	1209	0
		Cadmium, Cobalt, Zinc Total				***137***	***1094***	***1231***	***2403***	***0***
	**Calcium**	**Transport**	Ca_vacuolar_exchanger	Vacuolar calcium ion transporter		34	18	52	63	*0*
			ChaA	Sodium/calcium exchanger membrane region		8	117	125	261	*0*
		Calcium Total				***42***	***135***	***177***	***324***	***0***
	**Chromium**	**Detoxification**	Chrr	Putative NADPH-dependent FMN reductase		7	31	38	84	58
					Chromium Detoxification Total	*7*	*31*	*38*	*84*	*58*
		**Transport**	ChrA	Chromate reductase		244	1592	1836	3839	0
					Chromium Transport Total	*244*	*1592*	*1836*	*3839*	*0*
		Chromium Total				***251***	***1623***	***1874***	***3923***	***58***
	**Cobalt**	**Transport**	CorC	Hypothetical 33.3 kDa protein		22	205	227	491	*0*
		Cobalt Total				***22***	***205***	***227***	***491***	***0***
	**Cobalt,Nickel**	**Transport**	CnrA	Heavy metal efflux pump, CzcA family		1	21	22	49	0
			CnrC	Putative nickel and cobalt resistance protein		1	4	5	15	0
			RcnA	Nickel/cobalt efflux protein RcnA		9	19	28	59	0
		Cobalt, Nickel Total				***11***	***44***	***55***	***123***	***0***
	**Cobalt/Magnesium**	**Transport**	CorA	Cobalt/Magnesium efflux protein CorC		181	1584	1765	3811	*0*
		Cobalt/Magnesium Total				***181***	***1584***	***1765***	***3811***	***0***
	**Copper**	**Detoxification**	Cueo	Multicopper oxidase family protein		5	57	62	130	57
			PcoA	Copper resistance protein A		2	14	16	32	0
					Copper Detoxification Total	*7*	*71*	*78*	*162*	*57*
		**Transport**	CopA	Copper-exporting ATPase		123	1249	1372	2966	0
			CusA	Outer membrane copper (silver) and drug transport protein (RND family)		4	26	30	63	0
			CusC	Cation efflux system protein cusC		0	6	6	12	0
			CusF	Cation efflux system protein cusF		26	204	230	463	0
			CutA	Divalent cation tolerance protein		18	415	433	906	0
			PcoC	Copper resistance protein C		0	1	1	2	0
			PcoE	Putative copper-binding protein PcoE		0	2	2	4	0
			YcnJ	Copper transport protein YcnJ		2	7	9	24	0
					Copper Transport Total	*173*	*1910*	*2083*	*4440*	*0*
		Copper Total				***180***	***1981***	***2161***	***4602***	***57***
	**Iron**	**Oxidation**	Rusticyanin	Rusticyanin		0	1	1	2	*0*
					Iron Oxidation Total	*0*	*1*	*1*	*2*	*0*
		**Storage**	Bfr	bacterioferritin		34	360	394	810	0
			Dps	DNA-binding ferritin-like protein		39	859	898	2032	0
					Iron Storage Total	*73*	*1219*	*1292*	*2842*	*0*
		**Transport**	CirA	Colicin I receptor		187	1449	1636	3470	*0*
			EntB	2,3-dihydroxybenzoate-AMP ligase		1	29	30	59	*0*
			FecA	Dicitrate transport protein		30	258	288	597	*0*
			FeoB	Ferrous iron transport protein B		126	1504	1630	3707	*0*
			FepA_iron	Ferric enterobactin protein receptor		80	714	794	1663	*0*
			FhuA	Putative ferrichrome-iron receptor FhuA		17	81	98	212	*0*
			FhuE	Ferric-rhodotorulic acid outer membrane transporter		57	356	413	862	*0*
			Fiu	Catecholate siderophore receptor		30	270	300	603	*0*
					Iron Transport Total	*528*	*4661*	*5189*	*11173*	*0*
		Iron Total				***601***	***5881***	***6482***	***14017***	***0***
	**Lead**	**Transport**	PbrA	Heavy metal translocating P-type ATPase		8	32	40	75	0
			PbrD	Lead binding protein		0	1	1	4	0
			PbrT	Lead uptake protein		2	11	13	33	0
		Lead Total				***10***	***44***	***54***	***112***	***0***
	**Magnesium**	**Transport**	MgtA	Magnesium-translocating P-type ATPase		189	1769	1958	5012	*0*
			MgtC	Mg2+ transport P-type ATPase C		1	5	6	13	*0*
			MgtE	Magnesium (Mg2+) transporter-like protein		144	1401	1545	3378	*0*
		Magnesium Total				***334***	***3175***	***3509***	***8403***	***0***
	**Manganese**	**Transport**	MntH_Nramp	NRAMP family Mn2/Fe2 transporter		138	934	1072	2330	0
			PsaA_5f0_Mn	Manganese ABC transporter substrate-binding lipoprotein		30	261	291	607	0
		Manganese Total				***168***	***1195***	***1363***	***2937***	***0***
	**Mercury**	**Detoxification**	Mer	Pyridine mercuric reductase		83	603	686	1999	728
			MerB	Alkylmercury lyase		12	55	67	133	86
			MetC	Putative mercury transport protein MerC		3	15	18	70	24
					Mercury Detoxification Total	*98*	*673*	*771*	*2202*	*838*
		**Transport**	MerE	Mercuric transport protein		5	3	8	17	0
			MerF	Mercury ion transport protein		4	21	25	59	0
			MerG	Organomercury resistance protein		1	1	2	3	2
			MerH	Mercuric transporter MerH		0	1	1	2	0
			MerP	Heavy metal translocating P-type ATPase		17	101	118	243	0
			MerT	Mercuric transport protein MerT		23	112	135	306	0
					Mercury Transport Total	*50*	*239*	*289*	*630*	*2*
		Mercury Total				***148***	***912***	***1060***	***2832***	***840***
	**Multiple metals**	**Storage**	SmtA	Metallothionein		3	31	34	122	0
					Multiple Metals Storage Total	*3*	*31*	*34*	*122*	*0*
		**Transport**	RndA	Cation efflux system protein		158	1146	1304	2671	0
					Multiple Metals Transport Total	*158*	*1146*	*1304*	*2671*	*0*
		Multiple metals Total				***161***	***1177***	***1338***	***2793***	***0***
	**Nickel**	**Transport**	NiCoT	High-affinity nickel-transport protein		75	246	321	609	0
			NikA	Periplasmic nickel-binding protein		447	2374	2821	5624	0
			NikC	Nickel transport system permease protein NikC		7	28	35	68	0
			NreB	Nickel resistance protein/MFS family nickel efflux transporter protein B		7	68	75	110	0
		Nickel Total				***536***	***2716***	***3252***	***6411***	***0***
	**Potassium**	**Transport**	KdpA	Potassium ion P-type ATPase transporter		54	474	528	1119	0
			KefBC	Glutathione-regulated potassium-efflux system protein		67	531	598	1224	0
			KtrBD	Trk-type potassium uptake protein KtrB		8	133	141	291	0
			Kup	Low affinity potassium transport system protein Kup		33	243	276	560	0
			TrkA	Potassium transporter peripheral membrane component		97	1102	1199	2480	0
			TrkGH	H(+)-transporting two-sector ATPase		105	1342	1447	3290	0
		Potassium Total				***364***	***3825***	***4189***	***8964***	***0***
	**Selenium**	**Detoxification**	Se	Selenate reductase subunit alpha (Selenatereductase molybdenum subunit)		2	2	4	6	0
		Selenium Total				***2***	***2***	***4***	***6***	***0***

	Silver	Transport	SilA	Putative cation efflux system protein SilA	22	91	113	209	0
			SilC	Outer membrane lipoprotein silC	38	293	331	704	0
			SilE	Silver-binding protein silE	1	0	1	1	0
			SilP	Silver efflux system P-type ATPase	166	530	696	1304	0
		Silver Total			***227***	***914***	***1141***	***2218***	***0***
	Sodium	Transport	MrpA	Na(+)/H(+) antiporter subunit A	71	244	315	588	0
			NatB	ABC transporter sodium permease	31	383	414	866	0
			NhaA	Sodium/proton antiporter nhaA	55	607	662	1405	0
			NhaB	Sodium/proton antiporter nhaB	17	123	140	280	0
			NhaC	Sodium/proton antiporter nhaC	129	0	129	129	0
			NhaD	Sodium/proton antiporter nhaD	31	245	276	561	0
			NhaP	Sodium/proton antiporter nhaP	234	1317	1551	3349	0
			NqrB	Na(+)-translocating NADH-quinone reductase subunit B	34	258	292	594	0
		Sodium Total			***602***	***3177***	***3779***	***7772***	***0***
	Tellurium	Detoxification	TehB	Tellurite resistance protein TehB	28	352	380	1435	339
			TerC	Tellurium resistance protein terC	60	890	950	3749	1081
				Tellurium Detoxification Total	*88*	*1242*	*1330*	*5184*	*1420*
		Transport	TerD	Tellurium resistance protein terD	51	171	222	416	0
			TerZ	Tellurium resistance protein terZ	10	144	154	306	0
			TerZD	Tellurium resistance protein	86	660	746	1599	0
				Tellurium Transport Total	*147*	*975*	*1122*	*2321*	*0*
		Tellurium Total			***235***	***2217***	***2452***	***7505***	***1420***
	Zinc	Transport	AdcA	Zinc-binding lipoprotein AdcA	6	68	74	152	0
			Cot1	Metal cation transporter	6	7	13	24	0
			Msc2	Metal cation transporter	6	13	19	31	0
			TroA	Zinc ABC transporter, periplasmic zinc-binding protein	1	17	18	38	0
			Yiip_fieF	Cation-efflux pump FieF	12	72	84	164	0
			ZitB	Cation diffusion facilitator family transporter	16	128	144	284	0
			ZntA	Heavy metal translocating P-type ATPase	88	415	503	1254	0
			ZnuA	High-affinity zinc transporter periplasmic component	33	261	294	633	0
			ZnuC	Zinc import ATP-binding protein ZnuC	119	1270	1389	3093	0
			Zrc1	Zinc/cadmium resistance protein	7	6	13	19	0
			Zrt1	Zinc-regulated transporter 1/High-affinity zinc transport protein zrt1	29	23	52	73	0
			Zrt2	Zinc-regulated transporter 2/Low-affinity zinc transport protein ZRT2	24	20	44	65	0
			Zrt3	Zinc-regulated transporter 3/Vacuolar membrane zinc transporter	7	8	15	23	0
			ZupT_ygiE	Zinc transporter ZupT	15	83	98	228	0
		Zinc Total			***369***	***2391***	***2760***	***6081***	***0***
	Metal Homeostasis Total				***5084***	***37543***	***42627***	***94470***	***3939***
**Organic Contaminant Degradation**	**Aromatics**	**Aromatic alpha hydroxy acid**	Mdla	Mandelate racemase	40	206	246	580	32
				Aromatic alpha hydroxy acid Total	*40*	*206*	*246*	*580*	*32*
		**Aromatic carboxylic acid**	badi	2-Ketocyclohexane-1-carboxyl-CoA hydrolase	2	11		28	13
			badk	Cyclohex-1-ene-1-carboxyl-CoA hydratase	0	3		7	3
			Bco	Benzoyl-CoA reductase, subunit A	22	54	76	160	23
			bend	Cis-diol dehydrogenase					65
			Bph	Benzoate 4-monooxygenase cytochrome P450, putative	83	37	120	133	33
			Hcaacd	3-phenylpropionate dioxygenase alpha subunit (digoxigenin alph subunit)	2	10	12	37	14
			hcab	2,3-Dihydroxy-2,3-dihydro-phenylpropionate dehydrogenase					39
			mdlb	L-mandelate dehydrogenase					36
			mdlc	Benzoylformate decarboxylase					196
			Mhpa	3-(3-hydroxy-phenyl)propionate hydroxylase	36	29	65	92	73
			mhpb	3-(2,3-dihydroxyphenyl)propionate 1,2-dioxygenase					41
			mhpc	2-hydroxy-6-ketonona-2,4-dienedoic acid hydrolase					20
			Nagg	Putative Salicylate 1-monooxygenase	196	261	457	778	148
			Ophc	4,5-dihydroxyphthalate decarboxylase	1	30	31	77	32
			Phta	Phthalate 4,5-dioxygenase	19	92	111	222	24
			phtb	Phthalate 4,5-cis-dihydrodiol dehydrogenase					8
			Poba	4-Hydroxybenzoate 3-monooxygenase	27	272	299	917	320
			pobmo	Phenoxybenzoate dioxygenase	0	4		9	4
			thl	Pyrogallol hydroxytransferase	2	6		14	8
			Tpha	Putative terephthalate 1,2-dioxygenase, large subunit	1	7	8	15	9
			tphb	Terphthalate dihydrodiol dehydrogenase					3
			xylg	2-Hydroxymuconic semialdehyde dehydrogenase					147
			Xyll	2-Hydro-1,2-dihydroxybenzoate dehydrogenase	8	23	31	61	31
			Xylxy	Toluate 1,2-dioxygenase subunit alpha	1	10	11	25	14
				Aromatic carboxylic acid Total	*400*	*849*	*1221*	*2575*	*1304*
		**BTEX and related aromatics**	Apc	Related to beta-subunit of acetone carboxylase, possible subunit of acetophenone carboxylase	0	16	16	32	3
			Bbs	Putative E-phenylitaconyl-CoA hydratase	1	5	6	10	5
			bbsg	Benzylsuccinyl CoA dehydrogenase	1	4		8	5
			Catb	Muconate cycloisomerase	34	211	245	538	154
			EbdA	Ethylbenzene dehydrogenase, beta subunit	0	2	2	4	0
			Ebdabc	Ethylbenzene dehydrogenase, beta subunit	0	5	5	13	3
			Hbh	4-Hydroxybenzoate hydroxylase	18	69	87	522	87
			Pchcf	4-Cresol dehydrogenase	6	31	37	75	34
			Tmoabe	Toluene-4-monooxygenase system protein A	1	13	14	27	14
			Toma	Toluene 2-monooxygenase	2	10	12	22	8
			Tutfdg	Benzylsuccinate synthase	25	146	171	389	40
			xylc	Benzaldehyde dehydrogenase					71
			xylj	2-Oxopent-4-enoate hydratase					149
			Xylm	Xylene monooxygenase subunit 1	3	3	6	11	7
				BTEX and related aromatics Total	*91*	*515*	*601*	*1651*	*580*
		**Chlorinated aromatics**	Cbaa	3-Chlorobenzoate-3,4-dioxygenase oxygenase subunit	1	3	4	7	4
			Cbea	Achlorobenzoate 1,2-dioxygenase subunit alpha	1	3	4	7	4
			Fcba	4-Chlorobenzoyl CoA ligase	2	9	11	24	12
			fcbc	4-Hydroxybenzoyl CoA thioesterase	1	1		6	2
			fcbb	4-Chlorobenzoyl-CoA dehalogenase	1	2		10	3
			Tfda	2,4-D/alpha-ketoglutarate dioxygenase	100	305	405	803	184
			Tfdb	2,4-Dichlorophenol 6-monooxygenase	16	35	51	108	52
			Tfta	2,4,5-Trichlorophenoxyacetic acid oxygenase	1	0	1	1	1
			Tfth	Hydroxyquinol 1,2-dioxygenase	36	117	153	285	90
				Chlorinated aromatics Total	*159*	*475*	*629*	*1251*	*352*
		**Heterocyclic aromatics**	cara	Carbazole 1,9a-dioxygenase	3	16		62	19
			dbdc	Dibenzothiophene dioxygenase	1	1		2	2
			dbdd	Dibenzothiophene dihydrodiol dehydrogenase	2	3		7	5
			DbfB	2,2,3-Trihydroxy-biphenyl dioxygenase	1	0	1	1	0
			DbtAc	Dibenzothiophene dioxygenase	1	0	1	1	0
			Dxna	Alpha subunit of dibenzofuran dioxygenase	7	1	8	21	13
			Hdno	6-Hydroxy-D-nicotine oxidase	7	4	11	16	4
			Nicdehydr	Nicotine dehydrogenase chain A	0	19	19	42	8
				Heterocyclic aromatics Total	*22*	*44*	*40*	*152*	*51*
		**Nitoaromatics**	mhqa	Hydroquinone 1,2-dioxygenase					4
			Nbac	3-Hydroxyanthranilate 3,4-dioxygenase	17	88	105	225	98
			Nbza	Nitrobenzene nitroreductase	1	0	1	1	1
			nbzb	Hydroxylaminobenzene mutase	0	6		100	6
			nbzc	2-Aminophenol 1,6-dioxygenase	1	3		9	4
			Nfsa_2	Oxygen-insensitive NADPH nitroreductase	43	430	473	1818	121
			Nfsb_2	Oxygen-insensitive NAD(P)H nitroreductase/Dihydropteridine reductase/FMN-dependent nitroreductase	51	439	490	1162	55
			Nhh	Nitrile hydratase	34	186	220	593	148
			Nitroreductase_1	Nitroreductase	51	554	605	1360	0

			Nitroreductase_b	Nitroreductase	42	174	216	454	0
			Npha	Nitrophenol monooxygenase	1	0	1	1	2
			Oxdb	Phenylacetaldoxime dehydratase	8	46	54	108	56
				Nitoaromatics Total	*249*	*1926*	*2165*	*5831*	*495*
		**Other aromatics**	Amie	Aliphatic amidase	7	68	75	185	88
			Arylest	Arylesterase	41	373	414	1254	355
			Badh	Benzylalcohol dehydrogenase	89	199	288	638	308
			Catechol	Catechol 1,2-dioxygenase	85	292	377	945	235
			Catechol_b	Catechol 2,3-dioxygenase	37	155	192	548	232
			Cdd	2-Hydroxy-3-carboxy-6-oxo-7-methylocta-2,4-dienoate decarboxylase	4	10	14	24	0
			Cdo	2,3-Dihydroxy-p-cumate dioxygenase	1	2	3	5	3
			Cmci	3-Carboxy-ciscis-muconate cycloisomerase	36	139	175	440	191
			Cmtab	p-Cumate dioxygenase large subunit (CmtAb)	1	6	7	16	7
			cumb	Dihydroxyisopropylbenzene dehydrogenase	1	1		3	2
			cumc	3-Isopropylcatechol-2,3-dioxygenase	1	3		11	4
			cumd	2-Hydroxy-6-oxohepta-2,4-dienoate hydrolase	1	2		5	3
			cyma	p-Cymene methyl hydroxylase	2	6		15	8
			cymc	Aryl-aldehyde dehydrogenase	1	4		9	5
			mdld	Benzaldehyde dehydrogenase (NAD(+))	5	32		122	37
			Mult_ring_12DiOx	Multi-ring 1,2-Dioxygenase	97	190	287	977	206
			Nitrilase	Nitrilase	66	256	322	705	207
			One_ring_12diox	One-ring 1,2-Dioxygenase	37	143	180	522	148
			One_ring_23diox	One-ring 2,3-Dioxygenase	92	215	307	1019	313
			Pcag	Protocatechuate 3,4-dioxygenase, beta subunit	53	255	308	746	210
			ProO	Protocatechuate 4,5-dioxygenase	20	90	110	269	69
			Tdnb	Aniline dioxygenase	6	1	7	8	8
			Xlnd	4-Hydroxybenzoate 3-monooxygenase	12	21	33	445	30
				Other aromatics Total	*695*	*2463*	*3099*	*8911*	*2669*
		**Polycyclic aromatics**	bphb	2,3-Dihydro-2,3-dihydroxybiphenyl dehydrogenase	4	25		88	29
			bphc	2,3-Dihydroxybiphenyl 1,2-dioxygenase	25	125		422	150
			bphd	2-Hydroxy-6-oxo-6-phenylhexa-2,4-dienoate hydrolase	33	151		696	184
			BphF1	4-Hydroxy 2-oxovalerate aldolase	57	296	353	969	337
			nahb	1,2-Dihydroxy-1,2-dihydronaphthalene dehydrogenase	3	4		10	7
			nahc_2	1, 2-Dihydroxynaphthalene dioxygenase	1	1		3	2
			nahf	Salicyladehyde dehydrogenase	4	13		34	17
			phdci	2-Hydroxychromene-2-carboxylate isomerase	1	18		57	19
			phdi	1-Hydroxy-2-naphthoate dioxygenase	0	2		6	2
			phdj	Trans-2-carboxybenzalpyruvate hydratase	2	1		3	3
			Qorl	Quinoline 2-oxidoreductase large subunit	0	9	9	18	5
			Quinoline	Putative 2-oxo-1 2-dihydroquinoline 8-monooxygenase oxygenase subunit	3	7	10	19	10
			Phdk	2-Carboxybenzaldehyde dehydrogenase	4	11	15	25	17
				Polycyclic aromatics Total	137	663	387	2350	782
		Aromatics Total			***1793***	***7141***	***8388***	***23301***	***6265***
	**Chlorinated solvents**		Dcma	Dichloromethane dehalogenase	1	1	2	3	2
			Cmua	Uroporphyrinogen decarboxylase (URO-D)	4	24	28	139	26
			Dehh	Haloacetate dehalogenase	7	50	57	147	57
			Dehh109	haloacid dehalogenase, type II:HAD-superfamily hydrolase, subfamily IA, variant 2	32	217	249	500	258
			Dl_dex	DL-2-haloacid dehalogenase	1	0	1	1	1
			Exaa(moxf)	Quinoprotein ethanol dehydrogenase	46	275	321	663	205
			Rd	Reductive dehalogenase, putative	26	148	174	690	223
		Chlorinated solvents Total			***117***	***715***	***832***	***2143***	***772***
	**Herbicides related compound**		Atza	Atrazine chlorohydrolase	50	367	417	988	240
			Atzb	Hydroxyatrazine hydrolase	17	102	119	278	123
			Atzc	N-isopropylammelide isopropylaminohydrolase	1	71	72	142	1
			Atzd	Cyanuric acid amidohydrolase	5	27	32	84	29
			Mauab	Methylamine dehydrogenase small subunit	19	42	61	118	9
			PcpA	2,6-Dichloro-p-hydroquinone 1,2-dioxygenase	4	4		10	8
			Pcpb	Pentachlorophenol 4-monooxygenase	11	50	61	128	45
			pcpc	Tetrachlorohydroquinone reductive dehalogenase	1	0		1	1
			pcpe	Maleylacetate reductase	34	161		418	195
			Phn	Phosphonate C-P lyase system protein PhnL	47	370	417	1182	324
			Tcpa	Chlorophenol 4-monooxygenase	1	0	1	1	3
			Trza	N-ethylammeline chlorohydrolase	5	23	28	56	24
			Trze	Biuret hydrolase	5	34	39	77	41
			Trzn	Triazine hydrolase	18	68	86	267	33
		Herbicides related compound Total			***218***	***1319***	***1333***	***3750***	***1076***
	**Other Hydrocarbons**		Alkb	Alkane 1-monooxygenase	90	274	364	1252	432
			alkh	Aldehyde dehydrogenase	3	16		35	19
			alkj	Alcohol dehydrogenase	6	17		46	23
			Assa	Alkylsuccinate synthase	22	11	33	53	21
			Bmo	Butane monooxygenase beta subunit	9	2	11	11	2
			chna	Cyclohexanol dehydrogenase	2	21		40	23
			chnb	Cyclohexanone 1,2-monooxygenase	27	179		434	206
			chnc	1-Oxa-2-oxocycloheptane lactonase	6	15		30	21
			chnd	6-Hydroxyhexanoate dehydrogenase	11	26		66	37
			chne	6-Oxohexanoate dehydrogenase	1	15		29	16
			Cpna	Cyclopentanol dehydrogenase	10	35	45	84	44
			cpnb	Cyclopentanone 1,2-monooxygenase	8	11		30	19
			cpnc	5-Valerolactone hydrolase	2	9		27	11
			cpne	5-Oxovalerate dehydrogenase	1	4		9	5
			Xamo	Phenol 2-monooxygenase	18	45	63	117	20
		Other Hydrocarbons Total			***216***	***680***	***516***	***2263***	***899***
	**Others**		alkk	Medium-chain acyl-CoA synthetase	28	171		400	199
			Aln	Nitrilase	13	47	60	344	59
			CaaD	Cis-3-Chloroacrylic acid dehalogenase, putative	0	1	1	2	1
			Dmsa	Dimethyl sulfoxide reductase	7	81	88	1420	110
			Msad	Malonate semialdehyde decarboxylase	1	9	10	20	12
			Msmabcd	Dimethyl sulfoxide reductase	2	11	13	23	11
			Nitro	Xenobiotic reductase	22	96	118	282	121
			Onr	Pentaerythritol tetranitrate reductase	0	1	1	2	2
			Scnabc	Thiocyanate hydrolase gamma subunit	5	26	31	332	22
			Sdsa	Taurine dioxygenase	6	12	18	32	22
			ThmAB	Xenobiotic reductase	3	0	3	3	6
		Other Organic Contaminant Degradation Total		*Total*	***87***	***455***	***343***	***2860***	***565***
	**Pesticides related compound**		Adpb	Aryldialkylphosphatase	3	20	23	51	10
			lina	Gamma-hexachlorocyclohexane dehydrochlorinase	0	1		37	1
			linb	Haloalkane dehalogenase	35	298		1118	333
			linc	2,5-Dichloro-2,5-cyclohexadiene-1,4-diol dehydrogenase	11	73		176	84
			lind	2,5-Dichlorohydroquinone reductive dechlorinase	0	1		15	1
		Pesticides related compound Total		*Total*	***49***	***393***	***23***	***1397***	***429***
	**Terpenoid**		Camdcab	Putative Camphor 5-monooxygenase (P450cam)	2	8	10	18	9
		Terpenoid Total		*Total*	***2***	***8***	***10***	***18***	***9***
	Organic Contaminant Degradation Total				***2482***	***10711***	***11445***	***35732***	***10015***
**Stress**	**Acidic shock**		Asr	Acid shock protein precursor, exported protein	0	11	11	24	0
		Acidic Shock Total			***0***	***11***	***11***	***24***	***0***
	**Alkaline shock**		Asp_5f0_stress	Alkaline shock protein	0	20	20	44	0
		Alkaline Shock Total			***0***	***20***	***20***	***44***	***0***
	**Anti-sigma factor (anti-E)**		RseA	Anti sigma-E protein, RseA	13	126	139	322	0

			RseB	Sigma-E factor negative regulatory protein RseB	4	77	81	184	0
		Anti-sigma Factor Total			***17***	***203***	***220***	***506***	***0***
	**Cold shock**		CspA	Cold shock protein	3	91	94	266	0
			CspB	Cold shock protein CspB	1	52	53	161	0
			CspG	Cold shock-like protein CspG	2	36	38	102	0
			DesK	Two-component sensor histidine kinase desk	5	43	48	119	0
			DesR	Two-component system, NarL family, response regulator DesR	2	29	31	66	0
		Cold Shock Total			***13***	***251***	***264***	***714***	***0***
	**Envelope stress**		BaeR	Transcriptional regulatory protein, C-terminal domain protein	6	35	41	81	0
			BaeS	Sensory histidine kinase in two-component regulatory system with BaeR	2	11	13	28	0
			CpxA	Two component system histidine kinase	19	187	206	674	0
			CpxP	Envelope stress induced periplasmic protein	1	10	11	20	0
			CpxR	CpxR family transcriptional regulator	22	272	294	955	0
			PspA	Phage shock protein A	38	491	529	1163	0
			PspB	Phage shock protein B	4	106	110	392	0
			PspC	phage shock protein C, PspC	38	345	383	809	0
			PspD	Phage shock protein D	0	7	7	17	0
			PspF	Phage shock protein F	40	232	272	556	0
		Envelope Stress Total			***170***	***1696***	***1866***	***4695***	***0***
	**Glucose limitation**		BglH	Aryl-phospho-beta-d-glucosidase	8	81	89	200	0
			BglP	Phosphotransferase system (PTS) beta-glucoside-specific enzyme IIBCA component	2	65	67	170	0
			CcpA	Catabolite control protein A	43	205	248	645	0
			CsiD	Carbon starvation-inducible protein	0	18	18	46	0
			Lrp	Leucine-responsive regulatory protein	16	250	266	726	0
		Glucose Limitation Total			***69***	***619***	***688***	***1787***	***0***
	**Heat shock**		ClpP	ATP-dependent Clp protease proteolytic subunit	259	1289	1548	3177	0
			DnaK	Molecular chaperone DnaK	0	165	165	614	0
			GroEL	Putative heat-shock protein hsp60	60	131	191	407	0
			GroES	Chaperonin Cpn10	16	57	73	134	0
			GrpE	GrpE protein	16	359	375	1139	0
			HrcA	Heat-inducible transcription repressor HrcA	91	500	591	1985	0
			HspR	Heat shock protein transcriptional repressor HspR2 (Hspr2 protein)	0	1	1	2	0
		Heat Shock Total			***442***	***2502***	***2944***	***7458***	***0***
	**Intracellular survival**		HspX	Heat shock protein hspX	0	5	5	10	0
			KatB	Catalase-peroxidase KatB	0	4	4	15	0
			KatG	Catalase/peroxidase HPI	0	1	1	2	0
		Intracellular Survival Total			***0***	***10***	***10***	***27***	***0***
	**Nitrogen limitation**		GlnA	Glutamine synthetase, putative	7	1037	1044	4662	0
			GlnR	HTH-type transcriptional regulator glnR	14	165	179	1179	0
			NtrB	Nitrogen regulation protein NtrB	25	176	201	391	0
			NtrC	Nitrogen regulation protein NR(I)	32	496	528	1137	0
			TnrA	Nitrogen sensing transcriptional regulator	1	10	11	45	0
		Nitrogen Limitation Total			***79***	***1884***	***1963***	***7414***	***0***
	**Osmotic stress**		DegS	Two-component sensor histidine kinase DegS	16	114	130	276	0
			DegU	Transcriptional regulatory protein degU	8	97	105	209	0
			KdpE	KDP operon transcriptional regulatory protein KdpE	17	466	483	2737	0
			MtrA	DNA-binding response regulator MtrA	6	54	60	125	0
			MtrB	Two component sensor histidine kinase mtrB	3	59	62	137	0
			OmpR	Osmolarity response regulator	57	742	799	1710	0
			OpuE	Osmoregulated proline transporter OpuE	1	38	39	140	0
			ProV	Glycine betaine/L-proline transport ATP-binding subunit	7	214	221	1305	0
			ProW	Putative glycine betaine/L-proline transport system permease protein ProW	3	18	21	44	0
			ProX	L-proline glycine betaine binding ABC transporter protein proX	16	14	30	120	0
		Osmotic Stress Total			***134***	***1816***	***1950***	***6803***	***0***
	**Oxidative stress**		AhpC	Alkyl hydroperoxide reductase C	145	789	934	2134	0
			AhpF	Alkyl hydroperoxide reductase, F subunit	57	431	488	1522	0
			KatA	Catalase	14	154	168	455	0
			OxyR	Hydrogen peroxide-inducible genes activator OxyR/DNA-binding transcriptional regulator OxyR	9	297	306	1361	0
			PerR	Peroxide-responsive repressor PerR	7	58	65	192	0
			SodA	Superoxide dismutase	102	687	789	1614	0
			SoxR_OR_marC	Redox-sensitive transcriptional activator SoxR	6	158	164	354	0
			SoxS	DNA-binding transcriptional regulator SoxS	7	108	115	238	0
		Oxidative Stress Total			***347***	***2682***	***3029***	***7870***	***0***
	**Oxygen limitation**		ArcA	Aerobic respiration control protein ArcA	1	75	76	251	0
			ArcB	Multi-sensor hybrid histidine kinase	21	90	111	214	0
			CydA	Cytochrome d ubiquinol oxidase, subunit I	2	74	76	381	0
			CydB	Cytochrome bd plastoquinol oxidase subunit 2 apoprotein	21	519	540	1410	0
			Fnr	Transcriptional regulator, Crp/Fnr family	2	1522	1524	3067	0
			NarH	Nitrate reductase (beta subunit)	6	185	191	837	0
			NarI	Nitrate reductase (gamma subunit)	35	450	485	1759	0
			NarJ	Nitrate reductase (delta subunit)	9	164	173	467	0
			NsrR	Nitrite-sensitive transcriptional repressor NsrR	3	116	119	327	0
			ResD	Transcriptional regulatory protein ResD	2	125	127	354	0
			ResE	Sensor histidine kinase ResE	6	56	62	168	0
		Oxygen Limitation Total			***108***	***3376***	***3484***	***9235***	***0***
	**Phosphate limitation**		PhoA	Vacuolar alkaline phosphatase	19	110	129	453	0
			PhoB	Two component transcriptional regulator, winged helix family	17	349	366	1273	0
			PstA	Phosphate ABC transporter, inner membrane subunit PstA	0	454	454	1495	0
			PstB	Phosphate ABC transporter, ATP-binding protein	52	972	1024	7285	0
			PstC	Phosphate ABC transporter, permease protein PstC	48	423	471	1945	0
			PstS	ABC-type phosphate transport system periplasmic component PstS	24	173	197	394	0
		Phosphate Limitation Total			***160***	***2481***	***2641***	***12845***	***0***
	**Protein stress**		ClpC	ATP-dependent Clp protease, ATP-binding subunit ClpC	9	258	267	1566	0
			CtsR	Transcriptional regulator CtsR	14	191	205	1124	0
			DegP	Serine protease	14	270	284	727	0
		Protein Stress Total			***37***	***719***	***756***	***3417***	***0***
	**General stress**		KatE	Catalase 2	88	434	522	2218	0
		General stress Total			***88***	***434***	***522***	***2218***	***0***
	**RNA-binding protein**		Hfq_5f0_stress	RNA chaperone Hfq	3	35	38	77	0
		RNA-binding protein Total			***3***	***35***	***38***	***77***	***0***
	**Sigma factor**		Ecf	RNA polymerase, sigma-24 subunit, ECF subfamily	29	51	80	157	0
			Sigma_24	RNA polymerase, sigma-24 subunit, ECF subfamily	161	2194	2355	4927	0
			Sigma_32	RNA polymerase sigma factor	50	428	478	1106	0
			Sigma_38	RNA polymerase sigma-38 factor	31	266	297	916	0
			Sigma_70	RNA polymerase, sigma 70 subunit, RpoD family	106	1046	1152	2423	0
		Sigma Factor Total			***377***	***3985***	***4362***	***9529***	***0***
	**Stringent response**		ObgE	GTPase	26	604	630	4355	0
			SpoT	(p)ppGpp synthetase, RelA/SpoT family	28	306	334	730	0
		Stringent Response Total			***54***	***910***	***964***	***5085***	***0***
	Stress Total				**2098**	**23634**	**25732**	**79748**	**0**
**Plant Growth Promotion**	**Adhesion**		Paa	Porcine attaching-effacing associated protein	0	2	2	4	0
			Hsp60	Heat shock protein 60	1	0	1	1	0
			Pyoverdin_pvcC	Pyoverdin chromophore biosynthetic protein pvcC	0	3	3	6	0
		Adhesion Total			***1***	***5***	***6***	***11***	***0***
	**Disease suppression**		HcnB	Hydrogen cyanide synthase HcnB	12	6	18	22	0
		Disease Suppression Total			***12***	***6***	***18***	***22***	***0***
	**Drought tolerance**		Tre_arc	Trehalose synthase	1	5	6	12	0
			Tre_fun	Alpha,alpha-trehalose-phosphate synthase	131	63	194	277	0

		Drought Tolerance Total			***132***	***68***	***200***	***289***	***0***
	**Phytohormone**		Cad	Cadaverine	0	3	3	7	0
			Cks	Xanthine dehydrogenase subunit A	1	0	1	1	0
			Eth	Ethylene-forming enzyme	2	0	2	2	0
			Gas	Gibberellin biosynthesis-related	1	1	2	3	0
			Ipya	Thiamine pyrophosphate protein TPP binding domain protein	3	23	26	49	0
			Nep	Necrosis and ethylene-inducing	5	10	15	13	0
			Spe	Spermine synthase	25	118	143	262	0
			Sped_ara	Spermine synthase / spermidine synthase	2	23	25	49	0
			Sped_bac	Spermine synthase / spermidine synthase	27	255	282	558	0
		Phytohormone Total			***66***	***433***	***499***	***944***	***0***
	**Stress**		Acc	1-Aminocyclopropane-1-carboxylate deaminase	35	109	144	257	0
		Stress Total			***35***	***109***	***144***	***257***	***0***
	**Antioxidant enzyme**		Cat_arc	Catalase	2	8	10	18	0
			Cat_bac	Catalase	80	381	461	978	0
			Cat_fun	Peroxisomal catalase	69	36	105	134	0
			Per_arc	Alkyl hydroperoxide reductase/ Thiol specific antioxidant/ Mal allergen	2	20	22	42	0
			Per_bac	Peroxidase	6	27	33	63	0
			Per_fun	Versatile peroxidase VPL1	111	66	177	279	0
		Antioxidant Enzyme Total			***270***	***538***	***808***	***1514***	***0***
	**Anti-pathogen**		Sid_arc	LucA/IucC Family protein	0	11	11	22	0
			Sid_bac	Iron transporter	22	133	155	311	0
			Sid_fun	Aerobactin siderophore biosynthesis protein iucB	43	28	71	91	0
			Sod_CuZn	Superoxide dismutase [Cu-Zn] precursor	34	103	137	256	0
			Sod_FeMn	Mn-dependent superoxide dismutase	252	447	699	1212	0
			Sod_nickel	Putative nickel-containing superoxide dismutase precursor (NISOD)	1	23	24	47	0
			Sped_fungi	Spermine synthase / spermidine synthase	15	6	21	27	0
			Xyla	Xylose isomerase	71	352	423	884	494
			Pec	Polygalacturonase	3	1	4	5	0
		Anti-pathogen Total			***441***	***1104***	***1545***	***2855***	***494***
	Plant Growth Promotion Total				***957***	***2263***	***3220***	***5892***	***494***
**Microbial Defense**	**Antibiotic resistance**	**Degradation**	B_lactamase	Beta-lactamase	40	143	183	363	229
			B_lactamase_A	Beta-lactamase	68	198	266	586	270
			B_lactamase_b	Beta-lactamase	5	3	8	16	9
			B_lactamase_C	Beta-lactamase	59	250	309	1140	0
			Fosa	Glutathione transferase fosA	3	30	33	72	36
			Fosb	Fosfomycin resistance protein FosB	2	26	28	61	32
			Fosx	Fosfomycin resistance family protein	1	7	8	19	8
			Tetx_resistance	TetX monooxygenase	1	2	3	5	4
			Vgb	Virginiamycin B lyase	14	77	91	191	95
				Degradation Total	*193*	*736*	*929*	*2453*	*683*
		**Subunit modification**	Qnr	Quinolone resistance protein	3	5	8	14	0
			Van	D-alanine--D-lactate ligase/Vancomycin/teicoplanin resistance protein	14	34	48	119	0
				Subunit Modification Total	*17*	*39*	*56*	*133*	*0*
		**Transporter**	ABC_antibiotic_transporter	ABC antibiotic transporter	182	922	1104	2426	0
			DrrC	Daunorubicin resistance protein C	2	1	3	4	0
			MATE_antibiotic	Multidrug transport protein (MATE family)	80	312	392	824	0
			Mex	Acr family drug resistance transporter	395	3328	3723	7936	0
			MFS_antibiotic	Major facilitator superfamily MFS_1	977	7000	7977	17235	0
			SMR_antibiotics	Putative multidrug transporter, SMR family, DMT Superfamily protein	63	952	1015	3232	0
			Tet	Tetracycline resistance protein	46	230	276	735	0
				Transporter Total	*1745*	*12745*	*14490*	*32392*	*0*
		Antibiotic Resistance Total			***1955***	***13520***	***15475***	***34978***	***683***
	**Antimicrobial biosynthesis**	**Bacilysin**	BacA	Bacilysin biosynthesis	2	19	21	42	0
				Bacilysin Total	*2*	*19*	*21*	*42*	*0*
		**Chloramphenicol**	PabA	Aminodeoxychorismate synthase subunit II, component of p-aminobenzoate synthase multienzyme complex	5	27	32	61	0
				Chloramphenicol Total	*5*	*27*	*32*	*61*	*0*
		**Gramicidin**	LgrD	Linear gramicidin synthetase subunit D	1	7	8	15	0
				Gramicidin Total	*1*	*7*	*8*	*15*	*0*
		**Lantibiotic**	EpiA	Epidermin	0	1	1	2	0
			SpaR	Subtilin biosynthesis regulatory protein SpaR	0	11	11	22	0
				Lantibiotic Total	*0*	*12*	*12*	*24*	*0*
		**Lincosamides**	LmbA	Gamma-glutamyl transferase	2	1	3	5	0
				Lincosamides Total	*2*	*1*	*3*	*5*	*0*
		**Penicillin**	PcbC	Isopenicillin N synthase (IPNS)	41	110	151	263	0
				Penicillin Total	*41*	*110*	*151*	*263*	*0*
		**Phenazine**	PhlD	Putative PhlD	3	5	8	13	0
			PhzA	Isochorismatase	14	18	32	50	0
			PhzC	Phenazine biosynthesis protein PhzC	1	1	2	3	0
			PhzF	PhzF family phenazine biosynthesis protein	18	202	220	448	0
				Phenazine Total	*36*	*226*	*262*	*514*	*0*
		**Pyoluteorin**	PltC	Polyketide synthase type I	0	1	1	3	0
				Pyoluteorin Total	*0*	*1*	*1*	*3*	*0*
		**Pyrrolnitrin**	PrnB	PrnB	1	0	1	1	0
			PrnD	Aminopyrrolnitrin oxidase	3	2	5	9	0
				Pyrrolnitrin Total	*4*	*2*	*6*	*10*	*0*
		**Streptomycin**	StrR	Streptomycin biosynthesis operon regulator	2	2	4	6	0
				Streptomycin Total	*2*	*2*	*4*	*6*	*0*
		Antimicrobial Biosynthesis Total			***93***	***407***	***500***	***943***	***0***
	**CRISPR**		Cas1	CRISPR-associated protein Cas1	294	583	877	1503	0
			Cas10_Crm2	CRISPR-associated protein, Crm2 family	12	160	172	341	0
			Cas10d	CRISPR-associated protein Csc3	1	23	24	47	0
			Cas2	CRISPR-associated protein Cas2	79	412	491	973	0
			Cas2_Ie	CRISPR-associated protein Cas2	14	151	165	349	0
			Cas3	CRISPR-associated helicase Cas3, Anaes-subtype	261	563	824	1504	0
			Cas4	Conserved hypothetical protein	30	361	391	809	0
			Cas5	Fruiting body developmental protein S-like protein	55	410	465	931	0
			Cas6	CRISPR-associated protein Cas6	108	303	411	714	0
			Cas6e	CRISPR-associated protein	24	136	160	324	0
			Cas6f	CRISPR-associated protein, Csy4 family	13	67	80	176	0
			Cas7	CRISPR-associated protein, Csd2 family	96	413	509	959	0
			Cas8a1	Hypothetical protein lse_0420	2	71	73	154	0
			Cas8a2	Hypothetical protein, conserved	1	12	13	25	0
			Cas8b	Conserved hypothetical protein	7	82	89	184	0
			Cas8c	CRISPR-associated protein, Csd1 family	26	191	217	441	0
			Cas9_Csn1	CRISPR-associated protein, Csn1 family	25	130	155	311	0
			Cmr1	CRISPR-associated RAMP protein, Cmr1 family	4	87	91	181	0
			Cmr3	DNA repair (RAMP superfamily) family protein	10	114	124	243	0
			Cmr4	CRISPR-associated RAMP protein, Cmr4 family	8	113	121	240	0
			Cmr5	CRISPR-associated protein, Cmr5 family	6	78	84	163	0
			Cmr6	Predicted coding region AF_1861	11	97	108	207	0
			Csa5	CRISPR-associated protein, Csa5 family	0	18	18	37	0
			CsaX	Hypothetical protein ST2641	0	5	5	10	0
			Csb1	Hypothetical protein AZL_027380	8	41	49	90	0
			Csb2	CRISPR-associated protein, GSU0054 family	10	31	41	72	0
			Csb3	NAD-dependent aldehyde dehydrogenase	0	8	8	18	0
			Csc1	Hypothetical protein LD85_0990	3	28	31	61	0
			Csc2	CRISPR-associated protein Csc2	3	30	33	63	0

			Cse1	CRISPR-associated protein, Cse1 family	20	194	214	458	0
			Cse2	CRISPR-associated protein, Cse2 family	13	169	182	396	0
			Csf1	Hypothetical protein TK90_2701	1	6	7	13	0
			Csf2	Hypothetical protein p1B375	0	12	12	26	0
			Csf3	CRISPR-associated protein, Csf3 family	0	6	6	12	0
			Csf4	DEAD/DEAH box helicase-like	0	4	4	8	0
			Csm2	CRISPR-associated protein, Csm2 family	5	85	90	180	0
			Csm3	CRISPR-associated RAMP protein, Csm3 family	10	108	118	234	0
			Csm4	CRISPR-associated Csm4 family protein	8	76	84	162	0
			Csm5	CRISPR-associated RAMP protein, Csm5 family	6	88	94	189	0
			Csm6	Hypothetical protein HMPREF9333_00225	9	100	109	220	0
			Csx1	CRISPR-associated protein DxTHG motif protein	9	180	189	382	0
			Csx10	Csx10 family CRISPR-associated RAMP protein	0	10	10	20	0
			Csx14	Hypothetical protein GobsU_11505	1	6	7	13	0
			Csx15	Hypothetical protein Cpha266_2052	0	17	17	34	0
			Csx16	Hypothetical protein VVA1548	2	21	23	46	0
			Csx17	Hypothetical protein Btus_2683	6	12	18	30	0
			Csx3	CRISPR-associated Csx3 family protein	1	26	27	53	0
			Csy1	CRISPR-associated protein, Csy1 family	9	57	66	142	0
			Csy2	Hypothetical protein HMPREF0178_02595	8	56	64	126	0
			Csy3	CRISPR-associated protein, Csy3 family	9	68	77	192	0
		CRISPR Total			***1228***	***6019***	***7247***	***14066***	***0***
	**Environmental Toxins**	**Microcystin/Nodularin**	McyB	Non-ribosomal peptide synthetase module, adenylation domain (McyB)	2	2	4	7	0
			NdaA_mcyA	Non-ribosomal peptide synthetase module, adenylation domain (McyA)	3	2	5	12	0
				Microcystin/Nodularin Total	*5*	*4*	*9*	*19*	*0*
		**Saxitoxin**	Saxitoxin_sxtA	Polyketide synthase-related protein	2	1	3	54	0
			SxtH_T_Diox	Phenylpropionate dioxygenase	1	3	4	10	0
				Saxitoxin Total	*3*	*4*	*7*	*64*	*0*
		Environmental Toxins Total			***8***	***8***	***16***	***83***	***0***
	Microbial Defense Total				***3284***	***19954***	***23238***	***50070***	***683***
**Virulence**	**Adherence**		Aae	Autotransporter/adhesin Aae	0	1	1	2	0
			Aap	Dispersin	0	1	1	2	0
			AatA	AatA outermembrane protein	0	1	1	4	0
			AatC	AatC, ATP binding protein of ABC transporter	0	2	2	4	0
			AatD	AatD, apolipoprotein N-acyltransferase	0	3	3	6	0
			AcfA	Accessory colonization factor AcfA	0	7	7	20	0
			AcfB	Accessory colonization factor AcfB	0	1	1	2	0
			AcfD	Accessory colonization factor AcfD precursor	1	9	10	26	0
			AggA	Aggregative adherence fimbrial subunit AggA	1	0	1	1	0
			AggR	Transcriptional activator AggR (AAF/I regulatory protein)	0	1	1	2	0
			AidA	Adhesin AidA (adhesin-involved-in-diffuse-adherence)	1	2	3	5	0
			Ami	Autolysin; amidase	0	1	1	2	0
			Asa	Aggregation substance Asa1	0	1	1	2	0
			Bab	Adhesin-binding fucosylated histo-blood group antigen	3	2	5	7	0
			BabB	Adhesin-binding fucosylated histo-blood group antigen	2	0	2	2	0
			Bad	Surface protein/Bartonella adhesin	0	6	6	16	0
			BfpA	Prepilin-type domain protein	0	6	6	12	0
			BfpB	Outer membrane lipoprotein BfpB	0	2	2	6	0
			BfpC	Bundle forming pilus C	0	1	1	4	0
			BfpD	Bundle forming pilus D	0	2	2	6	0
			BfpF	Bundle forming pilus F	0	1	1	4	0
			BfpL	Inner membrane protein BfpL	0	1	1	2	0
			BfpU	Putative BfpU	0	1	1	4	0
			BfpW	Putative protein perC (Protein bfpW) (fragment)	0	1	1	2	0
			CblA	Cable Pili major subunit	0	1	1	2	0
			CbpD	Choline binding protein D	1	1	2	3	0
			CbpE	Choline binding protein E	2	1	3	4	0
			CbpF	Choline binding protein CbpF	0	1	1	2	0
			Clf	Clumping factor A	0	1	1	2	0
			Cmu	Hypothetical protein, conserved	0	1	1	2	0
			Colonization_factor	Accessory colonization factor AcfA	1	10	11	25	0
			CooA	Adhesin major subunit pilin	1	0	1	1	0
			Cpa135	A135 protein	2	1	3	4	0
			CpsN	Putative LPS biosynthesis related glycosyltransferase	0	1	1	2	0
			CsfA	CS5 fimbrial minor pilin subunit	1	2	3	7	0
			CssC	Chaperone protein CssC	0	2	2	18	0
			Dg	Delta giardin	1	0	1	1	0
			Eae	Intimin	2	6	8	26	0
			EbpS	Cell surface elastin binding protein EbpS	0	1	1	2	0
			Efa1	Putative cytotoxin	0	1	1	6	0
			EfaA	Endocarditis specific antigen	0	1	1	2	0
			EmaA	Extracellular matrix protein adhesin A	2	4	6	10	0
			Esp	Putative enterococcal surface protein	1	0	1	1	0
			Fap1	PT repeat family protein	1	0	1	1	0
			FbsA	Fruit-body specific gene A	1	0	1	1	0
			Fimbriae	K88 fimbrial protein AC precursor	3	5	8	50	0
			FimF	Fimbrial subunit FimF	0	3	3	6	0
			FimG	Putative protein FimG	0	10	10	20	0
			FimH	Fimbrial adhesin FimH	0	8	8	18	0
			FimU	Type 4 fimbrial biogenesis protein (FimU)	0	5	5	12	0
			FocG	f1C Minor fimbrial subunit protein G presursor	1	0	1	1	0
			GapA	Cytadhesin protein GapA	1	0	1	1	0
			Gasp180	Axoneme-associated protein GASP-180	9	2	11	11	0
			Gg	Gamma giardin	1	0	1	1	0
			Gp60	60 kDa Glycoprotein	2	4	6	19	0
			HifA	Major fimbria subunit	1	3	4	8	0
			HifB	Chaperone protein HifB	0	4	4	10	0
			HifC	Outer membrane usher protein hifC precursor	0	2	2	4	0
			HifE	Minor fimbrial subunit hifE/Minor pilin tip adhesin	0	5	5	28	0
			Hmw	HMW1A, high molecular weight adhesin 1	0	3	3	6	0
			Hmw1C	HMW1C, putative glycosyltransferase involved in glycosylation of HMW1A and HMW2A	0	1	1	2	0
			Hmw2A	HMW2A, high molecular weight adhesin 2	0	2	2	4	0
			HopZ	Helicobacter outer membrane protein	0	1	1	2	0
			IcaC	Intercellular adhesion protein C/Biofilm PIA synthesis protein icaC	1	2	3	5	0
			IcaD	Intercellular adhesion protein D	0	1	1	2	0
			IcaR	Intercellular adhesion regulator	0	2	2	4	0
			JlpA	Jejuni lipoprotein	0	1	1	2	0
			Lectin170	Galactose-specific adhesin	6	1	7	8	0
			Lgl3	Galactose-inhibitable lectin 35 kDa subunit precursor, putative	5	1	6	6	0
			LngA	Longus pilus structural subunit	1	1	2	12	0
			LpfA	Putative major fimbrial subunit	2	3	5	8	0
			LpfB	Putative fimbrial chaperone protein	0	6	6	12	0
			LpfC	Outer membrane usher protein lpfC	2	10	12	22	0
			LpfD	Predicted fimbrial protein precursor	0	3	3	11	0
			LpfE	Predicted fimbrial protein precursor	0	2	2	4	0
			Mic1	Microneme 1-like protein	0	1	1	8	0
			Mst101	Axoneme-associated protein mst101, putative	0	2	2	4	0
			NanA	N-Acetylneuraminate lyase	2	3	5	8	0

		NanB	Sialidase B/Neuraminidase B	0	1	1	2	0
		Oap	Opacity associated protein OapA	0	5	5	24	0
		OmpA	Outer membrane protein P5	0	4	4	8	0
		OpcA	Outer membrane protein OpcA	1	4	5	41	0
		OspA	Outer surface protein A	5	4	9	25	0
		OspE1	Outer surface protein OspE1	0	1	1	2	0
		P1	MgPa adhesin	4	2	6	8	0
		Pap	Autotransporter adhesin	3	41	44	118	0
		PapA	Major pilin protein PapA/Mating-type-like protein A1/MTLa1 protein	2	3	5	18	0
		PapB	Major pilus subunit operon regulatory protein PapB	2	14	16	43	0
		PapF	P pilus minor tip component PapF	0	1	1	2	0
		PapG	P pilus adhesin PapG protein	1	2	3	9	0
		PapH	Minor pilin protein PapH	0	1	1	2	0
		Pe35	PE family protein, PE35	0	1	1	2	0
		Peb1C	ABC transporter ATP-binding protein PEB1C	0	2	2	4	0
		Peb2	Major antigenic peptide PEB2	0	1	1	2	0
		Peb3	Major antigenic peptide PEB3	1	1	2	3	0
		Peb4	Major antigenic peptide/PpiC-type peptidyl-prolyl cis-trans isomerase	0	1	1	2	0
		PefA	Major fimbrial antigen subunit pefA	0	1	1	2	0
		PefB	Adhesin biosynthesis transcription regulatory protein	0	1	1	10	0
		PefC	Fimbrial outer membrane usher protein PefC	1	2	3	5	0
		Pertactin	Pertactin family virulence factor/autotransporter	0	6	6	12	0
		PfEMP3	Erythrocyte membrane protein PFEMP3, putative	3	1	4	5	0
		Phist	Phist protein (Pf-fam-b)	15	9	24	31	0
		PilC1	Type IV pilus-associated protein PilC1	1	0	1	1	0
		PilC2	Type IV pilus-associated protein PilC2	0	1	1	9	0
		Pilin	MSHA pilin protein MshA	39	484	523	1076	0
		PilY2	Type 4 fimbrial biogenesis protein PilY2	0	1	1	2	0
		PrgB	Aggregation substance PrgB	0	1	1	2	0
		Prt	UDP-glucose 4-epimerase	1	3	4	7	0
		PsaB	Chaperone protein PsaB	0	1	1	2	0
		PsaC	Outer membrane usher protein psac	0	12	12	24	0
		RatB	Putative outer membrane protein	0	4	4	11	0
		RcpB	Rough colony protein B	0	3	3	6	0
		RcpC	Flp pilus assembly protein RcpC/CpaB	0	2	2	4	0
		Rhamm	Hyaluronan mediated motility receptor	0	1	1	2	0
		Rif	RIFIN	153	18	171	184	0
		RrgB	Pilus subunit RrgB	0	3	3	8	0
		Sab	Outer membrane protein	0	1	1	2	0
		SagF	Streptolysin S biosynthesis protein SagF	1	0	1	1	0
		SagH	Streptolysin S export transmembrane protein/ATP-binding/permease protein SagH	1	0	1	1	0
		SagI	Streptolysin S export transmembrane protein SagI	1	0	1	1	0
		Salp1	SALP-1	1	0	1	1	0
		ScpB	C5a peptidase precursor ScpZ	3	13	16	29	0
		SdeA	SdeA protein	0	2	2	6	0
		SdrD	Ser-Asp rich fibrinogen/bone sialoprotein-binding protein SdrD	0	1	1	2	0
		SfaA	S-fimbrial protein subunit precursor	0	1	1	4	0
		ShdA	AIDA autotransporter-like protein ShdA	0	2	2	9	0
		Srt	Sortase	8	118	126	373	0
		SrtC2	Sortase SrtC2	0	1	1	2	0
		TadV	Prepilin peptidase/Type IV leader peptidase family/Peptidase A24	1	4	5	11	0
		TadZ	Flp pilus assembly protein, ATPase CpaE	2	13	15	29	0
		Tcp	Toxin co-regulated pilin	2	0	2	2	0
		TcpF	Toxin co-regulated pilus biosynthesis protein F	2	1	3	4	0
		TcpQ	Toxin coregulated pilus biosynthesis protein TcpY/TcpQ	0	1	1	2	0
		TopJ	Terminal organelle assembly protein TopJ	0	1	1	6	0
		Tsp	CpTSP6 extracellular membrane-associated protein	0	1	1	2	0
		UspA1	Ubiquitous surface protein A1 UspA1	15	7	22	36	0
		VompA	Surface protein/Bartonella adhesin	0	2	2	4	0
		YagW	YagW	0	1	1	2	0
		YkgK	Putative HTH-type transcriptional regulator YkgK	0	1	1	2	0
	Adherence Total			***330***	***999***	***1329***	***2833***	***0***
**Invasion**		ActA	Actin-assembly inducing protein	3	0	3	3	0
		Adp	Alanyl dipeptidyl peptidase	4	2	6	8	0
		Ag1	Ag-1 blood stage membrane protein	2	0	2	2	0
		Ail	Attachment invasion locus protein	1	9	10	21	0
		Ama1	Apical membrane antigen 1	10	12	22	405	0
		Bca	C protein alpha-antigen	0	2	2	4	0
		Cia	Invasion antigen B	2	4	6	10	0
		CloSI	Alpha-clostripain	0	1	1	2	0
		Enh	Enhanced entry protein EnhA	0	13	13	35	0
		EnhB	Hypothetical protein, weakly similar to enhanced entry protein EnhB	0	2	2	6	0
		EnhC	Enhanced entry protein EnhC	0	4	4	10	0
		EspP	Predicted serine protease EspP	0	1	1	2	0
		HysA	Polysaccharide lyase family 8, super-sandwich domain protein	0	1	1	2	0
		IbeC	Membrane protein YijP	0	2	2	24	0
		Icp2	Amoebiasin 2 precursor	0	1	1	2	0
		IcsP	Protease VII	4	0	4	4	0
		IgA1	IgA1 protease	7	22	29	62	0
		InlE	Internalin E	1	0	1	1	0
		Inv	Invasion protein	5	22	27	71	0
		InvF	Invasion regulatory protein	0	3	3	6	0
		InvG	Outer membrane invasion protein/Secretory protein	0	3	3	6	0
		InvH	Cell adherance/invasion protein	0	1	1	2	0
		Las	LasA protease precursor	0	6	6	14	0
		LasB	Elastase LasB	0	2	2	4	0
		Mf	Mitogenic factor - phage associated	0	1	1	2	0
		Mpl	Zinc metalloproteinase (elastase)	1	0	1	1	0
		Msp1	Merozoite surface protein 1	14	14	28	529	0
		Nag	Hyaluronidase	0	1	1	2	0
		NanI	Exo-alpha-sialidase	0	1	1	2	0
		NanJ	Hypothetical protein CLONEX_02961	0	1	1	2	0
		OrgA	Oxygen-regulated invasion protein; cell invasion protein	0	1	1	2	0
		PlcA	Phosphatidylinositol-specific phospholipase C	1	0	1	1	0
		PlcB	Zinc dependent phospholipase C	2	0	2	2	0
		Ptp1	Polar tube protein Ptp1	1	0	1	1	0
		Ptp2	Polar tube protein 2	2	3	5	8	0
		Ptp3	Polar tube protein 3	1	1	2	3	0
		Rama	Rhoptry associated membrane antigen	2	0	2	2	0
		Rap1	Rhoptry associated protein 1	1	2	3	5	0
		Ron2	Rhoptry neck protein 2	1	1	2	3	0
		Rop16	Rhoptry protein 16	0	1	1	4	0
		Rop5	Rhoptry protein 5B	0	1	1	5	0
		Sak	Plasminogen activator	1	0	1	1	0
		Sda	Deoxyribonuclease precursor	0	3	3	13	0
		Ska	Streptokinase	2	0	2	2	0
		StcE	Metalloprotease StcE	0	1	1	2	0
		Tsh	Temperature-sensitive hemagglutinin tsh	1	3	4	7	0

		ZmpA	ZmpA -like peptidase, Metallo peptidase, MEROPS family M04	0	1	1	2	0
	Invasion Total			***69***	***149***	***218***	***1307***	***0***
**Cellular components**	**Cell wall**	ExsA	Spore coat assembly protein exsA	0	3	3	10	0
		FadD28	Acyl-CoA synthetase	1	1	2	3	0
		MmaA1	Methoxy mycolic acid synthase 1	2	1	3	4	0
		MmaA2	Methoxy mycolic acid synthase 2 MmaA2 _1	1	1	2	3	0
		MmaA3	Methoxy mycolic acid synthase 3, MmaA3	1	0	1	1	0
		OmpD	Outer membrane porin protein ompD	3	4	7	11	0
		Swp1	Spore wall protein 1	1	0	1	1	0
			Cell Wall Total	*9*	*10*	*19*	*33*	*0*
	**Cyst wall**	Art	Articulin family protein	1	1	2	3	0
		Cowp	Oocyst wall protein	5	8	13	22	0
		Cwp1	Cyst wall protein 1	1	0	1	1	0
		Cwp2	Cyst wall protein 3	4	0	4	4	0
		Jacob	Cyst wall-specific glycoprotein Jacob family protein	2	7	9	17	0
			Cyst Wall Total	*13*	*16*	*29*	*47*	*0*
	**Spore**	Hswp	Hypothetical spore wall protein	6	0	6	6	0
			Spore Total	*6*	*0*	*6*	*6*	*0*
	Cellular Components Total			***28***	***26***	***54***	***86***	***0***
**Cellular Function**	**Mitosis**	Cald	Caldesmon, putative	4	5	9	14	0
			Mitosis Total	*4*	*5*	*9*	*14*	*0*
	**Cellular metabolism**	Ben_bcla	Benzoate-CoA ligase family	44	192	236	422	158
		LgtA	Lacto-N-neotetraose biosynthesis glycosyl transferase LgtA	0	4	4	8	0
		nap	Neutrophil activating protein	0	13	13	26	0
		NarX	Nitrate reductase narX	1	0	1	1	0
			Cellular Metabolism Total	*45*	*209*	*254*	*457*	*158*
	**Nutrient acquisition**	Ccm	Putative Redoxin family protein	0	4	4	12	0
		ChuA	Outer membrane hemin receptor ChuA	1	7	8	15	0
		ChuS	Putative heme/hemoglobin transport protein	0	3	3	6	0
		ChuT	Putative periplasmic binding protein (putative hemin binding protein)	0	2	2	4	0
		ChuU	Putative iron compound ABC transporter, permease protein	1	0	1	1	0
		ChuW	Coproporphyrinogen III oxidase	1	5	6	13	0
		Feo	Ferrous iron transporter B	0	4	4	8	0
		FptA	Fe(III)-pyochelin receptor precursor	1	1	2	3	0
		FreB	FreB protein	4	5	9	13	0
		FrgA	Putative siderophore biosynthesis protein	0	1	1	2	0
		Hgp	Hemoglobin binding protein A	0	1	1	2	0
		Hhu	Hemoglobin and hemoglobin-haptoglobin binding protein	3	9	12	23	0
		HxuA	Heme-hemopexin utilization protein A	0	4	4	8	0
		HxuB	Heme/hemopexin-binding protein B, hemolysin activation/secretion protein	0	4	4	12	0
		HxuC	heme-hemopexin utilization protein C	0	8	8	21	0
		Icl	Isocitrate lyase icl	1	0	1	1	0
		Ira	Small-molecule methyltransferase IraA	0	2	2	4	0
		IrgB	Iron-regulated virulence regulatory protein IrgB	0	5	5	16	0
		Iro	Outer membrane ferric siderophore receptor	40	382	422	858	0
		IroC	ABC transporter, ATP-binding protein	1	0	1	1	0
		IsdA	Iron (Fe2+)-regulated surface determinant protein IsdA	0	1	1	2	0
		IsdC	Iron transport associated protein	1	6	7	29	0
		Iuc	Aerobactin biosynthesis protein	3	60	63	156	0
		IutA	Aerobactin siderophore receptor iutA	1	16	17	37	0
		MbtA	Putative BIFUNCTIONAL ENZYME MBTA: SALICYL-AMP LIGASE (SAL-AMP LIGASE) + SALICYL-S-ArCP SYNTHETASE	1	1	2	3	0
		MbtB	Phenyloxazoline synthase MbtB	1	0	1	1	0
		MbtD	Polyketide synthetase mbtd	0	1	1	2	0
		MbtE	Non-ribosomal peptide synthetase MbtE	1	3	4	7	0
		MbtF	Mycobactin biosynthesis peptide synthetase MbtF	1	2	3	5	0
		MbtG	Lysine-N-oxygenase MbtG	1	0	1	1	0
		Mce3	Mce family protein	2	4	6	10	0
		MirB	Siderophore iron transporter mirB	21	9	30	37	0
		NpgA	4'-Phosphopantetheinyl transferase NpgA	4	7	11	18	0
		OrbA	Ornibactin receptor OrbA	0	1	1	2	0
		SreA	Siderophore transcription factor SreA	5	1	6	7	0
		TbpA	Probable transferrin binding protein 1 precursor	1	0	1	1	0
			Nutrient Acquisition Total	*96*	*559*	*655*	*1341*	*0*
	**Regulation**	Arid1	ARID1 AT-rich interaction domain protein	1	0	1	1	0
		Arid2	ARID2 AT-rich interaction domain protein	1	0	1	1	0
		Bos1	Two-component osmosensing histidine kinase (Bos1)	1	3	4	7	0
		BvrR	Two component transcriptional regulator, winged helix family	0	3	3	6	0
		CovR	Response regulator protein	0	1	1	2	0
		CovS	Sensor histidine kinase	0	1	1	2	0
		CqsS	Cholerae quorum sensing sensor	0	8	8	18	0
		DevR	Two component transcriptional regulatory protein devr	0	1	1	2	0
		DevS	Two component sensor histidine kinase DevS	1	1	2	3	0
		Glp	GARP-like protein 3	7	1	8	10	0
		GrlA	Putative LEE-encoded positive regulator of transcription	0	1	1	2	0
		HapB	CCAAT-binding transcription factor subunit HAPB	2	5	7	12	0
		HapC	CCAAT-binding factor complex subunit HapC	4	0	4	4	0
		HapX	bZIP transcription factor HapX	6	9	15	26	0
		Hfq	RNA-binding protein Hfq	9	43	52	101	0
		LasR	Transcriptional activator protein LasR	0	2	2	4	0
		Ler	Ler DNA-binding protein H-NS	0	1	1	2	0
		LetS	Legionella transmission sensor LetS	0	2	2	4	0
		Mod5	Mod5 protein-sorting protein	0	5	5	12	0
		MprA	Two component transcriptional regulator, winged helix family	0	1	1	2	0
		PhcA	PhcA	2	3	5	14	0
		Rpn2p	19S/PA700 proteasome regulatory particle subunit Rpn2p/S2	0	7	7	44	0
		RsaL	Virulence gene repressor RsaL	4	0	4	4	0
		SaeR	Response regulator SaeR	1	18	19	48	0
		SarA	Transcriptional regulator SarA	2	3	5	9	0
		VirF	Virulence regulon transcriptional activator VirF	1	2	3	5	0
			Regulation Total	*42*	*121*	*163*	*345*	*0*
	**Quorum sensing**	HapR	Quorum-sensing regulator of virulence HapR	0	4	4	8	0
			Quorum Sensing Total	*0*	*4*	*4*	*8*	*0*
	Cellular Function Total			***187***	***898***	***1085***	***2165***	***158***
**Immune evasion**	**Antiphagocytosis**	Alg	Type IV pilus assembly PilZ	2	7	9	18	0
		AlgB	Two component, sigma54 specific, Fis family transcriptional regulator	1	9	10	26	0
		AlgE	Alginate biosynthesis protein AlgE	0	6	6	18	0
		AlgF	Alginate O-acetyltransferase	0	4	4	8	0
		AlgG	Alginate-C5-mannuronan-epimerase AlgG	0	3	3	6	0
		AlgK	Alginate biosynthesis protein AlgK	2	7	9	18	0
		AlgX	Alginate biosynthesis protein AlgX	1	4	5	11	0
		Aur	Aureolysin	0	3	3	6	0
		Caf1R	F1 operon positive regulatory protein	0	1	1	2	0
		Cap	Capsule polysaccharide modification protein LipA	23	182	205	410	0
		Cap5A	Putative capsular polysaccharide synthesis enzyme Cap5A	0	2	2	4	0
		Cap8B	Type 8 capsule gene Cap8B	0	1	1	2	0
		Cap8E	Capsular polysaccharide synthesis enzyme Cap8E	0	1	1	2	0
		Cap8M	Sugar transferase	0	2	2	4	0
		CtrA	Capsule polysaccharide export outer membrane protein CtrA	0	2	2	4	0

		CtrB	Polysialic acid capsule export inner-membrane protein CtrB	0	3	3	14	0
		CtrC	Putative capsule polysaccharide export inner-membrane protein ctrC	0	5	5	10	0
		Lip	Manganese peroxidase 5	28	15	43	63	0
		LipB	Capsule polysaccharide modification protein LipB	0	2	2	6	0
		Mimp	24 kDa macrophage-induced major protein	0	2	2	5	0
		SiaB	Capsule biosynthesis protein SiaB	0	1	1	2	0
		SiaC	Sialic acid synthase	0	3	3	6	0
		SiaD	Sialyltransferase	0	1	1	2	0
			Antiphagocytosis Total	*57*	*266*	*323*	*647*	*0*
	**Intracellular survival**	Cgs	Enhanced intracellular survival protein	1	0	1	1	0
		IglA	Intracellular growth locus, subunit A, putative	0	1	1	2	0
		IglB	Intracellular growth locus, subunit B	0	1	1	2	0
		PdpD	Pathogenicity determinant protein D	0	1	1	2	0
			Intracellular Survival Total	*1*	*3*	*4*	*7*	*0*
	**Macrophage invasion**	MmpL7	MMPL domain protein	0	1	1	2	0
			Macrophage Invasion Total	*0*	*1*	*1*	*2*	*0*
	**Serum resistance**	Asc1	Hypothetical protein BATDEDRAFT_11488	3	0	3	3	0
		Brk	BrkB; serum resistance protein	1	0	1	1	0
		Dsr	Serum resistance protein A2	0	2	2	10	0
			Serum Resistance Total	*4*	*2*	*6*	*14*	*0*
	**Other**	AmsG	UDP-galactose-lipid carrier transferase	0	3	3	6	0
		Apsp	Apicomplexan protein	2	0	2	2	0
		Bg	Beta-giardin	1	0	1	1	0
		BptA	Protein BptA (Borrelial persistence in ticks protein A)	0	1	1	5	0
		Geh	Lipase (Glycerol ester hydrolase)	0	4	4	8	0
		LmaA	Antigen A	0	1	1	2	0
		LmaB	Antigen B	0	1	1	2	0
		LmaC	Antigen C	0	2	2	4	0
		LmaD	Antigen D	0	2	2	4	0
		OmpP2	Outer membrane protein P2 precursor (OMP P2)	3	7	10	18	0
		TviC	VI Polysaccharide biosynthesis protein VipB/TviC family protein	0	3	3	10	0
		TviE	VI Polysaccharide biosynthesis protein VipC/tviE	0	1	1	2	0
		VexA	VI Biosynthesis protein VexA	1	0	1	1	0
		VexC	VI Biosynthesis protein VexC	1	0	1	1	0
		VexE	VI Biosynthesis protein VexE	1	1	2	3	0
			Other Immune Evasion Total	*9*	*26*	*35*	*69*	*0*
	Immune Evasion Total			***71***	***298***	***369***	***739***	***0***
**Marker**		Asp	Adaptor protein complex large chain subunit BetaA	0	1	1	2	0
		BetaA	Adaptor protein complex large chain subunit BetaA	1	0	1	1	0
		CatL	Cathepsin L precursor	6	4	10	14	0
		Cdkl	Kinase, CMGC CDKL	1	1	2	3	0
		Cf1A	Cleavage and polyadenylation factor CF-IA component RNA15	0	1	1	8	0
		Cp15_60	Sporozoite surface antigen CP15/60	0	2	2	4	0
		Crp	CXC-rich protein	4	2	6	8	0
		Dlp	Dynein-like protein	1	1	2	3	0
		Dph2	Diphthamide synthase subunit DPH2	1	5	6	17	0
		EEF2	Peptide elongation factor 2	4	1	5	5	0
		Egf	High cysteine membrane EGF-like protein	10	14	24	27	0
		Fdnr	Nitroreductase family protein fused to ferredoxin domain Fd-NR1	3	2	5	7	0
		FprA	A-type flavoprotein lateral transfer candidate	6	0	6	6	0
		Ftl	Putative fucose translocatorw ith 8 transmembrane domains, within locus of 3 paralogous genes	1	5	6	10	0
		Gccap	Golgi/cell cycle associated protein, putative	1	1	2	3	0
		Gp49	Invariant antigen Gp49	1	0	1	1	0
		Gta2	Giardia trophozoite antigen GTA-2	1	0	1	1	0
		HCNCp	High cysteine membrane protein Group 6	98	101	199	247	0
		Hpsptmd	Hypothetical protein having a signal peptide and transmembrane domain near C-terminus	4	0	4	4	0
		IihP34	TFIIH P34	1	1	2	3	0
		InterB	Putative interB family protein, partial	0	3	3	9	0
		Ire	IRE protein kinase	5	2	7	8	0
		Klp5	E04F6.2 like protein	2	1	3	5	0
		Lrrp1	Leucine-rich repeat protein 1 virus receptor protein	1	0	1	1	0
		Mbp	Median body protein	1	0	1	1	0
		Mdrlp	MDR protein-like protein	1	0	1	1	0
		Myb1	Myb 1-like protein	3	0	3	3	0
		Nefm	Neurofilament medium polypeptide, putative	1	0	1	1	0
		Np1	Antigenic protein NP1/Non-pathogenic protein 1	0	2	2	4	0
		Nuf1	Spindle pole body component 110	1	0	1	1	0
		P115	P115, putative	1	1	2	3	0
		P40	Translation initiation factor IF-3 subunit 3	0	1	1	2	0
		Phkg	Phosphorylase B kinase gamma catalytic chain	3	2	5	7	0
		PirG	Exported repetitive protein precursor PirG (cell surface protein) (EXP53)	0	1	1	2	0
		Pmm2	Phosphomannomutase-2	3	1	4	5	0
		S1p1	S1/P1nuclease, putative	4	2	6	7	0
		Sec6	Exocyst complex subunit Sec6	1	5	6	11	0
		Smcp	Set and mynd domain containing protein, putative	1	0	1	1	0
		Spgpi	Signal peptide plus GPI anchored membrane protein	0	1	1	2	0
		Spp	Spindle pole protein, putative	3	3	6	9	0
		Spptmd	Hypothetical protein, conserved	0	1	1	2	0
		Spspr	Signal peptide, serine proline rich, possible low mw mucin glycoprotein locus of 6 genes	0	1	1	2	0
		Srap	Serine-rich 25 kDa antigen protein, putative	0	1	1	2	0
		Stu2	STU2-like protein	1	1	2	3	0
		Sycp	Synaptonemal complex protein, putative	4	3	7	10	0
		Tep	TM efflux prot	1	1	2	3	0
		Tmp52	TMP52	1	0	1	1	0
		Tmp55	TMP55	1	0	1	1	0
		Ubce14	Ubiquitin-conjugating enzyme 14/Ubiquitin-conjugating enzyme E2	1	1	2	3	0
		Uch	Ubiquitin C-terminal hydrolase of the cysteine proteinase fold	0	2	2	4	0
		Upl1	UPL-1	1	0	1	1	0
		VccZ	Vesicle coat complex subunit zeta	0	4	4	8	0
		Xoc	Ximpact ortholog conserved protein seen in bacteria and eukaryotes	0	2	2	4	0
		Yip	Yip	1	0	1	1	0
		Yip1	Yip interacting protein, putative	1	1	2	3	0
		Yip2	Yip interacting protein, putative	1	1	2	3	0
	Marker Total			***188***	***186***	***374***	***508***	***0***
**Soil-borne pathogens**		AcsC	Achromobactin biosynthetic protein AcsC	1	3	4	13	0
		AcsD	Achromobactin biosynthetic protein AcsD	0	5	5	15	0
		Arp1	Adhesin protein arp1	1	1	2	3	0
		AvrA	Type III effector protein AvrA	0	1	1	2	0
		AvrBs1	Avirulence protein AvrBs1	0	1	1	2	0
		AvrBs2	Avirulence protein avrBs2	0	1	1	2	0
		AvrBs3	AvrBs3 family type III effector protein	1	1	2	3	0
		CelA	Cellulase CelA	0	2	2	6	0
		Cyanide_hydratase_Fungi	Conserved hypothetical protein	49	21	70	75	0
		DspE	Avirulence protein DspE	1	2	3	5	0
		Enniatin_synthase_Fungi	Nonribosomal peptide synthase, putative	7	5	12	14	0
		EsaT6	6 kDa Early secretory antigenic target EsaT6 (EsaT-6)	0	1	1	2	0
		Evr	Evr regulated gene A (ErgA)	2	1	3	4	0
		ExoY	Adenylate cyclase ExoY	0	1	1	2	0

		FyuA	Pesticin receptor	1	6	7	14	0
		HasA	Hemophore HasA	0	9	9	18	0
		HopAF1	Type III effector HopAF1	1	4	5	14	0
		HrcU	Type III secretion system translocation protein HrcU	7	6	13	19	0
		HrpB2	Type III secretion protein HrpB	0	9	9	19	0
		HrpG	Response regulator protein	3	1	4	5	0
		HrpG1	Type III secretion protein HrpG	1	0	1	1	0
		HrpP	Type III secretion protein HrpP	2	1	3	4	0
		HrpX	AraC-Type transcriptional regulator HrpX	0	1	1	2	0
		HrpY2	Response regulator HrpY	1	1	2	3	0
		IgaA	Intracellular growth attenuator IgaA	2	29	31	62	0
		Imp	Immunodominant membrane protein	1	1	2	3	0
		ImpG	ImpG	1	5	6	17	0
		ImpH	ImpH	2	5	7	16	0
		ImpJ	Type VI secretion system protein ImpJ	0	6	6	12	0
		Iss	Lipoprotein	0	1	1	2	0
		MgtB	P-Type 2 magnesium transport ATPase	1	16	17	33	0
		Mip	Macrophage infectivity potentiator	7	4	11	15	0
		PapC	Outer membrane usher protein PapC precursor	12	47	59	108	0
		Pat1	Putative pat-1 homologue	3	1	4	6	0
		Pch	Isochorismate synthase	0	1	1	2	0
		PchB	Isochorismate-pyruvate lyase	0	1	1	2	0
		PchR	Transcriptional regulator PchR	0	1	1	2	0
		PinF1	Cytochrome P450-pinF	1	1	2	4	0
		Psn	Pesticin	2	0	2	2	0
		PtlB	VirB3-like type IV secretion system outer membrane component PtlB	0	1	1	2	0
		Scytalone_dehydratase_Fungi	Scytalone dehydratase	22	3	25	26	0
		Spiralin	Spiralin	3	1	4	6	0
		Tom	Tomatinase	2	0	2	2	0
		TxtA	Thaxtomin synthetase A	1	0	1	1	0
		VirE2	Type IV secretion system single-stranded DNA binding protein VirE2	1	0	1	1	0
		Vmp1	Variable membrane protein VMP1	0	1	1	26	0
		XcpY	Type II secretion system protein L/General secretion pathway protein L	1	0	1	1	0
		XcpZ	Type II secretion system protein M/General secretion pathway protein M	1	0	1	1	0
		XopD	Xanthomonas outer protein D	2	0	2	2	0
		YopD	Type III secretion host injection and negative regulator protein (YopD)	4	1	5	6	0
		YopN	Type III secretion regulator YopN	1	1	2	3	0
		YopT	Cytotoxic effector protein YopT/Cysteine protease YopT	0	3	3	6	0
		YscB	YopN-specific chaperone YscB	2	0	2	2	0
		YscO	Yop proteins translocation protein O	0	1	1	2	0
		YscX	Type III secretion protein YscX	2	3	5	11	0
		YscY	Type III secretion protein YscY	0	1	1	5	0
	Soil-borne pathogens Total			***152***	***219***	***371***	***636***	***0***
**Toxins**		aflatoxin_aflQ	O-methylsterigmatocystin oxidoreductase	23	9	32	33	0
		Amt	Nonribosomal peptide synthetase, putative	6	3	9	14	0
		BinAB	Binary toxin	0	4	4	12	0
		BoNT	Botulinum neurotoxin type F	1	2	3	13	0
		BplB	Acetyltransferase BplB	0	1	1	2	0
		BplC	Lipopolysaccharide biosynthesis protein BplC	0	2	2	4	0
		BplE	Glycosyl transferase BplE	0	2	2	4	0
		BplF	Lipopolysaccharide biosynthesis protein BplF	0	1	1	2	0
		BplG	Sugar transferase BplG	0	2	2	4	0
		BplH	Glycosyl transferase BplH	0	1	1	2	0
		BplL	Lipopolysaccharide biosynthesis protein BplL	0	1	1	2	0
		C3	Mono-ADP-ribosyltransferase C3 (Exoenzyme C3)	0	2	2	4	0
		Cdt	Cytolethal distending toxin A	0	3	3	7	0
		CdtB	Cytolethal distending toxin B-like protein	5	8	13	27	0
		CdtC	Cytolethal distending toxin CdtC	0	3	3	6	0
		Cnf	Cytotoxic necrotizing factor 1	3	7	10	26	0
		Cya	Calmodulin-sensitive adenylate cyclase	0	1	1	2	0
		CyaB	Cyclolysin secretion ATP-binding protein	0	3	3	6	0
		CylA	Cytolysin activator protein CylA	1	0	1	1	0
		Cylc2	Cylicin-2, putative	0	3	3	6	0
		CylI	Cytolysin immunity CylI domain protein	1	5	6	19	0
		CylM	Putative CylM protein	0	3	3	8	0
		Dnt	Dermonecrotic toxin	0	1	1	2	0
		Edin	Epidermal cell differentiation inhibitor	0	2	2	5	0
		EltA	Heat-labile enterotoxin A subunit	3	13	16	29	0
		EltB	Heat-labile enterotoxin B subunit	2	0	2	2	0
		Eta	Exfoliative toxin A	5	9	14	32	0
		Fumonisin_pks	Polyketide synthase	1	0	1	1	0
		Hla	Alpha-hemolysin	0	1	1	2	0
		Hlb	Beta-hemolysin	1	0	1	1	0
		Hly	Hemolysin	15	288	303	650	0
		Ibp	Iota toxin component Ib	0	3	3	7	0
		LukF	Putative leukocidin F subunit	0	2	2	5	0
		NetB	Delta toxin	0	2	2	53	0
		PagA	Protective antigen	1	3	4	8	0
		Pet	Serine protease (plasmid-encoded toxin Pet)	0	1	1	2	0
		Pfp	Pore-forming protein-like protein	4	1	5	6	0
		PlcB1	Membrane-associated phospholipase C 2 PlcB	1	0	1	1	0
		PlcC	Phospholipase C 3 plcC	1	0	1	1	0
		Ply	Alveolysin	1	0	1	1	0
		Pmt	Putative toxin	1	5	6	13	0
		Rtx	RTX toxin RtxA	0	6	6	20	0
		RtxA	Structural toxin protein RtxA	0	2	2	4	0
		Slo	Alveolysin	1	0	1	1	0
		SpvC	Type III effector phosphothreonine lyase SpvC	0	2	2	4	0
		Sta	Heat-stable enterotoxin	2	0	2	2	0
		Stx	Shiga-like toxin type 1 subunit A variant	0	1	1	3	0
		TccC1	Insecticidal toxin complex protein tccc2	1	2	3	5	0
		Tdh	Hemolysin S	0	1	1	2	0
		Toxin	Exfoliative toxin A	2	18	20	41	0
		ToxR	Transmembrane transcriptional activator protein	8	35	43	111	0
		VacA	Vacuolating cytotoxin (VacA) paralogue	4	8	12	24	0
		VvhA	Cytotoxin, cytolysin precursor VvhA	0	4	4	8	0
		VvhB	Cytolysin secretion protein VvhB	0	3	3	6	0
		WbpB	Lipopolysaccharide biosynthesis protein wbpB	0	1	1	2	0
		XaxA	Toxin XaxA	0	2	2	6	0
		XaxB	Toxin XaxB	0	2	2	6	0
		Ymt	Murine toxin	0	4	4	8	0
	Toxins Total			***94***	***488***	***582***	***1278***	***0***
**Secretion Systems**	**Type II secretion system**	AexT	Effector protein AexT	2	0	2	2	0
		BipB	Translocator protein BipB	0	1	1	2	0
		BipC	Type III secretion target BipC	0	1	1	2	0
		BsaK	Type III secretion system protein BsaK	0	1	1	2	0
		EsaV	Type III secretion protein EsvA	0	2	2	7	0
		EscD	Type III secretion system protein EscD	0	1	1	2	0

			EspA	Secreted protein EspA	0	3	3	9	0
			EspD	Secreted protein EspD	1	1	2	4	0
			EspG	Type III secretion system secreted effector EspG	0	1	1	2	0
			EspJ	Type III secretion system secreted effector EspJ	0	1	1	2	0
			EspM	Type III secretion system secreted effector EspM	0	2	2	6	0
			HilA	Invasion genes transcription activator	0	1	1	2	0
			HilD	Transcriptional regulator HilD	0	1	1	2	0
			HrpD	Type III secretion protein HrpD	4	8	12	17	0
			HrpQ	Type III secretion protein RscQab	10	12	22	34	0
			IpaC	Invasion protein SipC	1	2	3	5	0
			IpaD	Invasion plasmid antigen IpaD	1	3	4	7	0
			IpgD	Inositol phosphate phosphatase sopB	1	3	4	7	0
			IpgF	Putative transglycosylase protein	0	1	1	2	0
			LcrD	Low Calcium response protein D	0	2	2	4	0
			LcrE	Low Calcium response protein E	0	1	1	2	0
			LcrF	Thermoregulatory protein LcrF	0	4	4	8	0
			LcrG	Type III secretion cytoplasmic plug protein	1	1	2	4	0
			LcrH	Type III secretion chaperone/low calcium response protein H	0	3	3	29	0
			LspD	Type II secretory pathway protein LspD	0	1	1	4	0
			LspE	Type II protein secretion ATPase LspE	0	1	1	4	0
			LspF	Type II secretory pathway protein LspF	0	1	1	4	0
			LspG	Type II secretory pathway protein LspG	0	1	1	4	0
			LspJ	Type II secretory pathway protein LspJ	0	1	1	4	0
			LspK	type II secretory pathway protein LspK	0	1	1	4	0
			MxiC	Type III secretion regulator YopN/LcrE/InvE/MxiC	0	1	1	2	0
			OspG	Ribosomal protein S6 kinase alpha-5	0	3	3	6	0
			Pkn5	Putative serine/threonine protein kinase	0	2	2	4	0
			PrgJ	Conjugal transfer protein	0	1	1	2	0
			SifA	Secreted effector protein	1	2	3	5	0
			SipA	Pathogenicity island 1 effector protein SipA	1	1	2	3	0
			SlrP	E3 ubiquitin-protein ligase SlrP	0	1	1	2	0
			SopD	Sop effector protein SopD	1	0	1	1	0
			SopE	Guanine nucleotide exchange factor sopE/secreted effector protein SopE	0	1	1	2	0
			SopE2	Invasion-associated type III secreted effector protein	0	1	1	2	0
			Spa29	Surface presentation of antigens protein (Spa29 protein), Type III secretion apparatus	0	1	1	2	0
			SpiC	Pathogenicity island 2 secreted effector protein	0	1	1	2	0
			SprB	Transcriptional regulator SprB	1	0	1	1	0
			SptP	Protein tyrosine phosphate	0	1	1	2	0
			SycE	Type III secretion chaperone SycE	0	3	3	9	0
			SycN	Type III secretion chaperone SycN	2	6	8	27	0
			SycT	YopT-specific chaperone SycT	0	1	1	2	0
			Tir	Translocated intimin receptor protein	1	0	1	1	0
			Type_III_secretion	Type III secretion apparatus protein, YscD/HrpQ family	51	93	144	261	0
			YopB	Transmembrane effector protein YopB/Type III secretion host injection protein (YopB)	0	3	3	6	0
			YopE	Outer membrane virulence protein YopE	1	1	2	3	0
			YopM	Outer membrane protein YopM	0	2	2	4	0
			YopO	Targeted effector protein kinase	0	2	2	4	0
			YopP	Targeted effector protein YopP	0	2	2	4	0
			YscJ	Type III secretion protein SctJ	0	4	4	11	0
			YscL	Putative type III secretion protein SctL	0	1	1	2	0
				Type II Secretion System Total	*80*	*196*	*276*	*557*	*0*
		**Type IV secretion system**	BepA	BepA protein	0	2	2	4	0
			Cag2	Cag pathogenicity island protein	1	0	1	1	0
			Cag5	Cag pathogenicity island protein (cag5)	0	1	1	2	0
			DotB	Defect in organelle trafficking protein DotB (ATPase)	0	2	2	4	0
			DotD	Lipoprotein DotD	0	3	3	8	0
			EsxA	Virulence factor EsxA	0	3	3	6	0
			IcmC	IcmC protein	0	3	3	8	0
			IcmD	IcmD protein	0	6	6	20	0
			IcmQ	IcmQ	23	0	23	23	0
			IcmX	Intracellular multiplication protein IcmX	1	3	4	9	0
			LidA	Coiled coil protein, some similarities with LidA	0	1	1	2	0
			LigA	LigA, interaptin	0	1	1	2	0
			LvgA	Putative virulence protein LvgA	0	1	1	2	0
			MycP1	Peptidase S8 and S53, subtilisin, kexin, sedolisin	0	3	3	6	0
			MycP5	Proline rich membrane-anchored mycosin MycP5	1	0	1	1	0
			RalF	RalF protein, translocated into host cells by the Dot/Icm system	1	1	2	3	0
			Tra	Conjugal transfer entry exclusion protein TraS	1	2	3	5	0
			Trap	Thrombospondin related adhesive protein	2	0	2	2	0
			TraT	Conjugal transfer surface exclusion protein TraT	3	1	4	5	0
			TrwD	P-type DNA transfer ATPase VirB11	1	3	4	8	0
			TrwE	TrwE	2	0	2	2	0
			TrwF	TrwF protein	1	4	5	10	0
			TrwG	TrwG protein	2	4	6	11	0
			TrwI1	TrwI1 protein	1	1	2	4	0
			TrwI2	TrwI2 protein	1	2	3	6	0
			TrwJ1	TrwJ1 protein	1	2	3	6	0
			TrwJ2	TrwJ2 component of type IV secretion system	2	2	4	7	0
			TrwL1	TrwL1 protein	0	4	4	10	0
			TrwL2	TrwL2 protein	0	4	4	10	0
			TrwL3	TrwL3 protein	1	3	4	9	0
			TrwL4	TrwL4 protein	0	2	2	6	0
			TrwL5	TrwL5 protein	1	2	3	5	0
			TrwL7	TrwL7 protein	0	1	1	3	0
			TrwL8	TrwL8 protein	0	1	1	3	0
			VipF	N-terminal acetyltransferase, GNAT family	0	1	1	4	0
			Vir	Type IV secretion system protein VirB4	1	12	13	25	0
				Type IV Secretion System Total	*47*	*81*	*128*	*242*	*0*
		**ESX-1 secretion system**	Cfp10	10 kDa culture filtrate antigen	0	2	2	4	0
				ESX-1 Secretion System Total	*0*	*2*	*2*	*4*	*0*
		**Type VII secretion system**	EssC	Virulence protein EssC	0	5	5	12	0
				Type VII secretion system Total	*0*	*5*	*5*	*12*	*0*
		Secretion Systems Total			***127***	***284***	***411***	***815***	***0***
	**Other**		AVRk1	Avirulence protein AVRk1	3	1	4	5	0
			ChpB	Putative transcriptional regulator methylesterase	1	0	1	1	0
			Csp	Circumsporozoite protein	1	2	3	490	0
			Cyp51	Eburicol 14 alpha-demethylase	3	1	4	7	0
			EsaA	Virulence protein EsaA	0	2	2	4	0
			EsaB	Virulence protein EsaB	0	1	1	2	0
			LcrV	AcrV protein	5	3	8	11	0
			Lipo	Glycosyl transferase, group 1	0	7	7	14	0
			M2ap	MIC2-associated protein M2AP	0	1	1	4	0
			Vip	Adherence and virulence protein A	5	31	36	67	0
		Other Virulence Total			***18***	***49***	***67***	***605***	***0***
	Virulence Total				***1264***	***3596***	***4860***	***10972***	***158***
**Organism Specific Categories**									
**Virus**	**Algal Virus**	**Structural**	Chlorella_vp130	Surface protein vp130	0	5	5	12	0
			NCLDV_capsid_protein	Major capsid protein	18	46	64	133	0

			Algal Structural Total	*18*	*51*	*69*	*145*	*0*
	**Replication**	NCLDV_dna_polymerase	DNA polymerase	39	43	82	223	0
		RdRp_Picornavirales	RNA-dependent RNA polymerase	326	118	444	619	0
			Algal Replication Total	*365*	*161*	*526*	*842*	*0*
	**Infection**	Potassium_channel	Potassium channel protein	0	3	3	6	0
			Algal Infection Total	*0*	*3*	*3*	*6*	*0*
	Algal Virus Total			***383***	***215***	***598***	***993***	***0***
**Soil Borne Virus**	**Structural**	2b_Tobravirus	2b Protein	2	2	4	8	0
		Capsid_vp25_Cheravirus	Coat protein/Capsid protein/CP	1	1	2	3	0
		Coat_Aureusvirus	Coat protein/Capsid protein/CP	3	5	8	11	0
		Coat_Benyvirus	Coat protein/Capsid protein/CP	1	4	5	24	0
		Coat_Bymovirus	Coat protein/Capsid protein/CP	5	11	16	94	0
		Coat_Carmovirus	Coat protein/Capsid protein/CP	4	10	14	108	0
		Coat_Dianthovirus	Coat protein/Capsid protein/CP	1	3	4	7	0
		Coat_furovirus	Coat protein/Capsid protein/CP	5	6	11	25	0
		Coat_Necrovirus	Coat protein/Capsid protein/CP	6	7	13	21	0
		Coat_Nepovirus	Coat protein/Capsid protein/CP	29	19	48	70	0
		Coat_Ophiovirus	Coat protein/Capsid protein/CP	1	1	2	3	0
		Coat_Pecluvirus	Coat protein/Capsid protein/CP	5	2	7	9	0
		Coat_Pomovirus	Coat protein/Capsid protein/CP	0	5	5	62	0
		Coat_Tobravirus	Coat protein/Capsid protein/CP	4	2	6	8	0
		Coat_Tombusvirus	Coat protein/Capsid protein/CP	8	12	20	39	0
		Coat_Tritimovirus	Coat protein/Capsid protein/CP	4	5	9	15	0
		Coat_unassigned_Secoviridae	Coat protein/Capsid protein/CP	1	5	6	12	0
		Coat_Varicosavirus	Coat protein/Capsid protein/CP	1	0	1	1	0
			Soil Borne Structural Total	*81*	*100*	*181*	*520*	*0*
	**Movement Protein**	Movement_7b_Carmovirus	Movement protein/Double gene block protein 2	0	4	4	8	0
		Movement_Cheravirus	Movement protein	1	0	1	1	0
		Movement_Dianthovirus	Movement protein	1	3	4	7	0
		Movement_Furovirus	Movement protein	2	3	5	8	0
		Movement_Pecluvirus	Movement protein	3	1	4	5	0
		Movement_Tombusvirus	Movement protein	0	1	1	2	0
		TGB1_Pomovirus	Triple-gene-block protein 1	1	3	4	12	0
			Soil Borne Movement Protein Total	*8*	*15*	*23*	*43*	*0*
	**Replication**	RdRp_Dianthovirus	RNA-dependent RNA polymerase	8	2	10	13	0
		RdRp_Nepovirus	RNA-dependent RNA polymerase	16	13	29	39	0
		Replicase_Aureusvirus	RNA-dependent RNA polymerase	3	3	6	9	0
		Replicase_Benyivirus	RNA-dependent RNA polymerase	1	2	3	9	0
		Replicase_Carmovirus	RNA-dependent RNA polymerase	5	20	25	55	0
		Replicase_Cheravirus	RNA-dependent RNA polymerase	2	2	4	6	0
		Replicase_Furovirus	RNA-dependent RNA polymerase	5	5	10	18	0
		Replicase_Necrovirus	RNA-dependent RNA polymerase	2	9	11	28	0
		Replicase_Pomovirus	RNA-dependent RNA polymerase	1	5	6	18	0
		Replicase_Tobravirus	RNA-dependent RNA polymerase	2	3	5	17	0
		Replicase_Tombusvirus	RNA-dependent RNA polymerase	5	9	14	22	0
			Soil Borne Replication Total	*50*	*73*	*123*	*234*	*0*
	**RNA Silencing**	Silencer_Aureusvirus	RNA silencing suppressor p14/silencing suppressor protein	0	2	2	5	0
		Silencer_Tombusvirus	RNA silencing suppressor p19/19 kDa symptom severity modulator	0	1	1	2	0
			Soil Borne RNA Silencing Total	*0*	*3*	*3*	*7*	*0*
	Soil Borne Virus Total			***139***	***191***	***330***	***804***	***0***
**Eukaryotic Viruses**	**Infection**	P48_hypoviridae	Papain-like protease p48	2	9	11	22	0
			Eukaryotic Infection Total	*2*	*9*	*11*	*22*	*0*
	**Replication**	Adenoviridae_protease	Adenain/Endoprotease/Late L3 23 kDa protein	10	22	32	73	0
		Astroviridae_RdRp	RNA-dependent RNA polymerase	76	12	88	104	0
		Hepeviridae_pORF1	Non-structural polyprotein pORF1	49	9	58	73	0
		Picobirnaviridae_RdRp	RNA-dependent RNA polymerase	26	9	35	43	0
		Polyomaviridae_LT_ag	Large T antigen	8	5	13	32	0
		RdRp_Alphaflexiviridae	RNA-dependent RNA polymerase	15	42	57	113	0
		RdRp_Caliciviridae	RNA-dependent RNA polymerase	109	30	139	190	0
		RdRp_chrysoviridae	RNA-dependent RNA polymerase	9	5	14	21	0
		RdRp_narnaviridae	RNA-dependent RNA polymerase	5	7	12	20	0
		RdRp_partitiviridae	RNA-dependent RNA polymerase	23	21	44	69	0
		RdRp_reoviridae	RNA-dependent RNA polymerase	10	18	28	61	0
		RdRp_reoviridae2	RNA-dependent RNA polymerase	21	18	39	59	0
		RdRp_totiviridae	RNA-dependent RNA polymerase	31	22	53	74	0
		RdRp_unclassed_totiviridae	RNA-dependent RNA polymerase	2	3	5	8	0
		Rep_geminiviridae	Replication-associated protein	22	61	83	175	0
		Replication_associated_protein_Bacillariodnavirus	Replication-associated protein	2	2	4	6	0
			Eukaryotic Replication Total	*418*	*286*	*704*	*1121*	*0*
	**Structure**	Adenoviridae_fiber	Fiber protein	17	36	53	114	0
		Adenoviridae_hexon	Hexon protein	57	60	117	322	0
		Astroviridae_capsid	Virus capsid polyprotein	80	25	105	131	0
		Capsid_Alphaflexiviridae	Coat protein	17	37	54	123	0
		Capsid_chrysoviridae	Capsid protein	5	5	10	15	0
		Coronaviridae_M_protein	Membrane protein	19	19	38	71	0
		Coronaviridae_spike	Spike glycoprotein	64	41	105	188	0
		Major_capsid_protein_partitiviridae	Coat protein	4	8	12	26	0
		Polyomaviridae_capsid	VP1 protein	3	20	23	117	0
		VP1capsid_Caliciviridae	Coat protein/Capsid protein/CP	133	47	180	250	0
		VP4_Pserotype_Rotavirus	Outer capsid protein VP4	28	14	42	62	0
		VP6_Rotavirus	Intermediate capsid protein VP6	9	5	14	31	0
		VP7_Gserotype_Rotavirus	Outer capsid glycoprotein VP7	33	8	41	51	0
			Eukaryotic Structure Total	*469*	*325*	*794*	*1501*	*0*
	**Toxin**	Enterotoxin_Rotavirus	Non-structural glycoprotein 4/Enterotoxin	3	6	9	50	0
		Killer_toxin	Killer toxin zygocin precursor	6	3	9	16	0
			Eukaryotic Toxin Total	*9*	*9*	*18*	*66*	*0*
	Eukaryotic Viruses Total			***898***	***629***	***1527***	***2710***	***0***
**Bacteriophage**	**Host Recognition**	Host_recognition_T2_type	Receptor-recognizing protein/Tail fiber adhesin	1	2	3	8	0
		Host_recognition_T4_type	Long tail fiber distal subunit	7	12	19	32	0
			Host Recognition Total	*8*	*14*	*22*	*40*	*0*
	**Lysis**	Endolysin_glycosidase	e Lysozyme murein hydrolase	0	4	4	8	0
		Endolysin_transglycosylase	Muramidase/Endolysin	4	13	17	32	0
		Holin_type	gp17.5	0	4	4	8	0
		Holin_type_3	T holin lysis mediator	0	2	2	4	0
		Holin_type1	Lambda-like group I holin	1	2	3	6	0
		Lysin	Lysin/N-acetylmuramoyl-L-alanine amidase/Endolysin	9	10	19	32	0
		Mycobacterium_LysB_lipase	Lysin B	0	2	2	4	0
			Lysis Total	*14*	*37*	*51*	*94*	*0*
	**Replication**	AsiA	AsiA anti-sigma 70 protein	0	1	1	2	0
		Clamp_loader_T4_ATPase	gp44 Clamp-loader subunit	4	2	6	8	0
		DNA_ligase	Putative DNA ligase	1	14	15	32	0
		DNA_polymerase_type_I	DNA polymerase	3	27	30	62	0
		Helicase_family_1_Dda_like	Dda DNA helicase	0	2	2	4	0
		Helicase_family_4	DNA primase-helicase subunit	4	19	23	47	0
		Helicase_family_4_DnaB_like	DnaB-like helicase	0	7	7	17	0
		Helicase_P4alpha_type	Putative P4-specific DNA primase	3	8	11	20	0
		Initiator_protein__theta_replication	Replication protein O	2	2	4	6	0
		Integrase_serine	Serine recombinase	1	2	3	7	0

			Integrase_tyrosine	Tyrosine recombinase	1	7	8	15	0
			MotA	Middle transcription protein A	1	0	1	1	0
			Primase	DNA primase	3	12	15	33	0
			RNA_dependent__RNA_polymerase	Replicase	13	9	22	31	0
			Single_strand_annealing_protein	Single strand annealing protein	2	13	15	29	0
			Sliding_clamp_T4	gp45 Sliding clamp DNA polymerase	3	4	7	11	0
			Ssb	ssDNA binding protein gp32	1	8	9	18	0
			T4_recomb_endonuclease	gp47 Recombination endonuclease subunit	4	14	18	32	0
			T4_RNase_H	RNaseH ribonuclease	0	2	2	4	0
			T4_type_portal_protein	Portal vertex protein gp20	5	5	10	17	0
			T5_genome_internalization_A1	A1 protein	0	2	2	8	0
			Terminal_protein_linear_DNA_phage	Terminal protein	0	5	5	11	0
			Terminase_large_subunit	Putative large terminase subunit	2	13	15	31	0
			Terminase_small_subunit	Truncated gp16 terminase subunit	1	3	4	7	0
			UvsW	RNA-DNA and DNA-DNA helicase/ATPase	2	8	10	18	0
				Bacteriophage Replication Total	*56*	*189*	*245*	*471*	*0*
		**Structural**	Contractile_central_tail_tube_protein	T4-like tail tube protein	3	9	12	22	0
			Contractile_tail_sheath_protein	Tail sheath protein gp18	7	8	15	22	0
			Major_capsid_protein	Major coat protein	12	24	36	62	0
			Non_contractile_major_tail_protein	Major tail protein	1	5	6	13	0
			Scaffold	gp21 Prohead core scaffold protein and protease	0	9	9	20	0
			Tape_measure_protein	Putative tail tape-measure protein	0	6	6	13	0
				Bacteriophage Structural Total	*23*	*61*	*84*	*152*	*0*
		Bacteriophage Total			***101***	***301***	***402***	***757***	***0***
	Virus Total				***1521***	***1336***	***2857***	***5264***	***0***
**Protozoa**	**Carbon**	**Carbon fixation**	Rubisco_Alveolata	Ribulose-1,5-bisphosphate carboxylase/oxygenase large subunit	12	5	17	29	19
			Rubisco_Glau_Rhiz_Cryp	Ribulose-1,5-bisphosphate carboxylase/oxygenase	0	5	5	12	5
			Rubisco_Haptophyceae	Ribulose-1,5-bisphosphate carboxylase/oxygenase large subunit	0	1	1	2	2
			Rubisco_Stramenopiles	Ribulose-1,5-bisphosphate carboxylase/oxygenase large subunit	1	1	2	10	5
			Rubisco_Viridiplantae	Ribulose-1,5-bisphosphate carboxylase/oxygenase large subunit	1	0	1	1	1
				Carbon Fixation Total	*14*	*12*	*26*	*54*	*32*
		**Chitin synthesis**	Chitin_synthase_protist	Transmembrane chitin synthase, glycosaminyl transferas	21	13	34	49	*0*
				Chitin Synthesis Total	*21*	*13*	*34*	*49*	*0*
		**Carbon degradaton**	Cellulase_GH45_Parabasalia	Cellulase or Glycosyl hydrolase family 45 [Parabasalia]	1	2	3	4	0
			Cellulase_GH7_Parabasalia	Cellulase or Glycosyl hydrolase family 7 [Parabasalia]	4	2	6	11	0
			Pectate_lyase_Oomycetes	Pectate lyase [Oomycetes]	7	11	18	41	0
			Pectin_lyase_Oomycetes	Pectin lyase [Oomycetes]	8	12	20	27	21
			Pg_Oomycetes	Polygalacturonase [Oomycetes]	24	15	39	52	0
			Xylose_isomerase_Oomycetes	Xylose isomerase [Oomycetes]	2	1	3	4	3
				Carbon Degradation Total	*46*	*43*	*89*	*139*	*24*
		Carbon Total			***81***	***68***	***149***	***242***	***56***
	**Defense**		Trichocyst_matrix_ciliophora	Trichocyst matrix protein	1	0	1	1	0
		Defense Total			***1***	***0***	***1***	***1***	***0***
	**Electron transfer**		Carbamate_kinase_Fornicata	Carbamate kinase	2	0	2	2	0
			Carbamate_kinase_Parabasalia	Carbamate kinase, putative	0	3	3	6	0
			Fe_hydrogenase_Heterolobosea	Type B Fe-hydrogenase	3	1	4	4	0
		Electron Transfer Total			***5***	***4***	***9***	***12***	***0***
	**Metal Homeostasis**	**Metallothionein**	Cd_metallothionein_ciliophora	Subfamily 7a metallothionein 1	3	1	4	10	0
			Cu_metallothionein_ciliophora	Copper-inducible metallothionein CuMT1	1	0	1	1	0
				Metallothionein Total	*4*	*1*	*5*	*11*	*0*
		**Silicon**	Silaffin	Silaffin precursor	1	0	1	1	3
			silicon_transporter	Silicon transporter	35	8	43	52	63
				Silicon Total	*36*	*8*	*44*	*53*	*66*
		**Vanadium**	V_BPO_protist	Vanadium-dependent bromoperoxidase 1	4	2	6	8	0
				Vanadium Total	*4*	*2*	*6*	*8*	*0*
		Metal Homeostasis Total			***44***	***11***	***55***	***72***	***66***
	**Movement**		PFR_Euglenozoa	Paraxonemal rod protein PAR 1	7	3	10	13	0
		Movement Total			***7***	***3***	***10***	***13***	***0***
	**Nitrogen**		Ammonium_transporter	Nitrite transporter	63	51	114	176	0
			Glutamate_synthase_protist	Ferredoxin-dependent glutamate synthase/Fd-GOGAT	24	14	38	48	0
			Glutamine_synthetase_protist	Glutamine synthetase/Glutamate--ammonia ligase	50	25	75	94	0
			Nitrate_transporter	Nitrate transporter	38	19	57	68	0
			Nitrite_reductase_protist	Ferredoxin nitrite reductase	6	6	12	15	12
		Nitrogen Total			***181***	***115***	***296***	***401***	***12***
	**Photosynthesis**		ChlG	Chlorophyll synthase subunit G	4	2	6	8	0
			PS2_D1_Euglenozoa	Photosystem II protein D1	0	2	2	6	0
			Violaxanthin_de_epoxidase_protist	Violaxanthin de-epoxidase	4	9	13	21	0
		Photosynthesis Total			***8***	***13***	***21***	***35***	***0***
	**Phylogenetic**		Actin_amoebozoa	Actin	7	10	17	29	23
			Actin_Cercozoa	Actin	5	1	6	7	*6*
			Actin_ciliophora	Actin	17	8	25	36	48
			Actin_Cryptophyta	Actin	1	1	2	3	4
			Actin_Haptophyceae	Actin	1	1	2	4	2
			Actin_Perkinsea	Actin, putative	7	9	16	18	22
			Actin_Rhizaria	Actin	11	6	17	26	19
			Actin_Stramenopiles	Beta-actin	24	11	35	41	41
			Actin_Viridiplantae	Beta-actin	27	16	43	53	45
			Alpha_tubulin_Choanoflagellida	Tubulin	3	0	3	3	0
			Alpha_tubulin_ciliophora	Alpha-tubulin	2	3	5	12	23
			Alpha_tubulin_Oxymonads	Alpha-tubulin	0	1	1	2	3
			Cox1_ciliophora	Cytochrome oxidase subunit 1	14	2	16	19	26
			Cox1_Viridiplantae	Cytochrome oxidase subunit I	9	17	26	62	29
			EF1a_amoebozoa	Elongation factor 1 alpha	2	0	2	2	6
			EF1a_Fungi_Metazoa_incertae_sedis	Translation elongation factor 1-alpha-like protein	0	1	1	7	2
			EF1a_Heterolobosea	Elongation factor 1 alpha	3	3	6	9	7
			EF1a_Jakobida	Elongation factor 1 alpha	3	0	3	3	4
			EF1a_Oxymonads	Elongation factor-1 alpha	4	4	8	13	8
			EF1a_Stramenopiles	Elongation factor-like	13	1	14	16	21
			GAPDH_Euglenozoa	Glyceraldehyde-3-phosphate dehydrogenase-like protein	13	6	19	28	28
			GAPDH_Heterolobosea	Glyceraldehyde-3-phosphate dehydrogenase	1	1	2	3	2
			GAPDH_Parabasalia	Glyceraldehyde-3-phosphate dehydrogenase 2	29	3	32	38	37
			Hsp90_Apusozoa	HSP90	1	0	1	1	1
			Hsp90_Choanoflagellida	90 kDa Heat-shock protein	7	3	10	11	11
			Hsp90_Fornicata	Heat shock protein 90	11	2	13	15	15
			Hsp90_Stramenopiles	Heat shock protein 90	20	8	28	37	36
			Polyubiquitin_Rhizaria	Polyubiquitin	2	1	3	5	3
		Phylogenetic Total			***237***	***119***	***356***	***503***	***472***
	**Sulfur assimilation**		AcsF_protist	Magnesium-protoporphyrin IX monomethyl ester aerobic oxidative cyclase	1	12	13	30	0
			APS_kinase_protist	Putative bifunctional SAT/APS kinase 2, related	9	11	20	30	0
			APS_reductase_protist	APS reductase	1	3	4	9	0
			ATP_sulfurylase_protist	ATP sulfurylase Ats1	13	8	21	28	0
			Cysteine_synthase_protist	Cysteine synthase	52	44	96	168	0
			LPOR_protist	chloroplast light-dependent protochlorophyllide reductase	4	4	8	12	0
			Mg_chelatase	Magnesium chelatase subunit H, putative chloroplast precursor	28	49	77	125	83
			Nitrite_transporter	Nitrite transporter	15	14	29	40	0
			Serine_acetyltransferase_protist	Serine acetyltransferase	11	18	29	44	0
			Sulfate_transporter_protist	Sulfate permease	57	43	100	137	0
			Sulfite_reductase_protist	Ferredoxin-sulfite reductase	5	5	10	14	0

			Zeaxanthin_epoxidase_protist	Zeaxanthin epoxidase	8	13	21	28	0
		Sulfur Assimilation Total			***204***	***224***	***428***	***665***	***83***
	**Virulence**	**Toxin**	Glucanase_inhibitor_Oomycetes	Glucanase inhibitor protein, putative	5	1	6	6	0
			Necrosis_Oomycetes	Necrosis inducing protein	22	11	33	42	0
			PcF_Oomycetes	PcF and SCR74-like cys-rich secreted peptide, putative	4	9	13	25	0
			Serine_protease_inhibitor_Oomycetes	Serine protease inhibitor	3	8	11	25	0
			Trehalose_synthase_Cercozoa	Trehalose-6-phosphate synthase	1	0	1	1	0
				Toxin Total	*35*	*29*	*64*	*99*	*0*
		**Other**	ATR13_Oomycetes	Avirulence protein ATR13	1	0	1	1	0
			ATR1NdWsB_Oomycetes	Avirulence protein ATR1NdWsB	0	3	3	10	0
			AVR1_Oomycetes	Avr1 secreted RxLR effector peptide protein	1	1	2	3	0
			AVR1a_Oomycetes	Avirulence effector protein Avr1a	1	2	3	4	0
			AVR1b_Oomycetes	Elicitor Avr1b	3	2	5	10	0
			CBEL_Oomycetes	Cellulose binding elicitor lectin (CBEL), putative	1	2	3	5	0
			CRN_Oomycetes	Crinkling and necrosis-inducing protein	4	3	7	8	0
			INF1_elicitin_Oomycetes	Putative elicitin protein RAM3X	31	16	47	70	0
				Other Protozoa Virulence Total	*42*	*29*	*71*	*111*	*0*
		Virulence Total			***77***	***58***	***135***	***210***	***0***
	**Protozoa Total**				***845***	***615***	***1460***	***2154***	***689***
**Fungi**	**Carbon degradation**	**Cellulose**	Axe	Acetyl xylan esterase	48	20	68	75	70
			Cellobiase	Cellobiase or beta-glucosidase	187	534	721	1429	763
			Exoglucanase	Exoglucanase	144	75	219	314	267
				Cellulose Total	*379*	*629*	*1008*	*1818*	*1100*
		**Chitin**	Chitin_deacetylase_fungi	Chitin deacetylase [Fungi]	28	18	46	55	52
				Chitin Total	*28*	*18*	*46*	*55*	*52*
		**Cutin**	Cutinase	Cutinase	96	140	236	364	298
				Cutin Total	*96*	*140*	*236*	*364*	*298*
		**Glyoxylate cycle**	AceA_fungi	Isocitrate lyase AcuD	24	7	31	38	*0*
			AceB_fungi	Malate synthase, putative	23	12	35	49	0
				Glyoxylate cycle Total	*47*	*19*	*66*	*87*	*0*
		**Glucose**	Glucose_oxidase_fungi	Glucose oxidase/glucose-1-oxidase					45
				Glucose Total	*0*	*0*	*0*	*0*	*45*
		**Hemicellulose**	Xylose_reductase_fungi	Xylose reductase [Fungi]	2	1	3	4	0
				Hemicellulose Total	*2*	*1*	*3*	*4*	*0*
		**Inulin**	Inulinase	Inulinase or exo-inulinase	11	16	27	47	35
				Inulin Total	*11*	*16*	*27*	*47*	*35*
		**Lactose**	Lactase_fungi	Lactase or Beta-galactosidase [Fungi]	58	18	76	79	101
				Lactose Total	*58*	*18*	*76*	*79*	*101*
		**Lignin**	Ligninase	Ligninase or Lignin peroxidase	12	10	22	42	25
				Lignin Total	*12*	*10*	*22*	*42*	*25*
		**Lipids**	Lipase_fungi	Triacylglycerol acylhydrolase/triacylglycerol lipase					11
				Lipids Total	*0*	*0*	*0*	*0*	*11*
		**Other**	Alpha_galactosidase_fungi	Alpha-galactosidase [Fungi]	63	20	83	89	99
				Other Fungi Carbon Degradation Total	*63*	*20*	*83*	*89*	*99*
		**Pectin**	Endopolygalacturonase_fungi	Endopolygalacturonase [Fungi]	39	18	57	72	95
			Exopolygalacturonase_fungi	Exopolygalacturonase or polygalacturonase [Fungi]	74	31	105	117	145
			Pec_Cdeg	Pectate lyase [Fungi]	78	52	130	162	157
			Pel_Cdeg	Pectin lyase [Fungi]	13	3	16	16	21
			Pme_Cdeg	Pectin methylesterase [Fungi]	41	22	63	70	80
				Pectin Total	*245*	*126*	*371*	*437*	*498*
		**Phospholipids**	Phospholipase_A2_fungi	Phospholipase A2 (PlaA)	33	24	57	74	70
			Phospholipase_C_fungi	Phosphoinositide-specific phospholipase C, putative	73	41	114	130	146
			Phospholipase_D_fungi	Hypothetical protein NECHADRAFT_66296	75	36	111	127	139
				Phospholipids Total	*181*	*101*	*282*	*331*	*355*
		**Protein**	protease_aspartate_fungi	Aspartate protease					86
			protease_cysteine_fungi	Cysteine protease					23
			protease_serine_fungi	Serine protease					170
				Protein Total	*0*	*0*	*0*	*0*	*279*
		**Starch**	Glucoamylase	Glucoamylase	126	196	322	504	361
				Starch Total	*126*	*196*	*322*	*504*	*361*
		**Sucrose**	dextranase_fungi	Dextranase					6
			invertase_fungi	Invertase/beta-fructofuranosidase/saccharase					18
				Sucrose Total	*0*	*0*	*0*	*0*	*24*
		**Tannins**	Tannase_Cdeg	Tannase	55	28	83	98	119
				Tannins Total	*55*	*28*	*83*	*98*	*119*
		Carbon Degradation Total			***1303***	***1322***	***2625***	***3955***	***3402***
	**Metal Homeostasis**	**Aluminum Transport**	Al_fungi	Aluminum resistance putative divalent cation transporter	4	4	8	11	0
				Aluminum Transport Total	*4*	*4*	*8*	*11*	*0*
		**Arsenic Transport**	ArsA_fungi	Arsenical pump-driving ATPase	18	17	35	48	0
				Arsenic Transport Total	*18*	*17*	*35*	*48*	*0*
		**Iron Transport**	Ferric_reductase_transporter	Ferric reductase transmembrane component (Ferric-chelate reductase)	32	42	74	128	0
			Ferroxidase_high_affinity	Conidial pigment biosynthesis oxidase Abr1/brown 1	76	42	118	580	0
			Iron_permease_high_affinity	Plasma membrane iron permease	30	21	51	75	0
			Iron_permease_low_affinity	Manganese transporter SMF1	0	2	2	4	0
			SidA	L-Ornithine 5-monooxygenase	23	9	32	37	0
			SidC	Ferrichrome synthetase Sib1	12	5	17	19	0
			Siderophore_transporter	Siderochrome-iron transporter MirC	91	94	185	261	0
			Vacuolar_iron_transport	Vacuolar iron transporter	30	15	45	62	0
				Iron Transport Total	*294*	*230*	*524*	*1166*	*0*
		**Potassium Transport**	Trk_fungi	Potassium transport protein 1/High-affinity potassium transport protein	127	62	189	216	0
				Potassium Transport Total	*127*	*62*	*189*	*216*	*0*
		**Multiple Metal Sequestration**	Metallothionein	Putative metallothionein	1	2	3	5	0
				Multiple Metal Sequestration Total	*1*	*2*	*3*	*5*	*0*
		Metal Homeostasis Total			***444***	***315***	***759***	***1446***	***0***
	**Organic Remediation**	**Aromatics**	Aromatic_peroxygenase	Aromatic peroxygenase precursor	3	1	4	4	4
			Cl_peroxidase_fungi	Vanadium chloroperoxidase	1	3	4	9	4
			P450aro	PAH-inducible cytochrome P450 monooxygenase	77	33	110	116	141
				Aromatics Total	*81*	*37*	*118*	*129*	*149*
		**Halogenated compounds**	Dyp	Decolorizing Peroxidase/dye-decolorizing peroxidase	23	6	29	29	32
				Halogenated Compounds Total	*23*	*6*	*29*	*29*	*32*
		**Other**	Cah_fungi	Cyanamide hydratase, putative	5	4	9	12	0
				Other Fungi Organic Remediation Total	*5*	*4*	*9*	*12*	*0*
		Organic Remediation Total			***109***	***47***	***156***	***170***	***181***
	**Phosphorous**		Phospholipase_B_fungi	Phospholipase B/lysophospholipase/lysophospholipase-transacylase	56	32	88	113	118
		Phosphorous Total			***56***	***32***	***88***	***113***	***118***
	**Antibiotic resistance**		MFS_fungi	Multidrug resistance protein 10	16	11	27	35	0
			ABC_multidrug_fungi	TPA: ABC multidrug transporter (Eurofung)	248	130	378	437	0
		Antibiotic resistance Total			***264***	***141***	***405***	***472***	***0***
	**Virulence**	**Cellular components**	Conidial_laccase	Conidial pigment biosynthesis oxidase Arb2/brown2	6	5	11	11	0
				Cellular components Total	*6*	*5*	*11*	*11*	*0*
		**Colonization/Infection**	Als_adhesin_fungi	Agglutinin-like protein, putative; cell surface glycoprotein, putative	2	3	5	13	0
			Metalloprotease_fungi	Neutral protease	70	30	100	112	0
				Colonization/Infection Total	*72*	*33*	*105*	*125*	*0*
		**Other**	Calcineurin_A_fungi	Serine/threonine-protein phosphatase 2B catalytic subunit/Calmodulin-dependent calcineurin A subunit/Calcineurin A1	8	6	14	20	0
				Other Fungi Virulence Total	*8*	*6*	*14*	*20*	*0*
		**Survival**	Catalase_KatG_fungi	Catalase-peroxidase/Catalase-2	15	5	20	24	0
			Superoxide_dismutase_fungi	Cu-Zn superoxide dismutase	18	6	24	29	0
				Survival Total	*33*	*11*	*44*	*53*	*0*

		**Toxin**	DmaW_ergot	Tryptophan dimethylallyltransferase/4-dimethylallyltryptophan synthase/All-trans-hexaprenyl-diphosphate synthase/L-tryptophan dimethylallyl transferase/DMATS	33	26	59	78	0
			Ochratoxin_PKS	Ochratoxin A non-ribosomal peptide synthetase	11	2	13	14	0
			Patulin_6MSAS	Putative 6-MSAS-type polyketide synthase	8	2	10	11	0
			Trichodiene_synthase	Trichodiene synthase	3	2	5	7	0
				Toxin Total	*55*	*32*	*87*	*110*	*0*
		Fungi Virulence Total			***174***	***87***	***261***	***319***	***0***
	**Sulfur cycling**		ATP_sulphurylase	Sulfate adenylyltransferase/Sulfate adenylate transferase/ATP-sulfurylase	17	8	25	32	0
			PAPS_reductase	Phosphoadenosine phosphosulfate reductase	14	15	29	42	0
			Sulfate_transporter	Sulfate permease	81	38	119	134	0
		Sulfur Cycling Total			***112***	***61***	***173***	***208***	***0***
	**Ammonification**		glnA_fungi	Glutamine synthetase	22	17	39	64	0
			urease_arb_fungi	Urease	0	2	2	3	0
		Ammonification Total			***22***	***19***	***41***	***67***	***0***
	**N Assimilation**		Nitrate_reductase	Nitrate reductase	74	55	129	241	171
			P450nor	Cytochrome P450nor	1	0	1	1	*0*
		N Assimilation Total			***75***	***55***	***130***	***242***	***171***
	Fungi Total				***2559***	***2079***	***4638***	***6992***	***3872***
**GyrB**	**Phylogenetic**		GyrB	DNA topoisomerase type IIA subunit B region 2 domain protein	532	2234	2766	9997	0
	GyrB Total				***532***	***2234***	***2766***	***9997***	***0***
**Other Categories**									
**Electron transport**	**Photosynthetic**	**Bacteriochlorophyll**	BchG	Bacteriochlorophyll/chlorophyll a synthase	28	65	93	163	0
			BchQ	Bacteriochlorophyll c8 methyltransferase	0	4	4	8	0
			BciA	3,8-Divinyl protochlorophyllide a 8-vinyl reductase, putative chloroplast precursor	3	6	9	15	0
			LPOR	Light-dependent protochlorophyllide oxidoreductase/protochlorophyllide oxidoreductase/lpor/NADPH-protochlorophyllide oxidoreductase	20	73	93	171	0
				Bacteriochlorophyll Total	*51*	*148*	*199*	*357*	*0*
		**Bilin**	Biliverdin_reductase	Oxidoreductase, putative biliverdin reductase	1	5	6	11	0
			PcyA	Phycocyanobilin:ferredoxin oxidoreductase	8	17	25	42	0
			PebA	15,16-Dihydrobiliverdin:ferredoxin oxidoreductase	1	5	6	11	0
			PebB	Phycoerythrobilin:ferredoxin oxidoreductase	4	9	13	22	0
				Bilin Total	*14*	*36*	*50*	*86*	*0*
		**Carotenoid**	Beta_carotene_ketolase_crtW	Beta-carotene hydroxylase	11	26	37	87	0
			Beta_carotene_ketolase_protist	Beta-carotene ketolase/Beta-carotene oxygenase	1	4	5	16	0
			Blh	Beta-carotene 15,15-dioxygenase/carotene dioxygenase/carotene 15,15-dioxygenase/beta-carotene 15,15-monooxygenase	3	31	34	65	0
			Diapophytoene_synthase	Dehydrosqualene synthase/4,4'-diapophytoene synthase/Diapophytoene synthase	3	6	9	15	0
			GGPP_synthase	Phytoene synthase	24	40	64	107	0
			Hydroxyneurosporene_synthase	Hydroxyneurosporene synthase	4	46	50	100	0
			Lycopene_beta_cyclase	Lycopene cyclase, beta and epsilon	22	66	88	157	0
			Lycopene_epsilon_cyclase	Putative lycopene epsilon cyclase	2	24	26	50	0
			Methoxyneurosporene_desaturase	Putative methoxyneurosporene dehydrogenase	5	9	14	24	0
			Phytoene_desaturase_protist	Hypothetical protein VOLCADRAFT_88495	12	10	22	29	0
			Phytoene_synthase	Phytoene synthase	59	350	409	796	0
			Phytoene_synthase_protist	Phytoene synthase	11	9	20	28	0
			Spheroidene_monooxygenase	Spheroidene monooxygenase	4	19	23	42	0
			Zeaxanthin_glucosyltransferase	Glycosyltransferase, MGT family	9	4	13	17	0
				Carotenoid Total	*170*	*644*	*814*	*1533*	*0*
		**Chlorophyll**	AcsF	Magnesium-protoporphyrin IX monomethyl ester aerobic oxidative cyclase	7	78	85	163	0
				Chlorophyll Total	*7*	*78*	*85*	*163*	*0*
		**Rhodopsin**	Bacteriorhodopsin	Bacteriorhodopsin	7	19	26	44	33
				Rhodopsin Total	*7*	*19*	*26*	*44*	*33*
		Photosynthetic Total			***249***	***925***	***1174***	***2183***	***33***
	**Cytochrome**	**c-type**	C_type_cytochrome	Multicopper oxidase family protein	0	21	21	42	0
			C_type_cytochrome_1	Cytochrome C oxidase subunit transmembrane protein	1	45	46	91	0
			C_type_cytochrome_3	Cytochrome C class I	3	35	38	77	0
			C_type_cytochrome_4	Cytochrome c-type biogenesis protein DsbD, protein-disulfide reductase	4	10	14	37	0
			C_type_cytochrome_5	Conserved hypothetical protein	0	1	1	2	0
			C_type_cytochrome_6	Cytochrome c assembly protein	3	50	53	103	0
			C_type_cytochrome_b	Periplasmic nitrate reductase subunit NapB	19	12	31	43	0
				c-type Total	*30*	*174*	*204*	*395*	*0*
		**other**	Cytochrome	Cytochrome c, class I	296	146	442	585	0
			P450	Putative cytochrome P450	6	21	27	47	0
				Other Cytochrome Total	*302*	*167*	*469*	*632*	*0*
		Cytochrome Total			***332***	***341***	***673***	***1027***	***0***
	**Hydrogenase**		Hydrogenase	Nickel-dependent hydrogenase, large subunit	24	65	89	153	0
			Ni_Fe_hydrogenase	Putative (Fe) hydrogenase, HymD subunit	7	17	24	38	0
		Hydrogenase Total			***31***	***82***	***113***	***191***	*0*
	Electron Transport Total				***612***	***1348***	***1960***	***3401***	***33***
**Total**					31247	133592	161961	385417	57498

[A]Values are for 5.0M
