# Supplementary material for: Functional Gene Array-Based Ultrasensitive and Quantitative Detection of Microbial Populations in Complex Communities
Source: mSystems. 2019 Jun 18;4(4):e00296-19. doi: 10.1128/mSystems.00296-19 (PMC6581690; doi:10.1128/mSystems.00296-19)
Supplement: TABLE S2 [file mSystems.00296-19-st002.docx]

**Table S2**. Summary of probes on GeoChip 5.0M based on the phylogenetic distribution of the functional genes.

| Domain | Kingdom | Phylum (order for virus) | No. of class | No. of order | No. of family | No. of genus | No. of species | No. of strains | No. of gene | No. of covered CDS | No. of total probe | No. of group-specific probe | No. of sequence-specific probe |
| --- | --- | --- | --- | --- | --- | --- | --- | --- | --- | --- | --- | --- | --- |
| **Archaea** | **Archaea** | Crenarchaeota | 2 | 6 | 9 | 22 | 43 | 57 | 142 | 13921 | 1312 | 1133 | 179 |
|  |  | Euryarchaeota | 10 | 12 | 23 | 69 | 133 | 187 | 229 | 33323 | 3992 | 3625 | 367 |
|  |  | Korarchaeota | 1 | 1 | 1 | 1 | 1 | 1 | 33 | 3718 | 35 | 33 | 2 |
|  |  | Nanoarchaeota | 1 | 1 | 1 | 1 | 1 | 1 | 6 | 12 | 6 | 6 | 0 |
|  |  | Thaumarchaeota | 1 | 4 | 5 | 7 | 9 | 14 | 46 | 5218 | 95 | 73 | 22 |
|  |  | unclassified | 1 | 1 | 1 | 1 | 1 | 25 | 46 | 1885 | 288 | 115 | 173 |
|  |  | **Archaea Total** | **16** | **25** | **40** | **101** | **188** | **285** | **502** | **58077** | **5728** | **4985** | **743** |
| **Bacteria** | **Bacteria** | Acidobacteria | 5 | 4 | 4 | 8 | 13 | 18 | 155 | 20180 | 745 | 715 | 30 |
|  |  | Actinobacteria | 1 | 8 | 48 | 120 | 351 | 753 | 381 | 80060 | 15394 | 13618 | 1776 |
|  |  | Aquificae | 1 | 2 | 4 | 12 | 17 | 22 | 127 | 19379 | 533 | 498 | 35 |
|  |  | Bacteroidetes | 7 | 10 | 23 | 107 | 239 | 394 | 267 | 58970 | 9661 | 8790 | 871 |
|  |  | Caldiserica | 1 | 1 | 1 | 1 | 1 | 1 | 13 | 38 | 19 | 19 | 0 |
|  |  | Candidatus poribacteria | 1 | 1 | 1 | 1 | 1 | 1 | 2 | 5 | 4 | 1 | 3 |
|  |  | Candidatus saccharibacteria | 1 | 1 | 1 | 1 | 1 | 3 | 9 | 22 | 17 | 4 | 13 |
|  |  | Chlamydiae | 2 | 1 | 4 | 7 | 15 | 23 | 74 | 12652 | 241 | 230 | 11 |
|  |  | Chlorobi | 1 | 1 | 1 | 6 | 15 | 20 | 138 | 21932 | 995 | 944 | 51 |
|  |  | Chloroflexi | 8 | 11 | 12 | 14 | 19 | 28 | 209 | 32839 | 1353 | 1289 | 64 |
|  |  | Chrysiogenetes | 1 | 1 | 1 | 2 | 2 | 2 | 33 | 81 | 41 | 40 | 1 |
|  |  | Cyanobacteria | 2 | 8 | 12 | 57 | 97 | 203 | 237 | 46219 | 4727 | 4209 | 518 |
|  |  | Deferribacteres | 1 | 1 | 1 | 4 | 4 | 4 | 81 | 2583 | 165 | 154 | 11 |
|  |  | Deinococcus-thermus | 2 | 3 | 4 | 7 | 18 | 27 | 172 | 21352 | 1026 | 978 | 48 |
|  |  | Dictyoglomi | 1 | 1 | 1 | 1 | 2 | 2 | 51 | 9447 | 99 | 98 | 1 |
|  |  | Elusimicrobia | 2 | 2 | 2 | 2 | 2 | 2 | 34 | 5713 | 50 | 49 | 1 |
|  |  | Fibrobacteres | 2 | 1 | 1 | 1 | 1 | 2 | 50 | 6671 | 69 | 64 | 5 |
|  |  | Firmicutes | 6 | 10 | 42 | 176 | 593 | 1556 | 429 | 107993 | 25601 | 21921 | 3680 |
|  |  | Fusobacteria | 2 | 1 | 2 | 5 | 15 | 38 | 111 | 17980 | 541 | 493 | 48 |
|  |  | Gemmatimonadetes | 1 | 1 | 1 | 1 | 1 | 1 | 51 | 8772 | 71 | 69 | 2 |
|  |  | Ignavibacteriae | 1 | 1 | 2 | 2 | 2 | 2 | 27 | 85 | 43 | 42 | 1 |
|  |  | Lentisphaerae | 2 | 3 | 2 | 2 | 2 | 2 | 56 | 4823 | 93 | 91 | 2 |
|  |  | Nitrospirae | 2 | 2 | 2 | 4 | 5 | 10 | 114 | 8777 | 237 | 197 | 40 |
|  |  | Planctomycetes | 3 | 3 | 4 | 15 | 23 | 32 | 183 | 26590 | 994 | 765 | 229 |
|  |  | Poribacteria | 1 | 1 | 1 | 1 | 1 | 1 | 13 | 25 | 13 | 12 | 1 |
|  |  | Proteobacteria | 7 | 47 | 115 | 508 | 1132 | 2855 | 823 | 187705 | 68319 | 59749 | 8570 |
|  |  | Spirochaetes | 2 | 1 | 3 | 8 | 48 | 80 | 172 | 11471 | 1007 | 829 | 178 |
|  |  | Synergistetes | 2 | 2 | 2 | 11 | 13 | 14 | 109 | 11410 | 472 | 392 | 80 |
|  |  | Tenericutes | 1 | 3 | 4 | 7 | 39 | 82 | 56 | 11489 | 266 | 218 | 48 |
|  |  | Thermodesulfobacteria | 1 | 1 | 1 | 2 | 5 | 6 | 50 | 162 | 85 | 77 | 8 |
|  |  | Thermotogae | 1 | 1 | 2 | 8 | 19 | 26 | 93 | 16870 | 588 | 553 | 35 |
|  |  | Verrucomicrobia | 4 | 6 | 8 | 14 | 17 | 30 | 172 | 29149 | 947 | 824 | 123 |
|  |  | unclassified | 1 | 2 | 2 | 7 | 8 | 239 | 233 | 33937 | 6737 | 2583 | 4154 |
|  |  | **Bacteria Total** | **76** | **142** | **314** | **1122** | **2721** | **6479** | **4725** | **815381** | **141153** | **120515** | **20638** |
| Eukaryota | Fungi | Ascomycota | 10 | 25 | 70 | 164 | 245 | 402 | 201 | 19120 | 6995 | 2471 | 4524 |
|  |  | Basidiomycota | 7 | 24 | 61 | 104 | 122 | 175 | 119 | 8732 | 1549 | 530 | 1019 |
|  |  | Chytridiomycota | 1 | 2 | 2 | 1 | 2 | 2 | 7 | 10 | 10 | 0 | 10 |
|  |  | Glomeromycota | 1 | 2 | 2 | 3 | 3 | 3 | 9 | 71 | 12 | 7 | 5 |
|  |  | Microsporidia | 1 | 1 | 7 | 9 | 14 | 16 | 25 | 1900 | 82 | 47 | 35 |
|  |  | Neocallimastigomycota | 1 | 1 | 1 | 3 | 3 | 8 | 7 | 136 | 28 | 4 | 24 |
|  |  | unclassified | 1 | 4 | 10 | 13 | 15 | 20 | 19 | 725 | 180 | 90 | 90 |
|  |  | **Fungi Total** | **22** | **59** | **153** | **297** | **404** | **626** | **387** | **30694** | **8856** | **3149** | **5707** |
|  | Protist | Apicomplexa | 2 | 3 | 6 | 7 | 19 | 47 | 44 | 1875 | 374 | 123 | 251 |
|  |  | Bacillariophyta | 5 | 10 | 14 | 16 | 19 | 31 | 37 | 1269 | 218 | 96 | 122 |
|  |  | Chromerida | 1 | 1 | 1 | 2 | 2 | 2 | 3 | 6 | 5 | 1 | 4 |
|  |  | Euglenida | 1 | 4 | 6 | 6 | 6 | 7 | 3 | 17 | 12 | 4 | 8 |
|  |  | Eustigmatophyceae | 1 | 1 | 1 | 1 | 1 | 1 | 1 | 1 | 1 | 0 | 1 |
|  |  | Haplosporidia | 1 | 1 | 2 | 4 | 4 | 5 | 1 | 12 | 6 | 3 | 3 |
|  |  | Phaeophyceae | 1 | 2 | 2 | 2 | 2 | 2 | 19 | 1129 | 28 | 6 | 22 |
|  |  | Pinguiophyceae | 1 | 1 | 1 | 1 | 1 | 1 | 1 | 1 | 1 | 0 | 1 |
|  |  | Xanthophyceae | 1 | 2 | 2 | 2 | 2 | 2 | 4 | 7 | 5 | 2 | 3 |
|  |  | **Protist Total** | **14** | **25** | **35** | **41** | **56** | **98** | **113** | **4317** | **650** | **235** | **415** |
|  | Metazoa | Arthropoda | 2 | 5 | 5 | 7 | 7 | 7 | 4 | 19 | 13 | 5 | 8 |
|  |  | Chordata | 2 | 3 | 3 | 3 | 3 | 3 | 3 | 9 | 7 | 2 | 5 |
|  |  | Echinodermata | 1 | 1 | 1 | 1 | 1 | 1 | 1 | 2 | 3 | 1 | 2 |
|  |  | Nematoda | 1 | 2 | 3 | 3 | 3 | 4 | 6 | 9 | 8 | 2 | 6 |
|  |  | Platyhelminthes | 1 | 1 | 1 | 1 | 1 | 1 | 1 | 5 | 3 | 2 | 1 |
|  |  | **Metazoa Total** | **7** | **12** | **13** | **15** | **15** | **16** | **15** | **44** | **34** | **12** | **22** |
|  | Viridiplantae | Chlorophyta | 7 | 15 | 23 | 37 | 39 | 57 | 39 | 694 | 444 | 221 | 223 |
|  |  | Streptophyta | 7 | 11 | 12 | 12 | 12 | 13 | 15 | 432 | 31 | 12 | 19 |
|  |  | **Viridiplantae Total** | **14** | **26** | **35** | **49** | **51** | **70** | **54** | **1126** | **475** | **233** | **242** |
|  | unclassified | unclassified | 24 | 74 | 116 | 178 | 195 | 264 | 165 | 3148 | 1401 | 569 | 832 |
| Viruses | Viruses | Nidovirales | - | - | 1 | 7 | 18 | 75 | 2 | 259 | 143 | 60 | 83 |
|  |  | Picornavirales | - | - | 6 | 24 | 47 | 275 | 7 | 707 | 518 | 144 | 374 |
|  |  | Tymovirales | - | - | 1 | 6 | 8 | 46 | 2 | 236 | 111 | 79 | 32 |
|  |  | Caudovirales | - | - | 4 | 26 | 30 | 156 | 40 | 1506 | 347 | 279 | 68 |
|  |  | unclassified | - | - | 28 | 104 | 208 | 814 | 78 | 3320 | 1729 | 768 | 961 |
|  |  | **Virus Total** |  |  | **156** | **345** | **506** | **1630** | **294** | **9176** | **4249** | **1899** | **2350** |
| unclassified | - | - | - | - | - | - | - | 33 | 125 | 2561 | 816 | 293 | 523 |
