# Supplementary material for: Functional Gene Array-Based Ultrasensitive and Quantitative Detection of Microbial Populations in Complex Communities
Source: mSystems. 2019 Jun 18;4(4):e00296-19. doi: 10.1128/mSystems.00296-19 (PMC6581690; doi:10.1128/mSystems.00296-19)
Supplement: TABLE S3 [file mSystems.00296-19-st003.docx]

**Table S3.** Overall differences between GeoChip 4 and 5.

| **Entry** | **GeoChip 4** | **GeoChip 5.0S** | **GeoChip 5.0M** | **% increases in GeoChip 5.0M compared with GeoChip 4** |
| --- | --- | --- | --- | --- |
| **Manufacturers** | NimbleGen | Agilent | | - |
| **Feature shape** | Square | Circular | | - |
| **Feature size** | 13 µm × 13 µm | 30 micron (diameter) | | - |
| **Maximum features per array** | 135,000 | 60,000 | 180,000 | - |
| **No. of arrays per slide** | 12 | 8 | 4 | - |
| **No. of genes** | 410 | 373 | 1,447 | +253% |
| **No. of probes** | 82,074 | 57,498 | 161,961 | +97% |
| **No. of sequence-specific probes** | 18,098 | 11,819 | 30,640 | +69% |
| **No. of group-specific probes** | 63,976 | 45,679 | 131,321 | +105% |
| **No. of covered CDS** | 141,995 | 151,797 | 365,651 | +158% |
| **No. of covered strains** | 5,247 | 6,507 | 8,987 | +71% |
| **16S positive controls** | 640 | 1,536 | 5,282 | +540% |
| **Controls from thermophiles** | 1,689 | 1,130 | 3,378 | +100% |
| **Universal standards** | 6,000 | 480 | 1,360 | -78.6% |
| **Agilent negative controls** | - | 1,565 | 3,390 | - |
