# Supplementary figures and images for: Functional Gene Array-Based Ultrasensitive and Quantitative Detection of Microbial Populations in Complex Communities
Source: mSystems. 2019 Jun 18;4(4):e00296-19. doi: 10.1128/mSystems.00296-19 (PMC6581690; doi:10.1128/mSystems.00296-19)

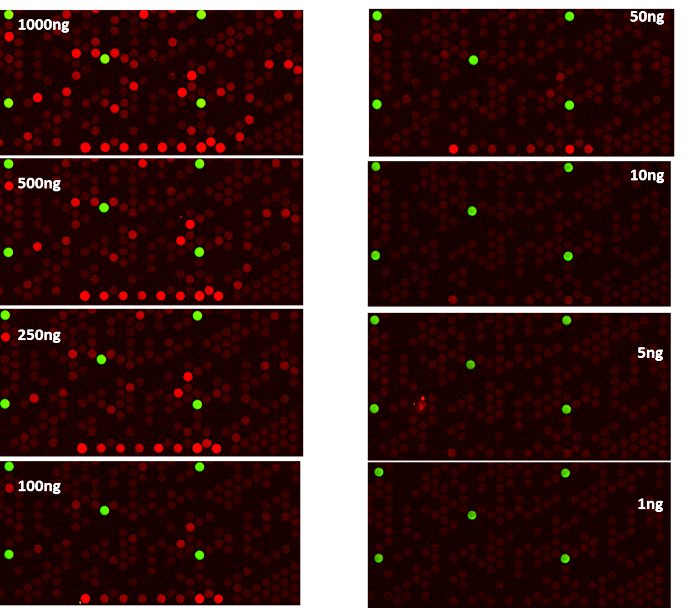

Supplement: FIG S1 [file mSystems.00296-19-sf001.tif]

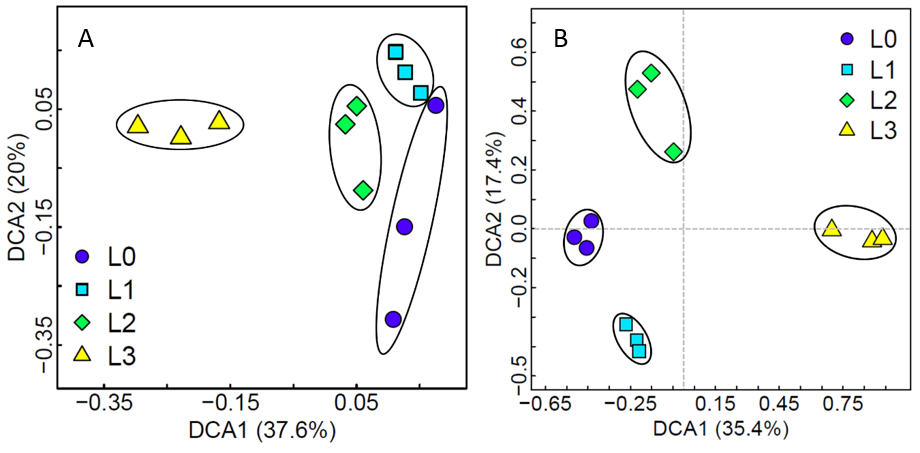

Supplement: FIG S2 [file mSystems.00296-19-sf002.tif]

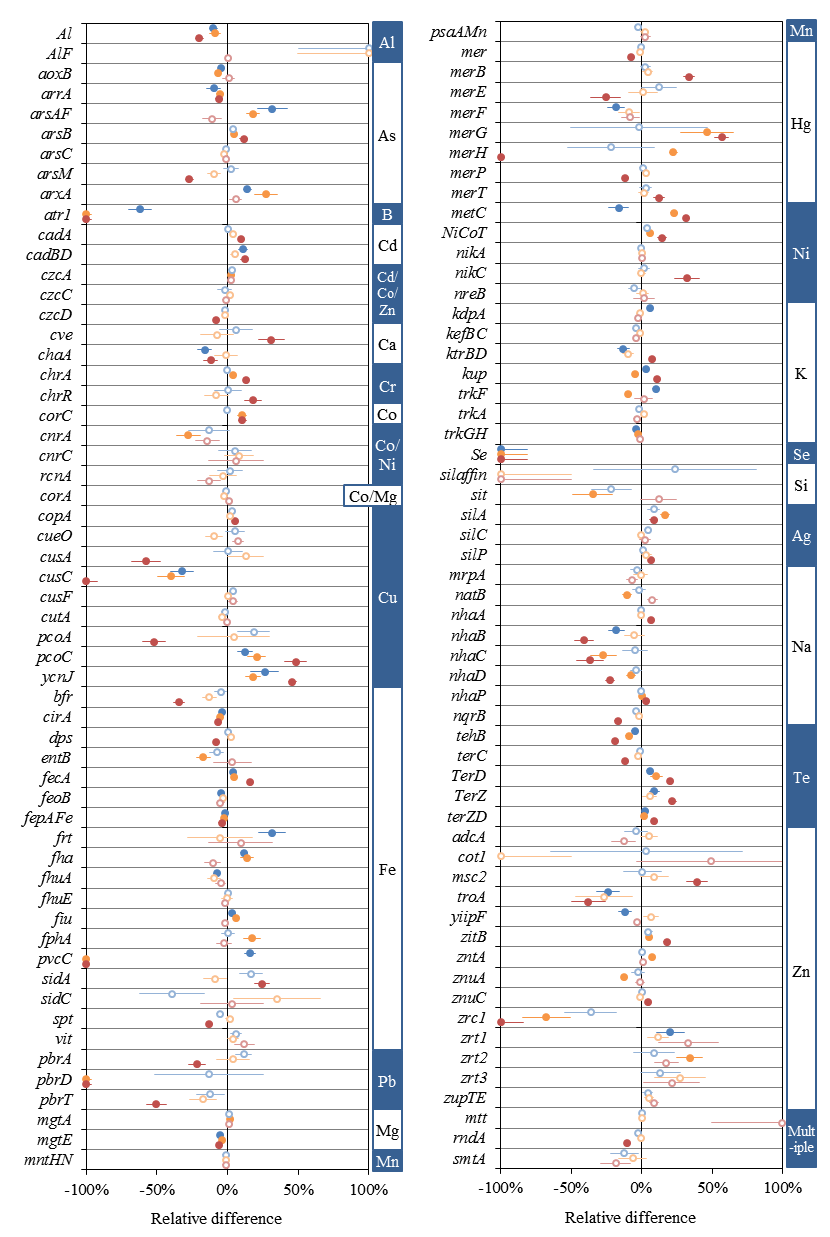

Supplement: FIG S3 [file mSystems.00296-19-sf003.tif]

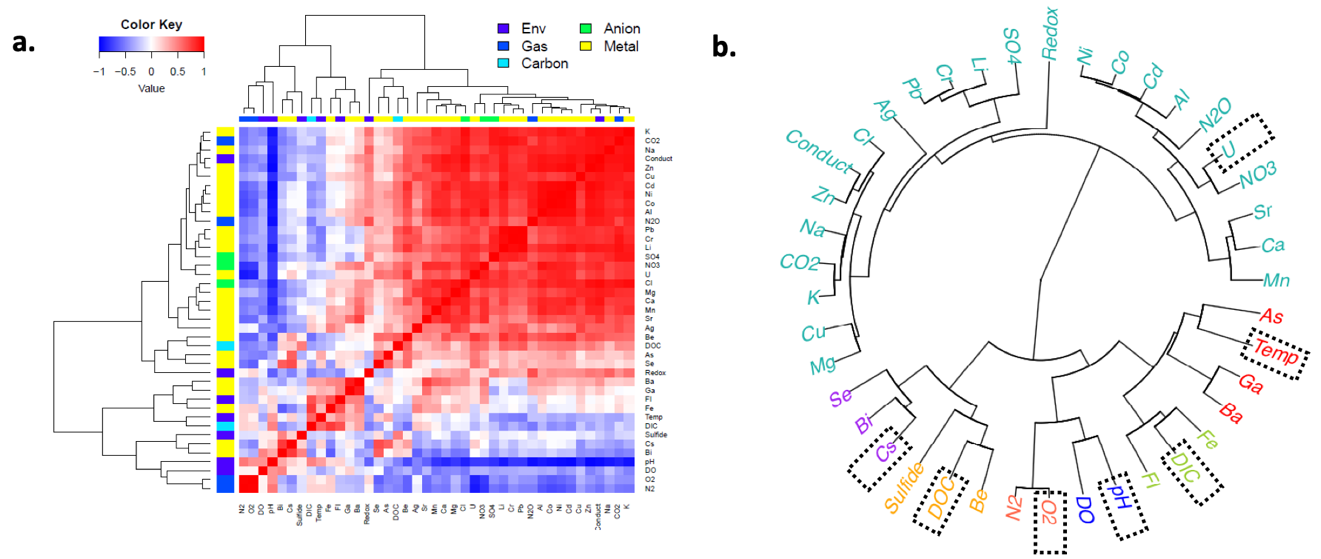

Supplement: FIG S4 [file mSystems.00296-19-sf004.tif]

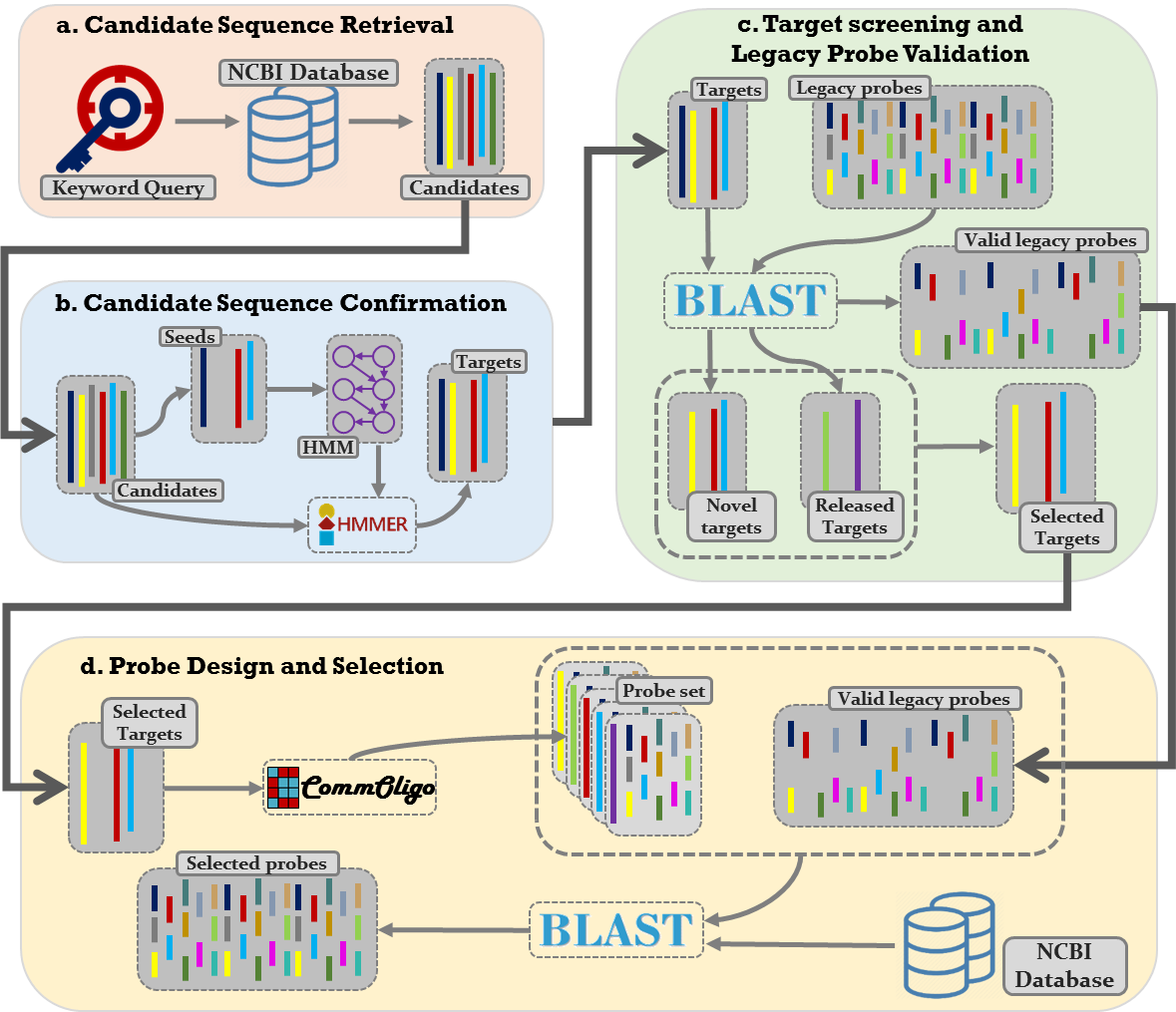

Supplement: FIG S5 [file mSystems.00296-19-sf005.tif]
