# Supplementary material for: Functional Gene Array-Based Ultrasensitive and Quantitative Detection of Microbial Populations in Complex Communities
Source: mSystems. 2019 Jun 18;4(4):e00296-19. doi: 10.1128/mSystems.00296-19 (PMC6581690; doi:10.1128/mSystems.00296-19)
Supplement: TABLE S4 [file mSystems.00296-19-st004.docx]

**Table S4**. Environmental variable measurements from groundwater samples used in the application study. A total of 12 samples were collected from wells representing 4 levels of contamination (L0, L1, L2 and L3). Variables were divided into 5 categories: general environmental parameters (Env. Parameters), gas TCD, dissolved Carbon (C), anion and metal ion.

|  | | **L0** | | | **L1** | | | **L2** | | | | | | **L3** | | | | |
| --- | --- | --- | --- | --- | --- | --- | --- | --- | --- | --- | --- | --- | --- | --- | --- | --- | --- | --- |
|  |  | **GW-654** | **GW-199** | **GW-350** | **FW-300** | **FW-301** | **FW-303** | **FW-215** | | **DP16D** | | **GW-101** | | **FW-106** | | **FW-126** | | **FW-021** |
| **General Env. Parameters** | **Temp. (°C)** | 13.01 | 14.29 | 17.98 | 15.54 | 15.77 | 15.82 | 17.88 | 17.05 | | 18.12 | | 14.84 | | 12.16 | | 16.41 | |
|  | **D.O. (mg/L)** | 2.35 | 0.5 | 0.02 | 0.28 | 0.78 | 0.71 | 0.13 | 0.26 | | 0.96 | | 0.18 | | 0.16 | | 0.27 | |
|  | **Conduct. (μS/cm)** | 269 | 581.6 | 545 | 378.9 | 334.4 | 316.4 | 637.4 | 661.0 | | 1721.0 | | 7864.0 | | 18770 | | 7967 | |
|  | **Redox (mV)** | 175 | 305 | 137 | -129 | 39 | 147 | 43 | -50 | | -72 | | 426 | | 168 | | 387 | |
|  | **pH** | 7.19 | 6.53 | 6.67 | 6.59 | 6.68 | 7.16 | 6.6 | 6.67 | | 6.81 | | 3.55 | | 3.04 | | 3.43 | |
|  | **Sulfide (mg/L)** | 0.003 | 0.003 | 0 | 0.026 | 0.188 | 0.041 | 0 | 0 | | 0 | | 0.004 | | 0.044 | | 0.004 | |
|  | **F.I. (mg/L)** | 0.1 | 0.13 | 0.38 | 0.5 | 0.98 | 0 | 0.21 | 1.46 | | 2.7 | | 1.02 | | 0.13 | | 0.03 | |
| **Gas TCD (µmol/mL)** | **N_2_** | 59.313 | 104.347 | 125.995 | 57.392***** | 45.931 | 68.853 | 31.878 | 30.766 | | 42.717 | | 28.132 | | 14.475 | | 29.89 | |
|  | **O_2_** | 12.429 | 23.330 | 24.629 | 11.256***** | 9.934 | 12.579 | 6.006 | 6.082 | | 8.156 | | 8.331 | | 4.423 | | 6.193 | |
|  | **CO_2_** | 36.69 | 172 | 127.76 | 54.3***** | 60.34 | 48.26 | 173.47 | 201.94 | | 283.42 | | 631.07 | | 718.48 | | 288.99 | |
|  | **N_2_O** | 0 | 0 | 0 | 0 | 0 | 0 | 0 | 0 | | 2.652 | | 22.639 | | 17.022 | | 30.117 | |
| **C (mg/L)** | **D.I.C** | 30.98 | 85.05 | 67.46 | 48.13 | 55.44 | 40.82 | 85.25 | 87.91 | | 115.9 | | 43.18 | | 36.65 | | 22.27 | |
|  | **D.O.C.** | 0.345 | 1.335 | 0.717 | 44.54 | 48.65 | 39.59 | 1.928 | 2.326 | | 4.065 | | 47.87 | | 128.2 | | 7.298 | |
| **Anion**  **(mg/L)** | **Cl** | 1.259 | 5.492 | 13.527 | 2.186 | 3.534 | 3.328 | 15.592 | 23.712 | | 42.244 | | 318.17 | | 373.69 | | 152.313 | |
|  | **NO3** | 0.460 | 0.169 | 0.315***** | 3.66 | 36.357 | 3.962 | 5.503 | 141.022 | | 1470.9 | | 2692.04 | | 11648.3 | | 4506.9 | |
|  | **SO4** | 15.642 | 20.966 | 13.388 | 6.472 | 9.139 | 7.397 | 75.914 | 64.844 | | 7.514 | | 2062.69 | | 1460.19 | | 41.945 | |
| **Metal (mg/L)** | **Ag** | 0.007 | 0.010 | 0.021 | 0.014 | 0.008 | 0.008 | 0.011 | 0.011 | | 0.022 | | 0.022 | | 0.023 | | 0.011 | |
|  | **Al** | 0.015 | 0.013 | 0.038 | 0.03 | 0.418 | 0.017 | 0.013 | 3.444 | | 1.129 | | 108.497 | | 558.765 | | 114.501 | |
|  | **As** | 0.003 | 0.002 | 0.005 | 0.021 | 0.003 | 0.003 | 0.011 | 0.011 | | 0.005 | | 0.008 | | 0.006 | | 0.011 | |
|  | **Ba** | 0.04 | 0.242 | 0.055 | 0.093 | 0.077 | 0.072 | 0.104 | 0.269 | | 2.820 | | 0.077 | | 0.131 | | 2.073 | |
|  | **Be** | 0.04 | 0.019 | 0.038 | 0.082 | 0.04 | 0.04 | 0.041 | 0.041 | | 0.039 | | 0.059 | | 0.149 | | 0.079 | |
|  | **Bi** | 0.038 | 0.001 | 0.005 | 0.018 | 0.038 | 0.038 | 0.009 | 0.009 | | 0.005 | | 0.005 | | 0.005 | | 0.009 | |
|  | **Ca** | 16.619 | 89.37 | 92.374 | 67.26 | 78.81 | 52.516 | 120.122 | 139.769 | | 419.983 | | 273.264 | | 9837.8 | | 3970.12 | |
|  | **Cd** | 0.003 | 0.002 | 0.004 | 0.006 | 0.006 | 0.003 | 0.003 | 0.01 | | 0.007 | | 0.132 | | 0.866 | | 0.173 | |
|  | **Co** | 0.004 | 0.000 | 0.002 | 0.006 | 0.011 | 0.004 | 0.003 | 0.056 | | 0.009 | | 0.509 | | 1.364 | | 1.225 | |
|  | **Cr** | 0.004 | 0.004 | 0.007 | 0.010 | 0.004 | 0.004 | 0.005 | 0.005 | | 0.007 | | 0.384 | | 0.798 | | 0.005 | |
|  | **Cs** | 0.029 | 0.008 | 0.016 | 0.064 | 0.029 | 0.029 | 0.032 | 0.032 | | 0.016 | | 0.017 | | 0.016 | | 0.032 | |
|  | **Cu** | 0.009 | 0.013 | 0.025 | 0.008 | 0.009 | 0.009 | 0.004 | 0.004 | | 0.222 | | 0.810 | | 1.587 | | 0.118 | |
|  | **Fe** | 0.011 | 0.851 | 0.338 | 0.067 | 0.030 | 0.011 | 0.016 | 1.933 | | 4.585 | | 0.038 | | 0.167 | | 0.016 | |
|  | **Ga** | 0.011 | 0.010 | 0.007 | 0.014 | 0.011 | 0.011 | 0.007 | 0.011 | | 0.089 | | 0.007 | | 0.009 | | 0.06 | |
|  | **K** | 0.996 | 3.291 | 3.435 | 1.546 | 2.372 | 1.456 | 5.166 | 5.962 | | 4.857 | | 216.441 | | 102.491 | | 28.827 | |
|  | **Li** | 0.038 | 0.022 | 0.042 | 0.096 | 0.038 | 0.038 | 0.048 | 0.048 | | 0.069 | | 1.946 | | 5.190 | | 0.227 | |
|  | **Mg** | 16.175 | 22.308 | 80.874 | 16.175 | 16.175 | 16.175 | 32.349 | 32.349 | | 80.874 | | 80.874 | | 216.229 | | 117.901 | |
|  | **Mn** | 0.011 | 4.318 | 0.291 | 0.199 | 2.249 | 0.043 | 0.475 | 9.393 | | 8.348 | | 32.498 | | 134.068 | | 128.452 | |
|  | **Na** | 39.949 | 20.806 | 52.015 | 10.403 | 10.403 | 10.403 | 20.806 | 29.262 | | 52.015 | | 865.339 | | 826.325 | | 269.202 | |
|  | **Ni** | 0.006 | 0 | 0.011 | 0.043 | 0.048 | 0.006 | 0.021 | 0.242 | | 0.026 | | 7.184 | | 15.352 | | 5.339 | |
|  | **Pb** | 0.003 | 0.002 | 0.003 | 0.006 | 0.003 | 0.003 | 0.003 | 0.003 | | 0.003 | | 0.032 | | 0.060 | | 0.004 | |
|  | **Se** | 0.009 | 0 | 0.004 | 0.018 | 0.009 | 0.009 | 0.009 | 0.009 | | 0.004 | | 0.024 | | 0.005 | | 0.013 | |
|  | **Sr** | 0.096 | 0.212 | 0.119 | 0.115 | 0.12 | 0.168 | 0.399 | 0.372 | | 2.219 | | 0.369 | | 2.43 | | 1.373 | |
|  | **U** | 0.051 | 0.003 | 0.006 | 0.216 | 0.16 | 0.081 | 1.452 | 0.744 | | 0.417 | | 16.625 | | 55.286 | | 3.751 | |
|  | **Zn** | 0.02 | 0.059 | 0.084 | 0.051 | 0.058 | 0.041 | 0.040 | 0.078 | | 0.093 | | 1.099 | | 2.189 | | 0.897 | |

* missing values were imputed using the mean of values from other two replicates in the same group.
