# Supplementary material for: Functional Gene Array-Based Ultrasensitive and Quantitative Detection of Microbial Populations in Complex Communities
Source: mSystems. 2019 Jun 18;4(4):e00296-19. doi: 10.1128/mSystems.00296-19 (PMC6581690; doi:10.1128/mSystems.00296-19)
Supplement: TEXT S1 [file mSystems.00296-19-s0001.pdf]

Supplementary text for Functional Gene Array-based Ultra-sensitive and -Quantitative Detection of Microbial Populations in complex communities

Zhou Shi[1], Huaqun Yin[2], Joy D. Van Nostrand[1], James W. Voordeckers[1], Qichao Tu[3], Ye Deng[3], Menting Yuan[1], Aifen Zhou[1], Ping Zhang[1], Naijia Xiao[1], Daliang Ning[1], Zhili He[4], Liyou Wu[1], and Jizhong Zhou[1,5,6]

[1]Institute for Environmental Genomic and Dept. Microbiology and Plant Biology, University of Oklahoma, Norman, OK, USA. [2]Key Laboratory of Biometallurgy of Ministry of Education, School of Minerals Processing and Bioengineering, Central South University, Changsha, China. [3]Institute of Marine Science and Technology, Shandong University, Qingdao, China. [4]School of Environmental Science and Engineering, Environmental Microbiomics Research Center, Sun Yat-sen University, Guangzhou, China. [5]Earth and Environmental Sciences, Lawrence Berkeley National Laboratory, Berkeley, CA, USA. [6]School of Environment, Tsinghua University, Beijing, China.

MATERIALS AND METHODS

Sequence retrieval and probe design. Sequence retrieval and probe design for the GeoChip 5.0 were performed using the GeoChip design pipeline as described previously (1, 2). Briefly, a keyword query for each protein-encoding gene of interest (see Supplementary text for a detailed description of the target genes) was submitted to the NCBI nr database to retrieve candidate sequences (Fig. S1). All sequences were downloaded from the NCBI databases before July 29, 2013. Next, protein/enzyme sequences that had been experimentally confirmed were selected as seed sequences to build a Hidden Markov Model (HMM), which was then used to search the retrieved sequences and confirm functions. Confirmed sequences were considered to be potential targets for probe design.

To maintain consistency between GeoChip versions and minimize the number of probes that needed to be designed, legacy probes from previous versions of GeoChip that were still valid were included on GeoChip 5. The legacy probes were searched against the entire confirmed target sequence database to determine if any of the confirmed sequences were covered by legacy probes. If a confirmed sequence was covered by a legacy probe, that sequence was immediately

assigned to the corresponding legacy probe and excluded from further probe design steps. If a legacy probe showed potential cross hybridization to a non-target sequence, it was voided and removed from the probe collection and the corresponding target sequences were released and reused for probe design.

Probe design was performed using a new version of the CommOligo software (3). Two types of probes were designed: gene-specific (each probe targets one gene sequence); and group-specific (one probe targets two or more highly homologous sequences) (1). The newly designed candidate probes and legacy probes were searched against the NCBI nt/env_nt databases to verify specificity based on experimentally determined sequence similarity (≤ 90%), continuous stretch (≤ 20 bases) and free energy (≥ -35 cal/mol) criteria (1). Potentially non-specific probes were removed from further consideration. Multiple probes targeting the same sequence or group of sequences were designed, so CommOligo was used to rank the remaining probes (3), and only top ranked probes were used for array construction.

Microarray construction. Two major formats of the GeoChip 5.0 array were developed. The smaller format (GeoChip 5.0S) has ~60,000 probes per array (See Table S1 for details). For delineating experimental parameters, several modified versions of GeoChip 5.0S were constructed that included perfect match (PM) and mismatch (MM) probes from different pure cultures. The larger format (GeoChip 5.0M) has ~180,000 probes per array (Table S1). All GeoChip 5.0 microarrays were manufactured by Agilent (Santa Clara, CA, USA) using either the 8x60K (8 arrays per slide) or the 4x180K (4 arrays per slide) format. Each GeoChip array was evenly divided into 96 (8×12 grids) subarrays for 5.0S, and 256 (8×32 grids) subarrays for 5.0M. Each subarray has sixteen 16S control probes and five common oligonucleotide reference standard probes (CORS) (4) at specific positions. The 16S control probes were split into two groups of 8 and were placed at the top and bottom of each subarray. CORS probes were placed in the central region of each subarray. Additionally, each subarray had 2 or 3 randomly placed Agilent negative control probes. The hyperthermophile control probes and functional gene probes were randomly placed across the array in the remaining available spaces.

DNA extraction, purification and quantification. Genomic DNA from *Desulfovibrio vulgaris* Hildenborough (DvH) and *Clostridium cellulolyticum* H10 (H10) were extracted using a GenElute Bacterial Genomic DNA Kit (Sigma-Aldrich) following the manufacturer's instructions. To evaluate GeoChip 5.0 performance, previously collected samples were used: soil from the BioCON experimental site (5), groundwater from the Department of Energy's Oak Ridge Integrated Field Research Center (OR-IFRC; Oak Ridge, TN, USA) (6, 7), and mixed liquor of wastewater and activated sludge from the aeration tank at the Norman Water Reclamation Facility (Norman, OK, USA). Soil (5 g soil) and groundwater (4-6 l) were extracted using freeze-grinding mechanical lysis (8). The soil community DNAs were then purified using a low-melting agarose gel followed by phenol extraction (8). Groundwater DNAs did not require further purification. The wastewater samples were centrifuged at 15,000 × g for 10 min to get pellets, and DNA was extracted using a PowerSoil DNA Isolation kit (Qiagen, Germantown, MD).

DNA quality was assessed by absorbance ratios (A260/A280 and A260/A230) using a NanoDrop ND-1000 spectrophotometer (NanoDrop Technologies, Wilmington, DE), and final DNA concentrations were quantified with PicoGreen (9) using a FLUOstar Optima microplate reader (BMG Labtech, Jena, Germany).

Target DNA preparation, amplification and labeling. Since very low amounts of community DNAs were obtained from groundwater, whole community genome amplification (WCGA) was required (10). Aliquots of DNA were amplified using the Templiphi kit (GE Healthcare) and a modified reaction buffer containing 0.1 mM spermidine and 267 ng ml^{-1} single stranded binding protein to improve the amplification efficiency (10). For samples with measurable DNA (by PicoGreen), 5-10 ng was used. For those samples without measurable DNA, the samples were concentrated to a volume of 10 µl and 2-5 µl was used for amplification (initial amplification was attempted with 5 µl and then reduced if unsuccessful). Samples were amplified for 6 hrs. All the amplified DNAs (~2 ug) were used for subsequent labeling.

DNA (amplified or unamplified) was mixed with 5.5 µl random primers (Life Technologies, random hexamers, 3 µg/µl), brought to 35 µl with nuclease-free water, heated to 99 °C for 5 min, and immediately placed on ice. Labelling master mix (15 µl), including 2.5 µl of dNTP (5 mM dAGC-TP, 2.5 mM dTTP), 0.5 µl of Cy-3 dUTP (25 nM; GE Healthcare), 1 µl of Klenow (imer; San Diego, CA; 40 U ml^{-1}), 5 µl Klenow buffer, and 2.5 µl of water, was added

and the samples were incubated at 37°C for 6 h in a thermocycler and then at 95°C for 3 min to inactivate the enzyme. After the addition of Cy3, samples were protected from the light as much as possible. Labelled DNA was cleaned using a QIAquick purification kit (Qiagen) per the manufacturer's instructions and then dried down in a SpeedVac (45°C, 45 min; ThermoSavant).

GeoChip hybridization. GeoChip 5.0S and 5.0M use different volumes of hybridization buffer. The volumes below are for GeoChip 5.0M, volumes for 5.0S are in parentheses. Standard hybridization conditions were followed unless otherwise indicated.

Labeled DNA was resuspended into 27.5 µl (11.9 µl) of DNase/RNase-free distilled water, and then mixed completely with 99.4 µl (43.1 µl) of hybridization solution containing 63.5 µl (27.5 µl) of 2×HI-RPM hybridization buffer, 12.7 µl (5.5 µl) of 10×aCGH blocking agent, formamide (10% final concentration), 0.05 µg/µl Cot-1 DNA, and 10 pM CORS (4). The solution was denatured at 95 ^{o}C for 3 min, and then incubated at 37 ^{o}C for 30 min. The DNA solution was centrifuged briefly (1 min, 6000 × g) to collect liquid at the bottom of the tube and then 110 µl (48 µl) of the solution was pipetted into the center of the well of the gasket slide (Agilent). The array slide was placed on the gasket slide, array side down, sealed using a SureHyb chamber, placed into the hybridization oven, and hybridized at 67 ^{o}C for 24 h.

After hybridization, slides were disassembled in room temperature Wash Buffer 1 (Agilent), then transferred to fresh room temperature Wash Buffer 1 on a magnetic stir plate set at 200 rpm and incubated for 5 min. The slides were then incubated at 37°C in Wash Buffer 2 (Agilent) for 1 min on a magnetic stir plate set at 140 rpm. Slides were then slowly removed from the buffer. The slide's hydrophobic coating allowed the slide to shed the buffer and dry almost immediately.

Microarray imaging and signal processing. The slides were imaged as a Multi-TIFF with a NimbleGen MS200 Microarray Scanner (Roche NimbleGen, Madison, WI, USA). The data was extracted using the Agilent Feature Extraction program, v11.5. Extracted data was then loaded onto the GeoChip data analysis pipeline (http://www.ou.edu/ieg/tools/data-analysis-pipeline.html).

Probe quality was assessed, and poor or low signal probes were removed. Probe spots with coefficient of variance (CV; probe signal SD/signal) >0.8 were removed. Then the signal-to-noise

ratio [SNR; (probe signal-background)/background SD] was calculated. As suggested by Agilent, the average signal of Agilent's negative control probes within each subarray was used as the background signal for the probes in that subarray instead of the local background typically used. If all negative control probes within a given sub-array failed to yield a valid signal, the mean background signal intensity from an adjacent sub-array was used instead. The signal intensity for each spot was corrected by subtracting the background signal intensity. If the net difference was <0, the spots were excluded from subsequent analysis.

A two-step data normalization and quality filtering method was performed for all arrays in an experiment (4, 11). First, the average signal intensity of CORS was calculated for each subarray, and the maximum average value among all subarrays was applied to normalize the signal intensity of samples in each array. Second, the sum of the signal intensity was calculated for each array, and the maximum sum value was applied to normalize the signal intensity of all spots in each array, which produced a normalized value for each spot in each array. Detailed descriptions of the optimized GeoChip sample preparation, hybridization, imaging and normalization methods and reagents and equipment needed is in (12).

Shotgun metagenomic sequencing. Libraries were constructed using a KAPA HyperPlus Prep kit (Roche, Pleasanton, CA). Shotgun sequencing was performed at the Oklahoma Medical Research Foundation on two lanes of a HiSeq 3000 (Illumina, San Diego, CA) using a 2×150 bp sequencing kit).

Statistical analysis. Various statistical methods were used for analyzing the GeoChip data. Three different nonparametric multivariate analysis methods, ADONIS (permutational multivariate analysis of variance using distance matrices), ANOSIM (analysis of similarities) and MRPP (multi-response permutation procedure), and detrended correspondence analysis (DCA), were used to measure the overall differences of community functional gene structure (13). The microbial community functional gene diversity was estimated using Shannon Index, Simpson Index and functional gene richness. Pearson correlation coefficient was used for testing the dependence among environmental factors. A dendrogram tree of environmental factors was constructed based on Manhattan distances using Ward's minimum variance method (14), and was cut into hierarchical clusters using the cutree method (15). Canonical correspondence analysis

(CCA) and partial CCA were used for analyzing statistical linkages between the functional gene structure and environmental variables. Variation partitioning analysis (VPA) was used to divide and assign the variance in microbial functional gene structure among samples. A forward selection procedure was used to build a stepwise CCA model (16). Briefly, at each step the model is extended by adding an additional variable to maximize the explanatory power of the model. The procedure is automatically terminated when (i) the explanatory power of the model starts to decrease, (ii) the permutation test fails after a variable is added or (iii) all variables are included. Welch's *t*-test was used to test the significance of differences in functional gene richness and alpha diversity between paired groups of samples without assuming unequal variances. All statistical analyses were performed in R (version 3.4.4, 2018-03-15) using packages stats, ape and vegan.

SELECTION OF GENE FAMILIES FOR GEOCHIP 5.0 FABRICATION

All functional gene families from previous GeoChips (a total of 410) were updated. Detailed rationale for selecting these gene families and categories was previously provided (1, 2, 17-19). All gene families from previous versions (all genes submitted prior to June 2009) were manually updated. The keyword queries, alignment cutoff values, and seed sequences were modified as necessary to increase sequence coverage and accuracy based on the NCBI databases available in July 29, 2013. During this update, some gene families were combined or separated based on newly discovered gene families or increased sequence availability. For example, twelve dioxygenase gene families from GeoChip 4 were combined into three gene families due to similarities in the sequences of these families; *norB* was spilt into two gene families to differentiate a new subgroup discovered after design of GeoChip 4. GeoChip 5.0 greatly expanded overall gene and sequence coverage by adding more than 1,000 new gene families and covered a total of 1,447 gene families involved in carbon (135 genes), nitrogen (28 genes), sulfur (27 genes), and phosphorus (7 genes) cycling, antibiotic resistance (19 genes), stress response (103 genes), microbial defense (65 genes), metabolic pathways (4 genes), plant growth viruses (115 genes), virulence (605 genes), metal homeostasis (119 genes), organic contaminant degradation (105 genes), pigments (30 genes) and electron transfer (11 genes) (Table 1). Detailed descriptions for all selected functional gene families is provided below and Table S1.

Several of the updated and expanded categories have been discussed in detail in other publications, including virulence genes (20) and stress response (21, 22) genes. In addition, microbial defense, plant growth promotion, pigments and protist phylogenetic markers were added to increase coverage of functional processes of importance to various ecological and environmental processes. Here we provide an overview of these gene families.

Categories for geochemical cycling

Microorganisms play key roles in geochemical cycling, including carbon, nitrogen, phosphorous, sulfur. Genes for these cycles were included on GeoChip 5. Many of these genes were also on versions 3 and 4 but have been greatly expanded and refined in this updated version.

Carbon cycling

Genes in the carbon cycling category include those for carbon degradation, carbon fixation, and methane cycling (methane oxidation and methanogenesis).

<u>Carbon degradation.</u>

Microorganisms are capable of degrading a wide variety of carbon sources. To cover some of that diversity, the number of carbon degradation genes covered on GeoChip 5.0 was greatly expanded. GeoChip versions 2-4 included genes for degradation of cellulose, lignin, chitin, starch, hemicellulose, and pectin (1, 11, 23). In addition to those genes, genes for degradation of terpenes and genes for the glyoxylate cycle were included.

Many of these carbon substrates are derived from plants. Cellulose, hemicellulose, and lignin are all components of the plant cell wall. Cellulose is the primary component of cell walls in green plants, many types of algae, oomycetes and some bacteria. A number of enzymes can be involved in cellulose degradation including cellobiase, cellobiohydrolase/exoglucanase, and cellulose/endoglucanase (24, 25). Hemicellulose is composed of a wide variety of compounds including xylan, the most abundant fraction of hemicellulose and second most abundant polysaccharide in nature (26), mannan/heteromannan, and xylose. Xylan can be degraded by endo-1,4-beta-xylanase (27), mannan by beta-mannanase/mannanase (28), and xylose by xylose isomerase(29). Lignin is an important part of plant, and some algal, cell walls. It is extremely complex in structure and recalcitrant to degradation. Enzymes including glyoxal oxidase (glx), manganese peroxidase (mnp), and phenol oxidase are involved in the degradation of lignin (30-32). Vanillin and related compounds are used to model degradation products from lignin(33, 34) , so the enzymes vanillate demethylase, vanillate monooxygenase(35, 36), and vanillin dehydrogenase (vdh) (37) were also included.

Pectin is a complex mixture of plant polysaccharides (38). Enzymes involved in degrading pectin include pectate lyase (39), pectin methylesterase (pme) (40), rhamnogalacturonan acetylesterase (41), rhamnogalacturonan hydrolase (42), and rhamnogalacturonan lyase (43). Arabinose is found in a number of plant polysaccharides as part of side chains. Arabinose is degraded by

arabinofuranosidase. Due to the number of sequences available, arabinofuranosidase has been split into bacterial-archaeal and fungal gene sets (44, 45).

Chitin is a polysaccharide present in many organisms including fungi, crustaceans, and insects. The two original genes (acetylglucosaminidase and chitinase) involved in chitin degradation were updated and chitinase was split into 3 sub-categories based on where the enzyme attacks (endo-, exo-, or undefined). Chitinases are found in any number of organisms including viruses (46).

Starch, glycogen, and related compounds are energy storage polysaccharides used by a number of eukaryotes. A wide variety of enzymes are used to breakdown these compounds: Alpha-amylase, pullulanase type I (*amyX*), gamma-amylase (glucoamylase), pullulanase, amylopullululanase (apu) (47-50), cyclomaltodextrinase (cda), neopullulanase (*nplT*), and isopullulanase (51, 52). Inulin is another polysaccharide that is used by plants for energy storage. Inulinase is currently the only degradative gene for this polysaccharide on GeoChip (53-55).

Terpenes are hydrocarbons that comprise a large class of plant-derived secondary metabolites and are the main component of plant essential oils (56). Various types of essential oils have been shown to have antiviral, antibacterial and antioxidant activity (56, 57). Enzymes for terpene degradation include carveol dehydrogenase, limonene-1,2-epoxide hydrolase (*limEH*), limonene monooxygenase (LMO) (58-63), and *camDCAB* (camphor 5-monooxygenase) (64-67).

The glyoxylate cycle is involved in the conversion of acetyl-CoA to succinate for use in a number of biosynthetic pathways and may also play an important role in bacterial and fungal virulence. The key enzymes are isocitrate lyase (AceA) and malate synthase (AceB) (68, 69).

<u>CO_2 fixation</u>.

Carbon fixation converts inorganic carbon (CO_2) into organic carbon that can be used by other organisms. Autotrophic CO_2 fixation is “the most important biosynthetic process in nature” (70). There are now six known pathways for autotrophic CO_2 fixation (71). When earlier versions of GeoChip were designed, only five pathways were known (72), and a single enzyme from each of four of these pathways were covered. Here we sought to increase coverage by including

additional enzymes from each cycle (total number of genes indicated in parentheses): Calvin cycle (9 genes) (73, 74), 3-hydroxypropionate bicycle (10 genes) (75), reductive acetyl–CoA pathway (2 genes) (76), reductive tricarboxylic acid cycle (8 genes) (77), and two new pathways: dicarboxylate/4-hydroxybutyrate cycle (12 genes) (78) and 3-hydroxypropionate/4-hydroxybutyrate cycle (12 genes) (79). Only the reductive acetyl-CoA pathway did not have genes added as many of the enzymes in this particular cycle have dual functions in other normal cellular processes.

In addition to enzymes, genes for carboxysomes, bacterial microcompartments that aid in the concentration of CO_2 (80-82), were also selected. The seven carboxysome genes cover shell proteins, which act as a CO_2 diffusion barrier, concentration mechanisms and carbonic anhydrase isoforms. One specific protein for each of the alpha and beta carboxysomes was also included (83).

<u>Methane metabolism</u>:

Methane accounts for about 10% of all greenhouse gas emissions and is primarily produced during decomposition of organic matter (84). Methanogenesis is the process by which single-carbon substrates are reduced to produce methane and generate energy. This methane can in turn be oxidized to create CO_2 and O_2. There are four methanogenesis pathways, the core pathway, acetoclastic, hydrogenotrophic, and methyl-corrinoid. The core pathway is the final reduction step to produce methane via methyl-coenzyme M. The hydrogenotrophic pathway reduces CO_2 to CO and then formate, which then feeds into the core pathway. The acetoclastic pathway reduces acetate to acetyl CoA. The acetyl CoA is then reduced in the final step of the hydrogenotrophic pathway. The methyl-corrinoid pathway reduces substrates containing methyl groups and then feeds into the core pathway. Previously, only methyl-coenzyme M reductase (*mcrA*) was covered as an indicator for methanogenesis. Additional genes were added to cover the acetoclastic (3 genes), hydrogenotrophic (6 genes), and methyl-corrinoid (3 genes) pathways (85). Particulate and soluble methane monooxygenases, *pmoA* and *mmoX*, are included for methane oxidation.

Nitrogen Cycling

While N is critical to all living things, the largest N pool, N_2, is extremely stable and requires a great deal of energy to reduce it to a state that is biologically available. In addition, most of the nitrogen in soil is biologically unavailable. While some nitrogen is available from minerals in the soil, nitrogen availability is largely controlled by microbial activity. Nitrogen-fixing bacteria convert atmospheric N_2 into NH_4 allowing it to be used by plants and other bacteria. Plant and animal decay releases NH_3 via ammonification. The NH_3/NH_4 can be converted to NO_2 then NO_3 via nitrification; NO_3 is converted back to N_2 via denitrification. NO_3 can also be reduced to NH_2 via N reduction. NH_4 and NO_2 can be oxidized to N_2 anaerobic ammonium oxidation (anammox). Each of these processes is covered on the GeoChip: N fixation (1 genes), nitrification (4 genes), denitrification (6 genes), ammonification (4 genes), dissimilatory (2 genes) and assimilatory (5 genes) N reduction to NH_4, and anammox (2 genes) (2, 86).

Nitrogen cycling genes that are new to GeoChip 5 include the nitrification gene, nitrite oxidoreductase (*nxrA*) (87) and hydrazine synthase (*hzsA*) involved in the anammox system (88). Two types of nitrous oxide reductase genes were included: *cnorB* a cytochrome bc complex type enzyme and *qnorB*, a quinol-oxidizing single-subunit class (89). An assimilatory nitrate reductase (*narB*) from cyanobacteria was added as well. This gene is similar to *nasA*, but has a different nomenclature (90-95).

Additionally, 3 new genes for nitrogen assimilation by bacteria and fungi, including ammonium, nitrate and nitrite transporters were added.

Phosphorus cycling

Phosphorus plays an important role in biological life as a component of cellular components such as nucleotides, ATP and membranes. Excess phosphate (Pi) is frequently stored by all living organisms as polyphosphate chains that can contain hundreds of Pi residues. Polyphosphate has numerous functions from Pi storage, stress response, virulence, and maintenance of stationary phase (96). Two genes for polyphosphate biosynthesis (*ppk* and phytase) and one for polyphosphate degradation (exopolyphosphatase/ppx) were included on earlier versions of

GeoChip. Polyphosphate kinase/*ppk* removes a Pi from ATP to lengthen poly P. Phytase is involved in the conversion of organic phosphorous to inorganic by hydrolyzing phytate.

Additional genes for oxidation of inorganic phosphate compounds found in the environment were added including hypophosphite dioxygenase (*htxA*), which oxidizes hypophosphite (97) and phosphonate dehydrogenase (*ptxD*), which oxidizes phosphonate (98, 99). Two new genes for polyphosphate degradation were also added. These include a second form of polyphosphate kinase (*ppk2*) that creates either GTP from GDP or ADP from AMP (100, 101) and an endopolyphosphatase (ppn) that cleaves poly-P into various sized units.

Sulfur cycling

Sulfur is the ancient "motor of life" and played a similar role in ancient Earth as O_2 plays now (102). In the current primarily aerobic environment, sulfur acts as both an electron acceptor and donor for anaerobic respiration and can be oxidized (102). The sulfate reduction/sulfide oxidation cycle is found in a variety of environments, such as freshwater and marine sediment or microbial mats, and the O_2-H_2S interface formed in this cycling often moves based on factors such as tides or amount of sunlight present (102, 103). Previous versions of GeoChip included *dsrA* and *dsrB* for sulfite reduction, sox for sulfate oxidation, and three genes for adenylylsulfate reductase (2 for *aprA* and *aprB*). Additional genes involved in sulfur transformation reactions were included on this newest version.

Dimethylsulfoniopropionate (DMSP), a major source of C and S in marine environments, is degraded by either cleavage by DMSP lyase or demethylated by DMSP demethylase (dmdA) (104). Cleavage of DMSP produces dimethylsulfide (DMS), a volatile compound which, when transported to the atmosphere and oxidized, can modulate the formation of clouds (105, 106). Demethylation of DMSP ultimately leads to release of additional C that can be further utilized by marine bacteria (104). The sox "gene" of previous GeoChips was split into five "active" component subunits: *soxA*, *soxB*, *soxC*, *soxV*, and *soxY* (107). *CysI* and *J* encode a sulfite reductase in the cysteine biosynthesis pathway (108).

Categories related to microbial response to environmental conditions

Metal Homeostasis:

High concentrations of metals can be toxic to microorganisms. This type of contamination is common due to both anthropogenic and natural causes (109). To limit exposure and protect against damage from these metals, microorganisms have developed resistance mechanisms (110, 111). Previous versions of GeoChip covered 44 genes/enzymes for resistance to 13 commonly detected metals with well-studies resistance mechanisms (Ag, Al, As, Cd, Co, Cr, Cu, Hg, Ni, Pb, Se, Te and Zn) (110-112) and one gene for bacterial metallothioneins and metallothionein-like proteins (smtA).

The metal resistance category has been expanded to include additional resistance, metal uptake and maintenance mechanisms (72 additional genes) in addition to metal resistance and has been renamed metal homeostasis. Metal acquisition genes included those for ion pumps for several metals including boron (2 genes), calcium (2 genes), magnesium (3 genes), manganese (2 genes), potassium (6 genes), and sodium (8 genes). A number of genes related to iron scavenging, such as transporters, siderophores or siderophore receptors, storage, and oxidation (11 genes) were also included. Metal resistance gene families were updated to include transport and enzymatic transformation genes for resistance to arsenic (6 genes), copper (10 genes), mercury (9 genes), and chromium (2 genes). Nutrient metals can also be toxic at higher concentration, so uptake and efflux transporters were included for metals such as nickel (7 genes), cobalt (5 genes), copper (10 genes) and zinc (14 genes). A majority of the genes in this category are transporters, which is the most common metal resistance mechanism for bacteria (112). In addition, transports are involved in uptake of nutrient metals. Other mechanisms of metal resistance are enzymatic medication of the toxic metal or sequestration, so the metal is no longer biologically available.

New genes include arsenic related genes *arrA* (respiratory arsenate reductase) (113) and *arxA* (anaerobic arsenite oxidase) (114), boron related genes *bor1* (boron transporter) (115), *atr1* (boron exporter from fungi) (116), calcium (*chaA*, calcium/proton antiporter) (117), cobalt/magnesium (*corA*) (118), and cobalt/nickel (*nreB*, MFS family protein) (119).

Several genes involved in iron uptake, storage, scavenging, and oxidation were added, including those for iron receptors [*fecA* (120), *feoB* (121), *fepA* (122), *fhuA* (123), *fhuE* (124), *fiu* (125), *cirA* (126)], siderophore biosynthesis [*entB* (127)], internal iron storage [bacterioferritin (*bfr*) (128), *dps* (129)], and iron oxidation [rustocyanin (130)]. Siderophores, high-affinity iron-chelating compounds, are produced when iron is limited (131, 132).

Genes for magnesium transporters include *mgtA* (133) and *mgtE* (134). Genes for manganese include the transporters *psaA/mntA* (135) and *mntH*/Nramps (136). New nickel genes included the periplasmic nickel-binding protein *nikA* (137), a nickel transporter permease *nikC* (138), and a high-affinity nickel-transport protein NiCoT (*nixA* and *hoxN*) (139, 140). In addition, vacuolar Ca exchangers were included. These exchangers allow growth in environments with elevated Ca (141).

New potassium genes include uptake proteins *trkA* (142), *ktrBD* (143), *Kup/trkD* (144), and *trkGH* (145), the P-type ATPase transporter *kdpA* (146) and the efflux transporter *kefBC* (147).

New genes for sodium include the sodium/proton antiporters *nhaA* (148), *nhaB* (149), *nhaC* (150), *nhaD* (151), *nhaP* (152), and *mrpA*-like subunits from several different systems (*mrpA*, *shaA*, *phaA*, *mnhA*, *snoA*) (153-155). In addition, a Na+-translocating NADH:ubiquinone oxidoreductase *nrqB* (156), and the permease *natB* (157) were included.

New zinc genes include the high-affinity transporters *znuA* and *znuC* (158); zinc-binding proteins *troA* (159) and *adcA* (160); transporters *Zrt1*, *Zrt2* (161), *Zrt3* (162), *zupT/ygiE* (163), *cot1* (164) and *msc2* (165); efflux transporters *yiiP/fieF* (166), and the zinc/cadmium resistance protein *zrc1* (167).

<u>Organic Contaminant Degradation</u>

Several major changes were made to this group of genes from earlier GeoChip versions. First, a number of old genes were removed as they no longer give useful information for various reasons such as crossover with other non-target genes that could not be resolved by HMMER or being so far down a degradation pathway(s) that they were involved in reactions that could be considered general cell metabolism. Second, several target genes were combined due to sequence similarity,

which meant they could not be fully separated. Several genes were split due to enantiomer selectivity and other genes that were composed of 2 or more subunits were reduced to a single subunit. Several new genes were also added.

Genes will be listed below by chemical with the reason for that chemical being chosen. Several of the genes can be utilized in multiple degradation pathways as they are further "downstream" but will only be listed here one time.

2-Aminobenzenesulfonate is used in the manufacture of dyes and pharmaceuticals. It can be degraded by catechol_B (catechol 2,3-dioxygenase) (168).

2-, 3-, & 4- Chlorobenzoate is an intermediate in dye manufacturing and is degraded by *cbeA* (chlorobenzoate 1,2-dioxygenase) (169).

2,4-Dichlorophenoxyacetic Acid (2,4-D) is a common herbicide and is degraded by *tfdA* (2,4-D/alpha-ketoglutarate dioxygenase) (170).

2,4-Dichlorophenol is an intermediate in the production of 2,4-D and is degraded by *tfdB* (2,4-dichlorophenol 6-monooxygenase) (171).

2,4,5-trichlorophenoxyacetic acid is a synthetic auxin and herbicide used from the 1940s through the 1980s. It is very toxic due to the presence of trace contaminants, introduced during manufacturing. It is degraded by *tftA* (2,4,5-trichlorophenoxyacetic acid oxygenase) and *tftH* (hydroxyquinol 1,2-dioxygenase) (172).

3-Chloroacrylic acid is a pesticide metabolite and is degraded by caad (trans-3-chloroacrylic acid dehalogenase), cis-caaD (cis-3-chloroacrylic acid dehalogenase) (173), and MSAD (malonate semialdehyde decarboxylase) (174).

3-Chlorobenzoate is an intermediate in dye manufacturing and is degraded by *cbaA* (3-chlorobenzoate-3,4-dioxygenase) (175).

4-Aminobenzenesulfonate is used in the manufacture of dyes and pharmaceuticals and is degraded by *pcaG* (protocatechuate 3,4-dioxygenase) (176) and *pcaB* (carboxymuconate cycloisomerase) (177).

4-Chlorobenzoate is an intermediate in the production of various organic chemicals including dyes and fungicides, and as a preservative. It is degraded by *fcbA* (4-chlorobenzoyl ligase) (178) and *fcbB* (4-CBA-CoA dehalogenase) and *fcbC* (chlorobenzoate thioesterase) (178).

Acetylene is a basic building block for a number of chemical processes and is degraded by Xamo (alkene monooxygenase) (179).

Acrylonitrile is used in production of plastics and is degraded by NHase (nitrile hydratase) (180) and ALN (aliphatic nitrilase) (181).

Saturated hydrocarbons (alkanes) are one of the main components of crude oil. Degradation of these compounds is important in bioremediation and in the ecology at natural oil seeps. Alkanes are degraded by alkylsuccinate synthase (*AssA*) (182).

Aniline is used in the manufacture of many products but mainly for polyurethane and is degraded by *tdnQ* (aniline dioxygenase) (183).

Anthracene is a natural product in coal tar and used in dye production and is degraded by *nahB* (naphthalene dihydrodiol) (184).

Atrazine is a widely used pesticide in US agriculture and is degraded by *atzA* (atrazine chlorohydrolase), *atzB* (hydroxyatrazine hydrolase), *atzC* (N-isopropylammelide isopropylaminohydrolase), *atzD* (cyanuric acid amidohydrolase), *trzN* (atrazine hydrolase), and *trzA* (triazine hydrolase) (185-188).

Benzaldehyde is a downstream product of a number of xenobiotic degradation pathways and is degraded by *xylC* (4-hydroxybenzaldehyde dehydrogenase) (189).

Benzoate is the downstream product of a number of xenobiotic degradation pathways and is degraded by BpH (benzoate-para-hydroxylase) (190), *benD* (2-hydro-1,2-dihydroxybenzoate

(DHB) dehydrogenase) (191), bco (benzoyl CoA reductase) (192), *bclA* (benzoate-coenzyme A ligase) (193), *badK* (cyclohex-1-ene-1-carboxyl-CoA hydratase), and *badI* (2-ketocyclohexane-1-carboxyl-CoA hydrolase) (194, 195).

Benzonitrile is a common solvent and intermediate in many industrial chemical processes and is degraded by nitrilase (196) and *amiE* (aliphatic amidase) (197).

Biphenyl is found naturally in coal tar, crude oil and natural gas. It is the starting material for PCBs and is used in the production of many other organic compounds. Biphenyl is degraded by *bphB* (2,3-dihydro-2,3-dihydroxybiphenyl dehydrogenase), *bphC* (2,3-dihydroxybiphenyl 1,2-dioxygenase), and *bphD* (2-hydroxy-6-oxo-6-phenylhexa-2,4-dienoate hydrolase) (198-201).

Carbazole is used in the minor amounts for the production of dyes and is produced during incomplete combustion. It is degraded by *carA* (carbazole 1,9a-dioxygenase) (202).

Catechol used in production of pesticides, fragrances and other compounds and is found in small amounts found in nature, but the bulk is man-made. It is degraded by catechol (catechol 1,2-dioxygenase) (203), *xylF* (2-hydroxymuconate semialdehyde hydrolase), *xylJ* (2-oxopent-4-enoate hydratase), and *bphF1* (4-hydroxy-2-oxovalerate aldolase) (204-208).

Chloromethane is produced in minor amounts by phytoplankton. It was once used as a refrigerant but has been discontinued. It is degraded by *cmuA* (isopropylbenzene dioxygenase) (209).

Cyanuric acid is used as part of, or in the manufacture of, bleaches, disinfectants or herbicides. It is also involved in atrazine degradation. It is degraded by *atzE* (biuret hydrolase) (210).

Cyclohexane is a common non-polar solvent and is degraded by BMO (butane monooxygenase), *chnA* (cyclohexanol dehydrogenase), *chnB* (cyclohexanone 1,2-monooxygenase), *chnC* (1-oxa-2-oxocycloheptane lactonase), *chnD* (hydroxyhexanoate dehydrogenase) (211-213), and *chnE* (6-oxohexanoate dehydrogenase) (214).

Cyclopentanol is used to make cyclopentanone, an important compound in fragrance and pharmaceutical manufacturing. It is degraded by *cpnA* (cyclopentanol dehydrogenase), *cpnB*

(cyclopentanone monooxygenase) (215, 216), *cpnC* (5-valerolactone hydrolase), and *cpnE* (5-oxovalerate dehydrogenase) (217)

Cymene is a natural compound in essential oils and is degraded by cymA (p-cymene methyl hydroxylase), BADH (benzylalcohol dehydrogenase), *cymC* (aryl-aldehyde dehydrogenase), *cmtAb* (p-cumate dioxygenase), *cmtC* (2,3-dihydroxy-p-cumate dioxygenase), *cmtD* (HCOMODA decarboxylase), and *cmtE* (HOMODA hydrolase) (218-220).

Dibenzothiophene is found in crude oil and is degraded by *dbtAc* (dibenzothiophene dioxygenase) and *dbtB* (dibenzothiophene dihydrodiol dehydrogenase) (221, 222).

Dibenzo-p-dioxin is produced in small amounts by natural fires. Larger amounts were made industrially for various uses including herbicides and defoliants. It is degraded by *dxnA* (dioxin dioxygenase) and *dbfB* (2,2,3-trihydroxy-biphenyl dioxygenase) (223).

Dichloroethane is used in polyvinyl chloride (PVC) production and is degraded by *linB* (haloalkane dehalogenase), *moxF* (methanol dehydrogenase), and *dehH* (haloacetate dehalogenase) (224-226).

Dichloromethane is a common solvent and used to “glue” some plastics together. It is degraded by *dcmA* (dichloromethane dehalogenase) (227).

Dimethyl sulfoxide is a solvent that can mix with many different organic solvents and with water and is degraded by *dmsA* (dimethyl sulfoxide reductase) (228).

Dodecyl sulfate is used for the production of SDS a common surfactant in cleaning supplies and is degraded by *sdsA* (alkyl sulfatase), *alkJ* (alcohol dehydrogenase), and *alkH* (aldehyde dehydrogenase) (229-231).

Ethylbenzene is an important intermediate in the production of the plastic polystyrene and is degraded by *ebdA* (ethylbenzene dehydrogenase), apc (acetophenone carboxylase), *akbA* (ethylbenzene dioxygenase), Ped (1-phenylethanol dehydrogenase) (232-235) .

Gamma-hexachlorocyclohexane (lindane) is an insecticide widely used in agriculture and also used to treat lice and scabies. It is degraded by *linA* (gamma-hexachlorocyclohexane dehydrochlorinase), *linC* (2,5-dichloro-2,5-cyclohexadiene-1,4-diol dehydrogenase), *linD* (2,5-dichlorohydroquinone reductive dechlorinase) (236).

Gallate is a natural coumpound found in a number of plants and is used to manufacture pharmaceuticals. Gallic acid was used in the manufacture of inks. It is degraded by *athL* (pyrogallol hydroxyltransferase) (237).

Glyphosate, also known as the herbicide “RoundUp” and has been in use since the 1970s. It is one of the most highly used herbicides in the world. It is degraded by Phn (carbon-phosphorus lyase) and *mauAB* (methylamine dehydrogenase) (238, 239).

Isopropylbenzene (cumene) is commonly found in crude oil and used as a base for the production of other chemical compounds. It is degraded by *cumB* (dihydroxyisopropylbenzene dehydrogenase), *cumC* (3-isopropylcatechol-2,3-dioxygenase), *cumD* (HOMODA hydrolase) (240).

Hydroxyacetophenone is an intermediate in the breakdown of other compounds such as bisphenol A and is degraded by arylest (arylesterase) (241).

Mandelate is the base chemical for a number of pharmaceuticals and is degraded by *mdlA* (mandelate racemase), *mdlB* (mandelate dehydrogenase), *mdlC* (benzoylformate decarboxylase) (242).

m-cresol is a solvent used to dissolve various polymers and is degraded by *mdlD* (benzaldehyde dehydrogenase (NAD(+)) and xlnD (3-hydroxybenzoate 6-hydroxylase) (242).

Methanesulfonic acid is used as an acid catalyst in a variety of organic solvents and is degraded by MSAMO (methanesulfonic acid monooxygenase) (243).

Methylquinoline is used in dye production and is degraded by qorL (quinoline 2-oxidoreductase), Quinoline (3-methyl-2-oxo-1,2-dihydroquinoline 8-monooxygenase) (244).

MTBE is widely used fuel oxygenate. It has caused widespread ground water contamination and is degraded by alkB (alkane 1-monooxygenase) (245).

Naphthalene is found in coal tar and used as a base compound in various chemical reactions. Main component in mothballs. It is degraded by *nahC* (dihydroxynaphthalene and dioxygenase), *nahD* (2-hydroxychromene-2-carboxylate isomerase) and *nahF* (salicyladehyde dehydrogenase) (246).

Nicotine is a natural alkaloid in several plants, especially tobacco. It is a stimulant in animals and was used as an insecticide. Nicotine is degraded by *ndhC* (nicotine dehydrogenase), 6HDNO ((S)-6-hydroxynicotine oxidase), 6HLNO ((R)-6-hydroxynicotine oxidase) (247).

Nitrobenzene is a basic building block used in a number of chemical reactions and is degraded by *amnB* (2-aminophenol 1,6-dioxygenase), *nbzA* (nitrobenzene nitroreductase), and *nbzB* (hydroxylaminobenzene mutase) (248).

Nitrobenzoate is an intermediate in the breakdown of a number of chemicals and is degraded by nbaC (3-hydroxyanthranilate 3,4-dioxygenase) (249).

Nitroglycerin is a common and widely used explosive (TNT). It is degraded by *xenAB* (nitroglycerin reductase) (250).

Nitrophenol is a precursor in the production of pharmaceutical, herbicide, and pesticide. It is degraded by *nphA* (4-nitrophenol 2-monooxygenase), *mhqA* (hydroquinone 1,2-dioxygenase), and *pcpE* (maleylacetate reductase) (251-254).

Octane is an alkane component of gasoline and is degraded by *alkK* (acyl-CoA synthetase) (255).

Organophosphates are a type of insecticide and are degraded by *adpB* (aryldialkylphosphatase) (256).

Pentachlorophenol (PCP) is a common herbicide, insecticide, fungicide, algaecide, general disinfectant, and used in antifouling paint. It has been in use since the 1930s and is highly toxic and slow to degrade. It is degraded by *pcpB* (pentachlorophenol 4-monooxygenase), *pcpC*

(tetrachlorohydroquinone reductive dehalogenase), and *tcpA* (chlorophenol 4-monooxygenase) (257, 258).

Pentaerythritol tetranitrate (PETN) is a high-power explosive and much more toxic than other explosives like RDX. It is considered a “munitions constituent of great concern” by the DOD. It is degraded by Onr (pentaerythritol tetranitrate reductase) (259).

Naphthalene and phenanthrene are used as base compounds in chemical reactions to make dyes, plastics, pesticides and other compounds. They are degraded by *nahD* (chromene-2-carboxylate isomerase), *phdJ* (carboxybenzalpyruvate hydratase-aldolase), *phdK* (carboxybenzaldehyde dehydrogenase) (260).

Phenoxybenzoate is used in the synthesis of larger chemical compounds and is degraded by *pobA* (p-hydroxybenzoate hydroxylase) and POBMO (phenoxybenzoate monooxygenase) (261, 262).

Phenylacetaldoxime is used as a base chemical for pesticides and has potential applications in cancer treatment. It is degraded by *oxdB* (phenylacetaldoxime dehydratase) (263).

Phenylpropionate is naturally produced during breakdown of plant material and is also a component of synthetic steroids. It is degraded by *hcaE* (3-phenylpropionate dioxygenase), *hcaB* (2,3-dihydroxy-2,3-dihydrophenylpropionate dehydrogenase), *mhpA* (3-(3-hydroxyphenyl)propionate hydroxylase), *mhpB* (3-(2,3-dihydroxyphenyl)propionate 1,2-dioxygenase), and *mhpC* (2-hydroxy-6-ketonona-2,4-dienedoic acid hydrolase) (264, 265).

Phthalate is commonly added to plastics to improve their physical properties but are easily released into the environment. It is degraded by *phtA* (phthalate 4,5-dioxygenase), *phtB* (phthalate 4,5-cis-dihydrodiol dehydrogenase), *ophC* (4,5-dihydroxyphthalate decarboxylase), *tphA* (terephthalate 1,2-dioxygenase), tphb (terephthalate 1,2-cis-dihydrodiol dehydrogenase), and HBH (3-hydroxybenzoate 4-hydroxylase) (266-269).

Pyrene is a natural compound found in coal tar and in combustion products, including those produced by the burning of gasoline. Used in the production of dyes. It is degraded by *nidA* (pyrene dioxygenase) (270)

Reductive dehalogenase (Rd) is involved in the removal of halogen atoms from parent compounds such as PCE and TCE (271, 272).

Salicylate is a downstream product of several pathways including naphthalene, phenanthrene, anthracene, and dibenzofuran. It is degraded by *nahG* (salicylate hydroxylase) and *nagG* (salicylate 5-hydroxylase) (273-275).

Tetrahydrofuran is a solvent and used in the manufacture of a variety of polymers and is degraded by *thmA* (tetrahydrofuran hydroxylase) (276).

Thiocyanate is used in the production of other chemicals and is degraded by *scnC* (thiocyanate hydrolase) (277).

Toluate is a base compound used in the manufacture of various plastics and is degraded by *xylX* (toluate dioxygenase), *xylL* (dihydrocyclohexadiene carboxylate dehydrogenase), and *xylG* (2-hydroxymuconic semialdehyde dehydrogenase) (278, 279).

Toluene is a solvent and compound in the production of various chemical compounds and is degraded by *bbsG* (benzylsuccinyl CoA dehydrogenase), *xylM* (xylene monooxygenase), *tmoA* (toluene-4-monooxygenase), *pchF* (4-cresol dehydrogenase), *tutFDG* (benzylsuccinate synthase), *bbsH* (E-phenylitaconyl-CoA hydratase), and *tomA* (toluene 2-monooxygenase) (280-284).

Xylene (or any other compound that has a cis,cis-muconate intermediate from catechol) is degraded by *catB* (muconate cycloisomerase) (285).

Dioxygenases were combined into three sets due to highly similar sequences. These include one_ring_12DiOx (one-ring dioxygenases) which combined benzene 1,2-dioxygenase, benzoate 1,2-dioxygenase, ortho-halobenozate dioxygenase, toluene dioxygenase, and halobenzoate 1,2-dioxygenase (286-288). Another one-ring dioxygenase (one_ring_23DiOx) combined biphenyl 2,3-dioxygenase and isopropylbenzene dioxygenase (289-291). The third set is multiring dioxygenases (mult_ring_12DiOx), which combined pyrene dioxygenase, PAH dioxygenase, phenanthrene dioxygenase, naphthalene 1,2-diooxygenase, and nitrobenzene 1,2-dioxygenase (292-294) .

Nitroreductases are involved in the reduction of both natural (chloramphenicol) and man-made nitroaromatic compounds. Type I nitroreductases are oxygen-insensitive while Type II are oxygen-sensitive. Genetics of the latter have not been well-studied. Genes involved in the degradation of these compounds include *nfsA* (Type I, Group A) and *nfsB (*Type I, Group B) (295, 296).

Stress Response:

Microorganisms are exposed to a variety of environmental stressors and have developed mechanisms to respond to and minimize the negative effects of the stressor. Covered genes include those for heat and cold shock, osmotic stress, oxidative stress, protein stress, stringent response, general stress response, and oxygen, glucose, phosphate and nitrogen limitation as well as sigma factors (18).

Selected sigma factors that are involved in transcription initiation for stress response genes include the housekeeping sigma factor σ70, σ38 for general stress response and σ32 and σ24 for heat shock (297). The haem-catalase *katE* was also included for general stress response (298) and a stringent response GTPase that maintains low intracellular concentrations of ppGpp (*obgE*) (299-301) (302). The ppGpp acts as a transcriptional regulator during periods of stress (303).

Heat and cold shock proteins were included because microorganisms are often exposed to temperature variations in the environment. Heat shock proteins include *dnaK, grpE, groES,* and *groEL*, molecular chaperones that prevent or correct denaturation (304) and the regulatory gene *hrcA* (305). Microbes handle cold shock by increasing the ratio of unsaturated to saturated fatty acids in membrane lipids. This is accomplished via the desaturase gene, *des*; the expression of which is controlled by the two-component system genes, *desK-desR* (306). In addition, there are also cold shock induced chaperon proteins, *cspA* and cspB (307).

Osmotic shock occurs when the cell encounters a sudden change in solute concentration in its surrounding environment, which can lead to a rapid increase or decrease of water in the cell. To protect themselves, microbial cells can modify the concentration of osmoprotectants within the cell using transport systems such as *opuE*, a sodium/proline symporter or the ProU transport system comprised of *proV, proW,* and *proX* (308). The ProU system has a broad substrate specificity, but preferentially transports glycine betaine and proline betaine (309, 310).

An increase in reactive oxygen species can trigger oxidative stress. This stress response is regulated by *perR* and *oxyR* and includes induction of *ahpCF*, an alkyl hydroperoxide reductase, and kata, a catalase, to detoxify reactive oxygen species (311-313).

In environments where there is insufficient oxygen, cytochrome genes (*cydA* and *cydB*) are activated via regulatory genes such as *fnr* and *arcA* and *arcB*, a two-component system (314, 315). In addition, some microorganisms contain genes that allow them to use other electron acceptors, such as nitrate nitrate reductase genes (*narG, narH, narJ*, and *narI*) (316).

Another stressor commonly encountered by microorganisms is nutrient limitation. Two common nutrients that are often limiting are carbon, phosphate and nitrogen. The genes *bglP* (aryl-beta-glucosidespecific enzyme II) and *bglH* (phospho-beta-glucosidase) allow for the use of aryl-β-glucosides as an alternate carbon source (317).

Under phosphate-limiting conditions, the response regulator *phoB* induces expression of the Pst inorganic phosphate (Pi) uptake system. The subunits *pstS, pstA, pstB,* and *pstC* form the transporter. Alkaline phosphatase (*phoA*) releases Pi (318-320) .

External N limitation is sensed by glutamine in enteric bacteria (321). Genes for glutamine synthase (*glnA*) and the regulatory genes *tnrA* and *glnR* were included (322).

Protein stress is triggered by over production of recombinant proteins in microbial cells (323, 324), which induces the activation of heat shock sigma factor σ32 and σ32-dependent genes (325). We selected *clpC* (ATPase subunit in the Clp machinery) and regulator gene *ctsR* to target protein stress (326, 327).

New genes added include antioxidant enzymes such as catalase, peroxidase, and superoxide dismutase, which protect organisms from abiotic and biotically produced oxygen radicals; envelope stress genes which are involved in modifying and repairing the cellular envelope when under stressful conditions (328), and pH stress response genes.

Plant growth promotion:

Plant-microbe interactions are an important aspect of plant growth and health and bacteria and fungi produce a number of compounds to alter host plant metabolism and growth and increase stress tolerance and resistance to pathogens (329). Genes covered in this category include plant-like hormones (9 genes) such as gibberellin, ethylene, auxin and polyamines (spermidine synthase), which are involved in plant growth (330-332) and trehalose synthase genes, which act as a protecting agent to maintain structural integrity of the cytoplasm under environmental stress, such as drought conditions (333). In addition, genes involved in pathogen suppression were included. Siderophores from these beneficial bacteria compete with pathogens for available iron (329) and references therein), so genes related to siderophore production were included. Antioxidants (superoxide dismutase, peroxidase) scavenge reactive oxygen species generated by plants in response to drought, nutrient and other stresses (334, 335).

Microbial defense*:*

Microbial defense mechanisms can indicate the presence of predators or competing microbes.

Antibiotic resistance. Microorganisms are frequently exposed to antibiotics both from natural sources (e.g., other bacteria in the surrounding environment) or from man-made sources (e.g., wastewater treatment plants). As such, microbes have developed mechanism to prevent damage from the antibiotics. These mechanisms can be intrinsic (functional or structural features that prevent the antibiotic from acting against the cell) or acquired (those resistance mechanisms derived from genetic elements that can be passed to other bacteria) (336). Primary mechanisms of resistance include prevention of entry, efflux, modification/absence of the antibiotic target, or inactivation of the antibiotic itself (336). Intrinsic features such as cell wall structures that minimize antibiotic entry or modified/absent targets generally do not require a specific gene to be present, so are difficult to test for with microarrays. So, most of the antibiotic resistance genes covered on the GeoChip are for efflux transporters (8 genes; e.g., ATP-binding cassette (ABC), multi-antimicrobial extrusion protein (MATE), major facilitator superfamily (MFS), resistance-nodulation-division (RND), small multidrug resistance (SMR) transporters) or enzymes responsible for antibiotic degradation (9 genes). Several genes from previous GeoChip versions

were split into multiple genes due to the number of sequences. These splits were done along phylogenetic lines. Two intrinsic resistance mechanisms were also included: the genes *qnr*, which expresses a protein that binds to and protects DNA gyrase and topoisomerase IV from attack by ciprofloxacin (337) and *vanA*, which codes for a ligase that modifies the peptidoglycan binding site for vancomycin and other similar antibiotics (338).

ABC transporters, MFS transporters, MATE transporters, RND transporters (includes Mex gene from previous GeoChip versions), and SMR transporters are broad classes of transporters that are involved in the transport of compounds across the cell membrane, both import and export, including antibiotics and other toxins (339-341). Because the transporters in each of these families is so similar, multiple genes within each family were combined into a single gene family. The individual genes may be specific to a particular genus (*vcaM* is found in *Virbio* spp.) or transport a specific drug type (*macB* transports macrolides) Genes from within each of these categories is listed in the table below.

**Gene family**	**Genes included**	**References**
ABC transporters	*macB, vcaM, smrA, lmrA, lmrCD, horA, yvcC/bmrA, patA/patB, msrA*	(342-350)
MFS transporters	*mdfA, norA, cmlA, mefA, mefE, pmrA, emrB, flo, emeA, bmr3, blt, qacA, lmrB, emrD, emrd-3, qepA, tetBCD, tetKL*	(351-371)
MATE transporters	*mepA, vcrM, abeM, vcmBDHN, norM, hmrM, pmpM*	(372-378)
RND transporters	*acrAB, adeABC, adeDE, adeXYZ, adeFGH, adeIJK, adeMNO, bseAB, bpeEF, cmeABC, cmeDEF, mexAB, mexCD, mexEF, mexJK, mexXY/amrAB, mexHI, mexVW, mexPQ, mexMN, smeABC, smeDEF, sdeAB, sdeCDE, sdeXY, mtrCDEML, ceoAB, tbtABM, zrpADBC, axyABM, bdeAB, bpeAB, vexAB, vexCD, aheABC, ttgABC, vmeAB*	(379-412)

SMR transporters	*mmr, mvrC, qacE/qacEΔ1/qacE2, qac* general, *qacF, qacG, qacJ, qacC, qacH, qacZ, abeS, ssmE, smr, ebrAB, ydgEF, yvdSR, yvaDE*	(413-429)

Beta-lactamases provide resistance to beta-lactam antibiotics such as penicillins, which are used to treat Gram-positive infections and a limited number of Gram-negative infections. There are several different mechanisms for inactivation and the beta-lactamases are classified by those mechanisms (430). The enzymes are separated into different "classes", including Beta-lactamase Class A (431), Beta-lactamase Class B (432), Beta-lactamase class C, (433), and Beta-lacatamase Class D (434).

Vancomycin is a glycopeptide antibiotic for Gram-positive infections. It was considered the "last resort" antibiotic for organisms resistant to other antibiotics. Resistance became common starting in 1990s. There are several variants, but *vanA*, *vanB*, and *vanC* are the main types found in clinical isolates (435).

Streptogramin type antibiotics are the main choice for vancomycin resistant organisms. Resistance to streptogramin is conferred by Vgb (436).

Fosfomycin is a broad spectrum antibacterial used alone or in conjunction with other antibiotics. *fosA* (437), *fosB* (438), and *fosX* (439) all catalyze the addition of another molecule to the oxirane portion of fosfomycin to cause inactivation.

Quinolones are broad spectrum synthetic antibiotics used for both Gram-positive and -negative infections in human medicine and agriculture. There are several different mechanisms of resistance. Qnr is a plasmid borne mechanism that protects bacterial topoisomerases (440).

Tetracycline-type antibiotics are natural and synthetically created polyketide antibiotics that have a broad spectrum of activity by inhibiting protein synthesis. In addition to the MFS transporters mentioned above, there are also enzymatic mechanisms. *TetX* is a monooxygenase that provides resistance to tetracycline antibiotics including those that have been only recently approved such as Tygacil (441). *TetM* and related genes provide resistance to tetracycline antibiotics through

protection of the ribosome (442). Other genes include *tetO* (443), *tetQ* (444), *tetW* (445), *tetS* (446), and *otrA* (447).

Antimicrobial biosynthesis. Microorganisms produce a number of compounds that inhibit growth or kill other organisms (448). These include “classic” antibiotics such as chloramphenicol (para-aminobenzoate synthase, glutamine amidotransferase, component II) and beta-lactams (isopenicillin N synthase), and well as other compounds like phenazines (*phzB*) (449) and pyrrolnitrin (*prnD*) (450), as well as vanadium haloperoxidase, which is involved in the production of various halogenated compounds in algae (451), and hydrogen cyanide synthase, which is involved in the production of the antimicrobial hydrogen cyanide (452).

CRISPR. Bacteria and archaea are under constant pressure from viruses and other mobile parasitic genetic elements. CRISPR-Cas systems are adaptive immune systems used to defend against these elements through a multistep process during which the invader is recognized, short pieces are incorporated between short DNA repeats and used to recognize subsequent infections (453). This “immune system” most likely also plays an important role in the environment in relation to predation by viruses and incorporation of exogenous DNA. The CRISPR locus itself is made up of viral or plasmid sequence genome snippets separated by short repeat sequences. It is not a “functional gene” in and of itself as these repeat sequences are too short to use for our current probe design pipeline and the interspersed viral/plasmid sequences are constantly changing as the organisms are exposed to new sequences. So, CRISPR associated (Cas) genes were chosen for this section. The Cas proteins are suitable for probe design in our pipeline. Previous research has also shown that by knowing which of the Cas proteins are present it is possible to define the type, and even subtype, of the CRISPR system(s) present in an organism. Cas proteins selected (49 genes) covered various types and subtypes of CRISPR-Cas systems were selected, such as *cas* and *cmr* (454, 455).

Environmental toxins. A small portion of marine algae produce toxins that can negatively impact humans and animals (456). Under favorable conditions, harmful algal blooms (HAB) can occur resulting in poisoning through ingestion of contaminated food or water, skin contact, or by inhalation of the toxins. The number of HABs occurring annually has been increasing over the past few decades and the number of areas affected by the blooms have increased likely due to

anthropogenic activities such as eutrophication, transport of harmful species via ballast water, warming-related weather events, and the increasing temperature and CO_2 associated with global climate change (456).

Saxitoxin is a neurotoxin produced by dinaflagellates (marine waters) and cyanobacteria (freshwater) and is the etiological agent of paralytic shellfish poisoning resulting from consuming contaminated shellfish (457). Microcystins are hepatoxins produced by cyanobacteria (458).

Virulence:

Pathogens possess a number of virulence factors that directly or indirectly assist them in infecting and surviving within its host. Genes within this category include those for surface attachment, that aid in the avoidance of the host's immune response, such as capsules (459), type III secretion systems that are involved in the transfer of toxins or effectors into hosts cells (460), invasins, which break down host defenses, siderophores for scavenging for Fe from within the host, and toxins. Because most virulence factors can have other functions other that aiding pathogenicity, all selected sequences were confirmed to have come from microorganisms that are known to be pathogens or opportunistic pathogens. Toxins and hemolysins are always considered to be virulence factors, so all of these selected sequences were included regardless of origin. Many of the genes included in this category were selected from the virulence factor database (Virulence Factors of Pathogenic Bacteria; http://www.mgc.ac.cn/VFs/main.htm).

Toxins and hemolysins damage host cells. Genes within the toxin subcategory include cytolethal distending toxins A, B and C, toxin B, murine toxin, shiga toxin IA and IIA, exfoliative toxins A, B and D, epsilon toxin, RTX toxin A and diphtheria toxin. Hemolysin included sequences for hemolysin III, thermostable direct hemolysin-related hemolysin, heat-labile hemolysin, thermostable hemolysin, adenylate cyclase hemolysin, TlyC family hemolysin, VHH/TLH hemolysin, hemolysin A and B and hemolysin II.

Adhesins are cellular components that aid in attachment of bacterial cells to the host cell (461). Genes within the adherence subcategory include adhesin A, sialic acid-binding adhesin, adhesin Aha1, Dr adhesin, AFA-III adhesin, P fimbrial adhesin PapG, autotransporter adhesin, adhesin

protein HpaA, adhesin MafA, MafB2, adhesin B, type V secretory pathway adhesin AidA, fimbrial adhesin FimH, pilus adhesin HifE, collagen adhesin Cna, collagen-binding adhesin Cnm, adhesin P1, adhesin Hia, F17 fimbrial adhesin protein and adhesin 20 K.

Two bacterial structures that are also involved in adherence are pili and fimbriae. Pili play roles in mobility, surface attachment, and conjugation in bacteria(462, 463). The major protein subunit of pili, pillin (464), was chosen to represent pili. Sequences within this gene family include type IV pilin PilA, type IV pili biogenesis protein PilE, MSHA pilin protein MshABCD, V10 pilin, fimbrial protein EcpC, fimbrial protein pilin FimTU and toxin-coregulated pilin subunit precursor TcpA. Another protein involved in attachment are fimbriae, short proteinaceous appendages present in many Gram-negative and some Gram-positive bacteria (465). Sequences within this gene family included adhesin F41, F18 fimbrial adhesin FedEF, K88 fimbrial protein FaeG, fimbrial subunit F17A, S fimbrial adhesin major subunit SfaA and AC/I pili.

Among the virulence factors that protect bacterial cells from the host immune response are those that protect against phagocytosis (antiphagocytosis subcategory). Bacterial capsules reduce the host immune responses (459) and disguise the bacterial cell. Sequences within this group include CapABCD (capsule biosynthesis), KpsF (polysialic acid capsule expression), KpsC (capsule polysaccharide export), KpsS (capsule polysaccharide export), LipAB (capsule polysaccharide modification), YwsC (capsule biosynthesis), HcsB (capsule polysaccharide modification), GfcE (capsule polysaccharide biosynthesis/export), SiaB (capsule biosynthesis) and PhyAB (capsule polysaccharide export).

Colonization factors allow bacteria to bind and colonize on host cells (466). This subcategory includes colonization factors, AcfA (accessory colonization factor), TcfA (tracheal colonization factor), antigen 1 and antigen b (colonization factors), CsfA and CsaB (major fimbriae subunits), CsuA1 and CsuA2 (CS14 major fimbrial subunits) and CsbA (CS17 fimbriae major subunit). Sortases are a family of proteases and transpeptidases found in Gram-positive bacteria. They are needed for anchoring of surface proteins to the cell wall and adhesion to and colonization of host cells and tissues (467, 468). Sortase sequences are for *srtABCDF*.

The type III secretion system is widespread among Gram-negative pathogens. This secretion system transports effector molecules into host cells (469). Sequences in this subcategory include

the translocation protein PscU, type III secretion proteins HrcCJQRTUV, EscRV, RhcV, RscU, SsaR, BasJ, BsaQ, FlhA and HrpEJQ, type III secretion component proteins HrpE, PsaN, SctCRU and SpaPS, type III secretion invasion protein InvA, type III secretion system ATP synthases FliI/YscN, HrcN, SsaN and SctN, type III secretion inner membrane protein YscR, type III secretion inner membrane channel proteins YscV and BcrD, type III secretion pathway protein LcrD/SctV, type III secretion system apparatus proteins VcrD2, EpaP and SsaV, type III secretion FHIPEP protein, type III secretion outer membrane protein and type III secretion effector delivery regulator.

Invasins are proteins that damage host cell membranes and allow pathogens to invade the cell (470). Invasin genes included in the invasion subcategory include Inv1 and 2, InvAE, IbeA, IagB, CipA, IpaB, HilA, SipAB, YopH, OrgA (oxygen-regulated), IipB (invasion and intracellular persistence protein) and p60 (invasion-associated protein).

Other virulence proteins included sequences from CrfA, SrfB, EsaA, EssB, pGP2-D and IpgD, surface-exposed virulence protein BigA, iron-regulated outer membrane virulence protein IrgA, adherence and virulence protein A and virulence proteins S and Q.

Organism-specific categories

Virus

Bacteriophages are an important part of the microbial community yet how this community changes in relation to environmental factors has not been studied in-depth. In the environment, these viruses are important to the turnover of nutrients by lysis of their hosts, the exchange of genetic information between hosts and to genetic drift by severely depleting or killing off particular strains of a host organism within a local area. Viruses of photosynthetic eukaryotic microorganisms can be important in both environmental and industrial settings. In the environment, they are involved in the turnover of nutrients and population control, especially in bloom situations of toxin producing organisms.

Gene selection for this group included identifying genes necessary for different points in the bacteriophage “life cycle”: replication, infection (host identification, genome injection) structural

components, and lysis of host organism as well as those that identify specific viral groups (genus or family). Proteins related to viral infection (tail fibers), replication (polymerases), and escape/lysis of the host cell (holins) as well as virion structural components (capsid/coat proteins) were selected to cover bacteriophages (prokaryotic hosts) and viral genera or families that infect fungi (mycoviruses) and other protists or who contain members that are known to be soil (e.g. Tobravirus) or water transmitted (e.g. Adenoviridae).

Genes covered in this section include transmission proteins are those that aid in the dispersal of the virus by its vector. These would include Tobravirus transmission protein (471) and Benyvirus p31 transmission protein (472). Movement proteins are involved in cell-to-cell movement of the virus (473). Silencer proteins are those that help to quell the host's response to infection allowing the virus to replicate (474). Killer toxins are found in some mycoviruses. This toxin gives the virus's host an ecological advantage by killing off competing sister strains that do not harbor the virus and the associated toxin and resistance genes. Collagen fibers form an important part of the viral capsid for the Mimiviradae and the virophage. Mimicyp and viral p450 represent genes that researchers believe are of interest and were previously not known to exist in viral genomes. Bacillariodnavirus is a relatively recent addition to protist viruses.

Other functional genes were submitted for specific virus groups (genera or species) rather than solely by function. A description of these follows.

Adenovirus (Adenoviridae) (475) genes include Adenoviridae_fiber for capsid fibers, which play an important role in the recognition and binding of the target receptor on the host cell (476). Adenoviridae_hexon is the major capsid protein of the virus coat and a regular target used in PCR detection of this virus type (477-482). Adenoviridae_protease is used for poly protein processing and is another common PCR target for this virus family (478).

Astroviridae (483) are covered by Astroviridae_capsid, the major capsid protein that is one of the two main targets used for virus detection (484) and Astroviridae_RdRp, the RNA dependent RNA polymerase, the main target for detection of Astroviruses (485).

Hepeviridae (486) are covered by the Hepeviridae_capsid, the major capsid protein is a regular target for pcr detection of this viral group (487, 488) and Hepeviridae_pORF1, which contains

several nonstructural proteins including RdRp and part of this orf has been used as a marker for viral detection(489).

Caliciviridae (490) are covered by RdRp_Caliciviridae and VP1capsid_Caliciviridae (491), both of which have been used for viral detection of this group.

Polyomaviridae (492) are covered by Polyomaviridae_capsid, a common target for this viral group (493, 494) and Polyomaviridae_LT_ag, a large T-antigen that serves multiple purposes in the polyomavirus life cycle including regulation of gene expression, DNA replication and maintenance of the infection (495).

Reoviridae are covered by VP7_Gserotype_Rotavirus, an outer capsid antigens that is commonly used for serotyping (496, 497), VP6_Rotavirus, which encodes for a protein used to define subtypes within the VP7-VP4 types (498), VP4_Pserotype_Rotavirus, an outer capsid antigens that is commonly used for serotyping. and Enterotoxin_Rotavirus, an enterotoxin linked to the cellular cascade that triggers diarrhea (499).

Picornavirales and Picobirnaviridae (500-502) are both covered by Picobirnaviridae_RdRp (503, 504).

Coronaviridae (505) is covered by Coronaviridae_M_protein, which plays an important role on virus assembly (506) and Coronaviridae_spike, a glycoprotein that helps determine host specificity and aids in entry into the host cell (507).

Protozoan:

Protists are key members of environmental food webs by linking different trophic levels together through detritivory and predation of lower levels and serving as food sources for higher levels. They also make significant contributions to primary production. Photosynthetic protists are among the primary aquatic species responsible for primary production and play important roles in the biogeochemical cycling of carbon (C), nitrogen (N) and phosphorus (P) (508, 509). Several genes were selected as phylogenetic markers for various non-fungal protozoan groups. These included actin, cytochrome oxidase subunit 1, glyceraldehyde 3-phosphate dehydrogenase, heat shock

protein 70, heat shock protein 90, elongation factor 1 alpha, polyubiquitin, and tubulin, based on a review of literature (510). Other genes such as trichocyst matrix protein were selected since some protists possess exocytotic organelles that are believed to perform defensive functions (511-513). Movement proteins such as the paraflagellar rod, a feature of kinetoplastid protozoa and necessary for their movement, were also included. These proteins may possibly also play other roles in pathogenesis (514). Attempts were made to cover as many members of the non-fungal protists as possible with genes that have previously been used for phylogenetic purposes by other researchers. Multiple genes were submitted when possible to ensure optimal coverage of the protistal groups.

Oomycetes are plant pathogens that produce a wide variety of avirulence and effector proteins that aid in pathogenesis: (515-518) Protease and glucanase inhibitors and are also believed to aid in maintaining infection (519, 520). The necrosis-inducing protein, involved in host cell death, is also important in the infection process (521). Oomycetes also produce a number of enzymes to help break down host cellular components including pectinases, cutinases and amylases (522, 523). Cercozoa (524) are known to express trehalose synthase in infected plant tissues (525).

Functional genes covered for protists are listed below.

Heterotrophic protists need a variety of carbon degradation enzymes for the breakdown of macromolecules. However, little work on this area been done in relation to most protistal groups, exceptions being the gut symbionts of termites and some ciliates. Covered genes include cellulases (526) and xylanase (527).

Carbon fixation is represented by Rubisco, the most important enzyme for carbon fixation in photosynthetic eukaryotes (528-530).

Nitrogen cycling included ammonium transporter, glutamate dehydrogenase, glutamate synthase, glutamine synthetase, nitrate reductase, nitrate transporter, nitrite reductase, and nitrite transporter (531-533).

Sulfur cycling genes included APS kinase, APS reductase, ATP sulfurylase, cysteine synthase, serine acetyltransferase, sulfate transporter, sulfite reductase (534, 535). Several genes related to

protistan sulfur assimilation, such as sulfate transporters and cysteine synthase (535, 536), were also selected for addition.

Silicon is an important element to a number of protists, including amoebas and diatoms and is used as the base element for the formation of protective shells or other structures (537-540). Silaffins are one of the important organic molecules associated with biosilica formation in diatoms, and the only one for which reliable sequences are known. It has been speculated that silaffins and the other biomolecules are involved in the deposition and patterning of the silica (541). Silicon transporters are needed for uptake of dissolved silicon (542). Genes for silicon biosynthesis (1 gene) used in the production of internal and external skeletons and a silicic acid transporter (1 gene) for internal enrichment of silicon(543) were included.

Photosynthesis is covered by chlorophyll, the major pigment involved in eukaryotic photosynthesis (544) and carotenoids, which act as accessory pigments in photosynthesis and as photoprotectants (545).

Energy processes are represented by carbamate kinase, which is involved in the energy metabolism for a few pathogenic protists, such as Giardia (546, 547).

What little is known about metal cycling in protists has mostly been aimed at metal resistance in relation to contamination though industrial activities (548, 549) and includes cadmium (550) and copper metallothionein (549). Other genes covered include the paraflagellar rod (551), which plays an important part in motilitiy in certain protitst including some important pathogens (514). Trichocysts are believed to be an important part of the defense mechanism for some protest groups such as Paramecium to avoid predation (511). Vanadium bromoperoxidase is an essential enzyme for the production of halogenated metabolites. These metabolites can include antibiotics and other bioactive compounds (552).

Fungi

Fungi are important to the environment and to numerous human activities. In the environment, they help in the turnover of nutrients by degrading a number of large organic molecules, transporters of inorganic nutrients as mycorrhizal symbionts to most land plants, and as

pathogens. To humans, fungi are an important source of food and many other products especially industrially useful enzymes. However, they can also cause a number of economically important diseases that affect humans, livestock, or agriculturally important crop plants.

The genes chosen for inclusion fell into several general categories: Organic remediation, carbon degradation, metal resistance, antifungal resistance, virulence and biogeochemical cycles of iron, sulfur, nitrogen, and phosphorous. The significance of these categories was described above. Specific fungal genes include cyanide dehydratase (553), needed to detoxify cyanide produced by cyanogenic plants during successful infection; enniatin synthase (554, 555), an important virulence gene, scytalone dehydratase (556) is a disease determinant in *Magnaporthe grisea*, an important rice pathogen, and a potassium uptake protein Trk_fungi (557). Chitin is a polysaccharide present in many organisms including fungi. Chitin synthase was added as chitin is an important biomolecule in fungi (558, 559).

Bacterial phylogeny

The phylogenetic marker *gyrB* was included to act as a phylogenetic marker, since it can be used for identification at the species/strain levels (560). The more commonly used 16S rRNA gene has a slower evolution rate, making it difficult to discern closely related strains. This gene was divided into several sets based on phylogenetic groups and included *gyrB*_Arch (archaea), *gyrB*_Actinobacteria, *gyrB*_Firmicutes, *gyrB*_G_proteobacteria, *gyrB*_Proteobacteria, and *gyrB*_Bact_other.

Other Categories

Energy generation

Photosynthetic. Prokaryotes that utilize light either for carbon fixation or other metabolic processes form an important part of the microbial world especially in aquatic environments. Prokaryotic pigments can have a wide range of function including photosynthesis, photoactive protein pumps and pathogenesis (545, 561, 562). Genes for a number of different photoactive systems, with emphasis on photosynthesis, were submitted. These will help our understanding of prokaryotic metabolism in surface environments especially those organisms that fix carbon

dioxide either as their main source of carbon, or as a backup source when fixed organic carbon becomes scarce. In pathogenic organisms these pigments may be involved in virulence mechanisms (562). Genes for the biosynthesis of pigments such as bacteriochlorophyll (16, magnesium protoporphyrin IX methyltransferase), chlorophyll (9, magnesium-protoporphyrin IX chelatase), bilins (4, phycocyanobilin:ferredoxin oxidoreductase), carotenoids (22, lycopene beta cyclase), and rhodopsins (1, bacteriorhodopsin) were selected due to their association with or involvement in photosynthesis and thus impact upon primary production (563-566). Carotenoids are also economically important as antioxidants and have beneficial health effects for humans and other animals.

Bacteriochlorophyll is involved in photosynthesis (567). Carotenoids can be involved in both photosynthesis and as photoprotectants (568). Phycobilins are involved in photosynthesis for a few groups of organisms (565, 569, 570). Proteorhodopsin is a light-driven proton pump and is theorized to have a range of physiological functions (571, 572).

Electron transfer. Microorganisms generate energy by "coupling the flow of electrons in membranes to the creation of an electron motive force" (573). The electrons travel from low to high potential via electron carriers. Prokaryotes use a variety of electron transfer pathways. Genes representing several cytochrome and hydrogenase genes were selected. Cytochromes are heme-containing proteins used to shuttle electrons (574). Hydrogenases catalyze the reversible oxidation of hydrogen, providing reducing ability or acting as an electron sink (575).

Reference

1. He Z, Deng Y, Van Nostrand JD, Tu Q, Xu M, Hemme CL, Li X, Wu L, Gentry TJ, Yin Y, Liebich J, Hazen TC, Zhou J. 2010. GeoChip 3.0 as a high-throughput tool for analyzing microbial community composition, structure and functional activity. ISME J 4:1167-1179.
2. Tu Q, Yu H, He Z, Deng Y, Wu L, Van Nostrand JD, Zhou A, Voordeckers J, Lee Y-J, Qin Y, Hemme CL, Shi Z, Xue K, Yuan T, Wang A, Zhou J. 2014. GeoChip 4: a functional gene-array-based high-throughput environmental technology for microbial community analysis. Molecular Ecology Resources 14:914-928.
3. Li X, He Z, Zhou J. 2005. Selection of optimal oligonucleotide probes for microarrays using multiple criteria, global alignment and parameter estimation. Nucleic acids research 33:6114-6123.
4. Liang Y, He Z, Wu L, Deng Y, Li G, Zhou J. 2010. Development of a common oligonucleotide reference standard for microarray data normalization and comparison across different microbial communities. Applied and environmental microbiology 76:1088-1094.

5. Reich PB, Knops J, Tilman D, Craine J, Ellsworth D, Tjoelker M, Lee T, Wedin D, Naeem S, Bahauddin D, Hendrey G, Jose S, Wrage K, Goth J, Bengston W. 2001. Plant diversity enhances ecosystem responses to elevated CO2 and nitrogen deposition. Nature 410:809-810.
6. Smith MB, Rocha AM, Smillie CS, Olesen SW, Paradis C, Wu L, Campbell JH, Fortney JL, Mehlhorn TL, Lowe KA. 2015. Natural Bacterial Communities Serve as Quantitative Geochemical Biosensors. mBio 6:e00326-15.
7. He Z, Zhang P, Wu L, Rocha AM, Tu Q, Shi Z, Wu B, Qin Y, Wang J, Yan Q. 2018. Microbial Functional Gene Diversity Predicts Groundwater Contamination and Ecosystem Functioning. mBio 9:e02435-17.
8. Zhou J, Bruns MA, Tiedje JM. 1996. DNA recovery from soils of diverse composition. Applied and environmental microbiology 62:316-322.
9. Ahn SJ, Costa J, Emanuel JR. 1996. PicoGreen quantitation of DNA: effective evaluation of samples pre-or psost-PCR. Nucleic acids research 24:2623-2625.
10. Wu L, Liu X, Schadt CW, Zhou J. 2006. Microarray-based analysis of subnanogram quantities of microbial community DNAs by using whole-community genome amplification. Applied and Environmental Microbiology 72:4931-4941.
11. Tu Q, Yu H, He Z, Deng Y, Wu L, Van Nostrand JD, Zhou A, Voordeckers J, Lee YJ, Qin Y. 2014. GeoChip 4: a functional gene‐array‐based high‐throughput environmental technology for microbial community analysis. Molecular ecology resources 14:914-928.
12. Van Nostrand JD, Yin H, Wu L, Yuan T, Zhou J. 2016. Hybridization of environmental microbial community nucleic acids by geochip. Microbial environmental genomics (meg):183-196.
13. Zhou J, Xue K, Xie J, Deng Y, Wu L, Cheng X, Fei S, Deng S, He Z, Van Nostrand JD, Luo Y. 2012. Microbial mediation of carbon-cycle feedbacks to climate warming. Nature Clim Change 2:106-110.
14. Murtagh F, Legendre P. 2014. Ward's hierarchical agglomerative clustering method: which algorithms implement Ward's criterion? Journal of classification 31:274-295.
15. Becker R, Chambers J, Wilks A. 1988. The New S Language: A Programming Environment for Data Analysis and Graphics (Pacific Grove, CA: Wadsworth & Brooks/Cole Advanced Books & Software).
16. Blanchet FG, Legendre P, Borcard D. 2008. Forward selection of explanatory variables. Ecology 89:2623-2632.
17. He Z, Gentry TJ, Schadt CW, Wu L, Liebich J, Chong SC, Huang Z, Wu W, Gu B, Jardine P, Criddle C, Zhou J. 2007. GeoChip: a comprehensive microarray for investigating biogeochemical, ecological and environmental processes. ISME J 1:67-77.
18. Zhou A, He Z, Qin Y, Lu Z, Deng Y, Tu Q, Hemme CL, Van Nostrand JD, Wu L, Hazen TC, Arkin AP, Zhou J. 2013. StressChip as a High-Throughput Tool for Assessing Microbial Community Responses to Environmental Stresses. Environmental Science & Technology 47:9841-9849.
19. Lee Y-J, van Nostrand JD, Tu Q, Lu Z, Cheng L, Yuan T, Deng Y, Carter MQ, He Z, Wu L, Yang F, Xu J, Zhou J. 2013. The PathoChip, a functional gene array for assessing pathogenic properties of diverse microbial communities. ISME J 7:1974-1984.
20. Lee Y-J, van Nostrand JD, Tu Q, Lu Z, Cheng L, Yuan T, Deng Y, Carter MQ, He Z, Wu L. 2013. The PathoChip, a functional gene array for assessing pathogenic properties of diverse microbial communities. The ISME journal 7:1974-1984.
21. Zhou A, He Z, Qin Y, Lu Z, Deng Y, Tu Q, Hemme CL, Van Nostrand JD, Wu L, Hazen TC. 2013. StressChip as a high-throughput tool for assessing microbial community responses to environmental stresses. Environmental science & technology 47:9841-9849.

22. Van Nostrand JD, Zhou A, Zhou J. 2016. StressChip for monitoring microbial stress response in the environment. Stress and Environmental Regulation of Gene Expression and Adaptation in Bacteria, 2 Volume Set.
23. He Z, Gentry TJ, Schadt CW, Wu L, Liebich J, Chong SC, Huang Z, Wu W, Gu B, Jardine P. 2007. GeoChip: a comprehensive microarray for investigating biogeochemical, ecological and environmental processes. The ISME journal 1:67-77.
24. Beguin P. 1990. Molecular Biology of Cellulose Degradation. Annual Review of Microbiology 44:219-248.
25. Ding S-Y, Liu Y-S, Zeng Y, Himmel ME, Baker JO, Bayer EA. 2012. How Does Plant Cell Wall Nanoscale Architecture Correlate with Enzymatic Digestibility? Science 338:1055-1060.
26. Gírio FM, Fonseca C, Carvalheiro F, Duarte LC, Marques S, Bogel-Łukasik R. 2010. Hemicelluloses for fuel ethanol: A review. Bioresource Technology 101:4775-4800.
27. Collins T, Gerday C, Feller G. 2005. Xylanases, xylanase families and extremophilic xylanases. FEMS Microbiol Rev 29:3-23.
28. Shallom D, Shoham Y. 2003. Microbial hemicellulases. Current Opinion in Microbiology 6:219-228.
29. Maris AA, Winkler A, Kuyper M, Laat WAM, Dijken J, Pronk J. 2007. Development of Efficient Xylose Fermentation in Saccharomyces cerevisiae: Xylose Isomerase as a Key Component, p 179-204. *In* Olsson L (ed), Biofuels, vol 108. Springer Berlin Heidelberg.
30. Kersten PJ, Cullen D. 1993. Cloning and characterization of cDNA encoding glyoxal oxidase, a H2O2-producing enzyme from the lignin-degrading basidiomycete Phanerochaete chrysosporium. Proceedings of the National Academy of Sciences 90:7411-7413.
31. Arora DS, Chander M, Gill PK. 2002. Involvement of lignin peroxidase, manganese peroxidase and laccase in degradation and selective ligninolysis of wheat straw. International Biodeterioration & Biodegradation 50:115-120.
32. Santhanam N, Badri DV, Decker SR, Manter DK, Reardon KF, Vivanco JM. 2012. Lignocellulose Decomposition by Microbial Secretions and Exudates in Biological Systems, p 125-153. *In* Vivanco JM, Baluška F (ed), vol 12. Springer Berlin Heidelberg.
33. Chen HP, Chow M, Liu CC, Lau A, Liu J, Eltis LD. 2012. Vanillin catabolism in Rhodococcus jostii RHA1. Appl Environ Microbiol 78:586-8.
34. Chen H-P, Chow M, Liu C-C, Lau A, Liu J, Eltis LD. 2012. Vanillin Catabolism in Rhodococcus jostii RHA1. Applied and Environmental Microbiology 78:586-588.
35. Brunel F, Davison J. 1988. Cloning and sequencing of Pseudomonas genes encoding vanillate demethylase. Journal of Bacteriology 170:4924-4930.
36. Providenti MA, O'Brien JM, Ruff J, Cook AM, Lambert IB. 2006. Metabolism of Isovanillate, Vanillate, and Veratrate by Comamonas testosteroni Strain BR6020. Journal of Bacteriology 188:3862-3869.
37. Overhage J, Priefert H, Rabenhorst J, Steinbüchel A. 1999. Biotransformation of eugenol to vanillin by a mutant of Pseudomonas sp. strain HR199 constructed by disruption of the vanillin dehydrogenase (vdh) gene. Applied Microbiology and Biotechnology 52:820-828.
38. Pedrolli DB, Monteiro AC, Gomes E, Carmona EC. 2009. Pectin and pectinases: production, characterization and industrial application of microbial pectinolytic enzymes. Open Biotechnol J 3:9-18.
39. Marín‐Rodríguez MC, Orchard J, Seymour GB. 2002. Pectate lyases, cell wall degradation and fruit softening. Journal of Experimental Botany 53:2115-2119.
40. Jolie RP, Duvetter T, Van Loey AM, Hendrickx ME. 2010. Pectin methylesterase and its proteinaceous inhibitor: a review. Carbohydr Res 345:2583-95.

41. Kauppinen S, Christgau S, Kofod LV, Halkier T, Dörreich K, Dalbøge H. 1995. Molecular Cloning and Characterization of a Rhamnogalacturonan Acetylesterase from Aspergillus aculeatus: SYNERGISM BETWEEN RHAMNOGALACTURONAN DEGRADING ENZYMES. Journal of Biological Chemistry 270:27172-27178.
42. Itoh T, Ochiai A, Mikami B, Hashimoto W, Murata K. 2006. A Novel Glycoside Hydrolase Family 105: The Structure of Family 105 Unsaturated Rhamnogalacturonyl Hydrolase Complexed with a Disaccharide in Comparison with Family 88 Enzyme Complexed with the Disaccharide. Journal of Molecular Biology 360:573-585.
43. McKie V, Vincken J, Voragen A, Van Den Broek L, Stimson E, Gilbert H. 2001. A new family of rhamnogalacturonan lyases contains an enzyme that binds to cellulose. Biochemical Journal 355:167.
44. Matsuo N, Kaneko S, Kuno A, Kobayashi H, Kusakabe I. 2000. Purification, characterization and gene cloning of two alpha-L-arabinofuranosidases from streptomyces chartreusis GS901. Biochemical Journal 346:9.
45. Numan M, Bhosle N. 2006. α-l-Arabinofuranosidases: the potential applications in biotechnology. Journal of Industrial Microbiology and Biotechnology 33:247-260.
46. Punja ZK, Zhang YY. 1993. Plant chitinases and their roles in resistance to fungal diseases. Journal of nematology 25:526-540.
47. Mathupala SP, Lowe SE, Podkovyrov SM, Zeikus JG. 1993. Sequencing of the amylopullulanase (apu) gene of Thermoanaerobacter ethanolicus 39E, and identification of the active site by site-directed mutagenesis. Journal of Biological Chemistry 268:16332-16344.
48. Coutinho PM, Reilly PJ. 1997. Glucoamylase structural, functional, and evolutionary relationships. Proteins: Structure, Function, and Bioinformatics 29:334-347.
49. Malle D, Itoh T, Hashimoto W, Murata K, Utsumi S, Mikami B. 2006. Overexpression, purification and preliminary X-ray analysis of pullulanase from Bacillus subtilis strain 168. Acta Crystallographica Section F 62:381-384.
50. Souza PMd, Magalhaes PdOe. 2010. Application of microbial a-amylase in industry - A review. Brazilian Journal of Microbiology 41:850-861.
51. Park K-H, Kim T-J, Cheong T-K, Kim J-W, Oh B-H, Svensson B. 2000. Structure, specificity and function of cyclomaltodextrinase, a multispecific enzyme of the α-amylase family. Biochimica et Biophysica Acta (BBA) - Protein Structure and Molecular Enzymology 1478:165-185.
52. Lee HS, Kim MS, Cho HS, Kim JI, Kim TJ, Choi JH, Park C, Lee HS, Oh BH, Park KH. 2002. Cyclomaltodextrinase, neopullulanase, and maltogenic amylase are nearly indistinguishable from each other. J Biol Chem 277:21891-7.
53. Chi Z, Chi Z, Zhang T, Liu G, Yue L. 2009. Inulinase-expressing microorganisms and applications of inulinases. Applied Microbiology and Biotechnology 82:211-220.
54. Chen H-Q, Chen X-M, Li Y, Wang J, Jin Z-Y, Xu X-M, Zhao J-W, Chen T-X, Xie Z-J. 2009. Purification and characterisation of exo- and endo-inulinase from Aspergillus ficuum JNSP5-06. Food Chemistry 115:1206-1212.
55. Liu G-L, Chi Z, Chi Z-M. 2012. Molecular characterization and expression of microbial inulinase genes. Critical Reviews in Microbiology 0:1-14.
56. Edris AE. 2007. Pharmaceutical and therapeutic potentials of essential oils and their individual volatile constituents: a review. Phytother Res 21:308-23.
57. Ambrosio CMS, de Alencar SM, de Sousa RLM, Moreno AM, Da Gloria EM. 2017. Antimicrobial activity of several essential oils on pathogenic and beneficial bacteria. Industrial Crops and Products 97:128-136.

58. Barbirato F, Verdoes JC, de Bont JAM, van der Werf MJ. 1998. The Rhodococcus erythropolis DCL14 limonene-1,2-epoxide hydrolase gene encodes an enzyme belonging to a novel class of epoxide hydrolases. FEBS Letters 438:293-296.
59. van der Werf MJ, van der Ven C, Barbirato F, Eppink MHM, de Bont JAM, van Berkel WJH. 1999. Stereoselective Carveol Dehydrogenase from Rhodococcus erythropolis DCL14: A NOVEL NICOTINOPROTEIN BELONGING TO THE SHORT CHAIN DEHYDROGENASE/REDUCTASE SUPERFAMILY. Journal of Biological Chemistry 274:26296-26304.
60. van der Werf MJ, Boot AM. 2000. Metabolism of carveol and dihydrocarveol in Rhodococcus erythropolis DCL14. Microbiology 146:1129-1141.
61. Arand M, Hallberg BM, Zou J, Bergfors T, Oesch F, van der Werf MJ, de Bont JAM, Jones TA, Mowbray SL. 2003. Structure of Rhodococcus erythropolis limonene-1,2-epoxide hydrolase reveals a novel active site. EMBO J 22:2583-2592.
62. Widersten M, Gurell A, Lindberg D. 2010. Structure–function relationships of epoxide hydrolases and their potential use in biocatalysis. Biochimica et Biophysica Acta (BBA) - General Subjects 1800:316-326.
63. Morisseau C, Hammock BD. 2005. Epoxide hydrolases: mechanisms, inhibitor designs, and biological roles. Annu Rev Pharmacol Toxicol 45:311-333.
64. Unger BP, Gunsalus IC, Sligar SG. 1986. Nucleotide sequence of the Pseudomonas putida cytochrome P-450cam gene and its expression in Escherichia coli. Journal of Biological Chemistry 261:1158-1163.
65. Meilleur F, Dauvergne M-T, Schlichting I, Myles DAA. 2005. Production and X-ray crystallographic analysis of fully deuterated cytochrome P450cam. Acta Crystallographica Section D 61:539-544.
66. Prasad B, Rojubally A, Plettner E. 2011. Identification of Camphor Oxidation and Reduction Products in Pseudomonas putida: New Activity of the Cytochrome P450cam System. Journal of Chemical Ecology 37:657-667.
67. Suenaga H, Goto M, Furukawa K. 2011. Distribution of Camphor Monooxygenase Genes in Soil Bacteria. Indonesian Journal of Biotechnology 10.
68. Kunze M, Pracharoenwattana I, Smith SM, Hartig A. 2006. A central role for the peroxisomal membrane in glyoxylate cycle function. Biochimica et Biophysica Acta (BBA) - Molecular Cell Research 1763:1441-1452.
69. Dunn MF, Ramírez-Trujillo JA, Hernández-Lucas I. 2009. Major roles of isocitrate lyase and malate synthase in bacterial and fungal pathogenesis. Microbiology 155:3166-3175.
70. Berg IA. 2011. Ecological aspects of the distribution of different autotrophic CO2 fixation pathways. Appl Environ Microbiol 77:1925-36.
71. Hügler M, Sievert SM. 2011. Beyond the Calvin Cycle: Autotrophic Carbon Fixation in the Ocean. Annual Review of Marine Science 3:261-289.
72. Berg IA, Kockelkorn D, Buckel W, Fuchs G. 2007. A 3-hydroxypropionate/4-hydroxybutyrate autotrophic carbon dioxide assimilation pathway in Archaea. Science 318:1782-6.
73. Calvin M. 1961. Quantum conversion in photosynthesis. J Theor Biol 1:258-287.
74. Raines C. 2003. The Calvin cycle revisited. Photosynthesis Research 75:1-10.
75. Zarzycki J, Brecht V, Müller M, Fuchs G. 2009. Identifying the missing steps of the autotrophic 3-hydroxypropionate CO2 fixation cycle in Chloroflexus aurantiacus. Proceedings of the National Academy of Sciences 106:21317-21322.
76. Evans MC, Buchanan BB, Arnon DI. 1966. New cyclic process for carbon assimilation by a photosynthetic bacterium. Science 152:673.
77. Ragsdale SW. 1991. Enzymology of the acetyl-CoA pathway of CO2 fixation. Crit Rev Biochem Mol Biol 26:261-300.

78. Huber H, Gallenberger M, Jahn U, Eylert E, Berg IA, Kockelkorn D, Eisenreich W, Fuchs G. 2008. A dicarboxylate/4-hydroxybutyrate autotrophic carbon assimilation cycle in the hyperthermophilic Archaeum Ignicoccus hospitalis. Proceedings of the National Academy of Sciences 105:7851-7856.
79. Berg IA, Kockelkorn D, Buckel W, Fuchs G. 2007. A 3-Hydroxypropionate/4-Hydroxybutyrate Autotrophic Carbon Dioxide Assimilation Pathway in Archaea. Science 318:1782-1786.
80. Espie G, Kimber M. 2011. Carboxysomes: cyanobacterial RubisCO comes in small packages. Photosynthesis Research 109:7-20.
81. Heinhorst S, Cannon GC, Shively JM. 2014. Carboxysomes and Their Structural Organization in Prokaryotes, p 75-101. *In* Barton LL, Bazylinski DA, Xu H (ed), Nanomicrobiology doi:10.1007/978-1-4939-1667-2_4. Springer New York.
82. Kimber M. 2014. Carboxysomes – Sequestering RubisCO for Efficient Carbon Fixation, p 133-148. *In* Hohmann-Marriott MF (ed), The Structural Basis of Biological Energy Generation, vol 39. Springer Netherlands.
83. Dou Z, Heinhorst S, Williams EB, Murin CD, Shively JM, Cannon GC. 2008. CO2 Fixation Kinetics of Halothiobacillus neapolitanus Mutant Carboxysomes Lacking Carbonic Anhydrase Suggest the Shell Acts as a Diffusional Barrier for CO2. Journal of Biological Chemistry 283:10377-10384.
84. EPA. 2017. Inventory of U.S. Greenhouse Gas Emissions and Sinks: 1990-2015.
85. Ferry JG. 2010. How to Make a Living by Exhaling Methane, vol 64. Annual Reviews, Palo Alto, CA, ETATS-UNIS.
86. He Z, Deng Y, Van Nostrand JD, Tu Q, Xu M, Hemme CL, Li X, Wu L, Gentry TJ, Yin Y. 2010. GeoChip 3.0 as a high-throughput tool for analyzing microbial community composition, structure and functional activity. The ISME journal 4:1167-1179.
87. Poly F, Wertz S, Brothier E, Degrange V. 2008. First exploration of Nitrobacter diversity in soils by a PCR cloning-sequencing approach targeting functional gene nxrA. FEMS Microbiology Ecology 63:132-140.
88. Jetten MSM. 2008. The microbial nitrogen cycle. Environmental Microbiology 10:2903-2909.
89. Braker G, Tiedje JM. 2003. Nitric Oxide Reductase (norB) Genes from Pure Cultures and Environmental Samples. Applied and Environmental Microbiology 69:3476-3483.
90. SIAS SR, STOUTHAMER AH, INGRAHAM JL. 1980. The Assimilatory and Dissimilatory Nitrate Reductases of Pseudomonas aeruginosa are Encoded by Different Genes. Journal of General Microbiology 118:229-234.
91. Unthan M, Klipp W, Schmid GH. 1996. Nucleotide sequence of the narB gene encoding assimilatory nitrate reductase from the cyanobacterium Oscillatoria chalybea. Biochimica et Biophysica Acta (BBA) - Gene Structure and Expression 1305:19-24.
92. Flores E, Frías J, Rubio L, Herrero A. 2005. Photosynthetic nitrate assimilation in cyanobacteria. Photosynthesis Research 83:117-133.
93. Paerl RW, Foster RA, Jenkins BD, Montoya JP, Zehr JP. 2008. Phylogenetic diversity of cyanobacterial narB genes from various marine habitats. Environmental Microbiology 10:3377-3387.
94. Martiny AC, Kathuria S, Berube PM. 2009. Widespread metabolic potential for nitrite and nitrate assimilation among Prochlorococcus ecotypes. Proceedings of the National Academy of Sciences 106:10787-10792.
95. Paerl RW, Tozzi S, Kolber ZS, Zehr JP. 2012. VARIATION IN THE ABUNDANCE OF SYNECHOCOCCUS SP. CC9311 NARB MRNA RELATIVE TO CHANGES IN LIGHT, NITROGEN GROWTH CONDITIONS AND NITRATE ASSIMILATION1. Journal of Phycology 48:1028-1039.
96. Kornberg A, Rao NN, Ault-Riche D. 1999. Inorganic polyphosphate: a molecule of many functions. Annu Rev Biochem 68:89-125.

97. Metcalf WW, Wolfe RS. 1998. Molecular Genetic Analysis of Phosphite and Hypophosphite Oxidation by Pseudomonas stutzeriWM88. Journal of Bacteriology 180:5547-5558.
98. White AK, Metcalf WW. 2004. Two C—P Lyase Operons in Pseudomonas stutzeri and Their Roles in the Oxidation of Phosphonates, Phosphite, and Hypophosphite. Journal of Bacteriology 186:4730-4739.
99. White AK, Metcalf WW. 2007. Microbial metabolism of reduced phosphorus compounds. Annual Review of Microbiology 61:379-400.
100. Ishige K, Zhang H, Kornberg A. 2002. Polyphosphate kinase (PPK2), a potent, polyphosphate-driven generator of GTP. Proc Natl Acad Sci U S A 99:16684-8.
101. Rao NN, Gómez-García MR, Kornberg A. 2009. Inorganic Polyphosphate: Essential for Growth and Survival. Annual review of biochemistry 78:605-647.
102. Lens PN, Kuenen JG. 2001. The biological sulfur cycle: novel opportunities for environmental biotechnology. Water Sci Technol 44:57-66.
103. van den Ende FP, Meier J, van Gemerden H. 1997. Syntrophic growth of sulfate-reducing bacteria and colorless sulfur bacteria during oxygen limitation1Dedicated to the memory of Prof. Dr. R.A. Prins.1. FEMS Microbiology Ecology 23:65-80.
104. Curson ARJ, Todd JD, Sullivan MJ, Johnston AWB. 2011. Catabolism of dimethylsulphoniopropionate: microorganisms, enzymes and genes. Nat Rev Micro 9:849-859.
105. Moran MA, Reisch CR, Kiene RP, Whitman WB. 2012. Genomic Insights into Bacterial DMSP Transformations. Annual Review of Marine Science 4:523-542.
106. Reisch CR, Moran MA, Whitman WB. 2011. Bacterial Catabolism of Dimethylsulfoniopropionate (DMSP). Frontiers in microbiology 2:172.
107. Bamford VA, Bruno S, Rasmussen T, Appia-Ayme C, Cheesman MR, Berks BC, Hemmings AM. 2002. Structural basis for the oxidation of thiosulfate by a sulfur cycle enzyme. EMBO J 21:5599-5610.
108. van der Ploeg JR, Barone M, Leisinger T. 2001. Functional analysis of the Bacillus subtilis cysK and cysJI genes. FEMS Microbiol Lett 201:29-35.
109. Adriano DC. 2001. Trace Elements in Terrestrial Environments: Biogeochemistry, Bioavailability, and Risks of Metals, 2nd ed. Springer-Verlag, New York.
110. Silver S, Phung le T. 2005. A bacterial view of the periodic table: genes and proteins for toxic inorganic ions. J Ind Microbiol Biotechnol 32:587-605.
111. Silver S. 1996. Bacterial resistances to toxic metal ions - a review. Gene 179:9-19.
112. Nies DH. 2003. Efflux-mediated heavy metal resistance in prokaryotes. FEMS Microbiology Reviews 27:313-339.
113. Afkar E, Lisak J, Saltikov C, Basu P, Oremland RS, Stolz JF. 2003. The respiratory arsenate reductase from Bacillus selenitireducens strain MLS10. FEMS Microbiology Letters 226:107-112.
114. Zargar K, Hoeft S, Oremland R, Saltikov CW. 2010. Identification of a Novel Arsenite Oxidase Gene, arxA, in the Haloalkaliphilic, Arsenite-Oxidizing Bacterium Alkalilimnicola ehrlichii Strain MLHE-1. Journal of Bacteriology 192:3755-3762.
115. Nozawa A, Takano J, Kobayashi M, Von Wirén N, Fujiwara T. 2006. Roles of BOR1, DUR3, and FPS1 in boron transport and tolerance in Saccharomyces cerevisiae. FEMS Microbiology Letters 262:216-222.
116. Kaya A, Karakaya HC, Fomenko DE, Gladyshev VN, Koc A. 2009. Identification of a novel system for boron transport: Atr1 is a main boron exporter in yeast. Mol Cell Biol 29:3665-74.
117. Ohyama T, Igarashi K, Kobayashi H. 1994. Physiological role of the chaA gene in sodium and calcium circulations at a high pH in Escherichia coli. Journal of Bacteriology 176:4311-4315.

118. Snavely MD, Florer JB, Miller CG, Maguire ME. 1989. Magnesium transport in Salmonella typhimurium: 28Mg2+ transport by the CorA, MgtA, and MgtB systems. Journal of Bacteriology 171:4761-4766.
119. Grass G, Fan B, Rosen BP, Franke S, Nies DH, Rensing C. 2001. ZitB (YbgR), a member of the cation diffusion facilitator family, is an additional zinc transporter in Escherichia coli. J Bacteriol 183:4664-7.
120. Staudenmaier H, Van Hove B, Yaraghi Z, Braun V. 1989. Nucleotide sequences of the fecBCDE genes and locations of the proteins suggest a periplasmic-binding-protein-dependent transport mechanism for iron(III) dicitrate in Escherichia coli. Journal of Bacteriology 171:2626-2633.
121. Velayudhan J, Hughes NJ, McColm AA, Bagshaw J, Clayton CL, Andrews SC, Kelly DJ. 2000. Iron acquisition and virulence in Helicobacter pylori: a major role for FeoB, a high-affinity ferrous iron transporter. Molecular Microbiology 37:274-286.
122. Lundrigan MD, Kadner RJ. 1986. Nucleotide sequence of the gene for the ferrienterochelin receptor FepA in Escherichia coli. Homology among outer membrane receptors that interact with TonB. Journal of Biological Chemistry 261:10797-801.
123. Fecker L, Braun V. 1983. Cloning and expression of the fhu genes involved in iron(III)-hydroxamate uptake by Escherichia coli. Journal of Bacteriology 156:1301-1314.
124. Sauer M, Hantke K, Braun V. 1987. Ferric-coprogen receptor FhuE of Escherichia coli: processing and sequence common to all TonB-dependent outer membrane receptor proteins. Journal of Bacteriology 169:2044-2049.
125. Nikaido H, Rosenberg EY. 1990. Cir and Fiu proteins in the outer membrane of Escherichia coli catalyze transport of monomeric catechols: study with beta-lactam antibiotics containing catechol and analogous groups. Journal of Bacteriology 172:1361-1367.
126. Worsham PL, Konisky J. 1985. Locus affecting regulation of the colicin I receptor by iron. Journal of Bacteriology 161:428-431.
127. Nahlik MS, Fleming TP, McIntosh MA. 1987. Cluster of genes controlling synthesis and activation of 2,3-dihydroxybenzoic acid in production of enterobactin in Escherichia coli. Journal of Bacteriology 169:4163-4170.
128. Andrews SC, Harrison PM, Guest JR. 1989. Cloning, sequencing, and mapping of the bacterioferritin gene (bfr) of Escherichia coli K-12. Journal of Bacteriology 171:3940-3947.
129. Grant RA, Filman DJ, Finkel SE, Kolter R, Hogle JM. 1998. The crystal structure of Dps, a ferritin homolog that binds and protects DNA. Nat Struct Biol 5:294-303.
130. Nunzi F, Woudstra M, Campése D, Bonicel J, Morin D, Bruschi M. 1993. Amino-acid sequence of rusticyanin from Thiobacillus ferrooxidans and its comparison with other blue copper proteins. Biochimica et Biophysica Acta (BBA) - Protein Structure and Molecular Enzymology 1162:28-34.
131. Neilands J. 1995. Siderophores: Structure and Function of Microbial Iron Transport Compounds. Journal of Biological Chemistry 270:26723-26726.
132. P. Bossier MH, W. Verstraete. Ecological Significance of Siderophores in Soil, p 385-414. *In* K.C. M (ed), Advances in Microbial Ecology. Springer, Boston, MA.
133. Maguire ME. 1992. MgtA and MgtB: Prokaryotic P-type ATPases that mediate Mg²⁺ influx. Journal of Bioenergetics and Biomembranes 24:319-328.
134. Smith RL, Thompson LJ, Maguire ME. 1995. Cloning and characterization of MgtE, a putative new class of Mg2+ transporter from Bacillus firmus OF4. Journal of Bacteriology 177:1233-8.
135. Bartsevich VV, Pakrasi HB. 1995. Molecular identification of an ABC transporter complex for manganese: analysis of a cyanobacterial mutant strain impaired in the photosynthetic oxygen evolution process. The EMBO journal 14:1845-1853.

136. Makui H, Roig E, Cole ST, Helmann JD, Gros P, Cellier MFM. 2000. Identification of the Escherichia coli K-12 Nramp orthologue (MntH) as a selective divalent metal ion transporter. Molecular Microbiology 35:1065-1078.
137. Navarro C, Wu L-F, Mandrand-Berthelot M-A. 1993. The nik operon of Escherichia coli encodes a periplasmic binding-protein-dependent transport system for nickel. Molecular Microbiology 9:1181-1191.
138. Li Y, Zamble DB. 2009. Nickel Homeostasis and Nickel Regulation: An Overview. Chemical Reviews 109:4617-4643.
139. Wolfram L, Friedrich B, Eitinger T. 1995. The Alcaligenes eutrophus protein HoxN mediates nickel transport in Escherichia coli. Journal of Bacteriology 177:1840-1843.
140. Fulkerson Jr JF, Garner RM, Mobley HLT. 1998. Conserved Residues and Motifs in the NixA Protein ofHelicobacter pylori Are Critical for the High Affinity Transport of Nickel Ions. Journal of Biological Chemistry 273:235-241.
141. Cai X, Lytton J. 2004. The Cation/Ca2+ Exchanger Superfamily: Phylogenetic Analysis and Structural Implications. Molecular Biology and Evolution 21:1692-1703.
142. Bakker EP. 1983. pH-dependent transport of rubidium by the constitutive potassium uptake system TrkA of Escherichia coli K-12. FEMS Microbiology Letters 16:229-233.
143. Nakamura T, Yuda R, Unemoto T, Bakker EP. 1998. KtrAB, a New Type of Bacterial K+-Uptake System from Vibrio alginolyticus. Journal of Bacteriology 180:3491-3494.
144. Bossemeyer D, Schlösser A, Bakker EP. 1989. Specific cesium transport via the Escherichia coli Kup (TrkD) K+ uptake system. Journal of Bacteriology 171:2219-2221.
145. Dosch DC, Helmer GL, Sutton SH, Salvacion FF, Epstein W. 1991. Genetic analysis of potassium transport loci in Escherichia coli: evidence for three constitutive systems mediating uptake potassium. Journal of Bacteriology 173:687-696.
146. Epstein W, Laimins L. 1980. Potassium transport in Escherichia coli: diverse systems with common control by osmotic forces. Trends in Biochemical Sciences 5:21-23.
147. Munro AW, Ritchie GY, Lamb AJ, Douglas RM, Booth IR. 1991. The cloning and DNA sequence of the gene for the glutathione-regulated potassium-efflux system KefC of Escherichia coli. Molecular Microbiology 5:607-616.
148. Verkhovskaya ML, Barquera B, Verkhovsky MI, Wikström M. 1998. The Na+ and K+ transport deficiency of an E. coli mutant lacking the NhaA and NhaB proteins is apparent and caused by impaired osmoregulation. FEBS Letters 439:271-274.
149. Pinner E, Kotler Y, Padan E, Schuldiner S. 1993. Physiological role of nhaB, a specific Na+/H+ antiporter in Escherichia coli. Journal of Biological Chemistry 268:1729-34.
150. Ito M, Guffanti AA, Zemsky J, Ivey DM, Krulwich TA. 1997. Role of the nhaC-encoded Na+/H+ antiporter of alkaliphilic Bacillus firmus OF4. Journal of Bacteriology 179:3851-7.
151. Nozaki K, Kuroda T, Mizushima T, Tsuchiya T. 1998. A new Na+/H+ antiporter, NhaD, of Vibrio parahaemolyticus. Biochimica et Biophysica Acta (BBA) - Biomembranes 1369:213-220.
152. Radchenko MV, Waditee R, Oshimi S, Fukuhara M, Takabe T, Nakamura T. 2006. Cloning, functional expression and primary characterization of Vibrio parahaemolyticus K+/H+ antiporter genes in Escherichia coli. Molecular Microbiology 59:651-663.
153. Putnoky P, Kereszt A, Nakamura T, Endre G, Grosskopf E, Kiss P, Kondorosi Á. 1998. The pha gene cluster of Rhizobium meliloti involved in pH adaptation and symbiosis encodes a novel type of K+ efflux system. Molecular Microbiology 28:1091-1101.
154. Kosono S, Ohashi Y, Kawamura F, Kitada M, Kudo T. 2000. Function of a Principal Na+/H+ Antiporter, ShaA, Is Required for Initiation of Sporulation in Bacillus subtilis. Journal of Bacteriology 182:898-904.

155. Blanco-Rivero A, Leganes F, Fernandez-Valiente E, Calle P, Fernandez-Pinas F. 2005. mrpA, a gene with roles in resistance to Na+ and adaptation to alkaline pH in the cyanobacterium Anabaena sp. PCC7120. Microbiology 151:1671-82.
156. Barquera B, Häse CC, Gennis RB. 2001. Expression and mutagenesis of the NqrC subunit of the NQR respiratory Na+ pump from Vibrio cholerae with covalently attached FMN. FEBS Letters 492:45-49.
157. Cheng J, Guffanti AA, Krulwich TA. 1997. A two-gene ABC-type transport system that extrudes Na+ in Bacillus subtilis is induced by ethanol or protonophore. Molecular Microbiology 23:1107-1120.
158. Ammendola S, Pasquali P, Pistoia C, Petrucci P, Petrarca P, Rotilio G, Battistoni A. 2007. High-affinity Zn2+ uptake system ZnuABC is required for bacterial zinc homeostasis in intracellular environments and contributes to the virulence of Salmonella enterica. Infect Immun 75:5867-76.
159. Lee YH, Deka RK, Norgard MV, Radolf JD, Hasemann CA. 1999. Treponema pallidum TroA is a periplasmic zinc-binding protein with a helical backbone. Nature structural biology 6:628-633.
160. Dintilhac A, Alloing G, Granadel C, Claverys J-P. 1997. Competence and virulence of Streptococcus pneumoniae: Adc and PsaA mutants exhibit a requirement for Zn and Mn resulting from inactivation of putative ABC metal permeases. Molecular Microbiology 25:727-739.
161. Zhao H, Eide D. 1996. The yeast ZRT1 gene encodes the zinc transporter protein of a high-affinity uptake system induced by zinc limitation. Proceedings of the National Academy of Sciences 93:2454-2458.
162. Gaither LA, Eide DJ. 2001. Eukaryotic zinc transporters and their regulation. BioMetals 14:251-270.
163. Grass G, Wong MD, Rosen BP, Smith RL, Rensing C. 2002. ZupT Is a Zn(II) Uptake System in Escherichia coli. Journal of Bacteriology 184:864-866.
164. MacDiarmid CW, Gaither LA, Eide D. 2000. Zinc transporters that regulate vacuolar zinc storage in Saccharomyces cerevisiae. EMBO J 19:2845-2855.
165. Li L, Kaplan J. 2001. The yeast gene MSC2, a member of the cation diffusion facilitator family, affects the cellular distribution of zinc. J Biol Chem 276:5036-43.
166. Wei Y, Fu D. 2005. Selective Metal Binding to a Membrane-embedded Aspartate in the Escherichia coli Metal Transporter YiiP (FieF). Journal of Biological Chemistry 280:33716-33724.
167. Miyabe S, Izawa S, Inoue Y. 2001. The Zrc1 is involved in zinc transport system between vacuole and cytosol in Saccharomyces cerevisiae. Biochem Biophys Res Commun 282:79-83.
168. Shu L, Chiou YM, Orville AM, Miller MA, Lipscomb JD, Que L. 1995. X-ray absorption spectroscopic studies of the Fe(II) active site of catechol 2,3-dioxygenase. Implications for the extradiol cleavage mechanism. Biochemistry 34:6649-6659.
169. Francisco PB, Ogawa N, Suzuki K, Miyashita K. 2001. The chlorobenzoate dioxygenase genes of Burkholderia sp. strain NK8 involved in the catabolism of chlorobenzoates. Microbiology 147:121-133.
170. Suwa Y, Wright AD, Fukimori F, Nummy KA, Hausinger RP, Holben WE, Forney LJ. 1996. Characterization of a chromosomally encoded 2,4-dichlorophenoxyacetic acid/alpha-ketoglutarate dioxygenase from Burkholderia sp. strain RASC. Applied and Environmental Microbiology 62:2464-9.
171. Köiv V, Marits R, Heinaru A. 1996. Sequence analysis of the 2,4-dichlorophenol hydroxylase gene tfdB and 3,5-dichlorocatechol 1,2-dioxygenase gene tfdC of 2,4-dichlorophenoxyacetic acid degrading plasmid pEST4011. Gene 174:293-297.

172. Ryan TP, Bumpus JA. 1989. Biodegradation of 2,4,5-trichlorophenoxyacetic acid in liquid culture and in soil by the white rot fungus <i>Phanerochaete chrysosporium</i>. Applied Microbiology and Biotechnology 31:302-307.
173. Poelarends GJ, Saunier R, Janssen DB. 2001. trans-3-Chloroacrylic Acid Dehalogenase from Pseudomonas pavonaceae 170 Shares Structural and Mechanistic Similarities with 4-Oxalocrotonate Tautomerase. Journal of Bacteriology 183:4269-4277.
174. Hartmans S, Jansen MW, van der Werf MJ, de Bont JAM. 1991. Bacterial metabolism of 3-chloroacrylic acid. Journal of General Microbiology 137:2025-2032.
175. Nakatsu CH, Straus NA, Wyndham RC. 1995. The nucleotide sequence of the Tn5271 3-chlorobenzoate 3,4-dioxygenase genes (cbaAB) unites the class IA oxygenases in a single lineage. Microbiology 141:485-495.
176. Kohlmiller NA, Howard JB. 1979. The primary structure of the alpha subunit of protocatechuate 3,4-dioxygenase. II. Isolation and sequence of overlap peptides and complete sequence. Journal of Biological Chemistry 254:7309-7315.
177. Williams SE, Woolridge EM, Ransom SC, Landro JA, Babbitt PC, Kozarich JW. 1992. 3-Carboxy-cis,cis-muconate lactonizing enzyme from Pseudomonas putida is homologous to the class II fumarase family: a new reaction in the evolution of a mechanistic motif. Biochemistry 31:9768-9776.
178. Schmitz A, Gartemann KH, Fiedler J, Grund E, Eichenlaub R. 1992. Cloning and sequence analysis of genes for dehalogenation of 4-chlorobenzoate from Arthrobacter sp. strain SU. Applied and Environmental Microbiology 58:4068-4071.
179. Small FJ, Ensign SA. 1997. Alkene Monooxygenase from Xanthobacter Strain Py2. Journal of Biological Chemistry 272:24913-24920.
180. Huang W, Jia J, Cummings J, Nelson M, Schneider G, Lindqvist Y. 1997. Crystal structure of nitrile hydratase reveals a novel iron centre in a novel fold. Structure 5:691-699.
181. Kobayashi M, Yanaka N, Nagasawa T, Yamada H. 1990. Purification and characterization of a novel nitrilase of Rhodococcus rhodochrous K22 that acts on aliphatic nitriles. Journal of Bacteriology 172:4807-4815.
182. Callaghan AV, Wawrik B, Ní Chadhain SM, Young LY, Zylstra GJ. 2008. Anaerobic alkane-degrading strain AK-01 contains two alkylsuccinate synthase genes. Biochemical and Biophysical Research Communications 366:142-148.
183. Liu ZLHYZHPZSJ. 2002. Degradation of aniline by newly isolated, extremely aniline-tolerant <E5>Delftia </E5>sp. AN3. Applied Microbiology and Biotechnology 58:679-682.
184. Boronin AM, Tsoĭ TV, Kosheleva IA, Arinbasarov MU, Adanin VM. 1989. [Cloning of Pseudomonas putida genes responsible for the primary stages of oxidation of naphthalene in Escherichia coli cells]. Genetika 25:226-237.
185. de Souza ML, Sadowsky MJ, Wackett LP. 1996. Atrazine chlorohydrolase from Pseudomonas sp. strain ADP: gene sequence, enzyme purification, and protein characterization. Journal of Bacteriology 178:4894-900.
186. de Souza ML, Seffernick J, Martinez B, Sadowsky MJ, Wackett LP. 1998. The Atrazine Catabolism Genes atzABC Are Widespread and Highly Conserved. Journal of Bacteriology 180:1951-1954.
187. Ostrofsky EB, Robinson JB, Traina SJ, Tuovinen OH. 2001. Effect of cyanuric acid amendment on atrazine mineralization in surface soils and detection of the s-triazine ring-cleavage gene trzD. Soil Biology and Biochemistry 33:1539-1545.
188. Seffernick JL, de Souza ML, Sadowsky MJ, Wackett LP. 2001. Melamine Deaminase and Atrazine Chlorohydrolase: 98 Percent Identical but Functionally Different. Journal of Bacteriology 183:2405-2410.

189. Shaw JP, Harayama S. 1990. Purification and characterisation of TOL plasmid-encoded benzyl alcohol dehydrogenase and benzaldehyde dehydrogenase of Pseudomonas putida. European Journal of Biochemistry 191:705-714.
190. Boschloo JG, Paffen A, Koot T, Tweel WJJ, Gorcom RFM, Cordewener JHG, Bos CJ. 1990. Genetic analysis of benzoate metabolism in <i>Aspergillus niger</i>. Applied Microbiology and Biotechnology 34:225-228.
191. Neidle E, Hartnett C, Ornston LN, Bairoch A, Rekik M, Harayama S. 1992. Cis-diol dehydrogenases encoded by the TOL pWW0 plasmid xylL gene and the Acinetobacter calcoaceticus chromosomal benD gene are members of the short-chain alcohol dehydrogenase superfamily. European Journal of Biochemistry 204:113-120.
192. Koch J, Fuchs G. 1992. Enzymatic reduction of benzoyl-CoA to alicyclic compounds, a key reaction in anaerobic aromatic metabolism. European Journal of Biochemistry 205:195-202.
193. Geissler JF, Harwood CS, Gibson J. 1988. Purification and properties of benzoate-coenzyme A ligase, a Rhodopseudomonas palustris enzyme involved in the anaerobic degradation of benzoate. Journal of Bacteriology 170:1709-1714.
194. Egland PG, Pelletier DA, Dispensa M, Gibson J, Harwood CS. 1997. A cluster of bacterial genes for anaerobic benzene ring biodegradation. Proceedings of the National Academy of Sciences 94:6484-6489.
195. Eberhard ED, Gerlt JA. 2004. Evolution of Function in the Crotonase Superfamily: The Stereochemical Course of the Reaction Catalyzed by 2-Ketocyclohexanecarboxyl-CoA Hydrolase. Journal of the American Chemical Society 126:7188-7189.
196. Pace HC, Brenner C. 2001. The nitrilase superfamily: classification, structure and function. BioMed Central Ltd.
197. Thiery A, Maestracci M, Arnaud A, Galzy P, Nicolas M. 1986. Purification and properties of an acylamide amidohydrolase (E. C. 3.5.1.4) with a wide activity spectrum from Brevibacterium sp. R 312. Journal of Basic Microbiology 26:299-311.
198. Furukawa K, Hayase N, Taira K, Tomizuka N. 1989. Molecular relationship of chromosomal genes encoding biphenyl/polychlorinated biphenyl catabolism: some soil bacteria possess a highly conserved bph operon. Journal of Bacteriology 171:5467-5472.
199. Furukawa K, Hirose J, Suyama A, Zaiki T, Hayashida S. 1993. Gene components responsible for discrete substrate specificity in the metabolism of biphenyl (bph operon) and toluene (tod operon). Journal of Bacteriology 175:5224-5232.
200. Senda T, Sugiyama K, Narita H, Yamamoto T, Kimbara K, Fukuda M, Sato M, Yano K, Mitsui Y. 1996. Three-dimensional Structures of Free Form and Two Substrate Complexes of an Extradiol Ring-cleavage Type Dioxygenase, the BphC Enzyme fromPseudomonassp. Strain KKS102. Journal of Molecular Biology 255:735-752.
201. Hülsmeyer M, Hecht H-J, Niefind K, Schomburg D, Hofer B, Timmis KN, Eltis LD. 1998. Crystal structure of cis-biphenyl-2,3-dihydrodiol-2,3-dehydrogenase from a PCB degrader at 2.0 Å resolution. Protein Science 7:1286-1293.
202. Sato SI, Nam JW, Kasuga K, Nojiri H, Yamane H, Omori T. 1997. Identification and characterization of genes encoding carbazole 1,9a-dioxygenase in Pseudomonas sp. strain CA10. Journal of Bacteriology 179:4850-8.
203. Neidle EL, Hartnett C, Bonitz S, Ornston LN. 1988. DNA sequence of the Acinetobacter calcoaceticus catechol 1,2-dioxygenase I structural gene catA: evidence for evolutionary divergence of intradiol dioxygenases by acquisition of DNA sequence repetitions. Journal of Bacteriology 170:4874-4880.
204. DUGGLEBY CJ, WILLIAMS PA. 1986. Purification and Some Properties of the 2-Hydroxy-6-oxohepta-2,4-dienoate Hydrolase (2-Hydroxymuconic Semialdehyde Hydrolase) Encoded by the

TOL Plasmid pWW0 from Pseudomonas putida mt-2. Journal of General Microbiology 132:717-726.
205. Harayama S, Rekik M, Wasserfallen A, Bairoch A. 1987. Evolutionary relationships between catabolic pathways for aromatics: Conservation of gene order and nucleotide sequences of catechol oxidation genes of pWW0 and NAH7 plasmids. Molecular and General Genetics MGG 210:241-247.
206. Kikuchi Y, Yasukochi Y, Nagata Y, Fukuda M, Takagi M. 1994. Nucleotide sequence and functional analysis of the meta-cleavage pathway involved in biphenyl and polychlorinated biphenyl degradation in Pseudomonas sp. strain KKS102. Journal of Bacteriology 176:4269-4276.
207. Díaz E, Timmis KN. 1995. Identification of Functional Residues in a 2-Hydroxymuconic Semialdehyde Hydrolase. Journal of Biological Chemistry 270:6403-6411.
208. Yrjälä K, Paulin L, Romantschuk M. 1997. Novel organization of catechol meta-pathway genes in Sphingomonas sp. HV3 pSKY4 plasmid. FEMS Microbiology Letters 154:403-408.
209. Vannelli T, Messmer M, Studer A, Vuilleumier S, Leisinger T. 1999. A corrinoid-dependent catabolic pathway for growth of a Methylobacterium strain with chloromethane. Proceedings of the National Academy of Sciences 96:4615-4620.
210. Martinez B, Tomkins J, Wackett LP, Wing R, Sadowsky MJ. 2001. Complete nucleotide sequence and organization of the atrazine catabolic plasmid pADP-1 from Pseudomonas sp. strain ADP. J Bacteriol 183:5684-97.
211. Chen YC, Peoples OP, Walsh CT. 1988. Acinetobacter cyclohexanone monooxygenase: gene cloning and sequence determination. Journal of Bacteriology 170:781-789.
212. Brzostowicz PB, Blasko MB, Rouvière PR. 2002. Identification of two gene clusters involved in cyclohexanone oxidation in <SMALL>Brevibacterium</SMALL> <SMALL>epidermidis</SMALL> strain HCU. Applied Microbiology and Biotechnology 58:781-789.
213. Mirza IA, Yachnin BJ, Wang S, Grosse S, Bergeron Hln, Imura A, Iwaki H, Hasegawa Y, Lau PCK, Berghuis AM. 2009. Crystal Structures of Cyclohexanone Monooxygenase Reveal Complex Domain Movements and a Sliding Cofactor. Journal of the American Chemical Society 131:8848-8854.
214. Iwaki H, Hasegawa Y, Teraoka M, Tokuyama T, Bergeron H, Lau PCK. 1999. Identification of a Transcriptional Activator (ChnR) and a 6-Oxohexanoate Dehydrogenase (ChnE) in the Cyclohexanol Catabolic Pathway in Acinetobacter sp. Strain NCIMB 9871 and Localization of the Genes That Encode Them. Applied and Environmental Microbiology 65:5158-5162.
215. Bes MT, Villa R, Roberts SM, Wan PWH, Willetts A. 1996. Oxidative biotransformations by microorganisms: production of chiral synthons by cyclopentanone monooxygenase fromPseudomonas sp. NCIMB 9872. Journal of Molecular Catalysis B: Enzymatic 1:127-134.
216. Clouthier CM, Kayser MM. 2006. Increasing the enantioselectivity of cyclopentanone monooxygenase (CPMO): profile of new CPMO mutants. Tetrahedron: Asymmetry 17:2649-2653.
217. Iwaki H, Hasegawa Y, Wang S, Kayser MM, Lau PCK. 2002. Cloning and Characterization of a Gene Cluster Involved in Cyclopentanol Metabolism in Comamonas sp. Strain NCIMB 9872 and Biotransformations Effected by Escherichia coli-Expressed Cyclopentanone 1,2-Monooxygenase. Applied and Environmental Microbiology 68:5671-5684.
218. Eaton RW. 1997. p-Cymene catabolic pathway in Pseudomonas putida F1: cloning and characterization of DNA encoding conversion of p-cymene to p-cumate. Journal of Bacteriology 179:3171-80.
219. Lee K, Ryu EK, Choi KS, Cho MC, Jeong JJ, Choi EN, Lee SO, Yoon DY, Hwang I, Kim CK. 2006. Identification and expression of the cym, cmt, and tod catabolic genes from Pseudomonas

putida KL47: expression of the regulatory todST genes as a factor for catabolic adaptation. Journal of microbiology (Seoul, Korea) 44:192-199.
220. Dutta TK, Dutta A, Chakraborty J, Sarkar J, Pal Chowdhury P, Gunsalus IC. 2012. Purification and properties of reductase of the three-component p-cymene methyl hydroxylase from Pseudomonas chlororaphis subsp. aureofaciens. Process Biochemistry 47:1263-1267.
221. Di Gregorio S, Zocca C, Sidler S, Toffanin A, Lizzari D, Vallini G. 2004. Identification of Two New Sets of Genes for Dibenzothiophene Transformation in <i>Burkholderia</i> sp. DBT1. Biodegradation 15:111-123.
222. Hirano S, Haruki M, Takano K, Imanaka T, Morikawa M, Kanaya S. 2006. Gene cloning and in vivo characterization of a dibenzothiophene dioxygenase from Xanthobacter polyaromaticivorans. Appl Microbiol Biotechnol 69:672-81.
223. Bünz PV, Cook AM. 1993. Dibenzofuran 4,4a-dioxygenase from Sphingomonas sp. strain RW1: angular dioxygenation by a three-component enzyme system. Journal of Bacteriology 175:6467-6475.
224. Janssen DB, Pries F, van der Ploeg J, Kazemier B, Terpstra P, Witholt B. 1989. Cloning of 1,2-dichloroethane degradation genes of Xanthobacter autotrophicus GJ10 and expression and sequencing of the dhlA gene. Journal of Bacteriology 171:6791-6799.
225. Anderson DJ, Morris CJ, Nunn DN, Anthony C, Lidstrom ME. 1990. Nucleotide sequence of the Methylobacterium extorquens AM1 moxF and moxJ genes involved in methanol oxidation. Gene 90:173-176.
226. KAWASAKI H, TSUDA K, MATSUSHITA I, TONOMURA K. 1992. Lack of homology between two haloacetate dehalogenase genes encoded on a plasmid from Moraxella sp. strain B. Journal of General Microbiology 138:1317-1323.
227. Bader R, Leisinger T. 1994. Isolation and characterization of the Methylophilus sp. strain DM11 gene encoding dichloromethane dehalogenase/glutathione S-transferase. Journal of Bacteriology 176:3466-3473.
228. Weiner JH, MacIsaac DP, Bishop RE, Bilous PT. 1988. Purification and properties of Escherichia coli dimethyl sulfoxide reductase, an iron-sulfur molybdoenzyme with broad substrate specificity. Journal of Bacteriology 170:1505-1510.
229. Kok M, Oldenhuis R, van der Linden MP, Meulenberg CH, Kingma J, Witholt B. 1989. The Pseudomonas oleovorans alkBAC operon encodes two structurally related rubredoxins and an aldehyde dehydrogenase. Journal of Biological Chemistry 264:5442-5451.
230. Kok M, Oldenhuis R, van der Linden MP, Raatjes P, Kingma J, van Lelyveld PH, Witholt B. 1989. The Pseudomonas oleovorans alkane hydroxylase gene. Sequence and expression. Journal of Biological Chemistry 264:5435-5441.
231. Davison J, Brunel F, Phanopoulos A, Prozzi D, Terpstra P. 1992. Cloning and sequencing of Pseudomonas genes determining sodium dodecyl sulfate biodegradation. Gene 114:19-24.
232. Johnson HA, Pelletier DA, Spormann AM. 2001. Isolation and characterization of anaerobic ethylbenzene dehydrogenase, a novel Mo-Fe-S enzyme. J Bacteriol 183:4536-42.
233. Jobst B, Schühle K, Linne U, Heider J. 2010. ATP-Dependent Carboxylation of Acetophenone by a Novel Type of Carboxylase. Journal of Bacteriology 192:1387-1394.
234. Kniemeyer O, Heider J. 2001. (S)-1-Phenylethanol dehydrogenase of Azoarcus sp. strain EbN1, an enzyme of anaerobic ethylbenzene catabolism. Archives of Microbiology 176:129-135.
235. Kim D, Chae J-C, Zylstra GJ, Kim Y-S, Kim S-K, Nam MH, Kim YM, Kim E. 2004. Identification of a Novel Dioxygenase Involved in Metabolism of o-Xylene, Toluene, and Ethylbenzene by Rhodococcus sp. Strain DK17. Applied and Environmental Microbiology 70:7086-7092.

236. Nagata Y, Miyauchi K, Takagi M. 1999. Complete analysis of genes and enzymes for γ-hexachlorocyclohexane degradation in <i>Sphingomonas paucimobilis</i> UT26. Journal of Industrial Microbiology & Biotechnology 23:380-390.
237. Baas D, Rétey J. 1999. Cloning, sequencing and heterologous expression of pyrogallol-phloroglucinol transhydroxylase from Pelobacter acidigallici. European Journal of Biochemistry 265:896-901.
238. Chen CM, Ye QZ, Zhu ZM, Wanner BL, Walsh CT. 1990. Molecular biology of carbon-phosphorus bond cleavage. Cloning and sequencing of the phn (psiD) genes involved in alkylphosphonate uptake and C-P lyase activity in Escherichia coli B. Journal of Biological Chemistry 265:4461-4471.
239. Chistoserdov AY, Boyd J, Mathews FS, Lidstrom ME. 1992. The genetic organization of the mau gene cluster of the facultative autotroph Paracoccus denitrificans. Biochemical and Biophysical Research Communications 184:1181-1189.
240. Habe H, Kasuga K, Nojiri H, Yamane H, Omori T. 1996. Analysis of cumene (isopropylbenzene) degradation genes from Pseudomonas fluorescens IP01. Applied and Environmental Microbiology 62:4471-7.
241. Choi KD, Jeohn GH, Rhee JS, Yoo OJ. 1990. Cloning and Nucleotide Sequence of an Esterase Gene from <i>Pseudomonas fluorescens</i> and Expression of the Gene in <i>Escherichia coli</i>. Agricultural and Biological Chemistry 54:2039-2045.
242. Tsou AY, Ransom SC, Gerlt JA, Buechter DD, Babbitt PC, Kenyon GL. 1990. Mandelate pathway of Pseudomonas putida: sequence relationships involving mandelate racemase, (S)-mandelate dehydrogenase, and benzoylformate decarboxylase and expression of benzoylformate decarboxylase in Escherichia coli. Biochemistry 29:9856-9862.
243. Higgins TP, Davey M, Trickett J, Kelly DP, Murrell JC. 1996. Metabolism of methanesulfonic acid involves a multicomponent monooxygenase enzyme. Microbiology 142:251-260.
244. Aislabie J, Bej AK, Hurst H, Rothenburger S, Atlas RM. 1990. Microbial degradation of quinoline and methylquinolines. Applied and Environmental Microbiology 56:345-351.
245. Staijen IE, Hatzimanikatis V, Witholt B. 1997. The AlkB Monooxygenase of Pseudomonas oleovorans. European Journal of Biochemistry 244:462-470.
246. Yen K-M, Serdar CM, Gunsalus IC. 1988. Genetics of Naphthalene Catabolism in Pseudomonads. Critical Reviews in Microbiology 15:247-268.
247. Brandsch R, Hinkkanen AE, Mauch L, Nagursky H, Decker K. 1987. 6-Hydroxy-D-nicotine oxidase of Arthrobacter oxidans. European Journal of Biochemistry 167:315-320.
248. Ma Y-F, Wu J-F, Wang S-Y, Jiang C-Y, Zhang Y, Qi S-W, Liu L, Zhao G-P, Liu S-J. 2007. Nucleotide Sequence of Plasmid pCNB1 from Comamonas Strain CNB-1 Reveals Novel Genetic Organization and Evolution for 4-Chloronitrobenzene Degradation. Applied and Environmental Microbiology 73:4477-4483.
249. Muraki T, Taki M, Hasegawa Y, Iwaki H, Lau PCK. 2003. Prokaryotic Homologs of the Eukaryotic 3-Hydroxyanthranilate 3,4-Dioxygenase and 2-Amino-3-Carboxymuconate-6-Semialdehyde Decarboxylase in the 2-Nitrobenzoate Degradation Pathway of Pseudomonas fluorescens Strain KU-7. Applied and Environmental Microbiology 69:1564-1572.
250. Blehert DS, Fox BG, Chambliss GH. 1999. Cloning and Sequence Analysis of Two PseudomonasFlavoprotein Xenobiotic Reductases. Journal of Bacteriology 181:6254-6263.
251. Müller D, Schlömann M, Reineke W. 1996. Maleylacetate reductases in chloroaromatic-degrading bacteria using the modified ortho pathway: comparison of catalytic properties. Journal of Bacteriology 178:298-300.

252. Takeo M, Yasukawa T, Abe Y, Niihara S, Maeda Y, Negoro S. 2003. Cloning and characterization of a 4-nitrophenol hydroxylase gene cluster from Rhodococcus sp. PN1. Journal of Bioscience and Bioengineering 95:139-145.
253. Kitagawa W, Kimura N, Kamagata Y. 2004. A novel p-nitrophenol degradation gene cluster from a gram-positive bacterium, Rhodococcus opacus SAO101. J Bacteriol 186:4894-902.
254. Takeo M, Murakami M, Niihara S, Yamamoto K, Nishimura M, Kato D-i, Negoro S. 2008. Mechanism of 4-Nitrophenol Oxidation in Rhodococcus sp. Strain PN1: Characterization of the Two-Component 4-Nitrophenol Hydroxylase and Regulation of Its Expression. Journal of Bacteriology 190:7367-7374.
255. Chakrabarty AM, Chou G, Gunsalus IC. 1973. Genetic Regulation of Octane Dissimilation Plasmid in Pseudomonas. Proceedings of the National Academy of Sciences 70:1137-1140.
256. McDaniel CS, Harper LL, Wild JR. 1988. Cloning and sequencing of a plasmid-borne gene (opd) encoding a phosphotriesterase. Journal of Bacteriology 170:2306-2311.
257. Cai M, Xun L. 2002. Organization and Regulation of Pentachlorophenol-Degrading Genes in Sphingobium chlorophenolicum ATCC 39723. Journal of Bacteriology 184:4672-4680.
258. Louie TM, Webster CM, Xun L. 2002. Genetic and Biochemical Characterization of a 2,4,6-Trichlorophenol Degradation Pathway in Ralstonia eutropha JMP134. Journal of Bacteriology 184:3492-3500.
259. French CE, Rosser SJ, Davies GJ, Nicklin S, Bruce NC. 1999. Biodegradation of explosives by transgenic plants expressing pentaerythritol tetranitrate reductase. Nat Biotech 17:491-494.
260. Kiyohara H, Nagao K. 1978. The Catabolism of Phenanthrene and Naphthalene by Bacteria. Journal of General Microbiology 105:69-75.
261. Entsch B, Nan Y, Weaich K, Scott KF. 1988. Sequence and organization of pobA, the gene coding for p-hydroxybenzoate hydroxylase, an inducible enzyme from Pseudomonas aeruginosa. Gene 71:279-291.
262. Dehmel U, Engesser K-H, Timmis KN, Dwyer DF. 1995. Cloning, nucleotide sequence, and expression of the gene encoding a novel dioxygenase involved in metabolism of carboxydiphenyl ethers in <i>Pseudomonas pseudoalcaligenes</i> POB310. Archives of Microbiology 163:35-41.
263. Kato Y, Nakamura K, Sakiyama H, Mayhew SG, Asano Y. 2000. Novel Heme-Containing Lyase, Phenylacetaldoxime Dehydratase from Bacillus sp. Strain OxB-1: Purification, Characterization, and Molecular Cloning of the Gene†,‡. Biochemistry 39:800-809.
264. Díaz E, Ferrández A, García JL. 1998. Characterization of the hca Cluster Encoding the Dioxygenolytic Pathway for Initial Catabolism of 3-Phenylpropionic Acid in Escherichia coliK-12. Journal of Bacteriology 180:2915-2923.
265. Ferrández A, Garciá JL, Díaz E. 1997. Genetic characterization and expression in heterologous hosts of the 3-(3-hydroxyphenyl)propionate catabolic pathway of Escherichia coli K-12. Journal of Bacteriology 179:2573-81.
266. Nomura Y, Nakagawa M, Ogawa N, Harashima S, Oshima Y. 1992. Genes in PHT plasmid encoding the initial degradation pathway of phthalate in Pseudomonas putida. Journal of Fermentation and Bioengineering 74:333-344.
267. Chang H-K, Zylstra GJ. 1998. Novel Organization of the Genes for Phthalate Degradation from Burkholderia cepacia DBO1. Journal of Bacteriology 180:6529-6537.
268. Cartwright CD, Thompson IP, Burns RG. 2000. Degradation and impact of phthalate plasticizers on soil microbial communities. Environmental Toxicology and Chemistry 19:1253-1261.
269. Hiromoto T, Fujiwara S, Hosokawa K, Yamaguchi H. 2006. Crystal Structure of 3-Hydroxybenzoate Hydroxylase from Comamonas testosteroni Has a Large Tunnel for Substrate and Oxygen Access to the Active Site. Journal of Molecular Biology 364:878-896.

270. Sho M, Hamel C, Greer CW. 2004. Two distinct gene clusters encode pyrene degradation in Mycobacterium sp. strain S65. FEMS Microbiol Ecol 48:209-20.
271. Holliger C, Wohlfarth G, Diekert G. 1998. Reductive dechlorination in the energy metabolism of anaerobic bacteria. FEMS Microbiology Reviews 22:383-398.
272. Futagami T, Morono Y, Terada T, Kaksonen AH, Inagaki F. 2009. Dehalogenation Activities and Distribution of Reductive Dehalogenase Homologous Genes in Marine Subsurface Sediments. Applied and Environmental Microbiology 75:6905-6909.
273. You IS, Ghosal D, Gunsalus IC. 1991. Nucleotide sequence analysis of the Pseudomonas putida PpG7 salicylate hydroxylase gene (nahG) and its 3'-flanking region. Biochemistry 30:1635-1641.
274. Lee J, Oh J, Min KR, Kim Y. 1996. Nucleotide Sequence of Salicylate Hydroxylase Gene and Its 5′-Flanking Region ofPseudomonas putidaKF715. Biochemical and Biophysical Research Communications 218:544-548.
275. Zhou NY, Fuenmayor SL, Williams PA. 2001. nag genes of Ralstonia (formerly Pseudomonas) sp. strain U2 encoding enzymes for gentisate catabolism. J Bacteriol 183:700-8.
276. Thiemer B, Andreesen JR, Schrader T. 2003. Cloning and characterization of a gene cluster involved in tetrahydrofuran degradation in Pseudonocardia sp. strain K1. Arch Microbiol 179:266-77.
277. Katayama Y, Narahara Y, Inoue Y, Amano F, Kanagawa T, Kuraishi H. 1992. A thiocyanate hydrolase of Thiobacillus thioparus. A novel enzyme catalyzing the formation of carbonyl sulfide from thiocyanate. Journal of Biological Chemistry 267:9170-9175.
278. Harayama S, Rekik M, Bairoch A, Neidle EL, Ornston LN. 1991. Potential DNA slippage structures acquired during evolutionary divergence of Acinetobacter calcoaceticus chromosomal benABC and Pseudomonas putida TOL pWW0 plasmid xylXYZ, genes encoding benzoate dioxygenases. Journal of Bacteriology 173:7540-7548.
279. Horn JM, Harayama S, Timmis KN. 1991. DNA sequence determination of the TOL plasmid (pWW0) xylGFJ genes of Pseudomonas putida: implications for the evolution of aromatic catabolism. Molecular Microbiology 5:2459-2474.
280. Suzuki M, Hayakawa T, Shaw JP, Rekik M, Harayama S. 1991. Primary structure of xylene monooxygenase: similarities to and differences from the alkane hydroxylation system. Journal of Bacteriology 173:1690-1695.
281. Yen KM, Karl MR, Blatt LM, Simon MJ, Winter RB, Fausset PR, Lu HS, Harcourt AA, Chen KK. 1991. Cloning and characterization of a Pseudomonas mendocina KR1 gene cluster encoding toluene-4-monooxygenase. Journal of Bacteriology 173:5315-5327.
282. Shields MS, Reagin MJ, Gerger RR, Campbell R, Somerville C. 1995. TOM, a new aromatic degradative plasmid from Burkholderia (Pseudomonas) cepacia G4. Applied and Environmental Microbiology 61:1352-6.
283. Leuthner B, Heider J. 2000. Anaerobic Toluene Catabolism of Thauera aromatica: the bbs Operon Codes for Enzymes of β Oxidation of the Intermediate Benzylsuccinate. Journal of Bacteriology 182:272-277.
284. Greated A, Lambertsen L, Williams PA, Thomas CM. 2002. Complete sequence of the IncP-9 TOL plasmid pWW0 from Pseudomonas putida. Environmental Microbiology 4:856-871.
285. Aldrich TL, Chakrabarty AM. 1988. Transcriptional regulation, nucleotide sequence, and localization of the promoter of the catBC operon in Pseudomonas putida. Journal of Bacteriology 170:1297-1304.
286. Yeates C, Holmes AJ, Gillings MR. 2000. Novel forms of ring-hydroxylating dioxygenases are widespread in pristine and contaminated soils. Environmental Microbiology 2:644-653.
287. Bagneris C, Cammack R, Mason JR. 2005. Subtle difference between benzene and toluene dioxygenases of Pseudomonas putida. Appl Environ Microbiol 71:1570-80.

288. Taylor PM, Janssen PH. 2005. Variations in the abundance and identity of class II aromatic ring-hydroxylating dioxygenase genes in groundwater at an aromatic hydrocarbon-contaminated site. Environmental Microbiology 7:140-146.
289. Erickson BD, Mondello FJ. 1992. Nucleotide sequencing and transcriptional mapping of the genes encoding biphenyl dioxygenase, a multicomponent polychlorinated-biphenyl-degrading enzyme in Pseudomonas strain LB400. Journal of Bacteriology 174:2903-2912.
290. Pflugmacher U, Averhoff B, Gottschalk G. 1996. Cloning, sequencing, and expression of isopropylbenzene degradation genes from Pseudomonas sp. strain JR1: identification of isopropylbenzene dioxygenase that mediates trichloroethene oxidation. Applied and Environmental Microbiology 62:3967-77.
291. Kumar P, Mohammadi M, Viger J-F, Barriault D, Gomez-Gil L, Eltis LD, Bolin JT, Sylvestre M. 2011. Structural Insight into the Expanded PCB-Degrading Abilities of a Biphenyl Dioxygenase Obtained by Directed Evolution. Journal of Molecular Biology 405:531-547.
292. Lu X-Y, Zhang T, Fang H. 2011. Bacteria-mediated PAH degradation in soil and sediment. Applied Microbiology and Biotechnology 89:1357-1371.
293. Martin F, Malagnoux L, Violet F, Jakoncic J, Jouanneau Y. 2012. Diversity and catalytic potential of PAH-specific ring-hydroxylating dioxygenases from a hydrocarbon-contaminated soil. Applied Microbiology and Biotechnology doi:10.1007/s00253-012-4335-2:1-11.
294. Bengtsson O, Hahn-Hagerdal B, Gorwa-Grauslund MF. 2009. Xylose reductase from Pichia stipitis with altered coenzyme preference improves ethanolic xylose fermentation by recombinant Saccharomyces cerevisiae. Biotechnol Biofuels 2:9.
295. Zenno S, Kobori T, Tanokura M, Saigo K. 1998. Conversion of NfsA, the Major Escherichia coliNitroreductase, to a Flavin Reductase with an Activity Similar to That of Frp, a Flavin Reductase in Vibrio harveyi, by a Single Amino Acid Substitution. Journal of Bacteriology 180:422-425.
296. Pérez-Reinado E, Blasco R, Castillo F, Moreno-Vivián C, Roldán MD. 2005. Regulation and Characterization of Two Nitroreductase Genes, nprA and nprB, of Rhodobacter capsulatus. Applied and Environmental Microbiology 71:7643-7649.
297. Hengge-Aronis R. 2002. Signal Transduction and Regulatory Mechanisms Involved in Control of the S (RpoS) Subunit of RNA Polymerase. Microbiology and Molecular Biology Reviews 66:373-395.
298. Engelmann S, Lindner C, Hecker M. 1995. Cloning, nucleotide sequence, and regulation of katE encoding a sigma B-dependent catalase in Bacillus subtilis. J Bacteriol 177:5598-605.
299. Scott JM, Haldenwang WG. 1999. Obg, an essential GTP binding protein of Bacillus subtilis, is necessary for stress activation of transcription factor sigma(B). J Bacteriol 181:4653-60.
300. Raskin DM, Judson N, Mekalanos JJ. 2007. Regulation of the stringent response is the essential function of the conserved bacterial G protein CgtA in Vibrio cholerae. Proc Natl Acad Sci U S A 104:4636-41.
301. Persky NS, Ferullo DJ, Cooper DL, Moore HR, Lovett ST. 2009. The ObgE/CgtA GTPase influences the stringent response to amino acid starvation in Escherichia coli. Mol Microbiol 73:253-66.
302. Wout P, Pu K, Sullivan SM, Reese V, Zhou S, Lin B, Maddock JR. 2004. The Escherichia coli GTPase CgtAE cofractionates with the 50S ribosomal subunit and interacts with SpoT, a ppGpp synthetase/hydrolase. J Bacteriol 186:5249-57.
303. Hauryliuk V, Atkinson GC, Murakami KS, Tenson T, Gerdes K. 2015. Recent functional insights into the role of (p)ppGpp in bacterial physiology. Nat Rev Microbiol 13:298-309.
304. Schumann W. 1996. Regulation of the heat shock response inEscherichia coli andBacillus subtilis. Journal of Biosciences 21:133-148.

305. Schulz A, Schumann W. 1996. hrcA, the first gene of the Bacillus subtilis dnaK operon encodes a negative regulator of class I heat shock genes. J Bacteriol 178:1088-93.
306. Aguilar PS, Hernandez-Arriaga AM, Cybulski LE, Erazo AC, de Mendoza D. 2001. Molecular basis of thermosensing: a two-component signal transduction thermometer in Bacillus subtilis. Embo j 20:1681-91.
307. Phadtare S, Alsina J, Inouye M. 1999. Cold-shock response and cold-shock proteins. Curr Opin Microbiol 2:175-80.
308. Kempf B, Bremer E. 1998. Uptake and synthesis of compatible solutes as microbial stress responses to high-osmolality environments. Arch Microbiol 170:319-30.
309. Gouesbet G, Jebbar M, Talibart R, Bernard T, Blanco C. 1994. Pipecolic acid is an osmoprotectant for Escherichia coli taken up by the general osmoporters ProU and ProP. Microbiology 140 (Pt 9):2415-22.
310. Haardt M, Kempf B, Faatz E, Bremer E. 1995. The osmoprotectant proline betaine is a major substrate for the binding-protein-dependent transport system ProU of Escherichia coli K-12. Mol Gen Genet 246:783-6.
311. Fuangthong M, Herbig AF, Bsat N, Helmann JD. 2002. Regulation of the Bacillus subtilis fur and perR genes by PerR: not all members of the PerR regulon are peroxide inducible. J Bacteriol 184:3276-86.
312. Pomposiello PJ, Demple B. 2001. Redox-operated genetic switches: the SoxR and OxyR transcription factors. Trends Biotechnol 19:109-14.
313. Mostertz J, Scharf C, Hecker M, Homuth G. 2004. Transcriptome and proteome analysis of Bacillus subtilis gene expression in response to superoxide and peroxide stress. Microbiology 150:497-512.
314. Govantes F, Orjalo AV, Gunsalus RP. 2000. Interplay between three global regulatory proteins mediates oxygen regulation of the Escherichia coli cytochrome d oxidase (cydAB) operon. Molecular Microbiology 38:1061-1073.
315. Spiro S, Guest JR. 1991. Adaptive responses to oxygen limitation inEscherichia coli. Trends in biochemical sciences 16:310-314.
316. and MMN, Zuber P. 1998. ANAEROBIC GROWTH OF A “STRICT AEROBE” (BACILLUS SUBTILIS). Annual Review of Microbiology 52:165-190.
317. Krüger S, Hecker M. 1995. Regulation of the putative bglPH operon for aryl-beta-glucoside utilization in Bacillus subtilis. Journal of Bacteriology 177:5590-5597.
318. Fiedler T, Mix M, Meyer U, Mikkat S, Glocker MO, Bahl H, Fischer R-J. 2008. The Two-Component System PhoPR of Clostridium acetobutylicum Is Involved in Phosphate-Dependent Gene Regulation. Journal of Bacteriology 190:6559.
319. Torriani-Gorini A. 1996. History of the Pho System, p 291-295, Regulation of Gene Expression in Escherichia coli doi:10.1007/978-1-4684-8601-8_14. Springer US, Boston, MA.
320. Fischer R-J, Oehmcke S, Meyer U, Mix M, Schwarz K, Fiedler T, Bahl H. 2006. Transcription of the pst Operon of Clostridium acetobutylicum Is Dependent on Phosphate Concentration and pH. Journal of Bacteriology 188:5469.
321. Yan D. 2007. Protection of the glutamate pool concentration in enteric bacteria. Proc Natl Acad Sci U S A 104:9475-80.
322. Wray LV, Jr., Ferson AE, Rohrer K, Fisher SH. 1996. TnrA, a transcription factor required for global nitrogen regulation in Bacillus subtilis. Proc Natl Acad Sci U S A 93:8841-5.
323. Goff SA, Goldberg AL. 1985. Production of abnormal proteins in E. coli stimulates transcription of lon and other heat shock genes. Cell 41:587-95.
324. Dong H, Nilsson L, Kurland CG. 1995. Gratuitous overexpression of genes in Escherichia coli leads to growth inhibition and ribosome destruction. J Bacteriol 177:1497-504.

325. Kosinski MJ, Rinas U, Bailey JE. 1992. Proteolytic response to the expression of an abnormal beta-galactosidase in Escherichia coli. Appl Microbiol Biotechnol 37:335-41.
326. Kruger E, Witt E, Ohlmeier S, Hanschke R, Hecker M. 2000. The clp proteases of Bacillus subtilis are directly involved in degradation of misfolded proteins. J Bacteriol 182:3259-65.
327. Derre I, Rapoport G, Msadek T. 1999. CtsR, a novel regulator of stress and heat shock response, controls clp and molecular chaperone gene expression in gram-positive bacteria. Mol Microbiol 31:117-31.
328. Raivio TL. 2005. MicroReview: Envelope stress responses and Gram‐negative bacterial pathogenesis. Molecular microbiology 56:1119-1128.
329. Davison J. 1988. Plant beneficial bacteria. Nature Biotechnology 6:282-286.
330. Xie S-S, Wu H-J, Zang H-Y, Wu L-M, Zhu Q-Q, Gao X-W. 2014. Plant Growth Promotion by Spermidine-Producing Bacillus subtilis OKB105. Molecular Plant-Microbe Interactions 27:655-663.
331. Glick BR. 2012. Plant Growth-Promoting Bacteria: Mechanisms and Applications. Scientifica 2012:15.
332. Spaepen S, Vanderleyden J. 2011. Auxin and Plant-Microbe Interactions. Cold Spring Harbor Perspectives in Biology 3.
333. Pan YT, Koroth Edavana V, Jourdian WJ, Edmondson R, Carroll JD, Pastuszak I, Elbein AD. 2004. Trehalose synthase of Mycobacterium smegmatis. European Journal of Biochemistry 271:4259-4269.
334. Kakar KU, Ren XL, Nawaz Z, Cui ZQ, Li B, Xie GL, Hassan MA, Ali E, Sun GC. 2016. A consortium of rhizobacterial strains and biochemical growth elicitors improve cold and drought stress tolerance in rice (Oryza sativa L.). Plant Biol (Stuttg) 18:471-83.
335. Radhakrishnan R, Hashem A, Abd Allah EF. 2017. Bacillus: A Biological Tool for Crop Improvement through Bio-Molecular Changes in Adverse Environments. Front Physiol 8:667.
336. Blair JM, Webber MA, Baylay AJ, Ogbolu DO, Piddock LJ. 2015. Molecular mechanisms of antibiotic resistance. Nature Reviews Microbiology 13:42-51.
337. Tran JH, Jacoby GA. 2002. Mechanism of plasmid-mediated quinolone resistance. Proceedings of the National Academy of Sciences 99:5638-5642.
338. Arthur M, Reynolds P, Courvalin P. 1996. Glycopeptide resistance in enterococci. Trends in microbiology 4:401-407.
339. Putman M, van Veen HW, Konings WN. 2000. Molecular Properties of Bacterial Multidrug Transporters. Microbiology and Molecular Biology Reviews 64:672-693.
340. Fath MJ, Kolter R. 1993. ABC transporters: bacterial exporters. Microbiological Reviews 57:995-1017.
341. Kuroda T, Tsuchiya T. 2009. Multidrug efflux transporters in the MATE family. Biochim Biophys Acta 1794:763-8.
342. Lin HT, Bavro VN, Barrera NP, Frankish HM, Velamakanni S, van Veen HW, Robinson CV, Borges-Walmsley MI, Walmsley AR. 2009. MacB ABC Transporter Is a Dimer Whose ATPase Activity and Macrolide-binding Capacity Are Regulated by the Membrane Fusion Protein MacA. Journal of Biological Chemistry 284:1145-1154.
343. Huda N, Lee E-W, Chen J, Morita Y, Kuroda T, Mizushima T, Tsuchiya T. 2003. Molecular Cloning and Characterization of an ABC Multidrug Efflux Pump, VcaM, in Non-O1 Vibrio cholerae. Antimicrobial Agents and Chemotherapy 47:2413.
344. Ross JI, Eady EA, Cove JH, Baumberg S. 1995. Identification of a chromosomally encoded ABC-transport system with which the staphylococcal erythromycin exporter MsrA may interact. Gene 153:93-98.

345. El Garch F, Lismond A, Piddock LJV, Courvalin P, Tulkens PM, Van Bambeke F. 2010. Fluoroquinolones induce the expression of patA and patB, which encode ABC efflux pumps in Streptococcus pneumoniae. Journal of Antimicrobial Chemotherapy 65:2076-2082.
346. Steinfels E, Orelle C, Fantino J-R, Dalmas O, Rigaud J-L, Denizot F, Di Pietro A, Jault J-M. 2004. Characterization of YvcC (BmrA), a Multidrug ABC Transporter Constitutively Expressed in Bacillus subtilis†. Biochemistry 43:7491-7502.
347. van Veen HW, Margolles A, Muller M, Higgins CF, Konings WN. 2000. The homodimeric ATP-binding cassette transporter LmrA mediates multidrug transport by an alternating two-site (two-cylinder engine) mechanism. EMBO J 19:2503-2514.
348. Lubelski J, De Jong A, Van Merkerk R, Agustiandari H, Kuipers OP, Kok J, Driessen AJM. 2006. LmrCD is a major multidrug resistance transporter in Lactococcus lactis. Molecular Microbiology 61:771-781.
349. Sakamoto K, Margolles A, van Veen HW, Konings WN. 2001. Hop Resistance in the Beer Spoilage Bacterium Lactobacillus brevis Is Mediated by the ATP-Binding Cassette Multidrug Transporter HorA. Journal of Bacteriology 183:5371-5375.
350. Al-Hamad A, Upton M, Burnie J. 2009. Molecular cloning and characterization of SmrA, a novel ABC multidrug efflux pump from Stenotrophomonas maltophilia. Journal of Antimicrobial Chemotherapy 64:731-734.
351. Edgar R, Bibi E. 1997. MdfA, an Escherichia coli multidrug resistance protein with an extraordinarily broad spectrum of drug recognition. Journal of Bacteriology 179:2274-80.
352. Yoshida H, Bogaki M, Nakamura S, Ubukata K, Konno M. 1990. Nucleotide sequence and characterization of the Staphylococcus aureus norA gene, which confers resistance to quinolones. Journal of Bacteriology 172:6942-6949.
353. Bissonnette L, Champetier S, Buisson JP, Roy PH. 1991. Characterization of the nonenzymatic chloramphenicol resistance (cmlA) gene of the In4 integron of Tn1696: similarity of the product to transmembrane transport proteins. Journal of Bacteriology 173:4493-4502.
354. Clancy J, Petitpas J, Dib-Hajj F, Yuan W, Cronan M, Kamath AV, Bergeron J, Retsema JA. 1996. Molecular cloning and functional analysis of a novel macrolide-resistance determinant, mefA, from Streptococcus pyogenes. Molecular Microbiology 22:867-879.
355. Arpin C, Canron M-H, Noury P, Quentin C. 1999. Emergence of mefA and mefE genes in beta-haemolytic streptococci and pneumococci in France. Journal of Antimicrobial Chemotherapy 44:133-134.
356. Gill MJ, Brenwald NP, Wise R. 1999. Identification of an Efflux Pump Gene, pmrA, Associated with Fluoroquinolone Resistance in Streptococcus pneumoniae. Antimicrobial Agents and Chemotherapy 43:187-189.
357. Lomovskaya O, Lewis K. 1992. Emr, an Escherichia coli locus for multidrug resistance. Proceedings of the National Academy of Sciences 89:8938-8942.
358. White DG, Hudson C, Maurer JJ, Ayers S, Zhao S, Lee MD, Bolton L, Foley T, Sherwood J. 2000. Characterization of Chloramphenicol and Florfenicol Resistance in Escherichia coli Associated with Bovine Diarrhea. Journal of Clinical Microbiology 38:4593-4598.
359. Jonas BM, Murray BE, Weinstock GM. 2001. Characterization of emeA, anorA Homolog and Multidrug Resistance Efflux Pump, inEnterococcus faecalis. Antimicrobial Agents and Chemotherapy 45:3574-3579.
360. Ohki R, Murata M. 1997. bmr3, a third multidrug transporter gene of Bacillus subtilis. Journal of Bacteriology 179:1423-7.
361. Ahmed M, Lyass L, Markham PN, Taylor SS, Vázquez-Laslop N, Neyfakh AA. 1995. Two highly similar multidrug transporters of Bacillus subtilis whose expression is differentially regulated. Journal of Bacteriology 177:3904-10.

362. Anthonisen I-L, Sunde M, Steinum TM, Sidhu MS, Sørum H. 2002. Organization of the Antiseptic Resistance Gene qacA and Tn552-Related β-Lactamase Genes in Multidrug- Resistant Staphylococcus haemolyticus Strains of Animal and Human Origins. Antimicrobial Agents and Chemotherapy 46:3606-3612.
363. Kim H-J, Kim Y-H, Lee M-S, Lee H-S. 2001. Gene lmrb of Corynebacterium glutamicum confers efflux-mediated resistance to lincomycin, vol 12. Springer, Singapore, SINGAPOUR.
364. Yin Y, He X, Szewczyk P, Nguyen T, Chang G. 2006. Structure of the Multidrug Transporter EmrD from Escherichia coli. Science 312:741-744.
365. Smith K, Kumar S, Varela M. 2009. Identification, cloning, and functional characterization of EmrD-3, a putative multidrug efflux pump of the major facilitator superfamily from <i>Vibrio cholerae</i> O395. Archives of Microbiology 191:903-911.
366. Yamane K, Wachino J-i, Suzuki S, Kimura K, Shibata N, Kato H, Shibayama K, Konda T, Arakawa Y. 2007. New Plasmid-Mediated Fluoroquinolone Efflux Pump, QepA, Found in an Escherichia coli Clinical Isolate. Antimicrobial Agents and Chemotherapy 51:3354-3360.
367. Yin C-C, Aldema-Ramos ML, Borges-Walmsley MI, Taylor RW, Walmsley AR, Levy SB, Bullough PA. 2000. The quarternary molecular architecture of TetA, a secondary tetracycline transporter from Escherichia coli. Molecular Microbiology 38:482-492.
368. Guay GG, Tuckman M, Rothstein DM. 1994. Mutations in the tetA(B) gene that cause a change in substrate specificity of the tetracycline efflux pump. Antimicrobial Agents and Chemotherapy 38:857-860.
369. Braus G, Argast M, Beck CF. 1984. Identification of additional genes on transposon Tn10: tetC and tetD. Journal of Bacteriology 160:504-509.
370. Ginn SL, Brown MH, Skurray RA. 2000. The TetA(K) Tetracycline/H+ Antiporter from Staphylococcus aureus: Mutagenesis and Functional Analysis of Motif C. Journal of Bacteriology 182:1492-1498.
371. Guffanti AA, Krulwich TA. 1995. Tetracycline/H+ antiport and Na+/H+ antiport catalyzed by the Bacillus subtilis TetA(L) transporter expressed in Escherichia coli. Journal of Bacteriology 177:4557-61.
372. Kaatz GW, DeMarco CE, Seo SM. 2006. MepR, a Repressor of the Staphylococcus aureus MATE Family Multidrug Efflux Pump MepA, Is a Substrate-Responsive Regulatory Protein. Antimicrobial Agents and Chemotherapy 50:1276-1281.
373. Huda MN, Chen J, Morita Y, Kuroda T, Mizushima T, Tsuchiya T. 2003. Gene cloning and characterization of VcrM, a Na+-coupled multidrug efflux pump, from Vibrio cholerae non-O1. Microbiology and immunology 47:419-427.
374. Su X-Z, Chen J, Mizushima T, Kuroda T, Tsuchiya T. 2005. AbeM, an H+-Coupled Acinetobacter baumannii Multidrug Efflux Pump Belonging to the MATE Family of Transporters. Antimicrobial Agents and Chemotherapy 49:4362-4364.
375. BEGUM, #160, Anowara, RAHMAN M, M., OGAWA, Wakano, MIZUSHIMA, Tohru, KURODA, Teruo, TSUCHIYA, Tomofusa. 2005. Gene cloning and characterization of four MATE family multidrug efflux pumps from Vibrio cholerae non-O1, vol 49. Wiley-Blackwell, Tokyo, JAPON.
376. Brown MH, Paulsen IT, Skurray RA. 1999. The multidrug efflux protein NorM is a prototype of a new family of transporters. Molecular Microbiology 31:394-395.
377. Xu XJ, Su XZ, Morita Y, Kuroda T, Mizushima T, Tsuchiya T. 2003. Molecular cloning and characterization of the HmrM multidrug efflux pump from Haemophilus influenzae Rd. Microbiology and immunology 47:937-943.
378. He G-X, Kuroda T, Mima T, Morita Y, Mizushima T, Tsuchiya T. 2004. An H+-Coupled Multidrug Efflux Pump, PmpM, a Member of the MATE Family of Transporters, from Pseudomonas aeruginosa. Journal of Bacteriology 186:262-265.

379. Okusu H, Ma D, Nikaido H. 1996. AcrAB efflux pump plays a major role in the antibiotic resistance phenotype of Escherichia coli multiple-antibiotic-resistance (Mar) mutants. Journal of Bacteriology 178:306-8.
380. Ruzin A, Keeney D, Bradford PA. 2007. AdeABC multidrug efflux pump is associated with decreased susceptibility to tigecycline in Acinetobacter calcoaceticus–Acinetobacter baumannii complex. Journal of Antimicrobial Chemotherapy 59:1001-1004.
381. Chau S-L, Chu Y-W, Houang ETS. 2004. Novel Resistance-Nodulation-Cell Division Efflux System AdeDE in Acinetobacter Genomic DNA Group 3. Antimicrobial Agents and Chemotherapy 48:4054-4055.
382. Chu YW, Chau SL, Houang ETS. 2006. Presence of active efflux systems AdeABC, AdeDE and AdeXYZ in different Acinetobacter genomic DNA groups. Journal of Medical Microbiology 55:477-478.
383. Coyne S, Rosenfeld N, Lambert T, Courvalin P, Périchon B. 2010. Overexpression of Resistance-Nodulation-Cell Division Pump AdeFGH Confers Multidrug Resistance in Acinetobacter baumannii. Antimicrobial Agents and Chemotherapy 54:4389-4393.
384. Damier-Piolle L, Magnet S, Brémont S, Lambert T, Courvalin P. 2008. AdeIJK, a Resistance-Nodulation-Cell Division Pump Effluxing Multiple Antibiotics in Acinetobacter baumannii. Antimicrobial Agents and Chemotherapy 52:557-562.
385. Bunikis I, Denker K, Östberg Y, Andersen C, Benz R, Bergström S. 2008. An RND-Type Efflux System in Borrelia burgdorferi Is Involved in Virulence and Resistance to Antimicrobial Compounds. PLoS Pathog 4:e1000009.
386. Kumar A, Chua K-L, Schweizer HP. 2006. Method for Regulated Expression of Single-Copy Efflux Pump Genes in a Surrogate Pseudomonas aeruginosa Strain: Identification of the BpeEF-OprC Chloramphenicol and Trimethoprim Efflux Pump of Burkholderia pseudomallei 1026b. Antimicrobial Agents and Chemotherapy 50:3460-3463.
387. Lin J, Michel LO, Zhang Q. 2002. CmeABC Functions as a Multidrug Efflux System in Campylobacter jejuni. Antimicrobial Agents and Chemotherapy 46:2124-2131.
388. Pumbwe L, Randall LP, Woodward MJ, Piddock LJV. 2005. Evidence for Multiple-Antibiotic Resistance in Campylobacter jejuni Not Mediated by CmeB or CmeF. Antimicrobial Agents and Chemotherapy 49:1289-1293.
389. Li XZ, Nikaido H, Poole K. 1995. Role of mexA-mexB-oprM in antibiotic efflux in Pseudomonas aeruginosa. Antimicrobial Agents and Chemotherapy 39:1948-53.
390. Morita Y, Murata T, Mima T, Shiota S, Kuroda T, Mizushima T, Gotoh N, Nishino T, Tsuchiya T. 2003. Induction of mexCD-oprJ operon for a multidrug efflux pump by disinfectants in wild-type Pseudomonas aeruginosa PAO1. Journal of Antimicrobial Chemotherapy 51:991-994.
391. Maseda H, Yoneyama H, Nakae T. 2000. Assignment of the Substrate-Selective Subunits of the MexEF-OprN Multidrug Efflux Pump of Pseudomonas aeruginosa. Antimicrobial Agents and Chemotherapy 44:658-664.
392. Chuanchuen R, Narasaki CT, Schweizer HP. 2002. The MexJK Efflux Pump of Pseudomonas aeruginosa Requires OprM for Antibiotic Efflux but Not for Efflux of Triclosan. Journal of Bacteriology 184:5036-5044.
393. Mine T, Morita Y, Kataoka A, Mizushima T, Tsuchiya T. 1999. Expression in Escherichia coli of a New Multidrug Efflux Pump, MexXY, from Pseudomonas aeruginosa. Antimicrobial Agents and Chemotherapy 43:415-417.
394. Sekiya H, Mima T, Morita Y, Kuroda T, Mizushima T, Tsuchiya T. 2003. Functional Cloning and Characterization of a Multidrug Efflux Pump, MexHI-OpmD, from a Pseudomonas aeruginosa Mutant. Antimicrobial Agents and Chemotherapy 47:2990-2992.

395. Li Y, Mima T, Komori Y, Morita Y, Kuroda T, Mizushima T, Tsuchiya T. 2003. A new member of the tripartite multidrug efflux pumps, MexVW–OprM, in Pseudomonas aeruginosa. Journal of Antimicrobial Chemotherapy 52:572-575.
396. Takehiko M, Hiroshi S, Tohru M, Teruo K, Tomofusa T. 2005. Gene Cloning and Properties of the RND-Type Multidrug Efflux Pumps MexPQ-OpmE and MexMN-OprM from Pseudomonas aeruginosa. Microbiology and Immunology 49:999-1002.
397. Chang L-L, Chen H-F, Chang C-Y, Lee T-M, Wu W-J. 2004. Contribution of integrons, and SmeABC and SmeDEF efflux pumps to multidrug resistance in clinical isolates of Stenotrophomonas maltophilia. Journal of Antimicrobial Chemotherapy 53:518-521.
398. Alonso A, Martínez JL. Cloning and Characterization of SmeDEF, a Novel Multidrug Efflux Pump from Stenotrophomonas maltophilia. American Society for Microbiology.
399. Kumar A, Worobec EA. 2005. Cloning, Sequencing, and Characterization of the SdeAB Multidrug Efflux Pump of Serratia marcescens. Antimicrobial Agents and Chemotherapy 49:1495-1501.
400. Begic S, Worobec EA. 2008. Characterization of the Serratia marcescens SdeCDE multidrug efflux pump studied via gene knockout mutagenesis. Canadian Journal of Microbiology 54:411-416.
401. Chen J, Kuroda T, Huda MN, Mizushima T, Tsuchiya T. 2003. An RND-type multidrug efflux pump SdeXY from Serratia marcescens. Journal of Antimicrobial Chemotherapy 52:176-179.
402. Hagman KE, Pan W, Spratt BG, Balthazar JT, Judd RC, Shafer WM. 1995. Resistance of Neisseria gonorrhoeae to antimicrobial hydrophobic agents is modulated by the mtrRCDE efflux system. Microbiology 141:611-622.
403. Burns JL, Wadsworth CD, Barry JJ, Goodall CP. 1996. Nucleotide sequence analysis of a gene from Burkholderia (Pseudomonas) cepacia encoding an outer membrane lipoprotein involved in multiple antibiotic resistance. Antimicrobial Agents and Chemotherapy 40:307-13.
404. Jude F, Arpin C, Brachet-Castang C, Capdepuy M, Caumette P, Quentin C. 2004. TbtABM, a multidrug efflux pump associated with tributyltin resistance in Pseudomonas stutzeri. FEMS Microbiology Letters 232:7-14.
405. Gristwood T, Fineran PC, Everson L, Salmond GPC. 2008. PigZ, a TetR/AcrR family repressor, modulates secondary metabolism via the expression of a putative four-component resistance-nodulation-cell-division efflux pump, ZrpADBC, in Serratia sp. ATCC 39006. Molecular Microbiology 69:418-435.
406. Bador J, Amoureux L, Duez J-M, Drabowicz A, Siebor E, Llanes C, Neuwirth C. 2011. First Description of an RND-Type Multidrug Efflux Pump in Achromobacter xylosoxidans, AxyABM. Antimicrobial Agents and Chemotherapy 55:4912-4914.
407. Lindemann A, Koch M, Pessi G, Müller AJ, Balsiger S, Hennecke H, Fischer H-M. 2010. Host-specific symbiotic requirement of BdeAB, a RegR-controlled RND-type efflux system in Bradyrhizobium japonicum. FEMS Microbiology Letters 312:184-191.
408. Chan YY, Tan TMC, Ong YM, Chua KL. 2004. BpeAB-OprB, a Multidrug Efflux Pump in Burkholderia pseudomallei. Antimicrobial Agents and Chemotherapy 48:1128-1135.
409. Bina J, Provenzano D, Wang C, Bina X, Mekalanos J. 2006. Characterization of the Vibrio cholerae vexAB and vexCD efflux systems. Archives of Microbiology 186:171-181.
410. Hernould M, Gagné S, Fournier M, Quentin C, Arpin C. 2008. Role of the AheABC Efflux Pump in Aeromonas hydrophila Intrinsic Multidrug Resistance. Antimicrobial Agents and Chemotherapy 52:1559-1563.
411. Terán W, Felipe A, Segura A, Rojas A, Ramos J-L, Gallegos M-T. 2003. Antibiotic-Dependent Induction of Pseudomonas putida DOT-T1E TtgABC Efflux Pump Is Mediated by the Drug Binding Repressor TtgR. Antimicrobial Agents and Chemotherapy 47:3067-3072.

412. Matsuo T, Hayashi K, Morita Y, Koterasawa M, Ogawa W, Mizushima T, Tsuchiya T, Kuroda T. 2007. VmeAB, an RND-type multidrug efflux transporter in Vibrio parahaemolyticus. Microbiology 153:4129-4137.
413. De Rossi E, Branzoni M, Cantoni R, Milano A, Riccardi G, Ciferri O. 1998. mmr, a Mycobacterium tuberculosis Gene Conferring Resistance to Small Cationic Dyes and Inhibitors. Journal of Bacteriology 180:6068-6071.
414. Morimyo M, Hongo E, Hama-Inaba H, Machida I. 1992. Cloning and characterization of the mvrC gene of Escherichia coli K-12 which confers resistance against methyl viologen toxicity. Nucleic Acids Research 20:3159-3165.
415. Kazama H, Hamashima H, Sasatsu M, Arai T. 1998. Distribution of the antiseptic-resistance gene qacEΔ1 in Gram-positive bacteria. FEMS Microbiology Letters 165:295-299.
416. Mayer S, Boos M, Beyer A, Fluit AC, Schmitz F-J. 2001. Distribution of the antiseptic resistance genes qacA, qacB and qacC in 497 methicillin-resistant and -susceptible European isolates of Staphylococcus aureus. Journal of Antimicrobial Chemotherapy 47:896-897.
417. Ploy M-C, Courvalin P, Lambert T. 1998. Characterization of In40 of Enterobacter aerogenes BM2688, a Class 1 Integron with Two New Gene Cassettes, cmlA2 and qacF. Antimicrobial Agents and Chemotherapy 42:2557-2563.
418. Heir E, Sundheim G, Holck AL. 1999. The qacG gene on plasmid pST94 confers resistance to quaternary ammonium compounds in staphylococci isolated from the food industry. Journal of Applied Microbiology 86:378-388.
419. Bjorland J, Steinum T, Sunde M, Waage S, Heir E. 2003. Novel Plasmid-Borne Gene qacJ Mediates Resistance to Quaternary Ammonium Compounds in Equine Staphylococcus aureus, Staphylococcus simulans, and Staphylococcus intermedius. Antimicrobial Agents and Chemotherapy 47:3046-3052.
420. Paulsen IT, Brown MH, Dunstan SJ, Skurray RA. 1995. Molecular characterization of the staphylococcal multidrug resistance export protein QacC. Journal of Bacteriology 177:2827-33.
421. Braga TM, Marujo PE, Pomba C, Lopes MFS. 2011. Involvement, and dissemination, of the enterococcal small multidrug resistance transporter QacZ in resistance to quaternary ammonium compounds. Journal of Antimicrobial Chemotherapy 66:283-286.
422. Srinivasan VB, Rajamohan G, Gebreyes WA. 2009. Role of AbeS, a Novel Efflux Pump of the SMR Family of Transporters, in Resistance to Antimicrobial Agents in Acinetobacter baumannii. Antimicrobial Agents and Chemotherapy 53:5312-5316.
423. Heir E, Sundheim G, Holck AL. 1998. The Staphylococcus qacH gene product: a new member of the SMR family encoding multidrug resistance. FEMS Microbiology Letters 163:49-56.
424. Minato Y, Shahcheraghi F, Ogawa W, Kuroda T, Tsuchiya T. 2008. Functional Gene Cloning and Characterization of the SsmE Multidrug Efflux Pump from <i>Serratia marcescens</i>. Biological and Pharmaceutical Bulletin 31:516-519.
425. Bjorland J, Sunde M, Waage S. 2001. Plasmid-Borne smr Gene Causes Resistance to Quaternary Ammonium Compounds in BovineStaphylococcus aureus. Journal of Clinical Microbiology 39:3999-4004.
426. Masaoka Y, Ueno Y, Morita Y, Kuroda T, Mizushima T, Tsuchiya T. 2000. A Two-Component Multidrug Efflux Pump, EbrAB, in Bacillus subtilis. Journal of Bacteriology 182:2307-2310.
427. Nishino K, Yamaguchi A. 2001. Analysis of a Complete Library of Putative Drug Transporter Genes in Escherichia coli. Journal of Bacteriology 183:5803-5812.
428. Higashi K, Ishigure H, Demizu R, Uemura T, Nishino K, Yamaguchi A, Kashiwagi K, Igarashi K. 2008. Identification of a Spermidine Excretion Protein Complex (MdtJI) in Escherichia coli. Journal of Bacteriology 190:872-878.

429. Bay D, Turner R. 2009. Diversity and evolution of the small multidrug resistance protein family. BioMed Central.
430. Bush K, Jacoby GA. 2010. Updated functional classification of beta-lactamases. Antimicrob Agents Chemother 54:969-76.
431. Sutcliffe JG. 1978. Nucleotide sequence of the ampicillin resistance gene of Escherichia coli plasmid pBR322.
432. Rasmussen BA, Gluzman Y, Tally FP. 1990. Cloning and sequencing of the class B beta-lactamase gene (ccrA) from Bacteroides fragilis TAL3636. Antimicrobial Agents and Chemotherapy 34:1590-1592.
433. Knott-Hunziker V, Petursson S, Waley SG, Jaurin B, Grundström T. 1982. The acyl-enzyme mechanism of beta-lactamase action. The evidence for class C Beta-lactamases.
434. Ledent P, Raquet X, Joris B, Van Beeumen J, Frère JM. 1993. A comparative study of class-D beta-lactamases. The Biochemical journal 292 (Pt 2):555-562.
435. Dutka-Malen S, Molinas C, Arthur M, Courvalin P. 1992. Sequence of the vanC gene of Enterococcus gallinarum BM4174 encoding a d-alanine:d-alanine ligase-related protein necessary for vancomycin resistance. Gene 112:53-58.
436. Allignet J, Loncle V, Mazodier P, El Solh N. 1988. Nucleotide sequence of a staphylococcal plasmid gene, vgb, encoding a hydrolase inactivating the B components of virginiamycin-like antibiotics. Plasmid 20:271-275.
437. Bernat BA, Laughlin LT, Armstrong RN. 1997. Fosfomycin Resistance Protein (FosA) Is a Manganese Metalloglutathione Transferase Related to Glyoxalase I and the Extradiol Dioxygenases. Biochemistry 36:3050-3055.
438. Zilhao R, Courvalin P. 1990. Nucleotide sequence of the fosB gene conferring fosfomycin resistance in Staphylococcus epidermidis. FEMS Microbiology Letters 68:267-272.
439. Fillgrove KL, Pakhomova S, Schaab MR, Newcomer ME, Armstrong RN. 2007. Structure and Mechanism of the Genomically Encoded Fosfomycin Resistance Protein, FosX, from Listeria monocytogenes. Biochemistry 46:8110-8120.
440. Li X, He Z, Zhou J. 2005. Selection of optimal oligonucleotide probes for microarrays using multiple criteria, global alignment and parameter estimation. Nucleic Acids Res 33:6114-23.
441. Speer BS, Bedzyk L, Salyers AA. 1991. Evidence that a novel tetracycline resistance gene found on two Bacteroides transposons encodes an NADP-requiring oxidoreductase. Journal of Bacteriology 173:176-183.
442. Brown JT, Roberts MC. 1987. Cloning and characterization of tetM gene from a Ureaplasma urealyticum strain. Antimicrobial Agents and Chemotherapy 31:1852-1854.
443. Sougakoff W, Papadopoulou B, Nordmann P, Courvalin P. 1987. Nucleotide sequence and distribution of gene tetO encoding tetracycline resistance in Campylobacter coli. FEMS Microbiology Letters 44:153-159.
444. Nikolich MP, Hong G, Shoemaker NB, Salyers AA. 1994. Evidence for natural horizontal transfer of tetQ between bacteria that normally colonize humans and bacteria that normally colonize livestock. Applied and Environmental Microbiology 60:3255-3260.
445. Scott KP, Barbosa TM, Forbes KJ, Flint HJ. 1997. High-frequency transfer of a naturally occurring chromosomal tetracycline resistance element in the ruminal anaerobe Butyrivibrio fibrisolvens. Applied and Environmental Microbiology 63:3405-11.
446. Charpentier E, Gerbaud G, Courvalin P. 1993. Characterization of a new class of tetracycline-resistance gene tet(S) in Listeria monocytogenes BM4210. Gene 131:27-34.
447. Doyle D, McDowall KJ, Butler MJ, Hunter IS. 1991. Characterization of an oxytetracycline-resistance gene, otrA, of Streptomyces rimosus. Molecular Microbiology 5:2923-2933.
448. Aminov RI. 2013. Biotic acts of antibiotics. Frontiers in microbiology 4:241.

449. Pierson L, III, Pierson E. 2010. Metabolism and function of phenazines in bacteria: impacts on the behavior of bacteria in the environment and biotechnological processes. Applied Microbiology and Biotechnology 86:1659-1670.
450. van Pée KH, Ligon JM. 2000. Biosynthesis of pyrrolnitrin and other phenylpyrrole derivatives by bacteria. Natural Product Reports 17:157-164.
451. Wever R. 2012. Structure and Function of Vanadium Haloperoxidases, p 95-125. *In* Michibata H (ed), Vanadium doi:10.1007/978-94-007-0913-3_5. Springer Netherlands.
452. Ramette A, Frapolli M, Défago G, Moënne-Loccoz Y. 2003. Phylogeny of HCN synthase-encoding hcnBC genes in biocontrol fluorescent pseudomonads and its relationship with host plant species and HCN synthesis ability. Molecular plant-microbe interactions : MPMI 16:525-535.
453. Bhaya D, Davison M, Barrangou R. 2011. CRISPR-Cas Systems in Bacteria and Archaea: Versatile Small RNAs for Adaptive Defense and Regulation. Annual Review of Genetics 45:273-297.
454. Makarova KS, Haft DH, Barrangou R, Brouns SJJ, Charpentier E, Horvath P, Moineau S, Mojica FJM, Wolf YI, Yakunin AF, van der Oost J, Koonin EV. 2011. Evolution and classification of the CRISPR–Cas systems. Nat Rev Micro 9:467-477.
455. Makarova K, Koonin E. 2013. Evolution and Classification of CRISPR-Cas Systems and Cas Protein Families, p 61-91. *In* Barrangou R, van der Oost J (ed), CRISPR-Cas Systems doi:10.1007/978-3-642-34657-6_3. Springer Berlin Heidelberg.
456. Van Dolah FM. 2000. Marine algal toxins: origins, health effects, and their increased occurrence. Environ Health Perspect 108 Suppl 1:133-41.
457. Cusick KD, Sayler GS. 2013. An overview on the marine neurotoxin, saxitoxin: genetics, molecular targets, methods of detection and ecological functions. Mar Drugs 11:991-1018.
458. Dawson RM. 1998. The toxicology of microcystins. Toxicon 36:953-62.
459. Singh A, Wyant T, Anaya-Bergman C, Aduse-Opoku J, Brunner J, Laine ML, Curtis MA, Lewis JP. 2011. The capsule of Porphyromonas gingivalis leads to a reduction in the host inflammatory response, evasion of phagocytosis, and increase in virulence. Infection and immunity 79:4533-4542.
460. Galán JE, Collmer A. 1999. Type III secretion machines: bacterial devices for protein delivery into host cells. Science 284:1322-1328.
461. Kline KA, Falker S, Dahlberg S, Normark S, Henriques-Normark B. 2009. Bacterial adhesins in host-microbe interactions. Cell Host Microbe 5:580-92.
462. Yang S, Bourne PE. 2009. The evolutionary history of protein domains viewed by species phylogeny. PLoS One 4:e8378.
463. Carter MQ, Chen J, Lory S. 2010. The Pseudomonas aeruginosa pathogenicity island PAPI-1 is transferred via a novel type IV pilus. J Bacteriol 192:3249-58.
464. Craig L, Taylor RK, Pique ME, Adair BD, Arvai AS, Singh M, Lloyd SJ, Shin DS, Getzoff ED, Yeager M, Forest KT, Tainer JA. 2003. Type IV pilin structure and assembly: X-ray and EM analyses of Vibrio cholerae toxin-coregulated pilus and Pseudomonas aeruginosa PAK pilin. Mol Cell 11:1139-50.
465. Blomfield IC, Calie PJ, Eberhardt KJ, McClain MS, Eisenstein BI. 1993. Lrp stimulates phase variation of type 1 fimbriation in Escherichia coli K-12. J Bacteriol 175:27-36.
466. Tobias J, Svennerholm AM. 2012. Strategies to overexpress enterotoxigenic Escherichia coli (ETEC) colonization factors for the construction of oral whole-cell inactivated ETEC vaccine candidates. Appl Microbiol Biotechnol 93:2291-300.
467. Cossart P, Jonquieres R. 2000. Sortase, a universal target for therapeutic agents against gram-positive bacteria? Proc Natl Acad Sci U S A 97:5013-5.
468. Mazmanian SK, Ton-That H, Schneewind O. 2001. Sortase-catalysed anchoring of surface proteins to the cell wall of Staphylococcus aureus. Mol Microbiol 40:1049-57.

469. Galan JE, Collmer A. 1999. Type III secretion machines: bacterial devices for protein delivery into host cells. Science 284:1322-8.
470. Isberg RR, Voorhis DL, Falkow S. 1987. Identification of invasin: a protein that allows enteric bacteria to penetrate cultured mammalian cells. Cell 50:769-78.
471. Vellios E, Duncan G, Brown D, MacFarlane S. 2002. Immunogold localization of tobravirus 2b nematode transmission helper protein associated with virus particles. Virology 300:118-24.
472. Rahim MD, Andika IB, Han C, Kondo H, Tamada T. 2007. RNA4-encoded p31 of beet necrotic yellow vein virus is involved in efficient vector transmission, symptom severity and silencing suppression in roots. Journal of General Virology 88:1611-1619.
473. Marcos JF, Vilar M, Pérez-Payá E, Pallás V. 1999. In VivoDetection, RNA-Binding Properties and Characterization of the RNA-Binding Domain of the p7 Putative Movement Protein from Carnation Mottle Carmovirus (CarMV). Virology 255:354-365.
474. Takeda A, Mise K, Okuno T. 2005. RNA silencing suppressors encoded by viruses of the family Tombusviridae. Plant Biotechnology 22:447-454.
475. Russell WC. 2009. Adenoviruses: update on structure and function. Journal of General Virology 90:1-20.
476. Stevenson SC, Rollence M, White B, Weaver L, McClelland A. 1995. Human adenovirus serotypes 3 and 5 bind to two different cellular receptors via the fiber head domain. Journal of Virology 69:2850-7.
477. Allard A, Girones R, Juto P, Wadell G. 1990. Polymerase chain reaction for detection of adenoviruses in stool samples. Journal of Clinical Microbiology 28:2659-2667.
478. Ali M, Seto D. Phylogenetic analysis of hexon and DNA polymerase genes from selected serotypes of human adenoviruses, using bioinformatics tools, p 107-114. *In* (ed),
479. Jothikumar N, Cromeans TL, Hill VR, Lu X, Sobsey MD, Erdman DD. 2005. Quantitative Real-Time PCR Assays for Detection of Human Adenoviruses and Identification of Serotypes 40 and 41. Applied and Environmental Microbiology 71:3131-3136.
480. Okada M, Ogawa T, Kubonoya H, Yoshizumi H, Shinozaki K. 2007. Detection and sequence-based typing of human adenoviruses using sensitive universal primer sets for the hexon gene. Archives of Virology 152:1-9.
481. Vigne E, Mahfouz I, Dedieu J-F, Brie A, Perricaudet M, Yeh P. 1999. RGD Inclusion in the Hexon Monomer Provides Adenovirus Type 5-Based Vectors with a Fiber Knob-Independent Pathway for Infection. Journal of Virology 73:5156-5161.
482. Crawford-Miksza L, Schnurr DP. 1996. Analysis of 15 adenovirus hexon proteins reveals the location and structure of seven hypervariable regions containing serotype-specific residues. Journal of Virology 70:1836-44.
483. Belliot G, Laveran H, Monroe SS. 1997. Detection and genetic differentiation of human astroviruses: phylogenetic grouping varies by coding region. Archives of Virology 142:1323-1334.
484. Sakamoto T, Negishi H, Wang Q-H, Akihara S, Kim B, Nishimura S, Kaneshi K, Nakaya S, Ueda Y, Sugita K, Motohiro T, Nishimura T, Ushijima H. 2000. Molecular epidemiology of astroviruses in Japan from 1995 to 1998 by reverse transcription-polymerase chain reaction with serotype-specific primers (1 to 8). Journal of Medical Virology 61:326-331.
485. Finkbeiner SR, Holtz LR, Jiang Y, Rajendran P, Franz CJ, Zhao G, Kang G, Wang D. 2009. Human stool contains a previously unrecognized diversity of novel astroviruses. Virol J 6:161.
486. Lu L, Li C, Hagedorn CH. 2006. Phylogenetic analysis of global hepatitis E virus sequences: genetic diversity, subtypes and zoonosis. Reviews in Medical Virology 16:5-36.
487. Kalia M, Chandra V, Rahman SA, Sehgal D, Jameel S. 2009. Heparan Sulfate Proteoglycans Are Required for Cellular Binding of the Hepatitis E Virus ORF2 Capsid Protein and for Viral Infection. Journal of Virology 83:12714-12724.

488. Liang J-H, Dai X, Dong C, Meng J-H. 2010. A Single Amino Acid Substitution Changes Antigenicity of ORF2-Encoded Proteins of Hepatitis E Virus. International Journal of Molecular Sciences 11:2962-2975.
489. Schlauder GG, Mushahwar IK. 2001. Genetic heterogeneity of hepatitis E virus*. Journal of Medical Virology 65:282-292.
490. Hardy ME. 2005. Norovirus protein structure and function. FEMS Microbiology Letters 253:1-8.
491. Donaldson EF, Lindesmith LC, LoBue AD, Baric RS. 2010. Viral shape-shifting: norovirus evasion of the human immune system. Nat Rev Micro 8:231-241.
492. Jiang M, Abend JR, Johnson SF, Imperiale MJ. 2009. The role of polyomaviruses in human disease. Virology 384:266-273.
493. Kean JM, Rao S, Wang M, Garcea RL. 2009. Seroepidemiology of Human Polyomaviruses. PLoS Pathog 5:e1000363.
494. SanJuan NA, Simula S, Casas J, Woscoff A. 2010. DETECTION OF POLYOMAVIRUS MAJOR CAPSID ANTIGEN (VP-1) IN HUMAN PILOMATRICOMAS, vol 70. Fundación Revista Medicina, Buenos Aires, ARGENTINE.
495. An P, Sáenz Robles MT, Pipas JM. 2012. Large T Antigens of Polyomaviruses: Amazing Molecular Machines. Annual Review of Microbiology 66:213-236.
496. Gouvea V, Santos N, Timenetsky MdC. 1994. Identification of bovine and porcine rotavirus G types by PCR. Journal of Clinical Microbiology 32:1338-1340.
497. Gentsch JR, Woods PA, Ramachandran M, Das BK, Leite JP, Alfieri A, Kumar R, Bhan MK, Glass RI. 1996. Review Of G And P Typing Results From A Global Collection Of Rotavirus Strains: Implications For Vaccine Development. Journal of Infectious Diseases 174:S30-S36.
498. Iturriza Gómara M, Wong C, Blome S, Desselberger U, Gray J. 2002. Molecular characterization of VP6 genes of human rotavirus isolates: correlation of genogroups with subgroups and evidence of independent segregation. Journal of Virology 76:6596-6601.
499. Dong Y, Zeng CQ-Y, Ball JM, Estes MK, Morris AP. 1997. The rotavirus enterotoxin NSP4 mobilizes intracellular calcium in human intestinal cells by stimulating phospholipase C-mediated inositol 1,4,5-trisphosphate production. Proceedings of the National Academy of Sciences 94:3960-3965.
500. Fregolente M, Gatti M. 2009. Nomenclature proposal for picobirnavirus. Archives of Virology 154:1953-1954.
501. van Leeuwen M, Williams MMW, Koraka P, Simon JH, Smits SL, Osterhaus ADME. 2010. Human Picobirnaviruses Identified by Molecular Screening of Diarrhea Samples. Journal of Clinical Microbiology 48:1787-1794.
502. Hamza IA, Jurzik L, Überla K, Wilhelm M. 2011. Evaluation of pepper mild mottle virus, human picobirnavirus and Torque teno virus as indicators of fecal contamination in river water. Water Research 45:1358-1368.
503. Bányai K, Jakab F, Reuter G, Bene J, Új M, Melegh B, Szücs G. 2003. Sequence heterogeneity among human picobirnaviruses detected in a gastroenteritis outbreak. Archives of Virology 148:2281-2291.
504. Bhattacharya R, Sahoo GC, Nayak MK, Saha DR, Sur D, Naik TN, Bhattacharya SK, Krishnan T. 2006. Molecular epidemiology of human picobirnaviruses among children of a slum community in Kolkata, India. Infection, Genetics and Evolution 6:453-458.
505. Cavanagh D. 2005. *Coronaviridae*: a review of coronaviruses and toroviruses

Coronaviruses with Special Emphasis on First Insights Concerning SARS, p 1-54. *In* Schmidt A, Weber O, Wolff M (ed) doi:10.1007/3-7643-7339-3_1. Birkhäuser Basel.

506. Neuman BW, Kiss G, Kunding AH, Bhella D, Baksh MF, Connelly S, Droese B, Klaus JP, Makino S, Sawicki SG, Siddell SG, Stamou DG, Wilson IA, Kuhn P, Buchmeier MJ. 2011. A structural analysis of M protein in coronavirus assembly and morphology. Journal of Structural Biology 174:11-22.
507. Gallagher T, Buchmeier M. 2001. Coronavirus Spike Proteins in Viral Entry and Pathogenesis. Virology 279:371-374.
508. Caron DA, Worden AZ, Countway PD, Demir E, Heidelberg KB. 2009. Protists are microbes too: a perspective. The ISME journal 3:4-12.
509. Adl M, Gupta VS. 2006. Protists in soil ecology and forest nutrient cycling. Canadian Journal of Forest Research 36:1805-1817.
510. Pawlowski J, Audic S, Adl S, Bass D, Belbahri L, Berney C, Bowser SS, Cepicka I, Decelle J, Dunthorn M, Fiore-Donno AM, Gile GH, Holzmann M, Jahn R, Jirků M, Keeling PJ, Kostka M, Kudryavtsev A, Lara E, Lukeš J, Mann DG, Mitchell EAD, Nitsche F, Romeralo M, Saunders GW, Simpson AGB, Smirnov AV, Spouge JL, Stern RF, Stoeck T, Zimmermann J, Schindel D, de Vargas C. 2012. CBOL Protist Working Group: Barcoding Eukaryotic Richness beyond the Animal, Plant, and Fungal Kingdoms. PLoS Biol 10:e1001419.
511. Harumoto T, Miyake A. 1991. Defensive Function of Trichocysts in Paramecium. Journal of Experimental Zoology 260:84-92.
512. Knoll G, Haacke-Bell B, Plattner H. 1991. Local trichocyst exocytosis provides an efficient escape mechanism for Paramecium cells. European Journal of Protistology 27:381-385.
513. Madeddu L, Gautier MC, Vayssie L, Houari A, Sperling L. 1995. A large multigene family codes for the polypeptides of the crystalline trichocyst matrix in Paramecium. Mol Biol Cell 6:649-59.
514. Portman N, Gull K. 2010. The paraflagellar rod of kinetoplastid parasites: From structure to components and function. International Journal for Parasitology 40:135-148.
515. Yu LM. 1995. Elicitins from Phytophthora and basic resistance in tobacco. Proc Natl Acad Sci U S A 92:4088-94.
516. Bos JIB, Armstrong M, Whisson SC, Torto TA, Ochwo M, Birch PRJ, Kamoun S. 2003. Intraspecific comparative genomics to identify avirulence genes from Phytophthora. New Phytologist 159:63-72.
517. Spanu P, Kamper J. 2010. Genomics of biotrophy in fungi and oomycetes - emerging patterns. Current Opinion in Plant Biology 13:409-414.
518. Richards TA, Soanes DM, Jones MDM, Vasieva O, Leonard G, Paszkiewicz K, Foster PG, Hall N, Talbot NJ. 2011. Horizontal gene transfer facilitated the evolution of plant parasitic mechanisms in the oomycetes. Proceedings of the National Academy of Sciences 108:15258-15263.
519. Rose JK, Ham KS, Darvill AG, Albersheim P. 2002. Molecular cloning and characterization of glucanase inhibitor proteins: coevolution of a counterdefense mechanism by plant pathogens. Plant Cell 14:1329-45.
520. Tian M, Win J, Song J, van der Hoorn R, van der Knaap E, Kamoun S. 2007. A Phytophthora infestans cystatin-like protein targets a novel tomato papain-like apoplastic protease. Plant Physiol 143:364-77.
521. Qutob D, Kamoun S, Gijzen M. 2002. Expression of a Phytophthora sojae necrosis-inducing protein occurs during transition from biotrophy to necrotrophy. Plant J 32:361-73.
522. Muñoz CI, Bailey AM. 1998. A cutinase-encoding gene from Phytophthora capsici isolated by differential-display RT-PCR. Current Genetics 33:225-230.
523. Gotesson A, Marshall JS, Jones DA, Hardham AR. 2002. Characterization and evolutionary analysis of a large polygalacturonase gene family in the oomycete plant pathogen Phytophthora cinnamomi. Molecular plant-microbe interactions : MPMI 15:907-21.
524. Cavalier-Smith T. 2003. Protist phylogeny and the high-level classification of Protozoa. European Journal of Protistology 39:338-348.

525. Brodmann A, Schuller A, Ludwig-Muller J, Aeschbacher RA, Wiemken A, Boller T, Wingler A. 2002. Induction of trehalase in Arabidopsis plants infected with the trehalose-producing pathogen Plasmodiophora brassicae. Mol Plant Microbe Interact 15:693-700.
526. Nakashima KI, Watanabe H, Azuma JI. 2002. Cellulase genes from the parabasalian symbiont Pseudotrichonympha grassii in the hindgut of the wood-feeding termite Coptotermes formosanus. Cell Mol Life Sci 59:1554-60.
527. Bera-Maillet C, Devillard E, Cezette M, Jouany JP, Forano E. 2005. Xylanases and carboxymethylcellulases of the rumen protozoa Polyplastron multivesiculatum, Eudiplodinium maggii and Entodinium sp. FEMS Microbiol Lett 244:149-56.
528. Tabita FR, Satagopan S, Hanson TE, Kreel NE, Scott SS. 2008. Distinct form I, II, III, and IV Rubisco proteins from the three kingdoms of life provide clues about Rubisco evolution and structure/function relationships. Journal of Experimental Botany 59:1515-1524.
529. Medlin LK, Kooistra WHCF. 2010. Methods to Estimate the Diversity in the Marine Photosynthetic Protist Community with Illustrations from Case Studies: A Review. Diversity 2:973-1014.
530. Raven JA. 2010. Inorganic carbon acquisition by eukaryotic algae: four current questions. Photosynth Res 106:123-34.
531. Guerrero MG, Vega JM, Losada M. 1981. The Assimilatory Nitrate-Reducing System and Its Regulation. Annual Review of Plant Physiology and Plant Molecular Biology 32:169-204.
532. Waser NAD, Harrison PJ, Nielsen B, Calvert SE, Turpin DH. 1998. Nitrogen Isotope Fractionation During the Uptake and Assimilation of Nitrate, Nitrite, Ammonium, and Urea by a Marine Diatom. Limnology and Oceanography 43:215-224.
533. Inokuchi R, Kuma K-i, Miyata T, Okada M. 2002. Nitrogen-assimilating enzymes in land plants and algae: phylogenic and physiological perspectives. Physiologia Plantarum 116:1-11.
534. Giordano M, Norici A, Ratti S, Raven JA. 2008. Role of Sulfur for Algae: Acquisition, Metabolism, Ecology and Evolution

Sulfur Metabolism in Phototrophic Organisms, p 397-415. *In* Hell R, Dahl C, Knaff D, Leustek T (ed), vol 27. Springer Netherlands.
535. Takahashi H, Kopriva S, Giordano M, Saito K, Hell R. 2011. Sulfur Assimilation in Photosynthetic Organisms: Molecular Functions and Regulations of Transporters and Assimilatory Enzymes. Annual Review of Plant Biology 62:157-184.
536. Pilsyk S, Paszewski A. 2009. Sulfate permeases - phylogenetic diversity of sulfate transport. Acta Biochim Pol 56:375-84.
537. Perry CC. 2003. Silicification: the processes by which organisms capture and mineralize silica. Reviews in Mineralogy and Geochemistry 54:291-327.
538. Descles J, Vartanian M, El Harrak A, Quinet M, Bremond N, Sapriel G, Bibette J, Lopez PJ. 2008. New tools for labeling silica in living diatoms. New Phytol 177:822-9.
539. Cornelis JT, Delvaux B, Cardinal D, Andre L, Ranger J, Opfergelt S. 2010. Tracing mechanisms controlling the release of dissolved silicon in forest soil solutions using Si isotopes and Ge/Si ratios. Geochimica Et Cosmochimica Acta 74:3913-3924.
540. Lombi E, Holm PE. 2010. Metalloids, soil chemistry and the environment. Adv Exp Med Biol 679:33-44.
541. Sumper M, Kroger N. 2004. Silica formation in diatoms: the function of long-chain polyamines and silaffins. Journal of Materials Chemistry 14:2059-2065.
542. Hildebrand M, Dahlin K, Volcani BE. 1998. Characterization of a silicon transporter gene family in <i>Cylindrotheca fusiformis</i> : sequences, expression analysis, and identification of homologs in other diatoms. Molecular and General Genetics MGG 260:480-486.

543. Lechner CC, Becker CF. 2015. Silaffins in silica biomineralization and biomimetic silica precipitation. Marine drugs 13:5297-5333.
544. Suzuki JY, Bollivar DW, Bauer CE. 1997. Genetic analysis of chlorophyll biosynthesis. Annu Rev Genet 31:61-89.
545. Britton G. 1995. Structure and properties of carotenoids in relation to function. The FASEB Journal 9:1551-1558.
546. Dimopoulos M, Bagnara AS, Edwards MR. 2000. Characterisation and Sequence Analysis of a Carbamate Kinase Gene from the Diplomonad Hexamita inflata1. Journal of Eukaryotic Microbiology 47:499-503.
547. Minotto L, Edwards MR, Bagnara AS. 2000. Trichomonas vaginalis: characterization, expression, and phylogenetic analysis of a carbamate kinase gene sequence. Exp Parasitol 95:54-62.
548. Shakoori AR, Rehman A, Riaz ul H. 2004. Multiple metal resistance in the ciliate protozoan, Vorticella microstoma, isolated from industrial effluents and its potential in bioremediation of toxic wastes. Bull Environ Contam Toxicol 72:1046-51.
549. Diaz S, Amaro F, Rico D, Campos V, Benitez L, Martin-Gonzalez A, Hamilton EP, Orias E, Gutierrez JC. 2007. Tetrahymena metallothioneins fall into two discrete subfamilies. PLoS One 2:e291.
550. Piccinni E, Bertaggia D, Santovito G, Miceli C, Kraev A. 1999. Cadmium metallothionein gene of Tetrahymena pyriformis. Gene 234:51-59.
551. Parnell JJ, Rompato G, Latta LCt, Pfrender ME, Van Nostrand JD, He Z, Zhou J, Andersen G, Champine P, Ganesan B, Weimer BC. 2010. Functional biogeography as evidence of gene transfer in hypersaline microbial communities. PLoS One 5:e12919.
552. Butler A, Carter-Franklin JN. 2004. The role of vanadium bromoperoxidase in the biosynthesis of halogenated marine natural products. Nat Prod Rep 21:180-8.
553. Wang P, Matthews DE, VanEtten HD. 1992. Purification and characterization of cyanide hydratase from the phytopathogenic fungus Gloeocercospora sorghi. Arch Biochem Biophys 298:569-75.
554. Herrmann M, Zocher R, Haese A. 1996. Enniatin production by fusarium strains and its effect on potato tuber tissue. Appl Environ Microbiol 62:393-8.
555. Herrmann M, Zocher R, Haese A. 1996. Effect of disruption of the enniatin synthetase gene on the virulence of Fusarium avenaceum. Mol Plant Microbe Interact 9:226-32.
556. Lundqvist T, Rice J, Hodge CN, Basarab GS, Pierce J, Lindqvist Y. 1994. Crystal structure of scytalone dehydratase--a disease determinant of the rice pathogen, Magnaporthe grisea. Structure 2:937-44.
557. Haro R, Rodríguez-Navarro A. 2002. Molecular analysis of the mechanism of potassium uptake through the TRK1 transporter of Saccharomyces cerevisiae. Biochimica et Biophysica Acta (BBA) - Biomembranes 1564:114-122.
558. Merzendorfer H. 2011. The cellular basis of chitin synthesis in fungi and insects: Common principles and differences. European Journal of Cell Biology 90:759-769.
559. Roncero C. 2002. The genetic complexity of chitin synthesis in fungi. Current Genetics 41:367-378.
560. Yamamoto S, Harayama S. 1995. PCR amplification and direct sequencing of gyrB genes with universal primers and their application to the detection and taxonomic analysis of Pseudomonas putida strains. Appl Environ Microbiol 61:1104-9.
561. Bryant DA, Frigaard NU. 2006. Prokaryotic photosynthesis and phototrophy illuminated. Trends in Microbiology 14:488-496.
562. Liu GY, Nizet V. 2009. Color me bad: microbial pigments as virulence factors. Trends in Microbiology 17:406-413.

563. Ernst OP, Lodowski DT, Elstner M, Hegemann P, Brown LS, Kandori H. 2014. Microbial and Animal Rhodopsins: Structures, Functions, and Molecular Mechanisms. Chemical Reviews 114:126-163.
564. Walter MH, Strack D. 2011. Carotenoids and their cleavage products: Biosynthesis and functions. Natural Product Reports 28:663-692.
565. Warren MJ, Smith AG, Frankenberg-Dinkel N, Terry MJ. 2009. Synthesis and Role of Bilins in Photosynthetic Organisms, p 208-220, Tetrapyrroles doi:10.1007/978-0-387-78518-9_12. Springer New York.
566. Heyes DJ, Hunter CN. 2009. Biosynthesis of Chlorophyll and Barteriochlorophyll, p 235-249, Tetrapyrroles. Springer.
567. Chew AG, Bryant DA. 2007. Chlorophyll biosynthesis in bacteria: the origins of structural and functional diversity. Annu Rev Microbiol 61:113-29.
568. Takaichi S, Mochimaru M. 2007. Carotenoids and carotenogenesis in cyanobacteria: unique ketocarotenoids and carotenoid glycosides. Cell Mol Life Sci 64:2607-19.
569. Beale SI. 1993. Biosynthesis of phycobilins. Chemical Reviews 93:785-802.
570. Wiethaus J, Busch AWU, Dammeyer T, Frankenberg-Dinkel N. 2010. Phycobiliproteins in Prochlorococcus marinus: Biosynthesis of pigments and their assembly into proteins. European Journal of Cell Biology 89:1005-1010.
571. Fuhrman JA, Schwalbach MS, Stingl U. 2008. Proteorhodopsins: an array of physiological roles? Nat Rev Microbiol 6:488-94.
572. Slamovits CH, Okamoto N, Burri L, James ER, Keeling PJ. 2011. A bacterial proteorhodopsin proton pump in marine eukaryotes. Nature Communications 2:-.
573. White D. 2000. The physiology and biochemistry of prokaryotes. Oxford University Press, New York.
574. Ludwig B. 1987. Cytochrome c oxidase in prokaryotes. FEMS Microbiology Letters 46:41-56.
575. Frey M. 2002. Hydrogenases: hydrogen-activating enzymes. Chembiochem 3:153-60.
